# Dual-regioselective direct C(sp^2^)-arylation of unprotected β-enamino esters with 2-indolylmethanols catalyzed by Brønsted acid

**DOI:** 10.1039/d5ra05581d

**Published:** 2025-09-29

**Authors:** Qing-Chun Song, Pei-Hua Zhao, Yu-Xi Xing, Chen-Xin Bao, Ling-Yan Chen, Ya Li

**Affiliations:** a College of Chemistry and Chemical Engineering, Shanghai University of Engineering Science 333 Longteng Road Shanghai 201620 China; b The Key Laboratory for Chemical Biology of Fujian Province, Xiamen University Xiamen 361005 China lingyan.chen@sues.edu.cn

## Abstract

An efficient dual-regioselective strategy has been developed for the direct C(sp^2^)-arylation of *β*-enamino esters with 2-indolylmethanols, employing diphenyl phosphate as the catalyst without the need for amino group protection. 48 structurally diverse indole-enamino ester hybrids were synthesized in moderate to excellent yields (up to 98%). Notably, this approach effectively suppresses the competitive *N*-arylation byproduct formation commonly encountered in traditional approaches and represents the successful precise integration of *β*-enamino esters and indoles, offering a modular and efficient route to complex heterocyclic architectures with potential bioactivities.

## Abstract 2

An dual-regioselective strategy has been developed for the direct C(sp^2^)-arylation of *β*-enamino esters with 2-indolylmethanols, delivering 48 structurally diverse indole-enamino ester hybrids in moderate to excellent yields (up to 98%).

## Graphical abstract

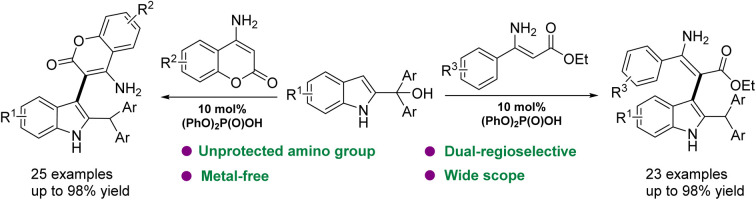

## 1. Introduction

**Fig. 1:**
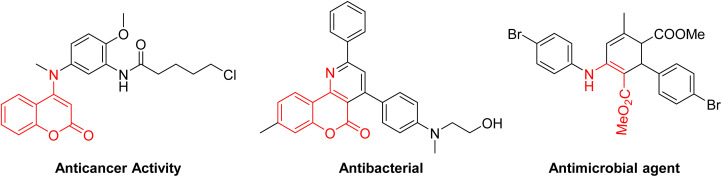
Bioactive compounds including *β*-enamino ester scaffolds.

**Scheme 1:**
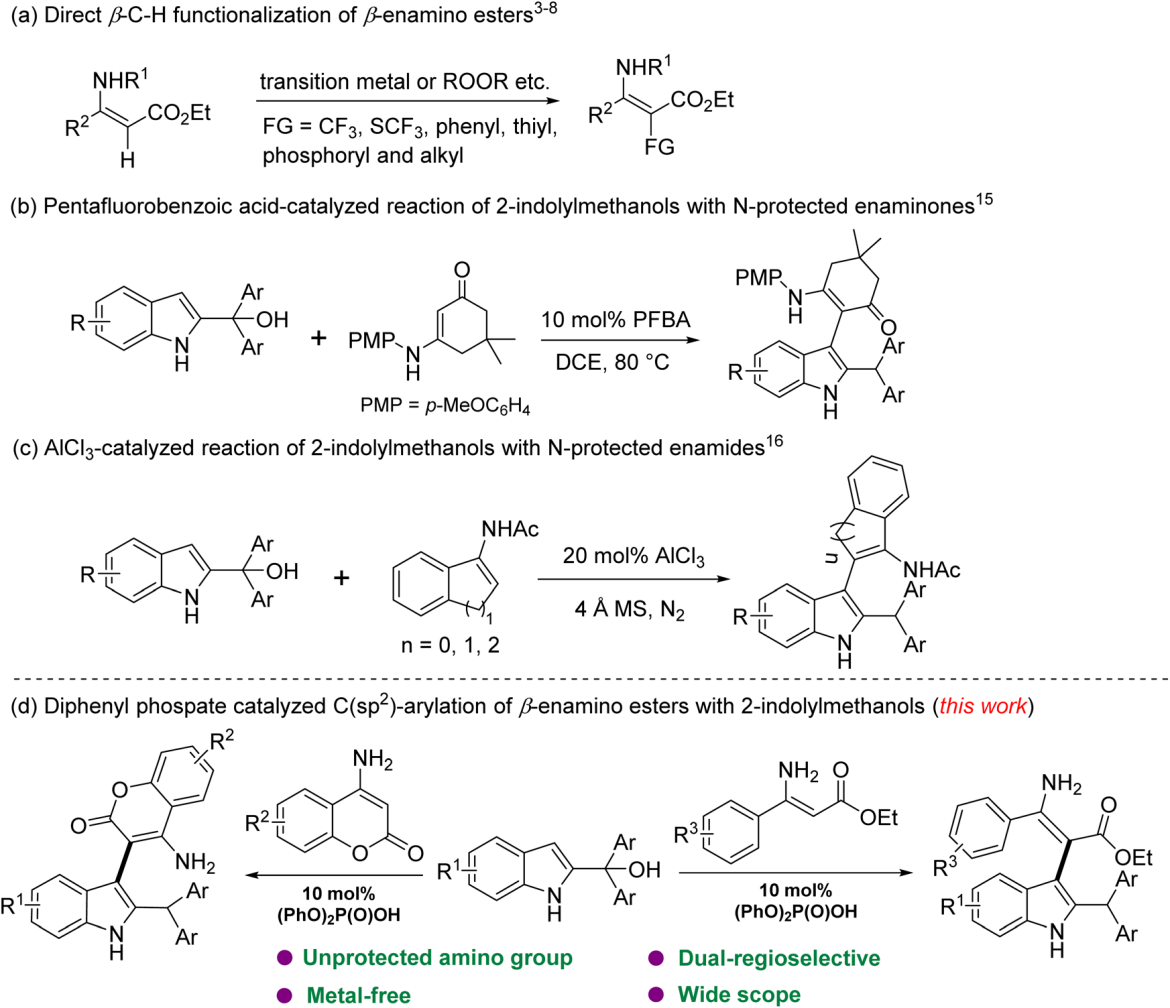
Synthetic strategies using *β*-enamino derivatives as substrates.

*β*-Enamino esters are structurally unique scaffolds that have attracted significant attention in drug discovery and bioactive molecule development (Fig. 1).^1^ As versatile *C*,*N*-dinucleophilic synthons, they are widely used to construct nitrogen-containing heterocycles with high application value.^2^ Recent advances have also enabled regioselective *β*-C–H functionalization (Scheme 1a), including trifluoromethylation,^3^ trifluoromethylthiolation,^4^ arylation,^5^ thiolation,^6^ phosphorylation,^7^ and *β*-ketoalkylation.^8^ Among these transformations, direct C(sp^2^)–H arylation of *β*-enamino esters has emerged as a key area of research. It usually utilizes electron-rich arenes,^9^*o*-silylaryl triflates,^10^ aryl boronic esters,^11^ and 3-aminoindazoles^12^ as arylating agents. Nevertheless, existing methods often face some limitations, including contamination from transition metals and the requirements for pre-protecting amino groups. Consequently, the development of milder reaction conditions for the direct C(sp^2^)-arylation of *β*-enamino esters remains a significant challenge in synthetic chemistry.

Indolylmethanols have proven to be versatile reactants for the synthesis of indole derivatives, as they can easily be converted into delocalized cation intermediates with carbocation resonance structures mediated by Brønsted or Lewis acids, followed by the nucleophilic substitution reactions^13^ and [3 + *n*] cycloadditions,^14^ affording benzylic site functionalized, C3-functionalized or multicyclic indole derivatives. Shi reported the reaction of 2-indolylmethanol with cyclic enaminones to access a series of C3-functionalized indole derivatives (Scheme 1b).^15^ Zhen demonstrated a highly efficient, AlCl_3_-catalyzed protocol enabling regioselective reactions between 2-indolylmethanols and enamides (Scheme 1c).^16^ However, these strategies still require protecting groups to manage the high reactivity of primary amines, and suppressing *N*-arylation side reactions remains a key challenge due to uncontrolled cascade processes arising from their inherent nucleophilicity. As a result, we envisioned that 2-indolylmethanols could be used as arylating agents in the C(sp^2^)-arylation with *β*-enamino esters. In continuation of our research on organocatalytic synthetic strategies,^17^ herein we report a Brønsted acid-catalyzed direct C(sp^2^)-arylation of *β*-enamino esters (including 4-aminocoumarins and acyclic derivatives) with 2-indolylmethanols (Scheme 1d). This method efficiently yields a series of diverse indole-enamine hybrids with good to excellent yields. The procedure is dual regioselective, operationally simple, avoids the need for amino group protection, and ensures the absence of *N*-arylation byproducts.

## 2. Results and discussion

Initially, we selected 2-indolylmethanol 1a and 4-aminocoumarin 2a (cyclic *β*-enamino ester) as model substrates in 1,2-dichloroethane (DCE) at room temperature (Table 1). Several Lewis acids were tested (entries 1–6), and Indium triflate [In(OTf)_3_] proved most effective, affording the desired product 3a in 76% yield (entry 5). The *N*-arylation byproduct was not observed. Considering the potential risks of metal residues and efficiency limitations of metal catalysts, we turned our attention to Brønsted acids (entries 7–11). Notably, diphenyl phosphate (C1) delivered 3a in 83% yield (entry 7), while other Brønsted acids including phenylphosphoric acid (C2), *p*-toluenesulfonic acid (TsOH), trifluoroacetic acid (TFA), and trifluoromethanesulfonic acid (TfOH) also provided moderate to good yields (entries 8–11, 57–81%). In order to further improve the reaction, different solvents were also investigated (entries 12–18). The halogenated solvents such as dichloromethane (DCM, entry 12) and chloroform (entry 13) could afford the desired product 3a in 88% and 85% yield, respectively. In contrast, other polar solvents such as tetrahydrofuran (THF), ethyl acetate, acetone and dimethylformamide (DMF) exhibited limited efficiency (<20%, entries 14–17). Toluene resulted in only a moderate yield (65%, entry 18). If the reaction temperature was increased to 40 °C, the reaction time could sharply shortened to 30 minutes and the yield reached 92% (entry 19). Remarkably, if the catalyst loading was reduced from 20 mol% to 10 mol%, the yield could remain (entry 20, 92%).

**Table 1:** Optimization of the reaction conditions^a^

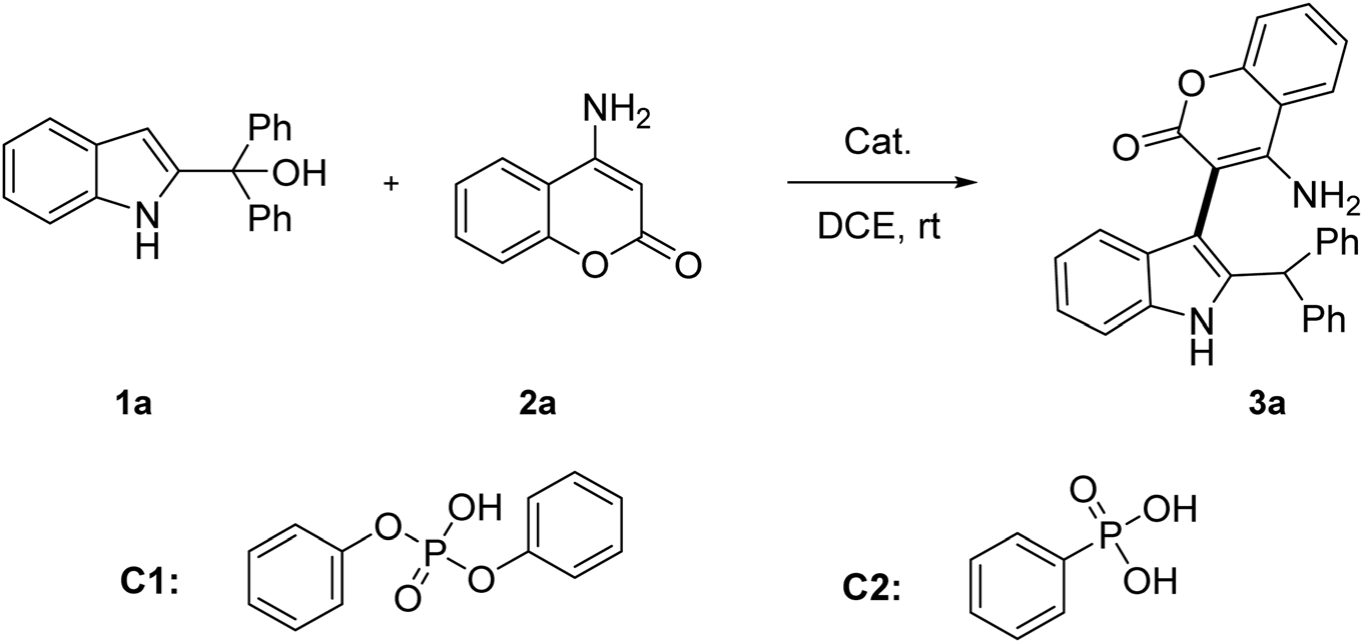			
Entry	Catalyst	Solvent	Yield^b^ (%)
1	ZnCl_2_	DCE	42
2	FeCl_3_	DCE	63
3	AlCl_3_	DCE	66
4	BF_3_·Et_2_O	DCE	70
5	In(OTf)_3_	DCE	76
6	Cu(OTf)_2_	DCE	70
7	C1	DCE	83
8	C2	DCE	57
9	TsOH	DCE	75
10	TFA	DCE	81
11	TfOH	DCE	78
12	C1	DCM	88
13	C1	CHCl_3_	85
14	C1	THF	10
15	C1	EtOAc	20
16	C1	Acetone	17
17	C1	DMF	13
18	C1	Toluene	62
19^c^^,^^e^	C1	DCM	92
20^c^^,^^d^^,^^e^	C1	DCM	92

a Unless otherwise noted, all reactions were conducted with 1a (0.2 mmol), 2a (0.24 mmol, 1.2 equiv.) and cat. (0.04 mmol, 20 mol%) in the solvent (2.0 mL) stirred at room temperature for 6 h.

b Isolated yield.

c At 40 °C.

d 10 mol% C1 (diphenyl phosphate) was used.

e Reaction time was shortened to 30 min.

With the optimal reaction conditions established, the scope of this reaction was then examined. The results were summarized in Table 2. A wide range of 2-indolylmethanols 1 could smoothly react with 4-aminocoumarin 2a, delivering the regioselective products 3. In detail, the influence of substituents on the indole ring of 2-indolylmethanols was examined. The reaction efficiency was largely insensitive to the electronic nature of the indole ring. 2-Indolylmethanols bearing either electron-donating or electron-withdrawing groups at C5 and C7 positions of the indole moiety generally afforded the corresponding products in excellent yields (90–98%, 3b–3i). Substituent effects on the benzyl ring were also investigated. *Para*-Substituted derivatives bearing methyl, fluoro, or chloro groups could maintain the yields above 83% (3j, 3l–m), while a strongly electron-donating *para*-methoxy group significantly reduced the yield to 56% (3k) probably due to less dehydration to form the carbocation intermediate. Steric effects were evaluated through *meta*- and *ortho*-methyl substitutions. While the *meta*-methyl analog (3m) maintained an 88% yield, the *ortho*-methyl derivative (3o) showed dramatically diminished reactivity (25% yield). Notably, *N*-benzyl group protection of the indole nitrogen atom still enabled efficient formation of 3p in 97% yield. To further examine the generality of the reaction, the scope of the reaction with respect to 4-aminocoumarin derivatives were also investigated. The electronic nature of the substituents of 4-aminocoumarins had noticeable effects on the outcome of the reaction. Substrates with electron-donating group substituent at C6 position afforded products with better yields than that with electron-withdrawing groups (3q–3r*vs.*3s–3u). Intriguingly, substituent at the C7 position exhibited different effects. Both the substrates with electron-donating group and electron-withdrawing group delivered the products in excellent yields (3v–3x). For the naphthalene-derived substrate, the yield of product 3y decreased to 43%, likely due to its unique aromatic system architecture. The distinct difference in electronic sensitivity between C6 and C7 substituents demonstrated the position-dependent nature of electronic effects in this system.

**Table 2:** Scope of the reaction between 4-aminocoumarins and 2-indolylmethanols^a^

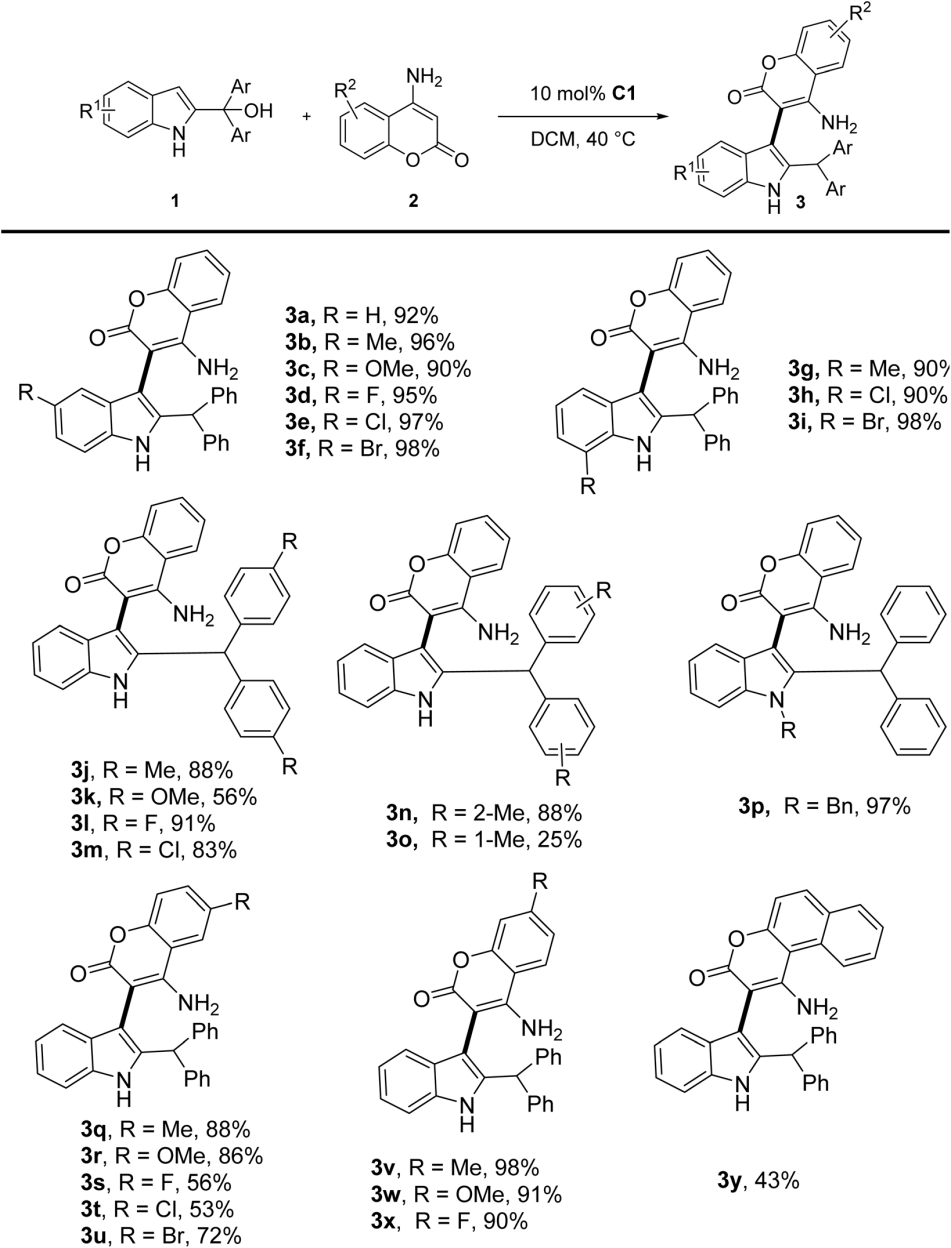

a Reaction conditions: 1 (0.2 mmol), 2a (0.24 mmol, 1.2 equiv.), C1 (0.02 mmol, 10 mol%) in DCM (2.0 mL) stirred at 40 °C for 30 min.

Since 4-aminocoumarins can be viewed as cyclic *β*-enamino esters, we next explored acyclic *β*-enamino esters as substrates to further broaden the reaction scope. To our delight, under the same conditions, the reaction of 2-indolylmethanols 1 with the acyclic *β*-enamino esters 4 also proceeded smoothly, leading to the corresponding product 5 in generally good to excellent yields (Table 3). For 2-indolylmethanols, regardless of the electronic properties and positions of the substituent on the indole ring (R^1^) (5a–5f) or benzyl aromatic ring (Ar) (5g–5j), the reaction pattern was similar to that with 4-aminocoumarins. It demonstrated the wide applicability of this regioselective reaction. For acyclic *β*-enamino esters, the varied substituents on different positions of β-phenyl ring afforded the desired product in 80–91% yield (5k–5r). Even strongly electron-withdrawing groups such as –CF_3_ and –NO_2_ were well tolerated (5p and 5q, 86% and 80% yield). Other aromatic heterocyclic substituents such as pyridyl and thiopheneyl also had good performance, giving the corresponding product (5s and 5t) in 94% and 90% yield, respectively. If the alkyl group (methyl) was used instead of phenyl group, the reaction yield still remained high (5u–5v). Different ester moiety proved good efficiency as well (5v, 83%). Additionally, the successful replacement of the ester group with a cyano (–CN) group in the acyclic *β*-enamino system could result in nitrile derivative 5w in 92% yield. The results presented the versatility of the catalytic system in accommodating both cyclic and acyclic *β*-enamino ester frameworks, significantly expanding the synthetic utility of this methodology for constructing diverse enamine-indole hybrid architectures.

**Table 3:** Scope of the reaction between acyclic *β*-enamino esters and 2-indolymethanols^a^

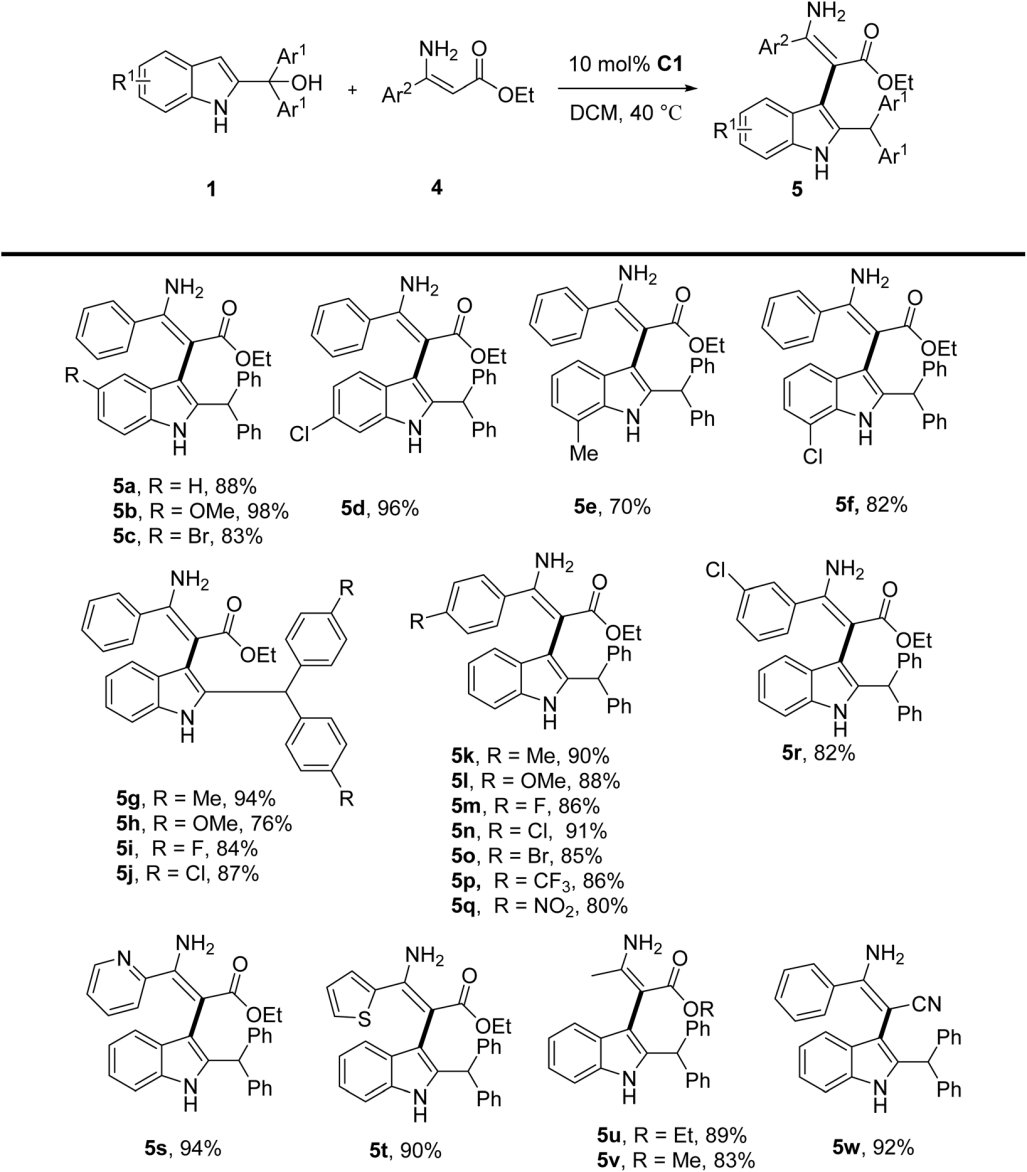

a Reaction conditions: all reactions were carried out using 1 (0.2 mmol), 4a (0.24 mmol, 1.2 equiv.), C1 (0.02 mmol, 10 mol%) in DCM (2.0 mL) stirred at 40 °C for 30 min.

**Scheme 2:**
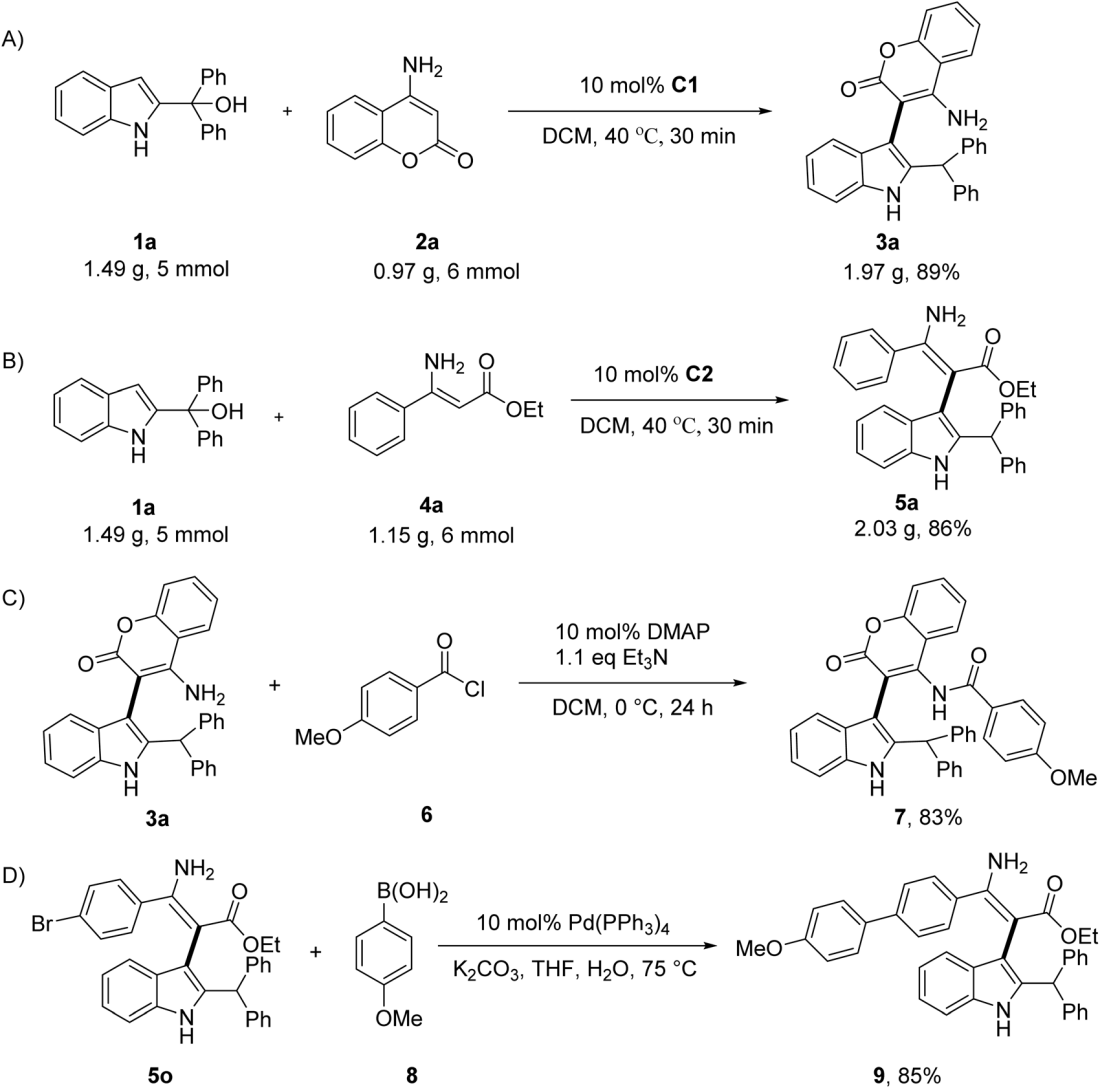
Gram-scale reactions and synthetic transformations.

To demonstrate the practical utility of this reaction, gram-scale syntheses and chemical transformations were conducted. As displayed in Scheme 2A and 2B, gram-scale reactions were performed with 5 mmol of 1a with 6 mmol of 2a and 4a, affording the corresponding products 3a and 5a without significant losses of yield compared with the small-scale reaction (89% and 86% yield, respectively). Several transformations were also performed (Scheme 2C and 2D). Treatment of 3a with *p*-methoxybenzoyl chloride under mild acylation conditions converted the primary amine to amide 7 in 83% yield (Scheme 2C). Suzuki–Miyaura coupling of 5o with 4-methoxyphenylboronic acid smoothly delivered biaryl product 9 in 85% yield (Scheme 2D). These transformations demonstrated compatibility of the products and highlighted their value as versatile intermediates for complex molecule synthesis.

**Scheme 3:**
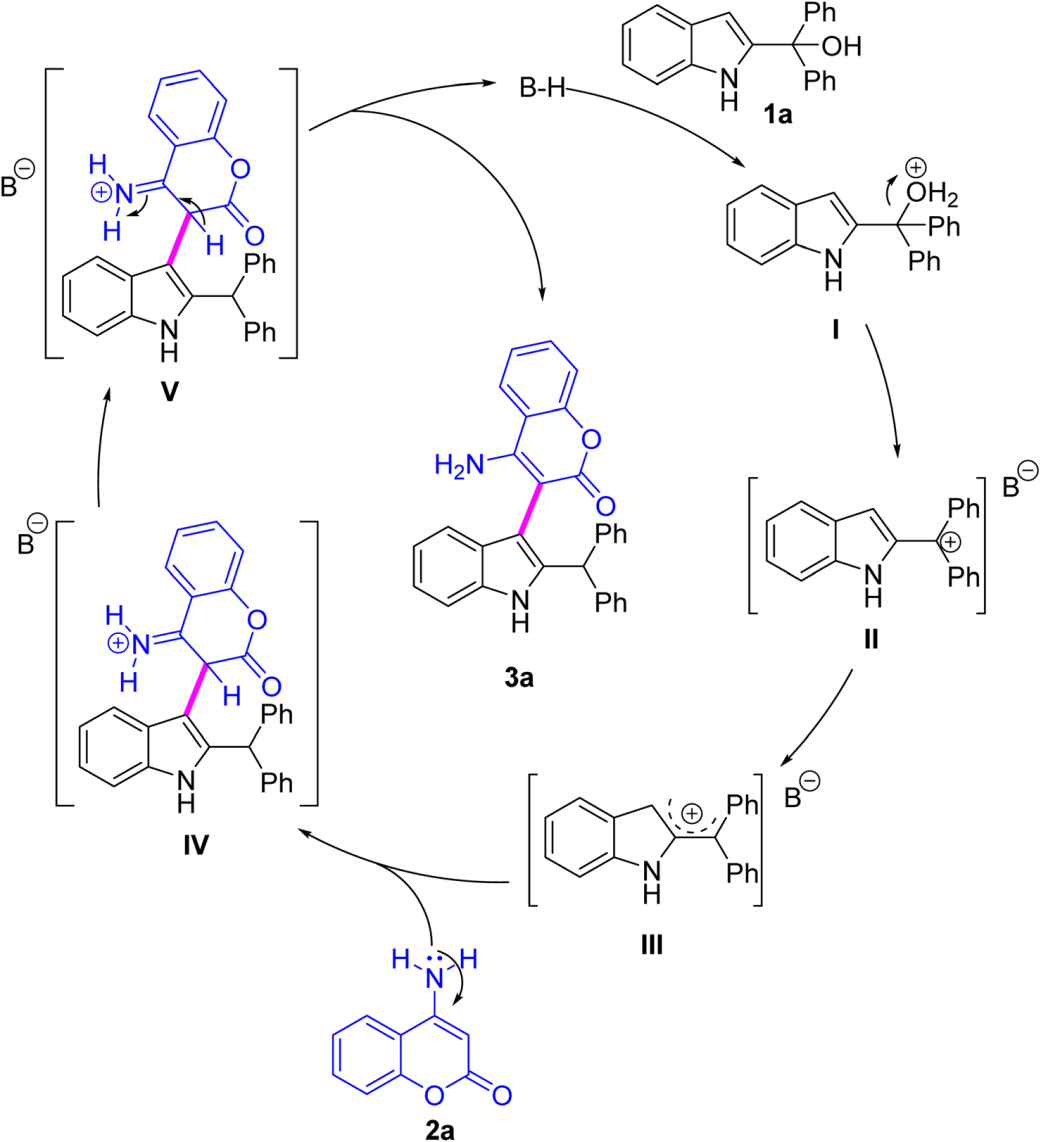
Plausible mechanism of the reaction.

Based on experimental evidence and literature precedents,^13^ we propose a plausible Brønsted acid-catalyzed mechanism for the reaction of 2-indolylmethanol with *β*-enamino ester (Scheme 3). The catalytic cycle begins with Brønsted acid protonation of 2-indolylmethanol 1a, generating an oxonium intermediate (I). Subsequent dehydration forms the highly reactive carbocation intermediate (II), which undergoes extensive resonance stabilization to yield the electrophilic species (III). The *β*-enamino ester 2a then undergoes nucleophilic attack at the electrophilic carbon of III, establishing the key *C*–C bond and forming iminium intermediate (IV). The inherent instability of IV promotes deprotonation at the activated site, simultaneously restoring the aromatic indole system and releasing the final product (3a). This step also regenerates the Brønsted acid catalyst, completing the catalytic cycle.

## 3. Conclusions

In summary, we have developed an efficient, dual-regioselective organocatalytic strategy for the direct C(sp^2^)-arylation of *β*-enamino esters (including 4-aminocoumarins and acyclic derivatives) with 2-indolylmethanols using diphenyl phosphate as the catalyst, without the need for amino group protection. This transformation exhibited broad substrate scope, accommodating a wide range of 2-indolylmethanols and *β*-enamino esters to deliver structurally diverse indole-enamine hybrids in good to excellent yields (48 examples, up to 98% yield). The protocol demonstrated excellent scalability in gram-scale reactions and offered versatile opportunities for further synthetic transformations, enhancing its practical utility. Notably, the reaction proceeded with high efficiency and complete dual regioselectivity, providing a robust and user-friendly method for accessing these valuable heterocyclic frameworks. Further investigations on asymmetric variants of this transformation are in progress.

## Conflicts of interest

There are no competing interest to declare.

## Supplementary Material

RA-015-D5RA05581D-s001

## Data Availability

The data supporting this article have been included as part of the SI. Supplementary information: experimental procedures and NMR spectra. See DOI: https://doi.org/10.1039/d5ra05581d.
