# Supplementary material for: Dual-regioselective direct C(sp2)-arylation of unprotected β-enamino esters with 2-indolylmethanols catalyzed by Brønsted acid
Source: RSC Adv. 2025 Sep 29;15(43):35782–9. doi: 10.1039/d5ra05581d (PMC12482906; doi:10.1039/d5ra05581d)
Supplement: RA-015-D5RA05581D-s001 [file RA-015-D5RA05581D-s001.pdf]

Supplementary Information (SI) for RSC Advances.
This journal is © The Royal Society of Chemistry 2025

Supporting Information

for

Dual-Regioselective Direct C(sp^2)-Arylation of Unprotected β-Enamino Esters with 2-Indolylmethanols Catalyzed by Brønsted Acid

Qing-Chun Song,[a,b] Pei-Hua Zhao,[a] Yu-Xi Xing,[a] Chen-Xin Bao,[a] Ling-Yan Chen,*[a,b] Ya Li[a]

[a]*College of Chemistry and Chemical Engineering, Shanghai University of Engineering Science, 333 Longteng Road, Shanghai 201620, China*

[b]*The key Laboratory for Chemical Biology of Fujian Province, Xiamen University, Xiamen 361005, China*

E-mail: lingyan.chen@sues.edu.cn

Contents

1. General Information S2

2. Synthesis of 2-Indolylmethanols S2

3. Synthesis of 4-aminocoumarins S3

4. Synthesis of Acyclic β-Enamine Esters S3

5. General Procedure for the Preparation of 3 .... S4

6. General Procedure for the Preparation of 5 .... S17

7. Procedure for the Gram-Scale Synthesis of 3a and 5a S31

8. References S31

9. Copies of NMR Spectra S32

1. General Information

Unless otherwise stated, all reagents were purchased from commercial suppliers and used without further purification. All reactions were carried out at air atmosphere using reaction tubes and were monitored through thin layer chromatography (TLC) on silica gel - precoated glass plates. Reactions were monitored by thin layer chromatography (TLC) using UV light to visualize the course of reaction. Column chromatography was performed using silica gel (300−400 mesh). Melting points were recorded on an Electrothermal digital melting point apparatus. ^{1}H, ^{13}C and ^{19}F NMR spectra were recorded on Bruker 400 MHz spectrometer in $CDCl_3$ (7.26 ppm) or DMSO-d_6 (2.50 and 3.33 ppm) or tetramethylsilane. Carbon chemical shifts were internally referenced to the deuterated solvent signals in $CDCl_3$ (77.00 ppm) or DMSO-d_6 (40.0 ppm). The chemical shifts are expressed in ppm and coupling constants are given in Hz. Data for ^{1}H NMR are recorded as follows: chemical shift (δ, ppm), multiplicity (s = singlet; d = doublet; t = triplet; q = quarter; p = pentet; m = multiplet; br = broad), coupling constant (Hz), integration. Data for ^{13}C NMR are reported in terms of chemical shift (δ, ppm). Data for ^{19}F NMR are reported in terms of chemical shift (δ, ppm). Products were identified by comparison to spectral data reported in the literature. High-resolution mass spectra were recorded on a time-of-flight (TOF) mass spectrometer with an electrospray ionization (ESI) source.

2. Synthesis of 2-Indolylmethanols[1]

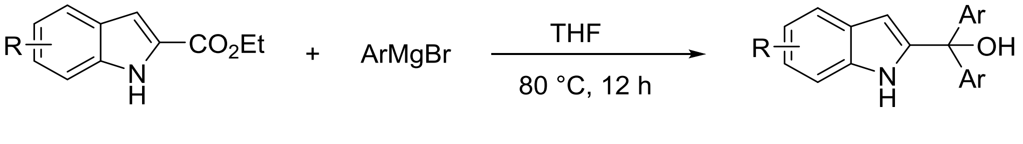

Under argon atmosphere, arylmagnesium bromide (30 mL, 1.0 M in THF, 5.0 equiv.) was added to a Schlenk bottle. Then, the solution of ethyl 1H-indole-2-carboxylate (6.0 mmol, 1.0 equiv.) in anhydrous THF (1 mol/mL) was added dropwise to the Schlenk bottle at 0 °C. Subsequently, the reaction mixture was stirred at 80°C overnight. After the completion of the reaction indicated by TLC, the reaction mixture was quenched by saturated ammonium

chloride solution and extracted by ethyl acetate for three times. The combined organic layers were dried and concentrated under reduced pressure to give a residue. Finally, the residue was purified by flash column chromatography on silica gel to afford pure 2-indolylmethanols

.

3. Synthesis of 4-aminocoumarins[2]

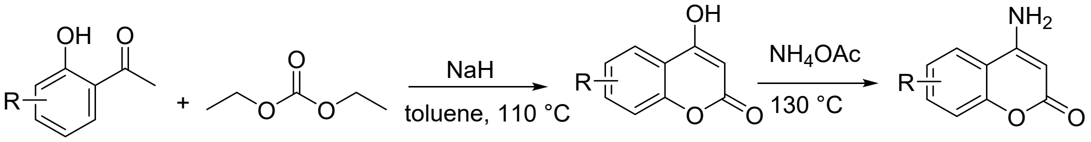

2'-Hydroxyacetopheone (10 mmol) was dissolved in 25 mL anhydrous toluene. NaH (100 mmol) was slowly added. Diethyl carbonate (15 mmol) in 5 mL anhydrous toluene was added dropwise when hydrogen evolution was ceased. The mixture was stirred at 110 ºC overnight. The mixture was filtered and washed once with toluene to obtain solid. The solid was slowly added to ice-cold water (30 mL), then acidified with 2 N HCl (aq.) until no precipitate was formed. The mixture was filtered to obtain crude product, which was washed with water and ethyl acetate to obtain pure.

4-Hydroxycoumarin. A neat mixture of 4-hydroxycoumarin (1 mmol) and ammonium acetate (20 mmol) was stirred at 130 ºC overnight. The mixture was then poured into water (20 mL) and stirred for 10 min. The resulting mixture was filtered to obtain crude product, which was washed with water and ethyl acetate to obtain pure 4-aminocoumarin.

4. Synthesis of Acyclic β-Enamine Esters[3]

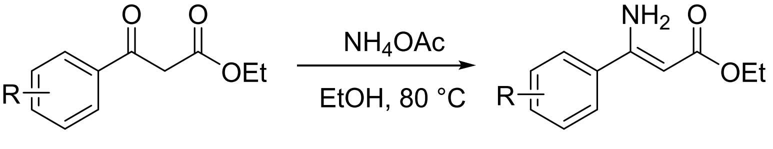

A mixture of β-keto ester (6.0 mmol, 1.0 equiv.) and ammonium acetate (12.0 mmol, 2.0 equiv.) in absolute ethanol (10 mL) was refluxed in a 100 mL round-bottom flask for 24 hours with reaction progress monitored by TLC. Upon completion, the solvent was removed

under reduced pressure, and the crude residue was dissolved in ethyl acetate (30 mL). The organic phase was sequentially washed with deionized water (3 × 20 mL) and saturated NaCl solution (3 × 20 mL), dried over anhydrous Na_2SO_4, filtered, and concentrated in vacuo. Purification of the crude product by silica gel column chromatography afforded the target acyclic β-enamine ester .

5. General Procedure for the Preparation of 3

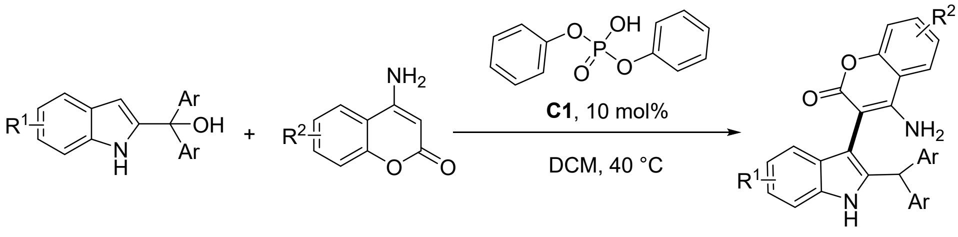

1 **2** **3**

An oven-dried 25 mL Schlenk tube was charged with 2-indolylmethanol **1** (0.2 mmol, 1.0 equiv.), 4-aminocoumarin **2** (0.24 mmol, 1.2 equiv.), and **C1** (0.02 mmol, 0.1 equiv.) in DCM (2 mL). The reaction mixture was stirred at 40 °C until the full consumption of 2-indolylmethanol **1** (typically, about 30 min). After the reaction was then quenched by saturated $NaHCO_3$, the organic layer was separated, and the aqueous phase was extracted with CH_2Cl_2 (3×10 mL). The combined organic layers were washed by saturated NaCl and dried over anhydrous Na_2SO_4. After filtration, the solution was concentrated and the crude product was purified by flash column chromatography on silica gel using gradient elution (petroleum ether/ethyl acetate = 7:1-3:1) to afford the target product **3a-3y**.

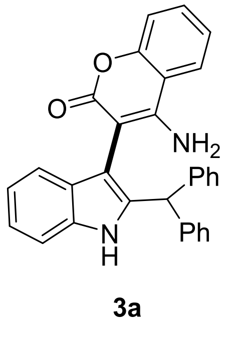

3a

4-Amino-3-(2-benzhydryl-1H-indol-3-yl)-2H-chromen-2-one (3a). White solid (81.4 mg, 92%); m.p. 166.4-166.8°C; ^1H NMR (400 MHz, $CDCl_3$) δ 7.87 (s, 1H), 7.51 (t, J = 8.8 Hz, 2H), 7.31 (d, J = 8.0 Hz, 1H), 7.26 – 7.18 (m, 5H), 7.17 – 7.12 (m, 5H), 7.11 – 7.03 (m, 8H), 6.98 (t, J = 7.2 Hz, 1H), 5.73 (s, 1H), 4.79 (s, 2H). ^{13}C NMR (100 MHz, $CDCl_3$) δ 162.02,

153.22, 151.34, 141.99, 141.56, 139.69, 135.63, 131.52, 129.01, 128.63, 128.56, 128.32, 127.35, 126.94, 126.53, 123.38, 121.87, 121.60, 119.95, 119.18, 117.28, 114.02, 111.14, 104.32, 92.53, 49.13; HRMS (ESI-TOF) m/z: $[M+H]^+$ Calcd for $C_{30}H_{23}N_2O_2$ 443.1754, Found 443.1748.

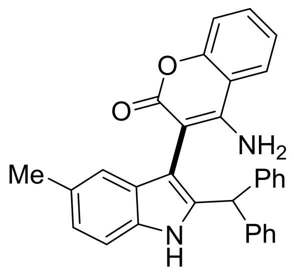

3b

4-Amino-3-(2-benzhydryl-5-methyl-1H-indol-3-yl)-2H-chromen-2-one (3b). White solid (87.1 mg, 96%); m.p. 162.6-162.9 °C; ^{1}H NMR (400 MHz, $CDCl_3$) δ 7.84 (s, 1H), 7.50 (t, *J* = 7.8 Hz, 1H), 7.39 (d, *J* = 8.0 Hz, 1H), 7.30 (d, *J* = 8.4 Hz, 1H), 7.25 – 7.19 (m, 4H), 7.19 – 7.03 (m, 9H), 6.93 (d, *J* = 8.4 Hz, 1H), 5.73 (s, 1H), 4.78 (s, 2H), 2.34 (s, 3H). ^{13}C NMR (100 MHz, $CDCl_3$) δ 161.87, 153.31, 150.99, 142.13, 141.62, 139.79, 134.04, 131.51, 129.42, 129.01, 128.62, 128.34, 127.58, 126.88, 126.55, 123.52, 123.30, 121.28, 118.86, 117.40, 114.00, 110.86, 103.86, 93.16, 49.13, 21.44; HRMS (ESI-TOF) m/z: $[M+H]^+$ Calcd for $C_{31}H_{25}N_2O_2$ 457.1911, Found 457.1907.

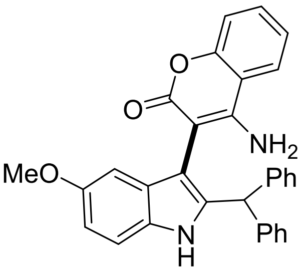

3c

4-Amino-3-(2-benzhydryl-5-methoxy-1H-indol-3-yl)-2H-chromen-2-one (3c). White solid (87.4 mg, 90%); m.p. 211.7-213.2°C; ^{1}H NMR (400 MHz, $CDCl_3$) δ 7.92 (s, 1H), 7.51 (t, *J* = 7.6 Hz, 1H), 7.45 (d, *J* = 8.0 Hz, 1H), 7.31 (d, *J* = 8.4 Hz, 1H), 7.25 – 7.02 (m, 12H), 6.77 (d, *J* = 8.8 Hz, 1H), 6.70 (s, 1H), 5.73 (s, 1H), 4.86 (s, 2H), 3.71 (s, 3H). ^{13}C NMR (100 MHz, $CDCl_3$) δ 161.95, 154.41, 153.24, 151.15, 142.03, 141.55, 140.43, 131.53, 130.84, 128.96, 128.60, 128.57, 128.34, 127.94, 126.85, 126.56, 123.34, 121.49, 117.32, 113.99,

111.97, 111.90, 104.16, 101.21, 92.82, 55.89, 49.20; HRMS (ESI-TOF) m/z: $[M+H]^+$ Calcd for $C_{31}H_{25}N_2O_3$ 473.1860, Found 473.1858.

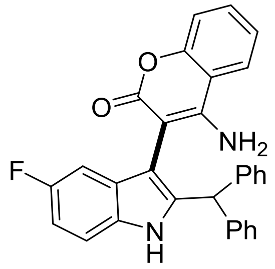

3d

4-Amino-3-(2-benzhydryl-5-fluoro-1H-indol-3-yl)-2H-chromen-2-one (3d). Pale yellow solid (87.2 mg, 95%); m.p. 157.1-158.5°C; ^{1}H NMR (400 MHz, $CDCl_3$) δ 7.97 (s, 1H), 7.53 (t, *J* = 7.6 Hz, 1H), 7.45 (d, *J* = 7.2 Hz, 1H), 7.32 (d, *J* = 8.0 Hz, 1H), 7.25 – 7.15 (m, 8H), 7.11 (d, *J* = 7.6 Hz, 3H), 7.09 – 7.02 (m, 1H), 6.89 – 6.80 (m, 2H), 5.73 (s, 1H), 4.81 (s, 2H). ^{13}C NMR (100 MHz, $CDCl_3$) δ 161.85, 158.07 (d, *J* = 234.1 Hz), 153.25, 151.26, 141.81, 141.26, 132.07, 131.74, 128.96, 128.71, 128.53, 128.43, 127.95 (d, *J* = 9.6 Hz), 127.02, 126.68, 123.46, 121.47, 117.40, 113.85, 111.84 (d, *J* = 9.4 Hz), 110.16 (d, *J* = 26.1 Hz), 104.53 (d, *J* = 4.3 Hz), 104.25, 104.01, 92.30, 49.21. ^{19}F NMR (376 MHz, $CDCl_3$) δ -123.38 (1F); HRMS (ESI-TOF) m/z: $[M+H]^+$ Calcd for $C_{30}H_{22}FN_2O_2$ 461.1660, Found 461.1652.

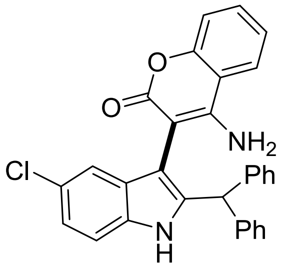

3e

4-Amino-3-(2-benzhydryl-5-chloro-1H-indol-3-yl)-2H-chromen-2-one (3e). Pale yellow solid (92.1 mg, 97%); m.p. 139.9-141.2°C; ^{1}H NMR (400 MHz, $CDCl_3$) δ 8.02 (s, 1H), 7.54 (t, *J* = 7.8 Hz, 1H), 7.43 (d, *J* = 8.0 Hz, 1H), 7.33 (d, *J* = 8.4 Hz, 1H), 7.26 – 7.15 (m, 9H), 7.12 (d, *J* = 7.2 Hz, 3H), 7.08 (d, *J* = 8.0 Hz, 2H), 5.74 (s, 1H), 4.77 (s, 2H). ^{13}C NMR (100 MHz, $CDCl_3$) δ 161.80, 153.28, 151.25, 141.70, 141.54, 141.14, 133.97, 131.81, 128.96, 128.74, 128.53, 128.47, 127.08, 126.74, 125.86, 123.48, 122.22, 121.39, 118.55, 117.46, 113.79, 112.23, 104.13, 92.22, 49.19; HRMS (ESI-TOF) m/z: $[M+H]^+$ Calcd for

$C_{30}H_{22}ClN_2O_2$ 477.1365, Found 477.1362.

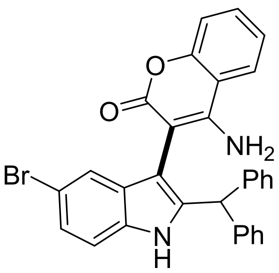

3f

4-Amino-3-(2-benzhydryl-5-bromo-1H-indol-3-yl)-2H-chromen-2-one (3f). Pale yellow solid (102.3 mg, 98%); m.p. 149.7-150.3°C; ^{1}H NMR (400 MHz, $CDCl_3$) δ 8.03 (s, 1H), 7.54 (t, J = 7.8 Hz, 1H), 7.44 – 7.38 (m, 2H), 7.34 (d, J = 8.4 Hz, 1H), 7.25 – 7.15 (m, 9H), 7.13 (d, J = 7.6 Hz, 3H), 7.07 (d, J = 8.4 Hz, 1H), 5.75 (s, 1H), 4.75 (s, 2H). ^{13}C NMR (100 MHz, $CDCl_3$) δ 161.73, 153.31, 151.18, 141.70, 141.41, 141.12, 134.26, 131.83, 129.16, 128.98, 128.77, 128.54, 128.48, 127.10, 126.75, 124.81, 123.47, 121.63, 121.30, 117.50, 113.77, 113.47, 112.68, 104.03, 92.30, 49.17; HRMS (ESI-TOF) m/z: $[M+H]^+$ Calcd for $C_{30}H_{22}BrN_2O_2$ 521.0859, Found 521.0852.

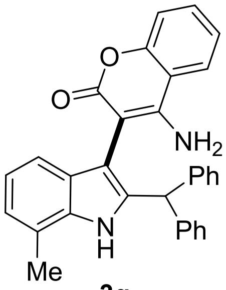

3g

4-Amino-3-(2-benzhydryl-6-methyl-1H-indol-3-yl)-2H-chromen-2-one (3g). Pale yellow solid (81.8 mg, 90%); m.p. 232.9-233.0°C; ^{1}H NMR (500 MHz, $CDCl_3$) δ 7.79 (s, 1H), 7.44 (t, J = 6.0 Hz, 1H), 7.33 (d, J = 6.4 Hz, 1H), 7.25 – 7.18 (m, 5H), 7.15 – 7.01 (m, 8H), 6.93 – 6.85 (m, 2H), 5.73 (s, 1H), 4.79 (s, 2H), 2.32 (s, 3H). ^{13}C NMR (126 MHz, $CDCl_3$) δ 161.89, 153.28, 151.00, 142.12, 141.59, 139.21, 135.30, 131.50, 129.00, 128.65, 128.61, 128.34, 126.91, 126.84, 126.55, 123.28, 122.67, 121.25, 120.36, 120.29, 117.37, 117.01, 113.99, 105.04, 93.20, 49.16, 16.44; HRMS (ESI-TOF) m/z: $[M+H]^+$ Calcd for $C_{31}H_{25}N_2O_2$ 457.1911, Found 457.1903.

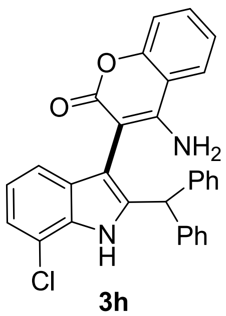

3h

4-Amino-3-(2-benzhydryl-6-chloro-1H-indol-3-yl)-2H-chromen-2-one(3h). White solid (86.6 mg, 90%); m.p. 155.4-155.9°C; ^{1}H NMR (400 MHz, $CDCl_3$) δ 8.06 (s, 1H), 7.46 (t, *J* = 7.8 Hz, 1H), 7.33 (d, *J* = 8.0 Hz, 1H), 7.25 – 7.17 (m, 5H), 7.17 – 7.09 (m, 7H), 7.07 (d, *J* = 7.6 Hz, 2H), 6.90 (t, *J* = 7.6 Hz, 1H), 5.71 (s, 1H), 4.74 (s, 2H). ^{13}C NMR (100 MHz, $CDCl_3$) δ 161.74, 153.32, 151.19, 141.67, 141.03, 140.54, 132.99, 131.80, 128.91, 128.77, 128.59, 128.50, 127.12, 126.76, 123.42, 121.50, 121.22, 121.00, 117.88, 117.50, 116.59, 113.81, 105.67, 92.63, 49.19; HRMS (ESI-TOF) m/z: $[M+H]^+$ Calcd for $C_{30}H_{22}ClN_2O_2$ 477.1365, Found 477.1360.

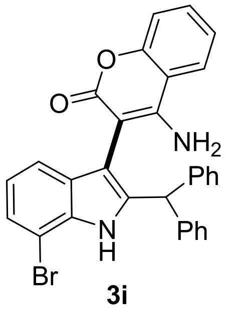

3i

4-Amino-3-(2-benzhydryl-6-bromo-1H-indol-3-yl)-2H-chromen-2-one(3i). White solid (103.4 mg, 98%); m.p. 164.3-166.3°C; ^{1}H NMR (500 MHz, $CDCl_3$) δ 8.05 (s, 1H), 7.49 (t, *J* = 6.2 Hz, 1H), 7.40 (d, *J* = 6.4 Hz, 1H), 7.25 – 7.20 (m, 6H), 7.18 – 7.12 (m, 7H), 7.10 – 7.04 (m, 1H), 6.85 (t, *J* = 6.2 Hz, 1H), 5.73 (s, 1H), 4.87 (s, 2H). ^{13}C NMR (126 MHz, $CDCl_3$) δ 161.83, 153.25, 151.37, 141.63, 141.02, 140.37, 134.38, 131.77, 128.86, 128.72, 128.56, 128.49, 127.08, 126.75, 124.39, 123.41, 121.41, 121.35, 118.43, 117.37, 113.80, 105.85, 104.66, 92.39, 49.19; HRMS (ESI-TOF) m/z: $[M+H]^+$ Calcd for $C_{30}H_{22}BrN_2O_2$ 521.0859, Found 521.0854.

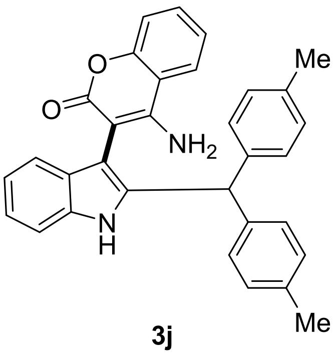

3j

4-Amino-3-(2-(di-p-tolylmethyl)-1H-indol-3-yl)-2H-chromen-2-one(3j). White solid (83.6 mg, 88%); m.p. 264.2-266.2°C; ^{1}H NMR (400 MHz, DMSO-d_6) δ 10.82 (s, 1H), 8.03 (d, J = 8.0 Hz, 1H), 7.57 (t, J = 7.6 Hz, 1H), 7.39 (d, J = 8.4 Hz, 1H), 7.35 – 7.25 (m, 2H), 7.12 (dd, J = 19.2, 8.0 Hz, 5H), 7.06 – 6.98 (m, 5H), 6.93 (t, J = 7.4 Hz, 1H), 5.46 (s, 1H), 2.21 (s, 3H), 2.15 (s, 3H). ^{13}C NMR (100 MHz, DMSO-d_6) δ 160.96, 152.83, 152.28, 139.54, 139.40, 136.63, 135.16, 135.13, 131.41, 128.82, 128.77, 128.63, 128.41, 127.40, 123.34, 123.09, 120.66, 118.52, 118.48, 116.41, 114.53, 111.37, 104.56, 90.13, 48.05, 20.54, 20.47; HRMS (ESI-TOF) m/z: $[M+H]^+$ Calcd for $C_{32}H_{27}N_2O_2$ 471.2067, Found 471.2057.

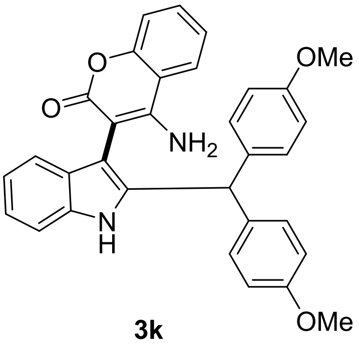

3k

4-Amino-3-(2-(bis(4-methoxyphenyl)methyl)-1H-indol-3-yl)-2H-chromen-2-one (3k). White solid (56.3 mg, 56%); m.p. 183.0-184.4°C; ^{1}H NMR (400 MHz, $CDCl_3$) δ 7.92 (s, 1H), 7.52 (t, J = 7.4 Hz, 1H), 7.45 (d, J = 7.6 Hz, 1H), 7.32 (d, J = 8.0 Hz, 1H), 7.22 (dd, J = 14.4, 7.6 Hz, 2H), 7.15 (t, J = 9.0 Hz, 3H), 7.10 (t, J = 7.4 Hz, 1H), 7.05 – 6.99 (m, 3H), 6.76 (d, J = 8.8 Hz, 2H), 6.68 (d, J = 8.4 Hz, 2H), 5.63 (s, 1H), 4.77 (s, 2H), 3.70 (s, 3H), 3.65 (s, 3H). ^{13}C NMR (100 MHz, $CDCl_3$) δ 161.91, 158.42, 158.07, 153.24, 150.96, 140.44, 135.59, 134.40, 133.91, 131.47, 129.94, 129.54, 127.46, 123.36, 121.82, 121.36, 119.94, 119.12, 117.34, 114.08, 113.94, 113.68, 111.11, 104.02, 93.16, 55.19, 55.12, 47.70; HRMS (ESI-TOF) m/z: $[M+H]^+$ Calcd for $C_{32}H_{27}N_2O_4$ 503.1966, Found 503. 1957.

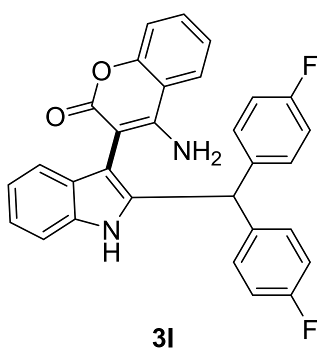

3l

4-Amino-3-(2-(bis(4-fluorophenyl)methyl)-1H-indol-3-yl)-2H-chromen-2-one (3l). White solid (87.4 mg, 91%); m.p. 160.3-161.2°C; ^{1}H NMR (400 MHz, $CDCl_3$) δ 7.93 (s, 1H), 7.54 (t, J = 7.8 Hz, 1H), 7.49 (d, J = 8.0 Hz, 1H), 7.32 (d, J = 7.6 Hz, 1H), 7.24 – 7.16 (m, 5H), 7.11 (t, J = 7.6 Hz, 1H), 7.08 – 6.99 (m, 3H), 6.91 (t, J = 8.6 Hz, 2H), 6.84 (t, J = 8.6 Hz, 2H), 5.73 (s, 1H), 4.87 (s, 2H). ^{13}C NMR (100 MHz, $CDCl_3$) δ 162.87 (d, J = 30.4 Hz), 161.99, 160.42 (d, J = 30.0 Hz), 153.19, 151.18, 139.35, 137.65, 137.62, 137.10, 137.07, 135.70, 131.76, 130.55, 130.47, 130.04, 129.96, 127.12, 123.54, 122.15, 121.39, 120.19, 119.19, 117.41, 115.37 (dd, J = 26.5, 21.5 Hz), 113.85, 111.30, 104.44, 92.61, 47.70. ^{19}F NMR (376 MHz, $CDCl_3$) δ -115.40 (t, J = 13.9 Hz, 1F), -115.79 (t, J = 13.9 Hz, 1F); HRMS (ESI-TOF) m/z: $[M+H]^+$ Calcd for $C_{30}H_{21}F_2N_2O_2$ 479.1566, Found 479.1558.

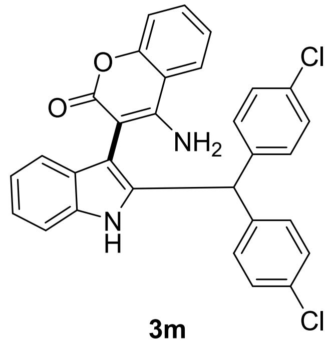

3m

4-Amino-3-(2-(bis(4-chlorophenyl)methyl)-1H-indol-3-yl)-2H-chromen-2-one (3m). White solid (85.5 mg, 83%); m.p. 162.4-163.1°C; ^{1}H NMR (400 MHz, $CDCl_3$) δ 7.93 (s, 1H), 7.55 (t, J = 7.8 Hz, 1H), 7.44 (d, J = 8.0 Hz, 1H), 7.35 (d, J = 8.0 Hz, 1H), 7.28 (d, J = 3.2 Hz, 2H), 7.25 – 7.19 (m, 5H), 7.18 – 7.11 (m, 3H), 7.10 – 7.01 (m, 3H), 5.75 (s, 1H), 4.84 (s, 2H). ^{13}C NMR (100 MHz, $CDCl_3$) δ 161.84, 153.26, 150.93, 140.15, 139.59, 138.72, 135.79, 133.17, 132.68, 131.84, 130.37, 129.87, 128.89, 128.60, 127.02, 123.57, 122.34, 121.15, 120.36, 119.31, 117.56, 113.81, 111.34, 104.72, 92.88, 47.96; HRMS (ESI-TOF)

m/z: $[M+H]^+$ Calcd for $C_{30}H_{21}Cl_2N_2O_2$ 511.0975, Found 511.0966.

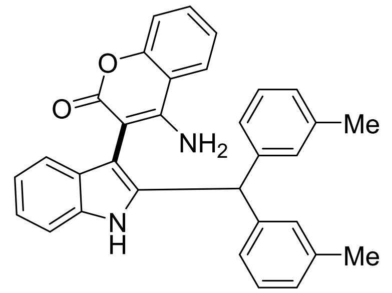

3n

4-Amino-3-(2-(di-m-tolylmethyl)-1H-indol-3-yl)-2H-chromen-2-one (3n). White solid (82.8 mg, 88%); m.p. 171.2-171.9°C; ^{1}H NMR (400 MHz, $CDCl_3$) δ 7.87 (s, 1H), 7.58 – 7.45 (m, 2H), 7.29 (d, *J* = 8.0 Hz, 1H), 7.20 (t, *J* = 7.4 Hz, 1H), 7.15 – 6.91 (m, 10H), 6.88 – 6.82 (m, 2H), 5.64 (s, 1H), 4.82 (s, 2H), 2.24 (s, 3H), 2.15 (s, 3H). ^{13}C NMR (100 MHz, $CDCl_3$) δ 162.03, 153.15, 151.25, 141.93, 141.49, 139.88, 138.33, 137.79, 135.43, 131.39, 129.82, 129.25, 128.48, 128.08, 127.71, 127.48, 127.22, 125.93, 125.53, 123.33, 121.66, 119.83, 119.07, 117.13, 114.07, 111.11, 104.13, 92.53, 49.37, 21.33, 21.27; HRMS (ESI-TOF) m/z: $[M+H]^+$ Calcd for $C_{32}H_{27}N_2O_2$ 471.2067, Found 471.2063.

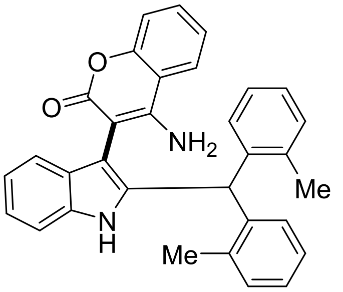

3o

4-Amino-3-(2-(di-o-tolylmethyl)-1H-indol-3-yl)-2H-chromen-2-one (3o). White solid (23.5 mg, 25%); m.p. 323.8-324.5°C; ^{1}H NMR (400 MHz, $CDCl_3$) δ 7.84 (s, 1H), 7.49 (t, *J* = 7.4 Hz, 1H), 7.32 (t, *J* = 6.0 Hz, 2H), 7.25 (s, 1H), 7.22 – 7.14 (m, 4H), 7.11 (q, *J* = 14.8, 7.8 Hz, 2H), 7.05 – 6.92 (m, 5H), 6.88 (d, *J* = 7.2 Hz, 1H), 6.02 (s, 1H), 4.60 (s, 2H), 2.37 (s, 3H), 2.02 (s, 3H). ^{13}C NMR (100 MHz, $CDCl_3$) δ 161.46, 153.25, 150.80, 141.06, 139.35, 138.54, 138.16, 136.39, 135.12, 131.46, 130.89, 130.46, 128.11, 127.97, 127.78, 127.17, 126.55, 126.14, 125.67, 123.25, 121.71, 121.21, 120.09, 119.30, 117.42, 113.91, 111.13, 104.35, 92.68, 43.08, 19.66, 19.28; HRMS (ESI-TOF) m/z: $[M+H]^+$ Calcd for $C_{32}H_{27}N_2O_2$

471.2067, Found 471.2067.

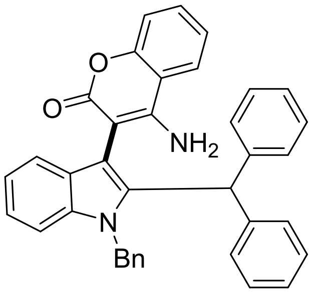

3p

4-Amino-3-(2-benzhydryl-1-benzyl-1H-indol-3-yl)-2H-chromen-2-one (3p). White solid (103.4 mg, 97%); m.p. 223.7-224.5°C; ^{1}H NMR (400 MHz, $CDCl_3$) δ 7.47 (t, *J* = 7.6 Hz, 1H), 7.39 (d, *J* = 7.6 Hz, 2H), 7.29 (d, *J* = 8.0 Hz, 2H), 7.24 (d, *J* = 8.0 Hz, 6H), 7.21 – 7.11 (m, 4H), 7.06 (t, *J* = 7.4 Hz, 1H), 6.96 (d, *J* = 7.6 Hz, 2H), 6.91 (d, *J* = 6.8 Hz, 2H), 6.82 (t, *J* = 7.2 Hz, 2H), 6.55 (t, *J* = 7.2 Hz, 1H), 5.60 (s, 1H), 5.31 (d, *J* = 8.6 Hz, 1H), 5.06 (d, *J* = 8.6 Hz, 1H), 4.57 (s, 1H). ^{13}C NMR (100 MHz, $CDCl_3$) δ 162.17, 153.11, 150.09, 140.33, 140.07, 139.46, 137.69, 137.38, 131.18, 129.53, 129.08, 128.71, 128.51, 127.46, 127.24, 126.82, 126.20, 125.82, 123.00, 122.40, 120.90, 120.08, 118.84, 117.07, 114.05, 109.75, 106.22, 94.55, 49.72, 47.22; HRMS (ESI-TOF) m/z: $[M+H]^+$ Calcd for $C_{37}H_{29}N_2O_2$ 533.2224, Found 533.2222.

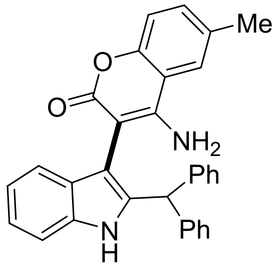

3q

4-Amino-3-(2-benzhydryl-1H-indol-3-yl)-6-methyl-2H-chromen-2-one (3q). White solid (80.8 mg, 88%); m.p. 158.8-160.4°C; ^{1}H NMR (400 MHz, $CDCl_3$) δ 7.89 (s, 1H), 7.30 (d, *J* = 8.4 Hz, 1H), 7.25 – 7.04 (m, 15H), 6.98 (t, *J* = 7.0 Hz, 1H), 5.74 (s, 1H), 4.70 (s, 2H), 2.38 (s, 3H). ^{13}C NMR (100 MHz, $CDCl_3$) δ 162.07, 151.41, 151.12, 142.08, 141.63, 139.75, 135.70, 132.93, 132.57, 129.04, 128.66, 128.58, 128.33, 127.35, 126.93, 126.54, 121.88, 121.24, 119.98, 119.26, 117.14, 113.63, 111.14, 104.45, 92.78, 49.03, 20.96; HRMS (ESI-TOF) m/z: $[M+H]^+$ Calcd for $C_{31}H_{25}N_2O_2$ 457.1911, Found 457.1908.

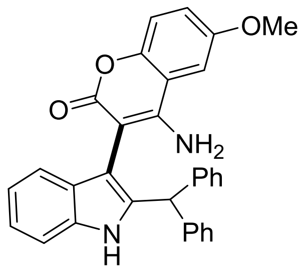

3r

4-Amino-3-(2-benzhydryl-1H-indol-3-yl)-6-methoxy-2H-chromen-2-one (3r). White solid (81.5 mg, 86%); m.p. 205.5-205.9°C; ^{1}H NMR (400 MHz, $CDCl_3$) δ 7.93 (s, 1H), 7.34 (d, *J* = 8.4 Hz, 1H), 7.31 – 7.26 (m, 3H), 7.25 – 7.15 (m, 7H), 7.12 (d, *J* = 6.2 Hz, 5H), 7.01 (t, *J* = 7.0 Hz,, 1H), 5.77 (s, 1H), 4.75 (s, 2H), 2.42 (s, 3H). ^{13}C NMR (100 MHz, $CDCl_3$) δ 162.12, 151.38, 151.21, 142.06, 141.63, 139.72, 135.69, 132.94, 132.55, 129.02, 128.63, 128.57, 128.32, 127.35, 126.92, 126.54, 121.85, 121.33, 119.94, 119.23, 117.09, 113.63, 111.12, 104.45, 92.65, 49.03, 20.94; HRMS (ESI-TOF) m/z: $[M+H]^+$ Calcd for $C_{31}H_{25}N_2O_3$ 473.1860, Found 473.1864.

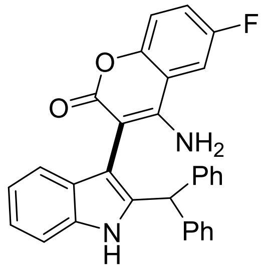

3s

4-Amino-3-(2-benzhydryl-1H-indol-3-yl)-6-fluoro-2H-chromen-2-one (3s). White solid (51.7 mg, 56%); m.p. 301.0-301.7°C; ^{1}H NMR (400 MHz, DMSO-d_6) δ 10.92 (s, 1H), 7.97 (dd, *J* = 10.0, 2.8 Hz, 1H), 7.46 (td, *J* = 8.8, 2.8 Hz, 1H), 7.44 – 7.33 (m, 2H), 7.29 (d, *J* = 7.6 Hz, 2H), 7.24 (t, *J* = 8.0 Hz, 5H), 7.22 – 7.03 (m, 5H), 6.94 (t, *J* = 7.4 Hz, 1H), 5.55 (s, 1H). ^{13}C NMR (100 MHz, DMSO-d_6) δ 160.85, 157.67 (d, *J* = 236.6 Hz), 151.72, 149.24, 142.39, 142.28, 139.18, 136.70, 129.03, 128.96, 128.07, 127.90, 127.28, 126.21, 126.16, 120.79, 118.80, 118.60 (d, *J* = 7.8 Hz), 118.32 (d, *J* = 8.5 Hz), 115.53 (d, *J* = 8.6 Hz), 111.44, 109.21 (d, *J* = 25.5 Hz), 104.63, 90.61, 48.85. ^{19}F NMR (376 MHz, DMSO-d_6) δ -118.87 (1F); HRMS (ESI-TOF) m/z: $[M+H]^+$ Calcd for $C_{30}H_{22}FN_2O_2$ 461.1660, Found 461.1659.

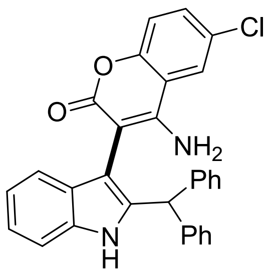

3t

4-Amino-3-(2-benzhydryl-1H-indol-3-yl)-6-chloro-2H-chromen-2-one (3t). White solid (50.5 mg, 53%); m.p. 188.6-189.3°C; ^{1}H NMR (400 MHz, DMSO-d_6) δ 10.91 (s, 1H), 8.20 (d, J = 2.0 Hz, 1H), 7.61 (dd, J = 8.8, 2.0 Hz, 1H), 7.37 (dd, J = 17.6, 8.0 Hz, 2H), 7.27 (t, J = 7.0 Hz, 3H), 7.22 (d, J = 6.0 Hz, 5H), 7.22 – 7.10 (m, 3H), 7.07 (t, J = 7.4 Hz, 1H), 6.94 (t, J = 7.4 Hz, 1H), 5.53 (s, 1H). ^{13}C NMR (100 MHz, DMSO-d_6) δ 160.57, 151.51, 151.40, 142.35, 142.25, 139.22, 136.67, 131.13, 129.00, 128.94, 128.06, 127.89, 127.34, 127.21, 126.21, 126.16, 122.96, 120.79, 118.64, 118.53, 118.42, 115.96, 111.43, 104.50, 90.68, 48.83; HRMS (ESI-TOF) m/z: $[M+H]^+$ Calcd for $C_{30}H_{22}ClN_2O_2$ 477.1365, Found 477.1359.

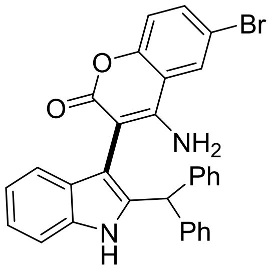

3u

4-Amino-3-(2-benzhydryl-1H-indol-3-yl)-6-bromo-2H-chromen-2-one (3u). Pale yellow solid (75.2 mg, 72%); m.p. 166.7-167.3°C; ^{1}H NMR (400 MHz, DMSO-d_6) δ 10.90 (s, 1H), 8.32 (s, 1H), 7.73 (d, J = 8.8 Hz, 1H), 7.39 (d, J = 8.0 Hz, 1H), 7.30 – 7.20 (m, 9H), 7.18 (t, J = 7.6 Hz, 1H), 7.13 (d, J = 8.0 Hz, 2H), 7.07 (t, J = 7.6 Hz, 1H), 6.93 (t, J = 7.4 Hz, 1H), 5.53 (s, 1H). ^{13}C NMR (100 MHz, DMSO-d_6) δ 160.51, 151.92, 151.31, 142.35, 142.25, 139.23, 136.66, 133.94, 128.99, 128.93, 128.06, 127.89, 127.19, 126.21, 126.16, 125.87, 120.79, 118.72, 118.64, 118.52, 116.42, 115.12, 111.43, 104.47, 90.66, 48.81; HRMS (ESI-TOF) m/z: $[M+H]^+$ Calcd for $C_{30}H_{22}BrN_2O_2$ 521.0859, Found 521.0859.

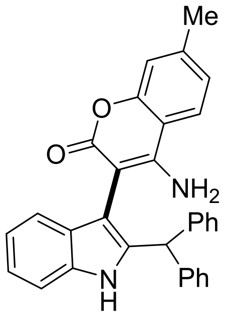

3v

4-Amino-3-(2-benzhydryl-1H-indol-3-yl)-7-methyl-2H-chromen-2-one (3v). White solid (90.1 mg, 98%); m.p. 181.6-182.7°C; ^{1}H NMR (400 MHz, $CDCl_3$) δ 7.90 (s, 1H), 7.39 (d, *J* = 8.0 Hz, 1H), 7.31 – 7.27 (m, 3H), 7.23 (d, *J* = 6.0 Hz, 1H), 7.18 (dd, *J* = 15.2, 8.0 Hz, 5H), 7.15 – 7.08 (m, 5H), 7.04 (t, *J* = 8.4, 2H), 5.77 (s, 1H), 4.77 (s, 2H), 2.48 (s, 3H). ^{13}C NMR (100 MHz, $CDCl_3$) δ 162.19, 153.32, 151.49, 142.57, 142.06, 141.63, 139.69, 135.66, 129.04, 128.63, 128.57, 128.31, 127.44, 126.92, 126.51, 124.56, 121.82, 121.30, 119.92, 119.24, 117.37, 111.53, 111.11, 104.46, 91.76, 49.08, 21.53; HRMS (ESI-TOF) m/z: $[M+H]^+$ Calcd for $C_{31}H_{25}N_2O_2$ 457.1911, Found 457.1902.

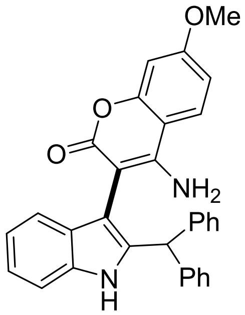

3w

4-Amino-3-(2-benzhydryl-1H-indol-3-yl)-7-methoxy-2H-chromen-2-one (3w). White solid (86.2 mg, 91%); m.p. 250.9-251.2°C; ^{1}H NMR (400 MHz, $CDCl_3$) δ 7.85 (s, 1H), 7.40 (d, *J* = 9.2 Hz, 1H), 7.23 (d, *J* = 9.6 Hz, 3H), 7.19 (d, *J* = 3.6 Hz, 1H), 7.18 – 7.01 (m, 9H), 6.97 (t, *J* = 7.2 Hz, 1H), 6.83 – 6.71 (m, 2H), 5.72 (s, 1H), 4.71 (s, 2H), 3.86 (s, 3H). ^{13}C NMR (100 MHz, $CDCl_3$) δ 162.48, 162.31, 154.98, 151.81, 142.07, 141.65, 139.68, 135.64, 129.03, 128.62, 128.57, 128.31, 127.55, 126.91, 126.50, 122.77, 121.80, 119.88, 119.21, 111.67, 111.09, 107.36, 104.44, 100.79, 90.17, 55.68, 49.07; HRMS (ESI-TOF) m/z: $[M+H]^+$ Calcd for $C_{31}H_{25}N_2O_3$ 473.1860, Found 473.1851.

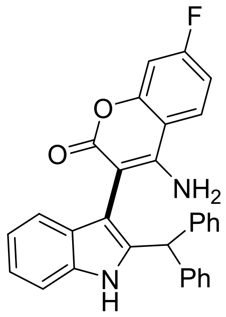

3x

4-Amino-3-(2-benzhydryl-1H-indol-3-yl)-7-fluoro-2H-chromen-2-one (3x). White solid (83.1 mg, 90%); m.p. 235.7-237.7°C; ^{1}H NMR (400 MHz, DMSO-d_6) δ 10.91 (s, 1H), 7.96 (dd, J = 10.0, 2.8 Hz 1H), 7.46 (td, J = 8.8, 2.8 Hz, 1H), 7.42 – 7.34 (m, 2H), 7.29 (d, J = 7.2 Hz, 2H), 7.24 (t, J = 8.0 Hz, 6H), 7.20 – 7.11 (m, 3H), 7.07 (t, J = 7.6 Hz, 1H), 6.94 (t, J = 7.6 Hz, 1H), 5.54 (s, 1H). ^{13}C NMR (100 MHz, DMSO-d_6) δ 160.83, 157.67 (d, J = 237.4 Hz), 151.72, 149.23, 142.38, 142.27, 139.17, 136.69, 129.02, 128.95, 128.06, 127.89, 127.28, 126.21, 126.16, 120.78, 118.80, 118.59 (d, J = 7.3 Hz), 118.31 (d, J = 8.5 Hz), 115.52 (d, J = 8.8 Hz), 111.43, 109.20 (d, J = 25.5 Hz), 104.63, 90.59, 48.84. ^{19}F NMR (376 MHz, DMSO-d_6) δ -118.87 (1F); HRMS (ESI-TOF) m/z: $[M+H]^+$ Calcd for $C_{30}H_{22}FN_2O_2$ 461.1660, Found 461.1650.

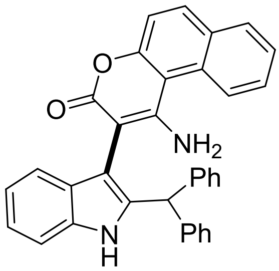

3y

1-Amino-2-(2-benzhydryl-1H-indol-3-yl)-3H-benzo[f]chromen-3-one (3y). Pale yellow solid (42.3 mg, 43%); m.p. 162.7-163.2°C; ^{1}H NMR (400 MHz, $CDCl_3$) δ 8.61 (d, J = 7.6 Hz, 1H), 7.97 (s, 1H), 7.84 (d, J = 7.6 Hz, 1H), 7.67 – 7.55 (m, 3H), 7.49 (d, J = 8.4 Hz, 1H), 7.31 (d, J = 7.6 Hz, 2H), 7.25 – 7.08 (m, 10H). 7.07 (d, J = 6.8 Hz, 1H), 7.03 – 6.95 (m, 1H), 5.83 (s, 1H), 4.96 (s, 2H). ^{13}C NMR (100 MHz, $CDCl_3$) δ 162.05, 152.44, 150.47, 142.09, 141.64, 139.82, 135.74, 134.60, 129.06, 128.63, 128.57, 128.41, 128.31, 127.49, 127.35, 126.94, 126.52, 123.44, 123.38, 122.94, 121.88, 119.99, 119.26, 117.67, 111.18, 108.82,

104.35, 92.04, 49.09; HRMS (ESI-TOF) m/z: $[M+H]^+$ Calcd for $C_{34}H_{25}N_2O_2$ 493.1911, Found 439.1902.

6. General Procedure for the Preparation of 5

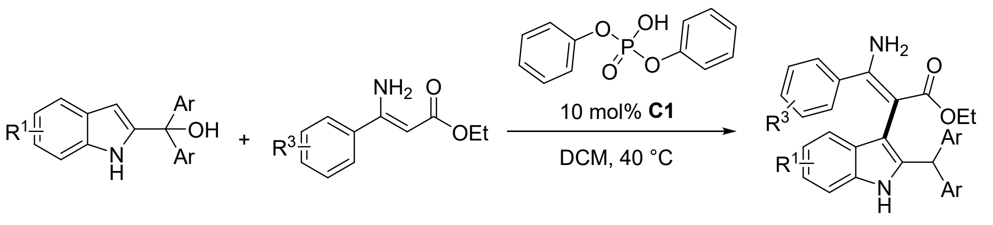

1 **4** **5**

An oven-dried 25 mL Schlenk tube was charged with 2-indolylmethanol **1** (0.2 mmol, 1.0 equiv.), acyclic *β*-enamino esters **4** (0.24 mmol, 1.2 equiv.), and **C1** (0.02 mmol, 0.1 equiv.) in DCM (2 mL). The reaction mixture was stirred at 40 °C until the full consumption of 2-indolylmethanol **1** (typically, about 30 min). After the reaction was then quenched by saturated $NaHCO_3$, the organic layer was separated, and the aqueous phase was extracted with CH_2Cl_2 (3×10 mL). The combined organic layers were washed by saturated NaCl and dried over anhydrous Na_2SO_4. After filtration, the solution was concentrated and the crude product was purified by flash column chromatography on silica gel using gradient elution (petroleum ether/ethyl acetate = 35/1-20/1) to afford the target product **5a-5w**.

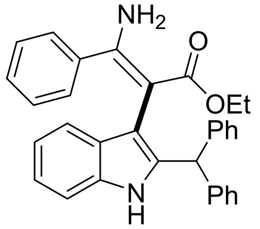

5a

Ethyl (Z)-3-amino-2-(2-benzhydryl-1H-indol-3-yl)-3-phenylacrylate (5a). White solid (88.5 mg, 88%); m.p. 199.6-201.3°C; ^{1}H NMR (400 MHz, $CDCl_3$) δ 7.52 (t, J = 4.0 Hz, 1H), 7.30 (s, 1H), 7.24 – 7.16 (m, 4H), 7.17 – 7.05 (m, 8H), 7.05 – 6.97 (m, 4H), 6.54 (d, J = 7.6 Hz, 2H), 5.32 (s, 1H), 4.19 – 3.48 (m, 2H), 0.97 (t, J = 7.0 Hz, 3H). ^{13}C NMR (100 MHz, $CDCl_3$) δ 171.19, 161.24, 142.24, 141.54, 138.82, 137.15, 135.09, 131.34, 128.85, 128.65, 128.38, 128.07, 127.89, 127.60, 126.38, 126.30, 120.90, 119.70, 119.28, 111.17, 110.39,

88.51, 59.00, 48.17, 14.39; HRMS (ESI-TOF) m/z: $[M+H]^+$ Calcd for $C_{32}H_{29}N_2O_2$ 473.2224, Found 473.2226.

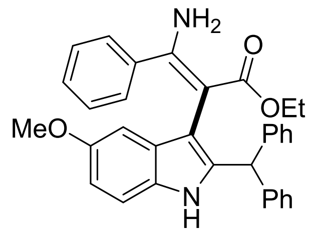

5b

Ethyl (Z)-3-amino-2-(2-benzhydryl-5-methoxy-1H-indol-3-yl)-3-phenylacrylate (5b). White solid (99.3 mg, 98%); m.p. 201.4-203.1°C; ^1H NMR (400 MHz, $CDCl_3$) δ 7.24 – 7.08 (m, 10H), 7.06 – 6.98 (m, 5H), 6.97 (s, 1H), 6.74 (d, J = 9.6 Hz, 1H), 6.54 (d, J = 6.8 Hz, 2H), 5.28 (s, 1H), 4.09 – 3.88 (m, 2H), 3.86 (s, 3H), 1.00 (t, J = 7.0 Hz, 3H). ^{13}C NMR (100 MHz, $CDCl_3$) δ 171.20, 161.24, 154.00, 142.23, 141.53, 138.79, 138.05, 131.84, 130.17, 128.83, 128.72, 128.36, 128.04, 127.89, 127.57, 126.37, 126.28, 111.09, 110.88, 101.46, 88.52, 58.99, 55.90, 48.28, 14.45; HRMS (ESI-TOF) m/z: $[M+H]^+$ Calcd for $C_{33}H_{31}N_2O_3$ 503.2329, Found 503.2325.

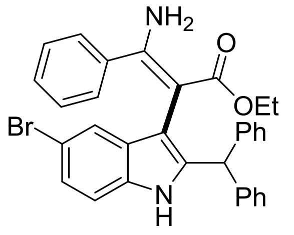

5c

Ethyl (Z)-3-amino-2-(2-benzhydryl-5-bromo-1H-indol-3-yl)-3-phenylacrylate (5c). White solid (91.3 mg, 83%); m.p. 208.8-208.9°C; ^1H NMR (400 MHz, $CDCl_3$) δ 7.64 (s, 1H), 7.34 (s, 1H), 7.24 – 7.10 (m, 10H), 7.04 (t, J = 7.2 Hz, 2H), 7.00 (d, J = 8.4 Hz, 3H), 6.51 (d, J = 7.2 Hz, 2H), 5.27 (s, 1H), 4.17 – 3.50 (m, 2H), 0.98 (t, J = 7.6 Hz, 3H). ^{13}C NMR (100 MHz, $CDCl_3$) δ 170.81, 161.45, 141.84, 141.15, 138.61, 138.58, 133.60, 133.07, 128.84, 128.80, 128.74, 128.47, 128.15, 127.99, 127.59, 126.56, 126.45, 123.73, 122.15, 112.71, 111.96, 110.96, 87.77, 59.11, 48.15, 14.37; HRMS (ESI-TOF) m/z: $[M+H]^+$ Calcd for $C_{32}H_{28}BrN_2O_2$ 551.1329, Found 551.1323.

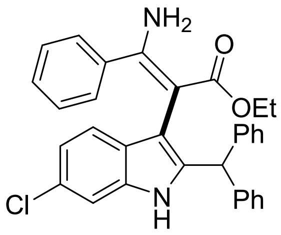

5d

Ethyl (Z)-3-amino-2-(2-benzhydryl-6-chloro-1H-indol-3-yl)-3-phenylacrylate (5d). White solid (98.2 mg, 96%); m.p. 225.7-226.2°C; ^{1}H NMR (400 MHz, $CDCl_3$) δ 7.41 (d, *J* = 8.0 Hz, 1H), 7.31 (s, 1H), 7.23 – 7.08 (m, 10H), 7.07 – 6.95 (m, 5H), 6.52 (d, *J* = 7.2 Hz, 2H), 5.29 (s, 1H), 4.17 – 3.66 (m, 2H), 0.97 (t, *J* = 7.0 Hz, 3H). ^{13}C NMR (100 MHz, $CDCl_3$) δ 170.93, 161.45, 141.90, 141.17, 138.60, 137.97, 135.33, 129.90, 128.79, 128.76, 128.46, 128.14, 127.97, 127.53, 126.69, 126.53, 126.44, 120.46, 120.01, 111.35, 110.52, 87.87, 59.07, 48.15, 14.38; HRMS (ESI-TOF) m/z: $[M+H]^+$ Calcd for $C_{32}H_{28}ClN_2O_2$ 507.1834, Found 507.1836.

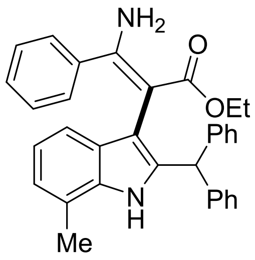

5e

Ethyl (Z)-3-amino-2-(2-benzhydryl-7-methyl-1H-indol-3-yl)-3-phenylacrylate (5e). White solid (68.8 mg, 70%); m.p. 203.0-203.3°C; ^{1}H NMR (400 MHz, $CDCl_3$) δ 7.39 (d, *J* = 7.6 Hz, 1H), 7.25 – 7.09 (m, 10H), 7.03 (dd, *J* = 17.6, 8.4 Hz, 5H), 6.90 (d, *J* = 7.2 Hz, 1H), 6.57 (d, *J* = 7.2 Hz, 2H), 5.35 (s, 1H), 4.09 – 3.84 (m, 2H), 2.30 (s, 3H), 0.99 (t, *J* = 7.0 Hz, 3H). ^{13}C NMR (100 MHz, $CDCl_3$) δ 171.22, 161.25, 142.35, 141.57, 138.85, 136.74, 134.71, 130.90, 128.84, 128.82, 128.59, 128.33, 128.08, 127.92, 127.61, 126.34, 126.28, 121.77, 119.43, 119.22, 117.53, 111.79, 88.76, 58.99, 48.21, 16.48, 14.44; HRMS (ESI-TOF) m/z: $[M+H]^+$ Calcd for $C_{33}H_{31}N_2O_2$ 487.2380, Found 487.2375.

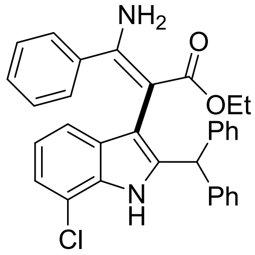

5f

Ethyl (Z)-3-amino-2-(2-benzhydryl-7-chloro-1H-indol-3-yl)-3-phenylacrylate (5f). White solid (83.2 mg, 82%); m.p. 214.4-216.9°C; ^{1}H NMR (400 MHz, $CDCl_3$) δ 7.47 (s, 1H), 7.43 (d, J = 7.6 Hz, 1H), 7.25 – 7.20 (m, 3H), 7.17 (t, J = 7.8 Hz, 4H), 7.10 (t, J = 8.4 Hz, 3H), 7.07 – 6.99 (m, 5H), 6.57 (d, J = 6.8 Hz, 2H), 5.35 (s, 1H), 4.37 – 3.40 (m, 2H), 0.98 (t, J = 7.2 Hz, 3H). ^{13}C NMR (100 MHz, $CDCl_3$) δ 170.90, 161.57, 141.88, 140.99, 138.54, 138.09, 132.76, 132.29, 128.77, 128.47, 128.19, 128.02, 127.51, 126.56, 126.46, 120.53, 120.12, 118.31, 115.76, 112.40, 88.07, 59.07, 48.20, 14.37. ; HRMS (ESI-TOF) m/z: $[M+H]^+$ Calcd for $C_{32}H_{28}ClN_2O_2$ 507.1834, Found 507.1838.

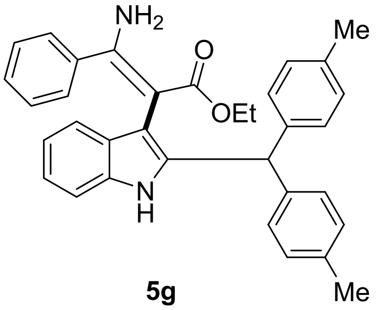

5g

Ethyl (Z)-3-amino-2-(2-(di-p-tolylmethyl)-1H-indol-3-yl)-3-phenylacrylate (5g). White solid (93.9 mg, 94%); m.p. 188.6-189.3°C; ^{1}H NMR (400 MHz, $CDCl_3$) δ 7.55 – 7.48 (m, 1H), 7.30 (s, 1H), 7.20 – 7.10 (m, 4H), 7.07 (dd, J = 5.6, 3.2 Hz, 2H), 7.04 – 6.97 (m, 4H), 6.92 (t, J = 8.6 Hz, 4H), 6.43 (d, J = 8.0 Hz, 2H), 5.24 (s, 1H), 4.07 – 3.85 (m, 2H), 2.30 (d, J = 3.2 Hz, 6H), 0.98 (t, J = 7.6 Hz, 3H). ^{13}C NMR (100 MHz, $CDCl_3$) δ 171.24, 161.17, 139.39, 138.91, 138.73, 137.62, 135.82, 135.65, 135.04, 131.40, 129.03, 128.70, 128.58, 127.83, 127.63, 120.75, 119.62, 119.18, 110.87, 110.35, 88.64, 58.98, 47.40, 20.94, 14.35; HRMS (ESI-TOF) m/z: $[M+H]^+$ Calcd for $C_{34}H_{33}N_2O_2$ 501.2537, Found 501.2534.

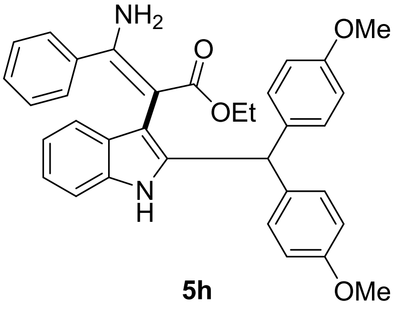

5h

Ethyl(Z)-3-amino-2-(2-(bis(4-methoxyphenyl)methyl)-1H-indol-3-yl)-3-phenylacrylate (5h). White solid (80.9 mg, 76%); m.p. 193.5-194.3°C; ^{1}H NMR (400 MHz, $CDCl_3$) δ 7.57 – 7.48 (m, 1H), 7.30 (s, 1H), 7.21 – 7.11 (m, 4H), 7.08 (dd, *J* = 6.0, 3.6 Hz, 2H), 7.01 (t, *J* = 7.6 Hz, 2H), 6.93 (d, *J* = 8.4 Hz, 2H), 6.74 (d, *J* = 8.4 Hz, 2H), 6.66 (d, *J* = 8.4 Hz, 2H), 6.44 (d, *J* = 8.4 Hz, 2H), 5.21 (s, 1H), 4.07 – 3.88 (m, 2H), 3.76 (d, *J* = 5.2 Hz, 6H), 0.99 (t, *J* = 7.0 Hz, 3H). ^{13}C NMR (100 MHz, $CDCl_3$) δ 171.21, 161.17, 158.04, 157.98, 138.91, 137.84, 135.02, 134.74, 133.93, 131.42, 129.75, 129.72, 128.60, 127.84, 127.61, 120.77, 119.62, 119.21, 113.72, 113.43, 110.77, 110.36, 88.59, 58.98, 55.20, 46.51, 14.42; HRMS (ESI-TOF) m/z: $[M+H]^+$ Calcd for $C_{34}H_{33}N_2O_4$ 533.2435, Found 533.2436.

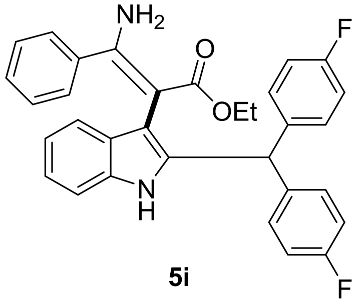

5i

Ethyl (Z)-3-amino-2-(2-(bis(4-fluorophenyl)methyl)-1H-indol-3-yl)-3-phenylacrylate (5i). White solid (85.4 mg, 84%); m.p. 212.6-213.0°C; ^{1}H NMR (400 MHz, $CDCl_3$) δ 7.54 (dd, *J* = 5.6, 3.2 Hz, 1H), 7.23 – 7.08 (m, 7H), 7.02 (t, *J* = 7.8 Hz, 2H), 6.98 – 6.93 (m, 2H), 6.89 (t, *J* = 8.6 Hz, 2H), 6.80 (t, *J* = 8.6 Hz, 2H), 6.42 (dd, *J* = 8.4, 5.6 Hz, 2H), 5.26 (s, 1H), 4.18 – 3.78 (m, 2H), 0.98 (t, *J* = 7.0 Hz, 3H). ^{13}C NMR (100 MHz, $CDCl_3$) δ 171.06, 161.47 (d, *J* = 244.2 Hz), 161.26, 138.74, 137.85, 137.82, 137.02, 136.99, 136.52, 135.18, 131.37, 130.23, 130.21, 130.15, 130.13, 128.76, 127.95, 127.56, 121.24, 119.81, 119.56, 115.10 (dd, *J* = 27.2, 21.0 Hz), 111.45, 110.48, 88.23, 59.05, 46.58, 14.43. ^{19}F NMR (376 MHz, $CDCl_3$)

δ -116.24 (t, J = 13.9 Hz, 1F), -116.45 (t, J = 13.1 Hz, 1F); HRMS (ESI-TOF) m/z: $[M+H]^+$ Calcd for $C_{32}H_{27}F_2N_2O_2$ 509.2035, Found 509.2035.

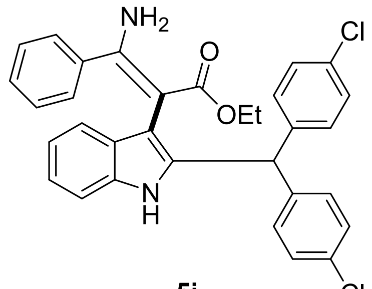

5j

Ethyl (Z)-3-amino-2-(2-(bis(4-chlorophenyl)methyl)-1H-indol-3-yl)-3-phenylacrylate (5j). White solid (94.5 mg, 87%); m.p. 203.9-205.6°C; ^{1}H NMR (400 MHz, $CDCl_3$) δ 7.55 (dd, J = 5.2, 3.2 Hz, 1H), 7.23 (s, 1H), 7.18 (d, J = 8.0 Hz, 3H), 7.15 – 7.05 (m, 7H), 7.01 (t, J = 7.6 Hz, 2H), 6.92 (d, J = 8.4 Hz, 2H), 6.39 (d, J = 8.0 Hz, 2H), 5.26 (s, 1H), 4.22 – 3.50 (m, 2H), 0.98 (t, J = 6.8 Hz, 3H). ^{13}C NMR (100 MHz, $CDCl_3$) δ 171.01, 161.32, 140.34, 139.49, 138.67, 135.87, 135.27, 132.46, 132.40, 131.32, 130.08, 130.01, 128.77, 128.59, 128.34, 127.96, 127.52, 121.38, 119.83, 119.63, 111.76, 110.53, 88.10, 59.08, 46.85, 14.40; HRMS (ESI-TOF) m/z: $[M+H]^+$ Calcd for $C_{32}H_{27}Cl_2N_2O_2$ 541.1444, Found 541.1434.

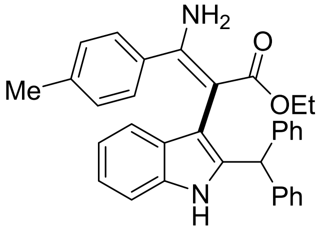

5k

Ethyl (Z)-3-amino-2-(2-benzhydryl-1H-indol-3-yl)-3-(p-tolyl)acrylate (5k). White solid (87.6 mg, 90%); m.p. 245.6-246.1°C; ^{1}H NMR (400 MHz, $CDCl_3$) δ 7.53 (s, 1H), 7.30 (s, 1H), 7.24 – 7.06 (m, 9H), 7.01 (d, J = 6.8 Hz, 4H), 6.81 (d, J = 7.2 Hz, 2H), 6.52 (d, J = 6.8 Hz, 2H), 5.31 (s, 1H), 4.06 – 3.78 (m, 2H), 2.24 (s, 3H), 0.96 (t, J = 6.6 Hz, 3H). ^{13}C NMR (100 MHz, $CDCl_3$) δ 171.25, 161.36, 142.31, 141.60, 138.65, 136.94, 135.93, 135.15, 131.42, 128.89, 128.84, 128.53, 128.23, 128.05, 127.54, 126.27, 120.89, 119.76, 119.26, 111.38, 110.39, 88.24, 58.94, 48.03, 21.14, 14.41; HRMS (ESI-TOF) m/z: $[M+H]^+$ Calcd for $C_{33}H_{31}N_2O_2$ 487.2380, Found 487.2375.

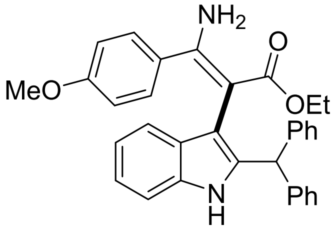

5l

Ethyl (Z)-3-amino-2-(2-benzhydryl-1H-indol-3-yl)-3-(4-methoxyphenyl)acrylate (5l). White solid (88.4 mg, 88%); m.p. 239.5-239.9°C; ^{1}H NMR (400 MHz, $CDCl_3$) δ 7.56 – 7.48 (m, 1H), 7.35 (s, 1H), 7.24 – 7.06 (m, 9H), 7.03 (t, J = 9.4 Hz, 4H), 6.57 (d, J = 7.2 Hz, 2H), 6.49 (d, J = 8.4 Hz, 2H), 5.33 (s, 1H), 4.05 – 3.89 (m, 2H), 3.70 (s, 3H), 0.98 (t, J = 7.0 Hz, 3H). ^{13}C NMR (100 MHz, $CDCl_3$) δ 171.27, 161.14, 159.87, 142.39, 141.54, 136.92, 135.21, 131.37, 131.08, 129.01, 128.86, 128.80, 128.26, 128.07, 126.30, 126.27, 120.91, 119.74, 119.27, 113.18, 111.46, 110.40, 88.06, 58.93, 55.11, 48.07, 14.43; HRMS (ESI-TOF) m/z: $[M+H]^+$ Calcd for $C_{33}H_{31}N_2O_3$ 503.2329, Found 503.2324.

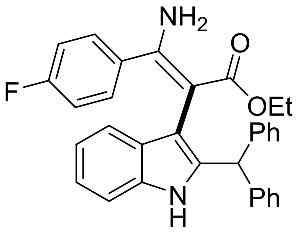

5m

Ethyl (Z)-3-amino-2-(2-benzhydryl-1H-indol-3-yl)-3-(4-fluorophenyl)acrylate (5m). White solid (88.3 mg, 86%); m.p. 139.9-142.5°C; ^{1}H NMR (400 MHz, DMSO-d_6) δ 10.36 (s, 1H), 7.24 (d, J = 6.8 Hz, 3H), 7.19 (s, 5H), 7.06 (d, J = 7.2 Hz, 2H), 6.99 – 6.87 (m, 4H), 6.78 (s, 2H), 6.64 (t, J = 8.4 Hz, 2H), 5.39 (s, 1H), 3.86 (d, J = 4.8 Hz, 2H), 0.85 (t, J = 6.8 Hz, 3H). ^{13}C NMR (100 MHz, DMSO-d_6) δ 170.36, 161.72 (d, J = 244.0 Hz), 161.69, 142.95, 141.38, 137.68, 135.69, 134.34, 129.86, 129.78, 129.70, 128.73, 128.67, 127.95, 126.03, 120.03, 118.49, 118.32, 114.05, 113.84, 110.99, 110.04, 85.75, 58.10, 47.87, 14.32. ^{19}F NMR (376 MHz, DMSO-d_6) δ -113.28(1F); HRMS (ESI-TOF) m/z: $[M+H]^+$ Calcd for $C_{32}H_{28}FN_2O_2$ 491.2130, Found 491.2132.

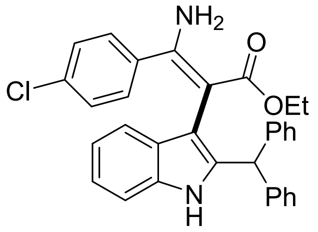

5n

Ethyl (Z)-3-amino-2-(2-benzhydryl-1H-indol-3-yl)-3-(4-chlorophenyl)acrylate (5n). Yellow solid (92.6 mg, 91%); m.p. 245.2-247.0°C; ^{1}H NMR (400 MHz, DMSO-d_6) δ 10.39 (s, 1H), 7.29 – 7.12 (m, 8H), 7.05 (d, J = 7.2 Hz, 2H), 6.97 – 6.83 (m, 6H), 6.77 – 6.70 (m, 2H), 5.37 (s, 1H), 3.94 – 3.76 (m,2H), 0.85 (t, J = 7.0 Hz, 3H). ^{13}C NMR (100 MHz, DMSO) δ 170.31, 161.45, 142.91, 141.32, 137.75, 136.76, 135.69, 132.86, 129.87, 129.39, 128.71, 128.69, 127.97, 127.96, 127.15, 126.07, 126.03, 120.10, 118.49, 118.39, 111.03, 109.89, 85.89, 58.16, 47.83, 14.34; HRMS (ESI-TOF) m/z: [M+H]$^+$ Calcd for $C_{32}H_{28}ClN_2O_2$ 507.1834, Found 507.1840.

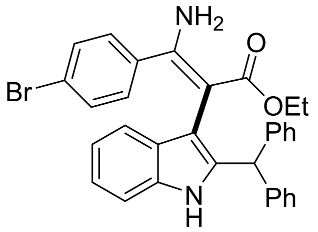

5o

Ethyl (Z)-3-amino-2-(2-benzhydryl-1H-indol-3-yl)-3-(4-bromophenyl)acrylate (5o). White solid (93.7 mg, 85%); m.p. 252.1-252.2°C; ^{1}H NMR (400 MHz, CDCl$_3$) δ 7.49 (d, J = 4.8 Hz, 1H), 7.39 (s, 1H), 7.24 – 7.06 (m, 9H), 7.02 (dd, J = 13.6, 8.0 Hz, 4H), 6.91 (d, J = 8.0 Hz, 2H), 6.59 (d, J = 6.0 Hz, 2H), 5.35 (s, 1H), 4.28 – 3.87 (m, 2H), 1.00 (t, J = 7.0 Hz, 3H). ^{13}C NMR (100 MHz, CDCl$_3$) δ 171.11, 160.13, 142.43, 141.03, 137.43, 137.20, 135.25, 131.15, 130.98, 129.14, 128.75, 128.69, 128.46, 128.19, 126.40, 122.89, 121.18, 119.53, 119.48, 110.86, 110.55, 88.95, 59.20, 48.01, 14.41; HRMS (ESI-TOF) m/z: [M+H]$^+$ Calcd for $C_{32}H_{28}BrN_2O_2$ 551.1329, Found 551.1326.

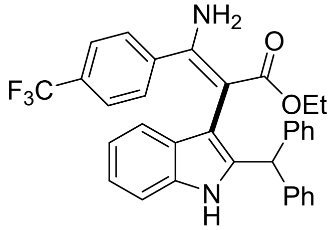

5p

Ethyl(Z)-3-amino-2-(2-benzhydryl-1H-indol-3-yl)-3-(4-(trifluoromethyl)phenyl) acrylate (5p). White solid (93.1 mg, 86%); m.p. 239.0-239.2°C; ^{1}H NMR (400 MHz, $CDCl_3$) δ 7.52 (s, 1H), 7.38 (s, 1H), 7.23 – 7.07 (m, 13H), 6.99 (d, J = 7.2 Hz, 2H), 6.52 (d, J = 7.6 Hz, 2H), 5.34 (s, 1H), 4.09 – 3.93 (m, 2H), 1.01 (t, J = 7.0 Hz, 3H). ^{13}C NMR (100 MHz, $CDCl_3$) δ 171.04, 159.64, 142.33, 142.04, 140.91, 137.37, 135.23, 131.15, 130.43 (d, J = 32.4 Hz), 128.73, 128.54, 128.44, 128.21, 127.90, 126.47 (d, J = 4.5 Hz), 124.77 (q, J = 3.7 Hz), 121.26, 119.59, 119.47, 110.61, 110.58, 89.46, 59.29, 47.98, 14.39. ^{19}F NMR (376 MHz, $CDCl_3$) δ -62.73 (3F); HRMS (ESI-TOF) m/z: $[M+H]^+$ Calcd for $C_{33}H_{28}F_3N_2O_2$ 541.2098, Found 541.2094.

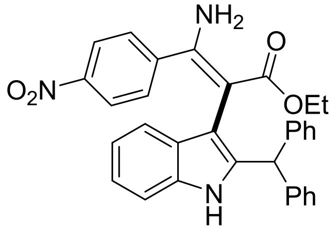

5q

Ethyl (Z)-3-amino-2-(2-benzhydryl-1H-indol-3-yl)-3-(4-nitrophenyl)acrylate (5q). Yellow solid (83.6 mg, 80%); m.p. 257.6-257.9°C; ^{1}H NMR (400 MHz, DMSO-d_6) δ 10.50 (s, 1H), 7.54 (d, J = 8.8 Hz, 2H), 7.33 – 7.21 (m, 3H), 7.20 – 7.10 (m, 5H), 7.07 (d, J = 8.8 Hz, 2H), 7.02 (d, J = 7.6 Hz, 2H), 6.99 – 6.87 (m, 2H), 6.81 (d, J = 6.8 Hz, 2H), 5.40 (s, 1H), 4.03 – 3.84 (m, 2H), 0.92 (t, J = 7.0 Hz, 3H). ^{13}C NMR (100 MHz, DMSO-d_6) δ 170.15, 160.36, 146.67, 144.30, 143.17, 140.89, 137.87, 135.74, 129.68, 128.95, 128.73, 128.59, 128.01, 127.91, 126.09, 122.07, 120.33, 118.57, 118.52, 111.08, 109.38, 86.76, 58.41, 47.71, 14.39; HRMS (ESI-TOF) m/z: $[M+H]^+$ Calcd for $C_{32}H_{28}N_3O_4$ 518.2075, Found 518.2073.

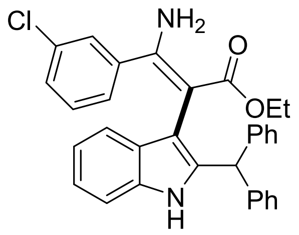

5r

Ethyl (Z)-3-amino-2-(2-benzhydryl-1H-indol-3-yl)-3-(3-chlorophenyl)acrylate (5r). White solid (83.1 mg, 82%); m.p. 201.9-202.0°C; ^{1}H NMR (400 MHz, $CDCl_3$) δ 7.51 – 7.44 (m, 1H), 7.39 (s, 1H), 7.20 (d, J = 7.6 Hz, 2H), 7.19 – 7.13 (m, 6H), 7.12 – 7.05 (m, 3H), 7.01 (d, J = 7.2 Hz, 2H), 6.88 (dt, J = 8.0, 1.2 Hz, 1H), 6.82 (t, J = 7.8 Hz, 1H), 6.67 (dd, J = 6.8, 1.2 Hz, 2H), 5.36 (s, 1H), 4.07 – 3.86 (m, 2H), 0.98 (t, J = 7.0 Hz, 3H). ^{13}C NMR (100 MHz, $CDCl_3$) δ 171.07, 159.73, 142.39, 141.23, 140.35, 137.42, 135.16, 133.76, 131.07, 129.01, 128.80, 128.70, 128.50, 128.16, 127.37, 126.62, 126.40, 126.10, 121.13, 119.51, 119.47, 110.67, 110.50, 89.19, 59.22, 48.25, 14.38; HRMS (ESI-TOF) m/z: $[M+H]^+$ Calcd for $C_{32}H_{28}ClN_2O_2$ 507.1834, Found 507.1826.

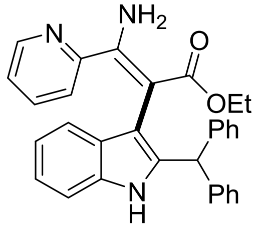

5s

Ethyl (Z)-3-amino-2-(2-benzhydryl-1H-indol-3-yl)-3-(pyridin-2-yl)acrylate (5s). Yellow solid (89.4 mg, 94%); m.p. 210.3-211.0°C; ^{1}H NMR (400 MHz, $CDCl_3$) δ 8.45 (s, 1H), 7.52 (d, J = 8.0 Hz, 2H), 7.22 – 7.15 (m, 4H), 7.15 – 7.02 (m, 7H), 7.00 (d, J = 7.2 Hz, 2H), 6.79 (d, J = 3.6 Hz, 1H), 6.63 (d, J = 6.8 Hz, 2H), 5.39 (s, 1H), 4.12 – 3.81 (m, 2H), 0.99 (t, J = 7.0 Hz, 3H). ^{13}C NMR (100 MHz, $CDCl_3$) δ 171.20, 157.99, 154.64, 148.61, 142.24, 141.28, 137.48, 135.37, 135.33, 130.73, 128.76, 128.46, 128.06, 126.37, 126.34, 124.77, 123.48, 121.30, 119.78, 119.59, 111.13, 110.56, 88.36, 59.14, 48.04, 14.38; HRMS (ESI-TOF) m/z: $[M+H]^+$ Calcd for $C_{31}H_{28}N_3O_2$ 474.2176, Found 474.2175.

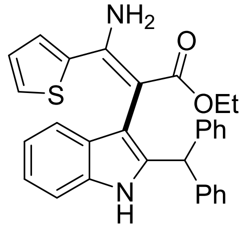

5t

Ethyl (Z)-3-amino-2-(2-benzhydryl-1H-indol-3-yl)-3-(thiophen-2-yl)acrylate (5t). Yellow solid (86.7 mg, 90%); m.p. 212.0-212.3°C; ^{1}H NMR (400 MHz, $CDCl_3$) δ 7.60 (s, 1H), 7.49 (d, *J* = 7.2 Hz, 1H), 7.25 – 7.04 (m, 12H), 6.94 (d, *J* = 3.2 Hz, 1H), 6.77 (t, *J* = 4.0 Hz, 1H), 6.72 (d, *J* = 3.2 Hz, 2H), 5.41 (s, 1H), 4.07 – 3.79 (m, 2H), 0.96 (t, *J* = 7.0 Hz, 3H). ^{13}C NMR (100 MHz, $CDCl_3$) δ 171.01, 153.45, 142.09, 141.76, 139.46, 137.38, 135.25, 131.18, 128.83, 128.44, 128.20, 128.01, 126.44, 126.31, 126.26, 121.16, 119.62, 119.47, 110.69, 110.54, 89.13, 59.17, 48.36, 14.30; HRMS (ESI-TOF) m/z: $[M+H]^+$ Calcd for $C_{30}H_{27}N_2O_2S$ 479.1788, Found 479.1787.

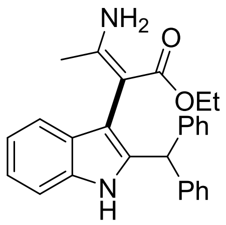

5u

Ethyl (Z)-3-amino-2-(2-benzhydryl-1H-indol-3-yl)but-2-enoate (5u). Yellow solid (73.6 mg, 89%); m.p. 228.1-228.4°C; ^{1}H NMR (400 MHz, DMSO-d_6) δ 10.69 (s, 1H), 7.38 – 7.26 (m, 5H), 7.26 – 7.18 (m, 4H), 7.14 (d, *J* = 8.0 Hz, 3H), 6.99 (t, *J* = 7.4 Hz, 1H), 6.90 (t, *J* = 7.8 Hz, 1H), 5.48 (s, 1H), 3.89 – 3.69 (m, 2H), 1.37 (s, 3H), 0.82 (t, *J* = 7.2 Hz, 3H). ^{13}C NMR (100 MHz, DMSO-d_6) δ 169.79, 162.05, 142.73, 142.39, 138.14, 136.07, 128.81, 128.75, 128.33, 128.00, 126.29, 126.12, 120.22, 118.38, 118.34, 111.11, 110.18, 84.35, 57.75, 48.12, 20.32, 14.35; HRMS (ESI-TOF) m/z: $[M+H]^+$ Calcd for $C_{27}H_{27}N_2O_2$ 411.2067, Found 411.2060.

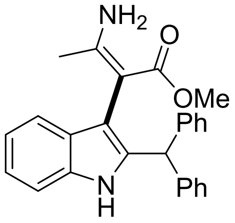

5v

Methyl (Z)-3-amino-2-(2-benzhydryl-1H-indol-3-yl)but-2-enoate (5v). White solid (68.3 mg, 83%); m.p. 239.1-239.6°C; ^{1}H NMR (400 MHz, $CDCl_3$) δ 7.83 (s, 1H), 7.43 (d, *J* = 7.6 Hz, 1H), 7.37 – 7.24 (m, 7H), 7.21 (d, *J* = 7.6 Hz, 4H), 7.18 – 7.09 (m, 2H), 5.70 (s, 1H), 3.43 (s, 3H), 1.60 (s, 3H). ^{13}C NMR (100 MHz, $CDCl_3$) δ 171.08, 160.95, 142.18, 142.12, 138.19, 135.45, 129.56, 128.92, 128.89, 128.47, 128.33, 126.55, 126.50, 121.31, 119.47, 119.20, 110.77, 110.68, 86.74, 50.25, 48.41, 21.28; HRMS (ESI-TOF) m/z: $[M+H]^+$ Calcd for $C_{26}H_{25}N_2O_2$ 397.1911, Found 397.1912.

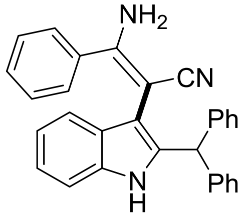

5w

(Z)-3-amino-2-(2-benzhydryl-1H-indol-3-yl)-3-phenylacrylonitrile (5w).Yellow solid (78.9 mg, 92%); m.p. 197.1-197.2°C; ^{1}H NMR (400 MHz, DMSO-d_6) δ 11.00 (s, 1H), 7.67 – 7.56 (m, 2H), 7.54 – 7.46 (m, 3H), 7.41 (d, *J* = 7.6 Hz, 2H), 7.37 – 7.28 (m, 8H), 7.29 – 7.21 (m, 2H), 7.11 (t, *J* = 7.6 Hz, 1H), 7.05 (t, *J* = 7.2 Hz, 1H), 6.13 (s, 1H), 5.77 (s, 1H). ^{13}C NMR (100 MHz, DMSO-d_6) δ 160.25, 142.25, 141.96, 139.09, 136.45, 136.10, 129.78, 128.92, 128.85, 128.50, 128.36, 128.34, 128.20, 126.58, 126.48, 122.98, 121.16, 119.02, 118.49, 111.68, 104.79, 68.28, 48.56; HRMS (ESI-TOF) m/z: $[M+H]^+$ Calcd for $C_{30}H_{24}N_3$ 426.1965, Found 426.1964.

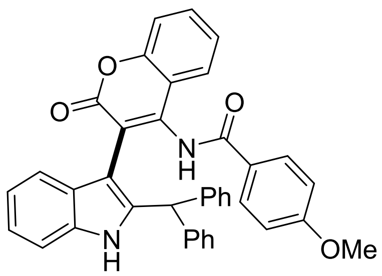

7

N-(3-(2-benzhydryl-1H-indol-3-yl)-2-oxo-2H-chromen-4-yl)-4-methoxybenzamide (7). Yellow solid (95.7 mg, 83%); m.p. 245.6-246.0°C;^{1}H NMR (400 MHz, DMSO-d_6) δ 10.95 (s, 1H), 9.76 (s, 1H), 7.67 – 7.53 (m, 3H), 7.44 (d, J = 8.0 Hz, 2H), 7.37 – 7.27 (m, 2H), 7.27 – 7.18 (m, 5H), 7.18 – 6.98 (m, 7H), 6.97 – 6.86 (m, 3H), 5.64 (s, 1H), 3.80 (s, 3H). ^{13}C NMR (100 MHz, DMSO-d_6) δ 166.38, 162.26, 160.27, 152.14, 146.90, 142.47, 141.72, 138.99, 136.17, 131.39, 130.16, 129.02, 128.91, 128.15, 127.85, 127.51, 126.33, 126.20, 125.79, 125.30, 123.88, 120.84, 119.08, 118.84, 117.56, 117.36, 116.19, 113.35, 111.36, 104.91, 55.47, 48.61; HRMS (ESI-TOF) m/z: [M+H]$^+$ Calcd for $C_{38}H_{29}N_2O_4$ 577.2122, Found 577.2124.

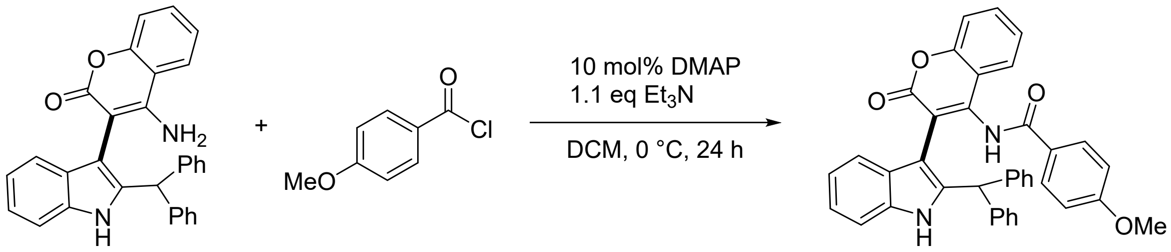

3a **6** **7**

Under an argon atmosphere, compound **3a** (0.2 mmol, 1.0 equiv) and DMAP (0.02 mmol, 10 mol%) were added to a 10 mL reaction tube. Anhydrous DCM (3 mL) was introduced, and the mixture was cooled to 0 °C. Et_3N (0.22 mmol, 1.1 equiv.) was added, followed by dropwise addition of **6** over 10 minutes. The reaction was maintained at 0 °C for 2 h and then warmed to room temperature for 24 h. Reaction progress was monitored by TLC. Upon completion, the mixture was quenched with saturated aqueous $NaHCO_3$. The aqueous layer was extracted with dichloromethane (3 × 5 mL). The combined organic extracts were dried over anhydrous Na_2SO_4, filtered, and concentrated in vacuo to afford the

crude product. Purification by column chromatography on silica gel (gradient elution: hexanes/dichloromethane = 10/1-3/1) yielded pure product **7** (95.7 mg, 83%).

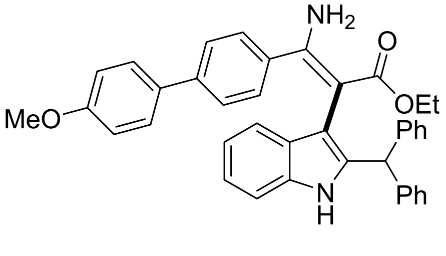

9

Ethyl (Z)-3-amino-2-(2-benzhydryl-1H-indol-3-yl)-3-(4'-methoxy-[1,1'-biphenyl]-4- yl) acrylate (9). Yellow solid (98.4 mg, 85%); mp 278.6-278.9°C; ^{1}H NMR (400 MHz, DMSO-*d*$_6$) δ 10.26 (s, 1H), 7.47 (d, *J* = 8.8 Hz, 2H), 7.31 (dd, *J* = 6.4, 2.0 Hz, 1H), 7.24 (t, *J* = 7.4 Hz,2H), 7.21 – 7.13 (m, 4H), 7.11 – 7.00 (m, 7H), 6.97 (d, *J* = 8.8 Hz, 2H), 6.95 – 6.89 (m, 2H), 6.59 (d, *J* = 7.6 Hz, 2H), 5.36 (s, 1H), 3.89 – 3.78 (m, 2H), 3.77 (s, 3H), 0.81 (t, *J* = 7.0 Hz, 3H). ^{13}C NMR (100 MHz, DMSO) δ 170.48, 162.30, 158.98, 142.76, 141.64, 139.62, 137.67, 136.49, 135.76, 131.57, 130.33, 128.76, 128.70, 128.29, 127.98, 127.79, 127.57, 126.05, 125.87, 124.85, 120.03, 118.63, 118.40, 114.32, 111.07, 110.32, 85.64, 58.00, 55.14, 47.80, 14.34; HRMS (ESI-TOF) m/z: $[M+H]^+$ Calcd for $C_{39}H_{35}N_2O_3$ 579.2642, Found 579.2636.

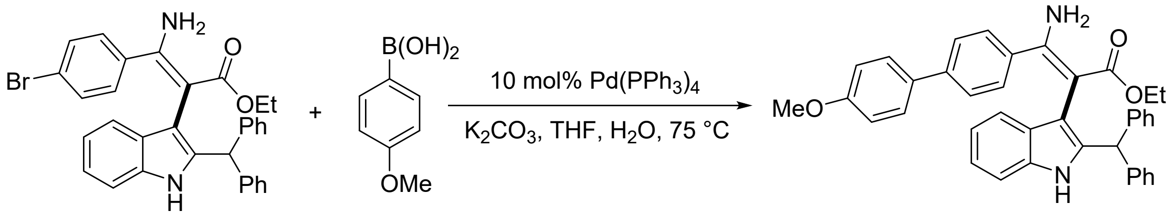

5o **8** **9**

Under a nitrogen atmosphere, substrate **5o** (0.2 mmol, 1.0 equiv), **8** (0.4 mmol, 2.0 equiv), K_2CO_3 (0.6 mmol, 3.0 equiv), and $Pd(PPh_3)_4$ (0.02 mmol, 10 mol%) were charged into a 10 mL reaction flask. Anhydrous THF (4 mL) and deionized H_2O (1 mL) were added, resulting in a 4:1 (v/v) THF/H_2O solvent mixture. The reaction mixture was stirred at 75 °C for 10 h, with progress monitored by TLC. Upon completion, the reaction was quenched with H_2O (10 mL) and extracted with ethyl acetate (3 × 10 mL). The combined organic layers were dried over anhydrous Na_2SO_4, filtered, and concentrated in vacuo. The crude product

was purified by silica gel column chromatography (eluent: hexanes/dichloromethane = 5/1-2/1) to afford product **9** (98.4 mg, 85%).

7. Procedure for the Gram-Scale Synthesis of 3a and 5a

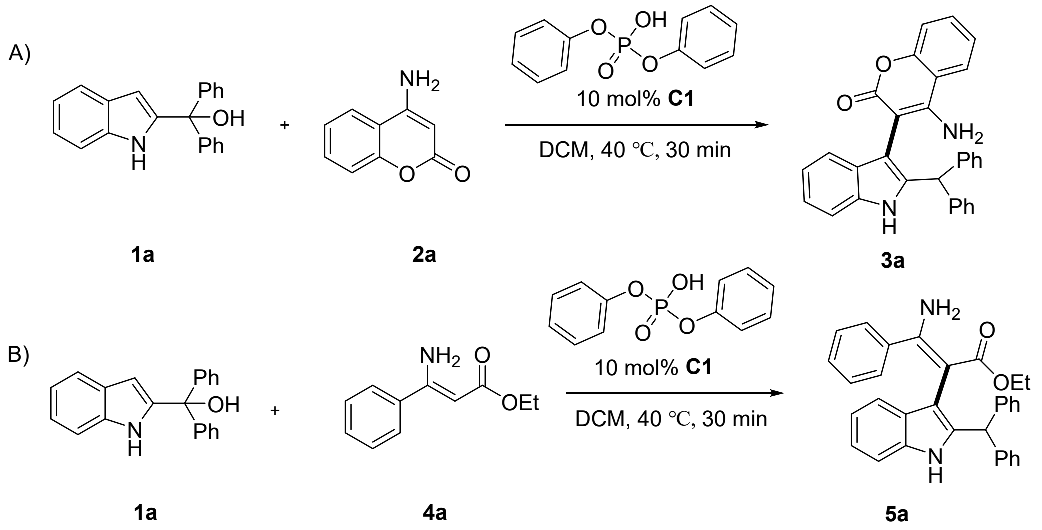

An oven-dried 50 mL Schlenk tube was charged with 2-indolylmethanol **1a** (5 mmol, 1.49 g), 4-aminocoumarin **2a** (6 mmol, 0.97 g) or acyclic *β*-enamino esters **4a** (6 mmol, 1.15 g), and **C1** (0.5 mmol, 0.125 g) in DCM (15 mL). The reaction mixture was stirred at 40 °C until the full consumption of 2-indolylmethanol **1** (typically, about 30 min). After the reaction was then quenched by saturated $NaHCO_3$, the organic layer was separated, and the aqueous phase was extracted with CH_2Cl_2 (3×10 mL). The combined organic layers were washed by saturated NaCl and dried over anhydrous Na_2SO_4. After filtration, the solution was concentrated and the crude product was purified by flash column chromatography on silica gel to afford the target product **3a** (1.97 g, 89% yield) and **5a** (2.03 g, 86% yield).

8. References

[1] T.-Z. Li, S.-J. Liu, Y.-W. Sun, S. Deng, W. Tan, Y. Jiao, Y.-C. Zhang, F. Shi, *Angew. Chem. Int. Ed.* 2021, **60**, 2355.

[2] Peng, S., Wang, L., Huang, J., Sun, S., Guo, H. and Wang, J. *Adv. Synth. Catal.*, 2013, **355**, 2550-2557.

[3] S. Roy, S. K. Mandal, A. Mandal, S. Das, *Tetrahedron Lett.*, 2023, **133**, 154832.

9. Copies of NMR Spectra

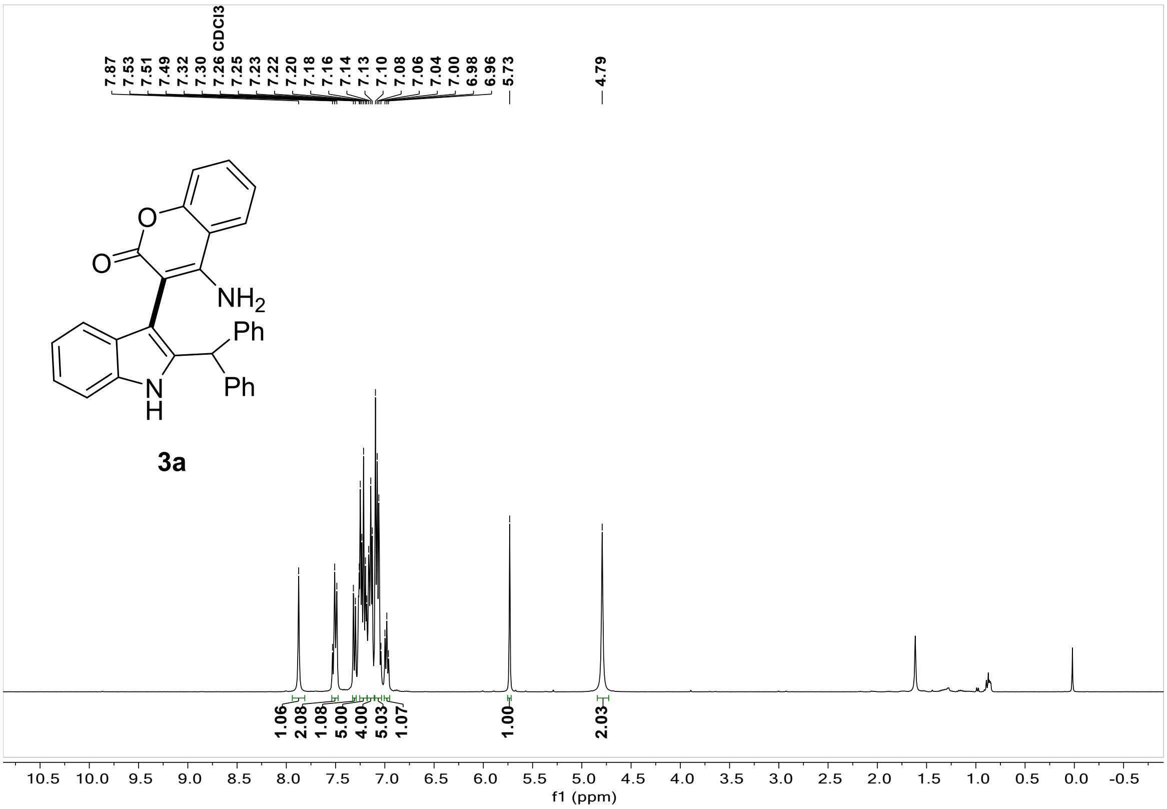

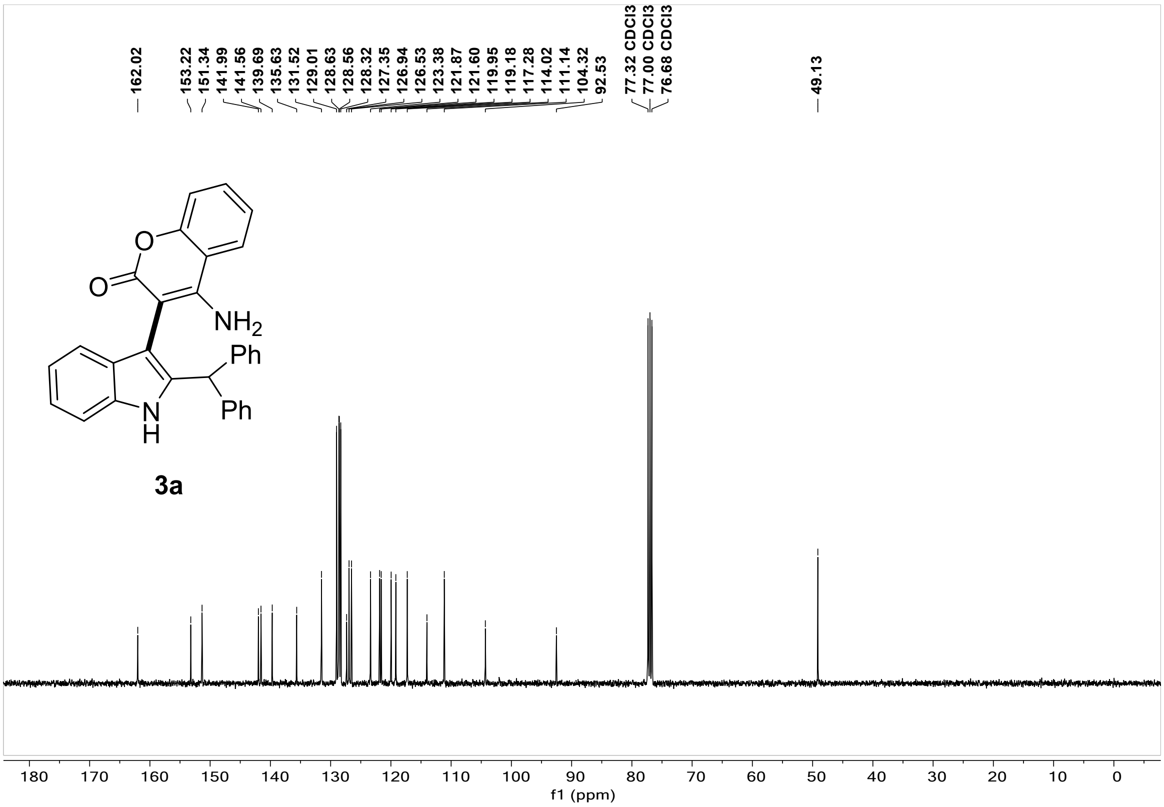

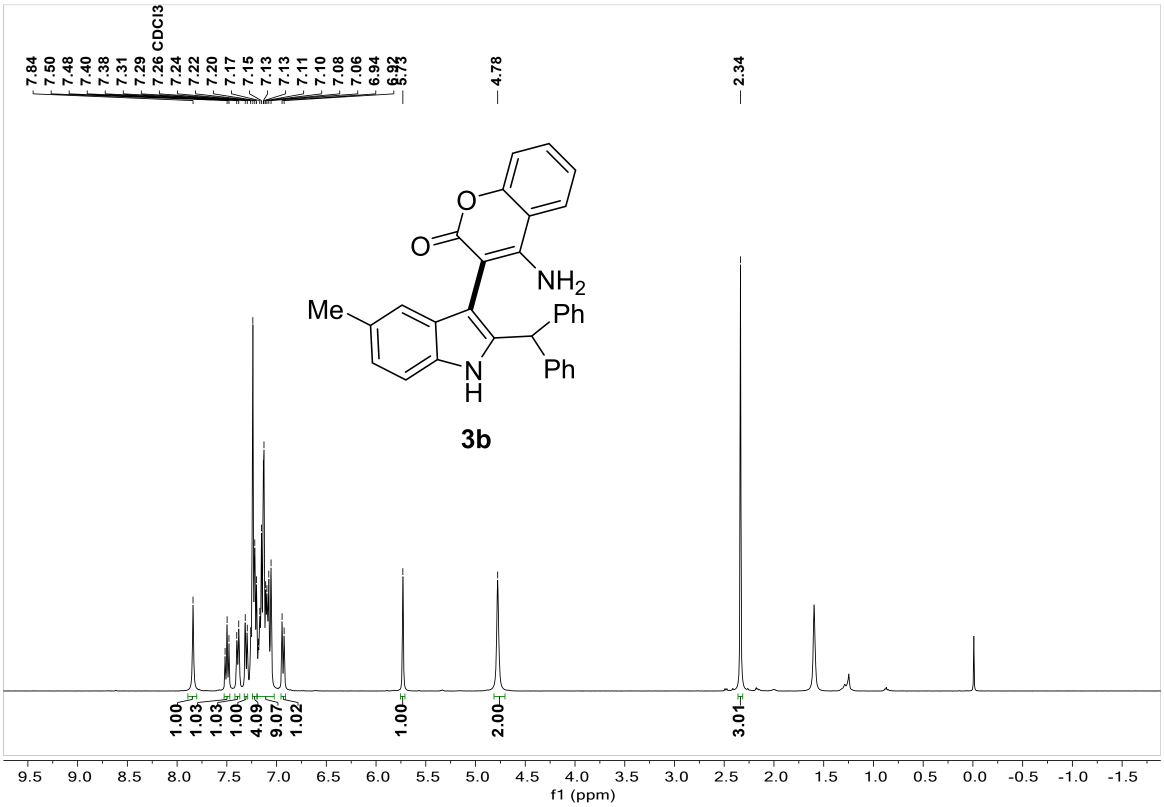
7.84
7.50
7.48
7.40
7.38
7.31
7.29
7.26 CDCl3
7.24
7.22
7.20
7.17
7.15
7.13
7.13
7.11
7.10
7.08
7.06
6.94
6.92
5.73
4.78
2.34
Me
NH2
Ph
Ph
N
H
O
O
3b
1.00
1.03
1.03
1.00
4.09
9.07
1.02
1.00
2.00
3.01
f1 (ppm)

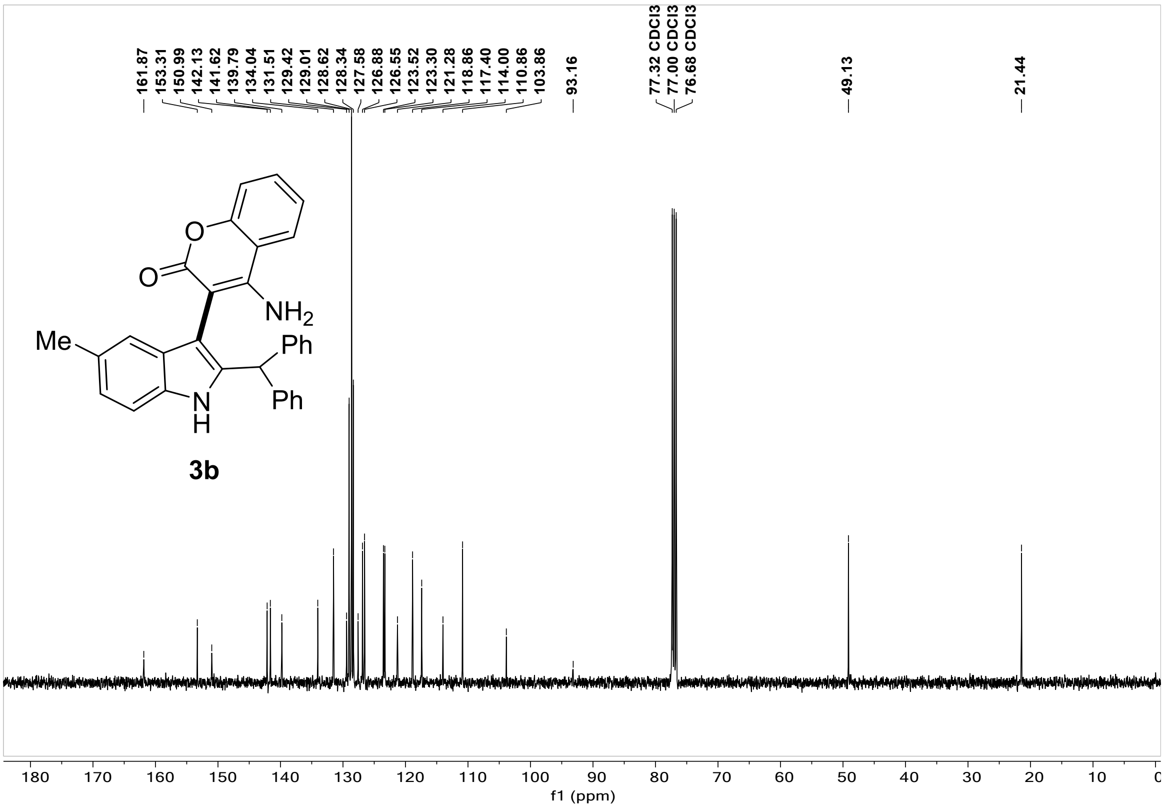
161.87
153.31
150.99
142.13
141.62
139.79
134.04
131.51
129.42
129.01
128.62
128.34
127.58
126.88
126.55
123.52
123.30
121.28
118.86
117.40
114.00
110.86
103.86
93.16
77.32 CDCl3
77.00 CDCl3
76.68 CDCl3
49.13
21.44
Me
NH2
Ph
Ph
N
H
O
O
3b
f1 (ppm)

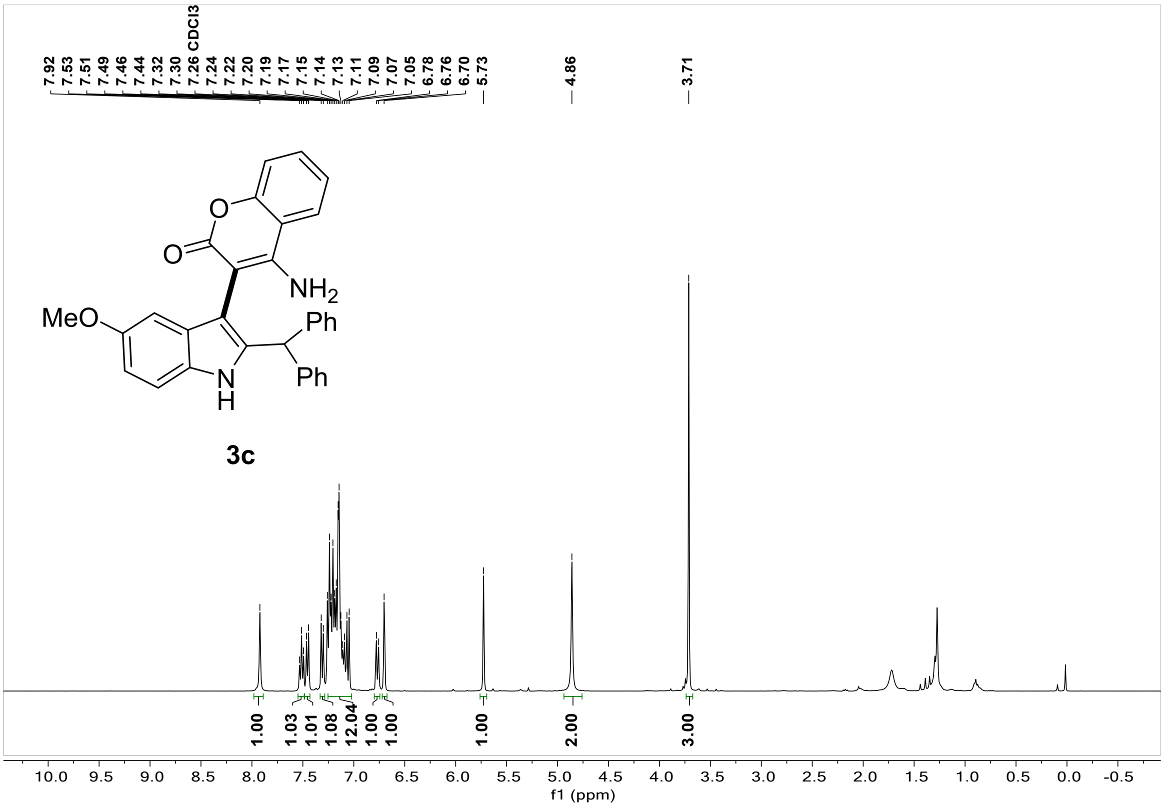
7.92
7.53
7.51
7.49
7.46
7.44
7.32
7.30
7.26 CDCl3
7.24
7.22
7.20
7.19
7.17
7.15
7.14
7.13
7.11
7.09
7.07
7.05
6.78
6.76
6.70
5.73
4.86
3.71
MeO
O
O
NH2
Ph
Ph
N
H
3c
1.00
1.03
1.01
1.08
12.04
1.00
1.00
1.00
2.00
3.00
f1 (ppm)

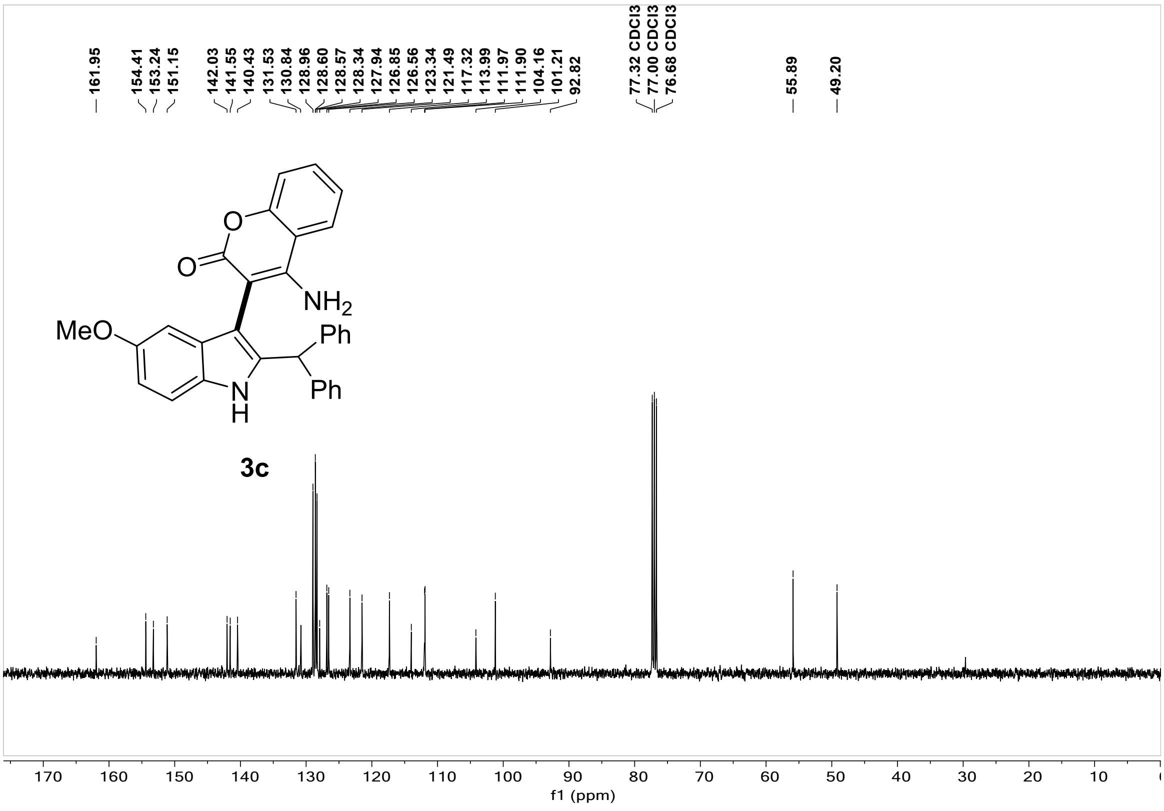
161.95
154.41
153.24
151.15
142.03
141.55
140.43
131.53
130.84
128.96
128.60
128.57
128.34
127.94
126.85
126.56
123.34
121.49
117.32
113.99
111.97
111.90
104.16
101.21
92.82
77.32 CDCl3
77.00 CDCl3
76.68 CDCl3
55.89
49.20
MeO
O
O
NH2
Ph
Ph
N
H
3c
f1 (ppm)

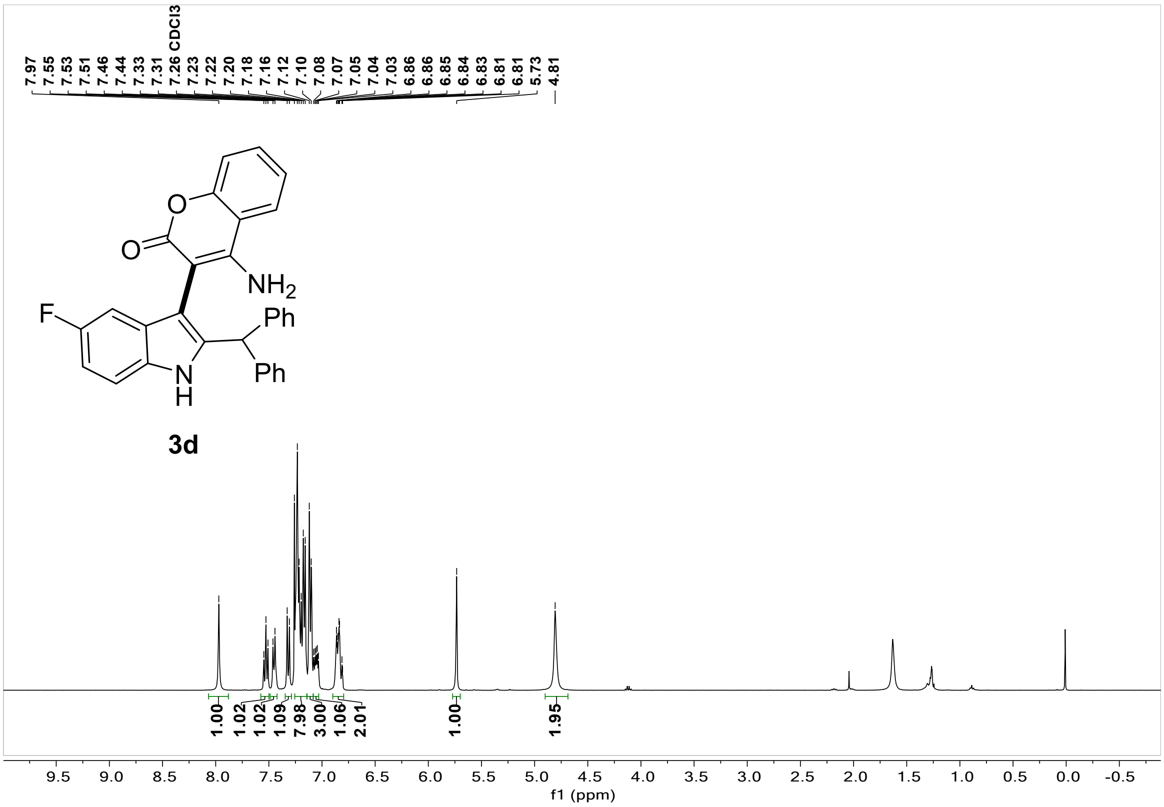
7.97
7.55
7.53
7.51
7.46
7.44
7.33
7.31
7.26 CDCl3
7.23
7.22
7.20
7.18
7.16
7.12
7.10
7.08
7.07
7.05
7.04
7.03
6.86
6.86
6.85
6.84
6.83
6.81
6.81
5.73
4.81
O
O
NH2
F
Ph
Ph
N
H
3d
1.00
1.02
1.02
1.09
7.98
3.00
1.06
2.01
1.00
1.95
f1 (ppm)

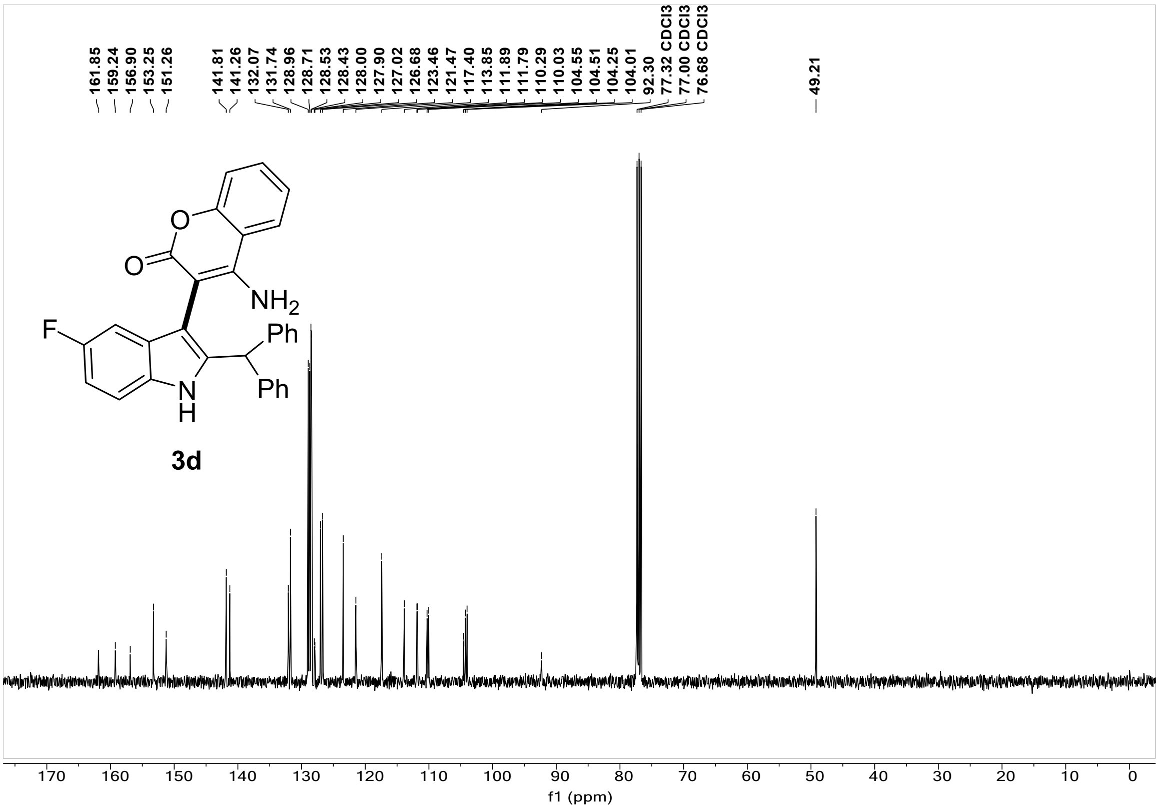
161.85
159.24
156.90
153.25
151.26
141.81
141.26
132.07
131.74
128.96
128.71
128.53
128.43
128.00
127.90
127.02
126.68
123.46
121.47
117.40
113.85
111.89
111.79
110.29
110.03
104.55
104.51
104.25
104.01
92.30
77.32 CDCl3
77.00 CDCl3
76.68 CDCl3
49.21
O
O
NH2
F
Ph
Ph
N
H
3d
f1 (ppm)

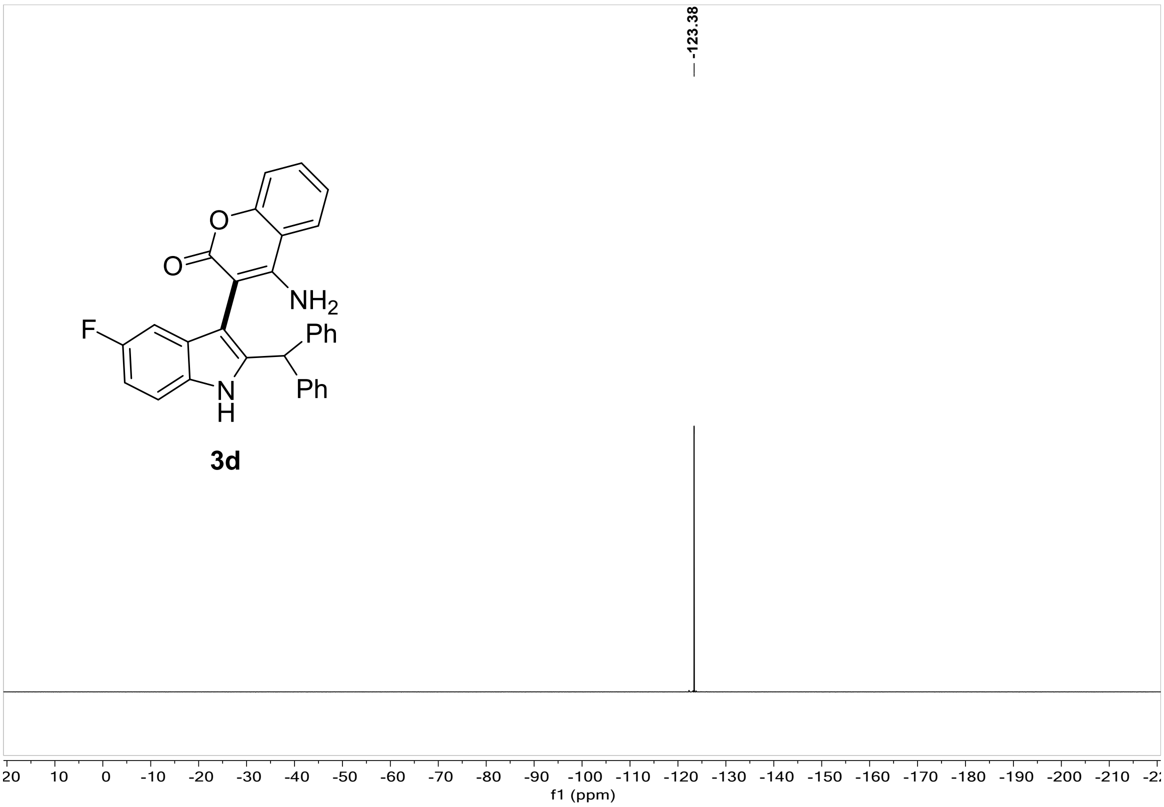
-123.38
F
O
O
NH2
Ph
Ph
N
H
3d
20 10 0 -10 -20 -30 -40 -50 -60 -70 -80 -90 -100 -110 -120 -130 -140 -150 -160 -170 -180 -190 -200 -210
f1 (ppm)

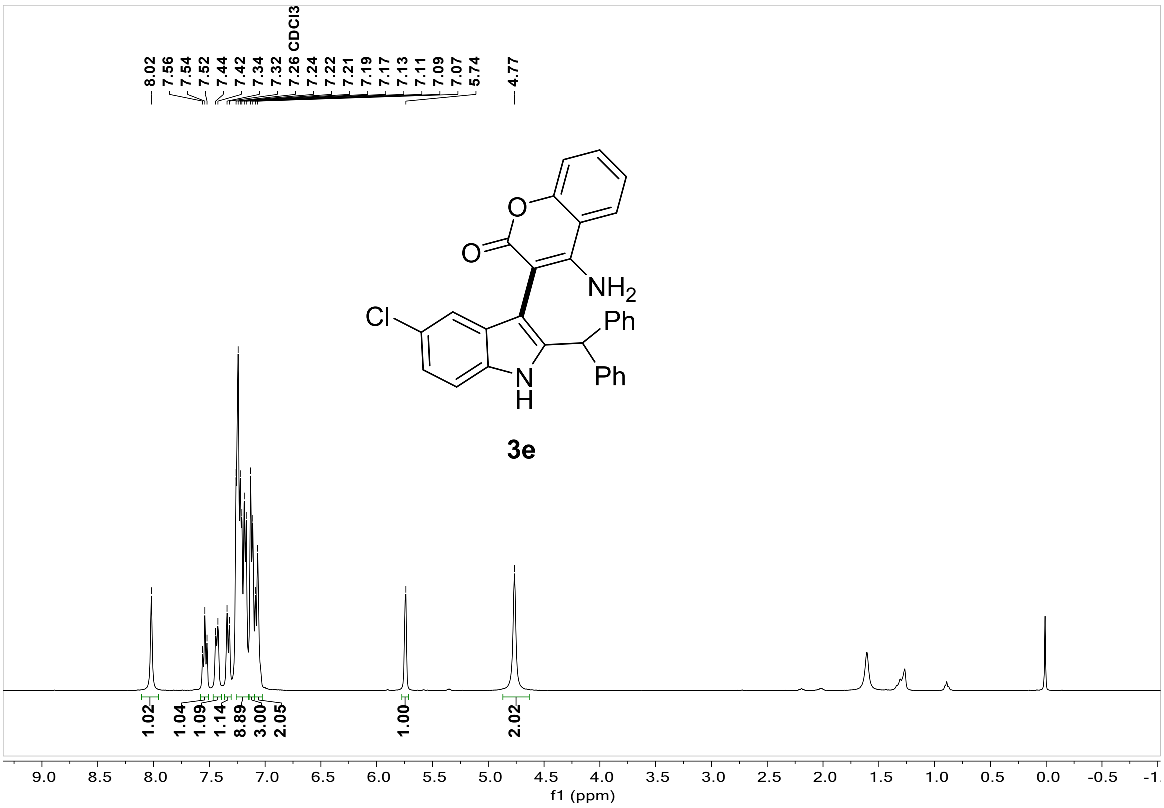
8.02
7.56
7.54
7.52
7.44
7.42
7.34
7.32
7.26 CDCl3
7.24
7.22
7.21
7.19
7.17
7.13
7.11
7.09
7.07
5.74
4.77
Cl
O
O
NH2
Ph
Ph
N
H
3e
1.02
1.04
1.09
1.14
8.89
3.00
2.05
1.00
2.02
9.0 8.5 8.0 7.5 7.0 6.5 6.0 5.5 5.0 4.5 4.0 3.5 3.0 2.5 2.0 1.5 1.0 0.5 0.0 -0.5
f1 (ppm)

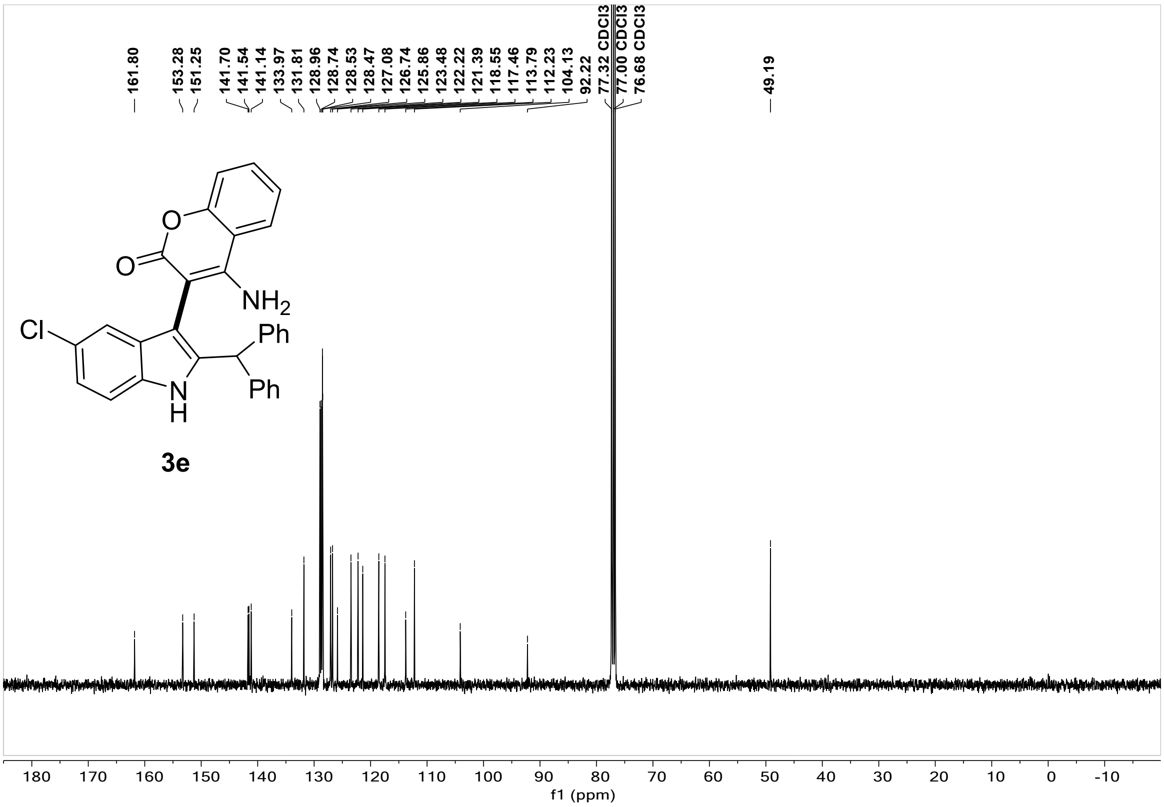
161.80
153.28
151.25
141.70
141.54
141.14
133.97
131.81
128.96
128.74
128.53
128.47
127.08
126.74
125.86
123.48
122.22
121.39
118.55
117.46
113.79
112.23
104.13
92.22
77.32 CDCl3
77.00 CDCl3
76.68 CDCl3
49.19
Cl
O
O
NH2
Ph
Ph
N
H
3e
180 170 160 150 140 130 120 110 100 90 80 70 60 50 40 30 20 10 0 -10
f1 (ppm)

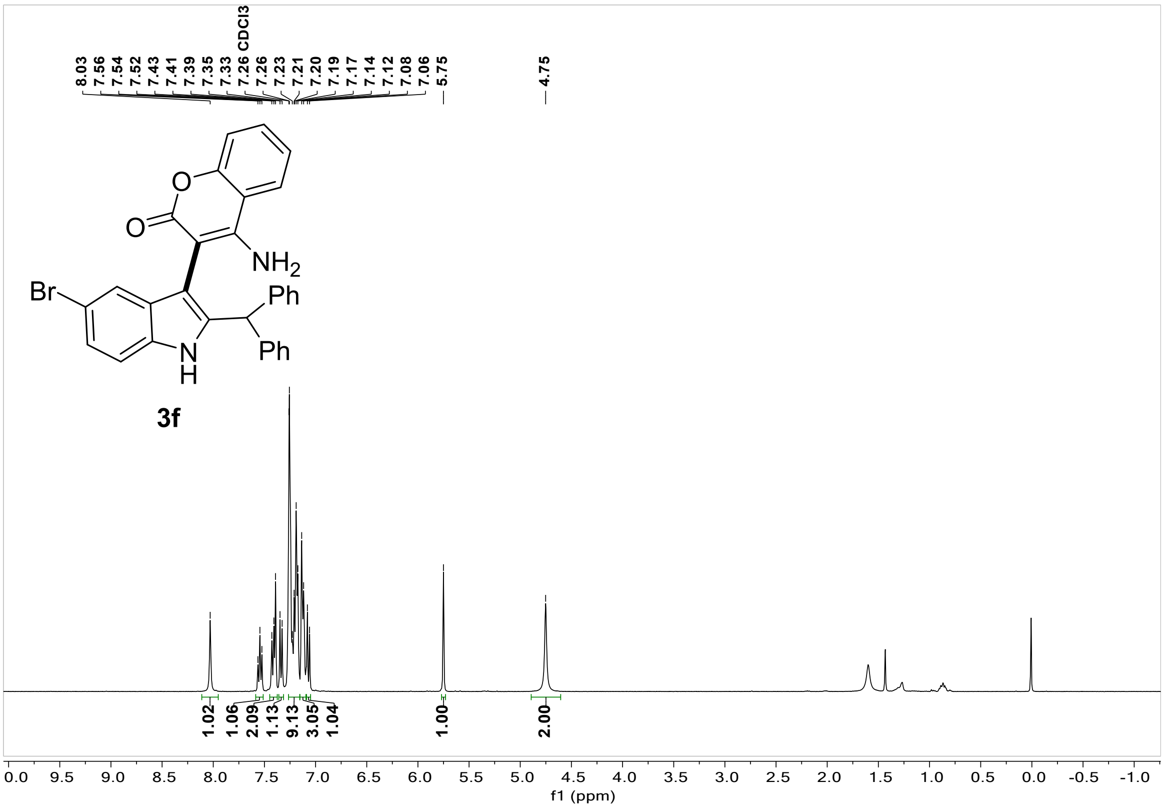
8.03
7.56
7.54
7.52
7.43
7.41
7.39
7.35
7.33
7.26 CDCl3
7.26
7.23
7.21
7.20
7.19
7.17
7.14
7.12
7.08
7.06
5.75
4.75
Br
O
O
NH2
Ph
Ph
N
H
3f
1.02
1.06
2.09
1.13
9.13
3.05
1.04
1.00
2.00
0.0 9.5 9.0 8.5 8.0 7.5 7.0 6.5 6.0 5.5 5.0 4.5 4.0 3.5 3.0 2.5 2.0 1.5 1.0 0.5 0.0 -0.5 -1.0
f1 (ppm)

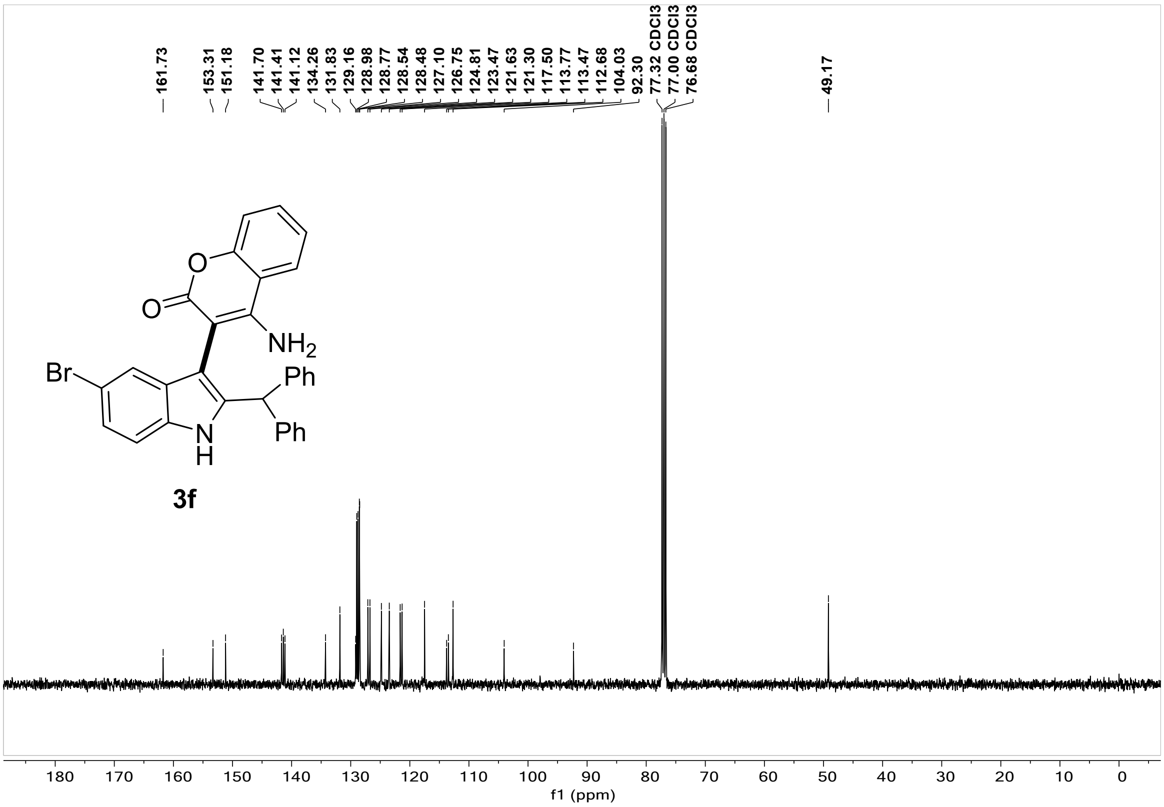
161.73
153.31
151.18
141.70
141.41
141.12
134.26
131.83
129.16
128.98
128.77
128.54
128.48
127.10
126.75
124.81
123.47
121.63
121.30
117.50
113.77
113.47
112.68
104.03
92.30
77.32 CDCl3
77.00 CDCl3
76.68 CDCl3
49.17
Br
O
O
NH2
Ph
Ph
N
H
3f
f1 (ppm)

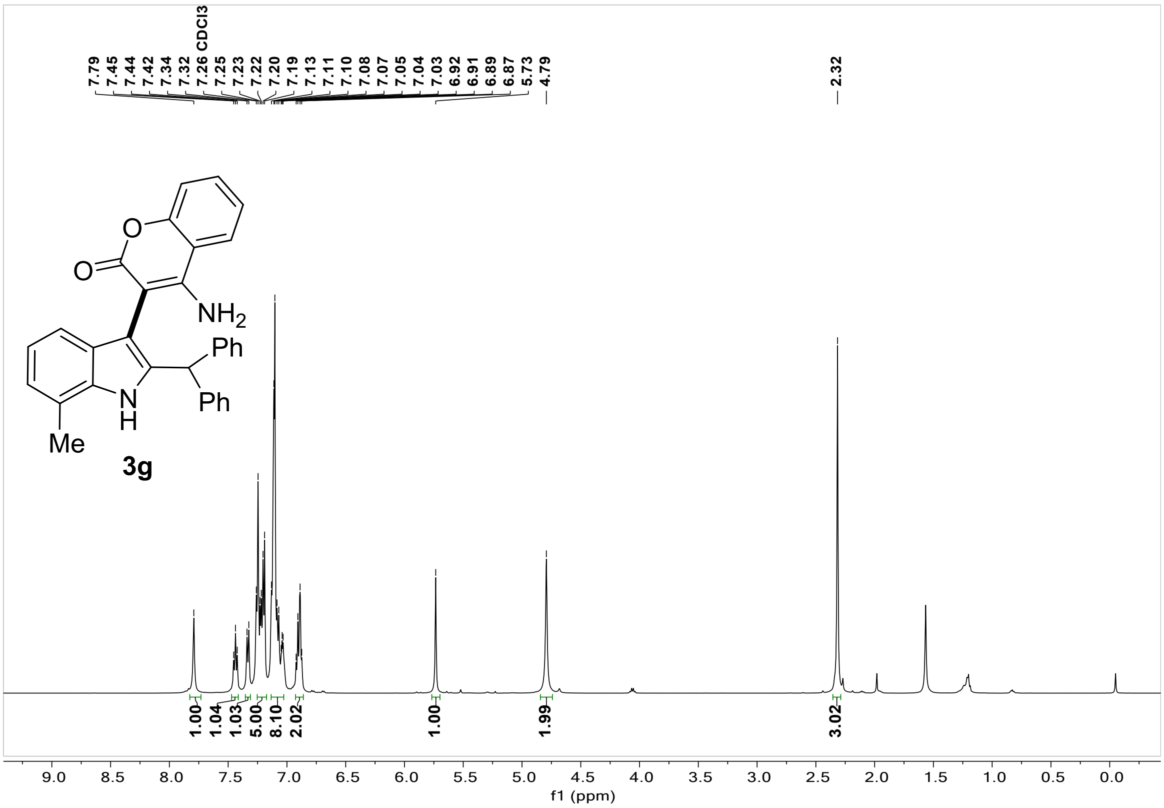
7.79
7.45
7.44
7.42
7.34
7.32
7.26 CDCl3
7.25
7.23
7.22
7.20
7.19
7.13
7.11
7.10
7.08
7.07
7.05
7.04
7.03
6.92
6.91
6.89
6.87
5.73
4.79
2.32
O
O
NH2
Ph
Ph
N
H
Me
3g
1.00
1.04
1.03
5.00
8.10
2.02
1.00
1.99
3.02
f1 (ppm)

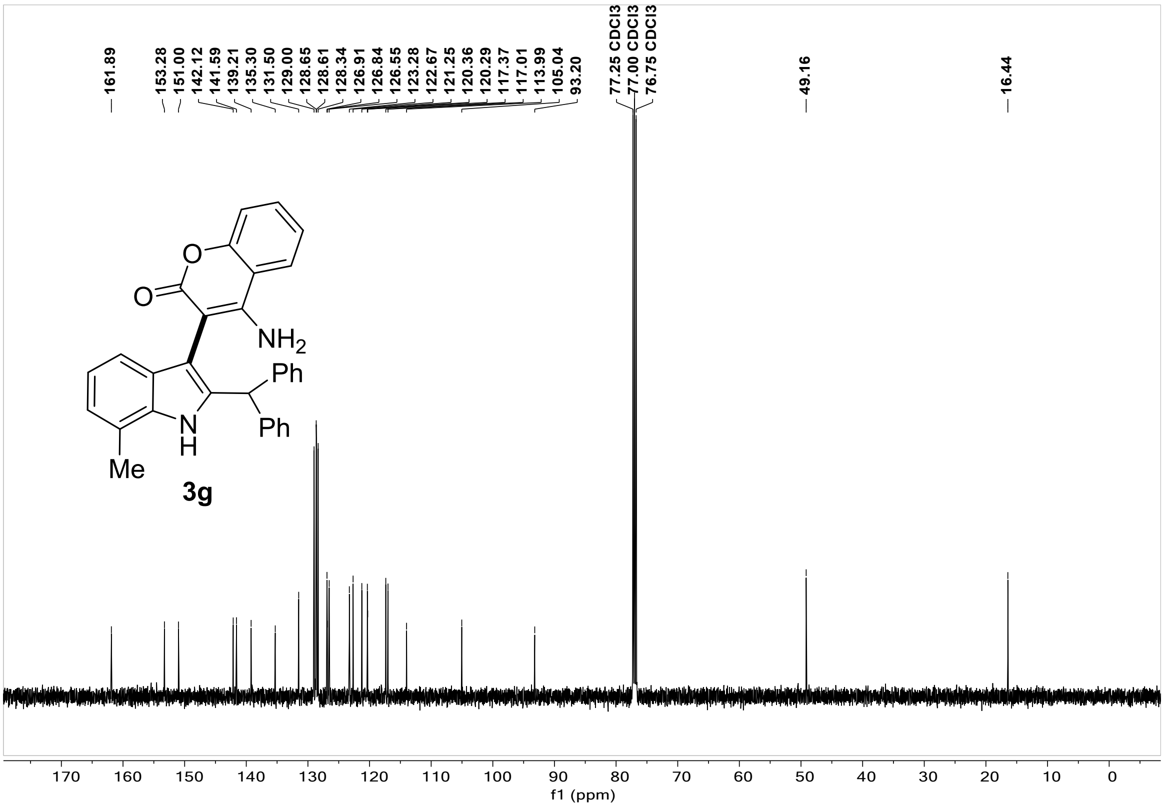
161.89
153.28
151.00
142.12
141.59
139.21
135.30
131.50
129.00
128.65
128.61
128.34
126.91
126.84
126.55
123.28
122.67
121.25
120.36
120.29
117.37
117.01
113.99
105.04
93.20
77.25 CDCl3
77.00 CDCl3
76.75 CDCl3
49.16
16.44
O
O
NH2
Ph
Ph
N
H
Me
3g
f1 (ppm)

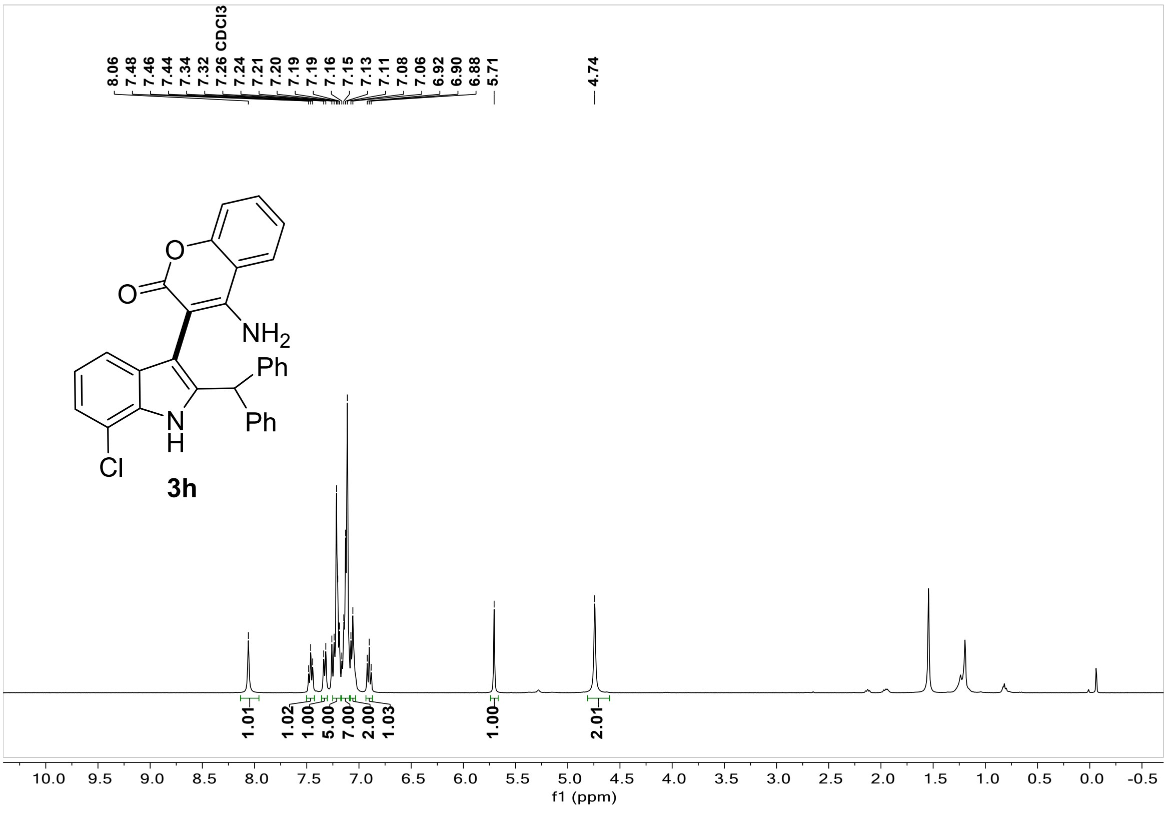
8.06
7.48
7.46
7.44
7.34
7.32
7.26 CDCl3
7.24
7.21
7.20
7.19
7.16
7.15
7.13
7.11
7.08
7.06
6.92
6.90
6.88
5.71
4.74
O
O
NH2
Ph
Ph
N
H
Cl
3h
1.01
1.02
1.00
5.00
7.00
2.00
1.03
1.00
2.01
f1 (ppm)

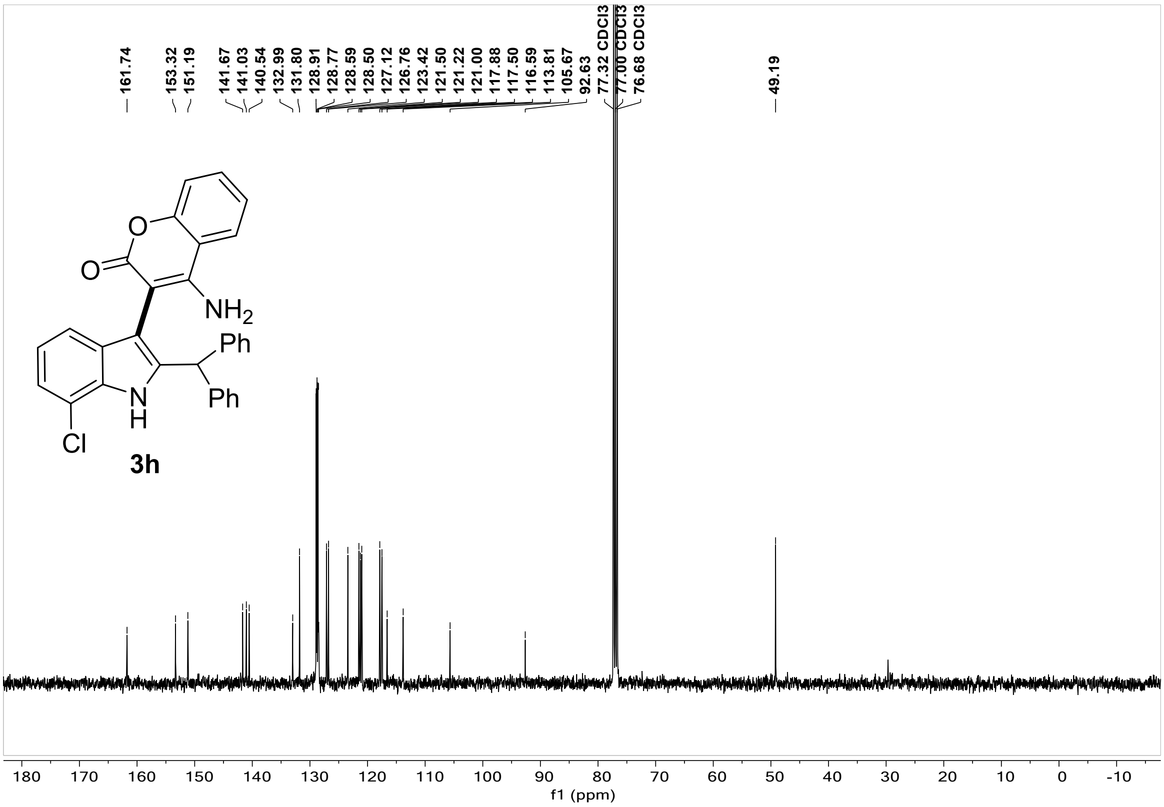
161.74
153.32
151.19
141.67
141.03
140.54
132.99
131.80
128.91
128.77
128.59
128.50
127.12
126.76
123.42
121.50
121.22
121.00
117.88
117.50
116.59
113.81
105.67
92.63
77.32 CDCl3
77.00 CDCl3
76.68 CDCl3
49.19
O
O
NH2
Ph
Ph
N
H
Cl
3h
f1 (ppm)

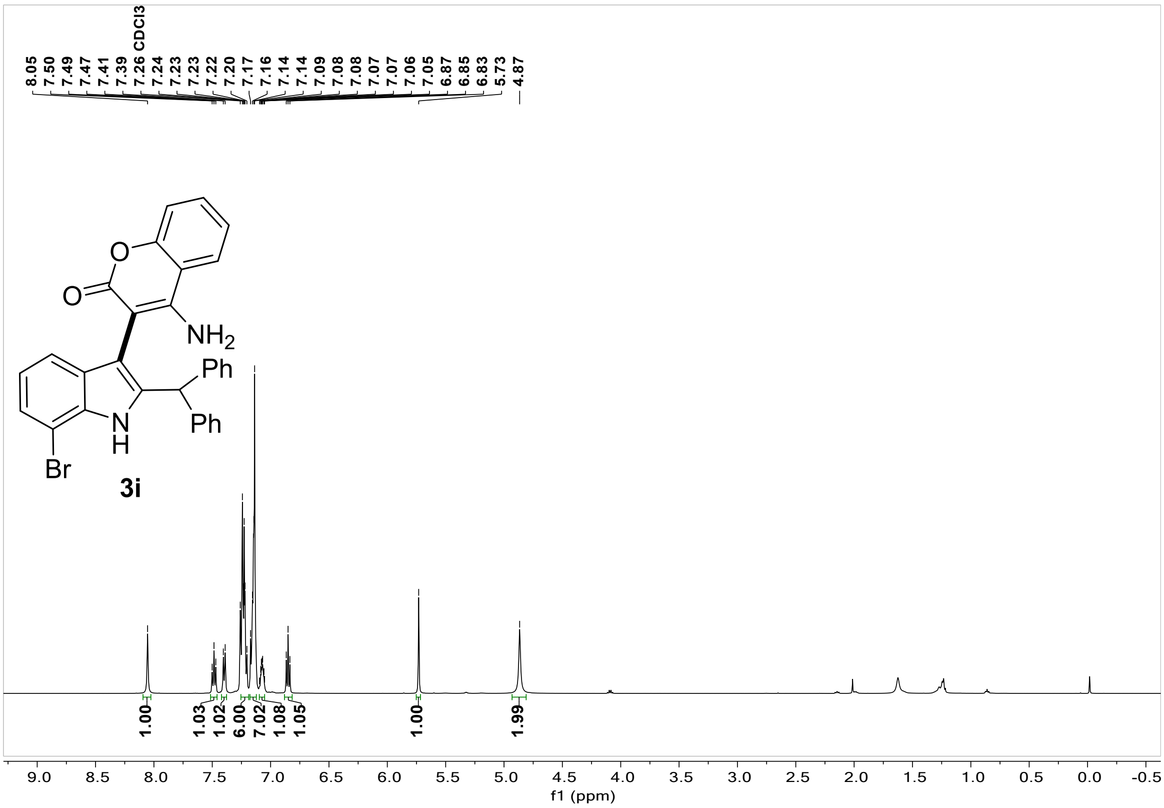
8.05
7.50
7.49
7.47
7.41
7.39
7.26 CDCl3
7.24
7.23
7.23
7.22
7.20
7.17
7.16
7.14
7.14
7.09
7.08
7.08
7.07
7.07
7.06
7.05
6.87
6.85
6.83
5.73
4.87
O
O
NH2
Ph
Ph
N
H
Br
3i
1.00
1.03
1.02
6.00
7.02
1.08
1.05
1.00
1.99
f1 (ppm)

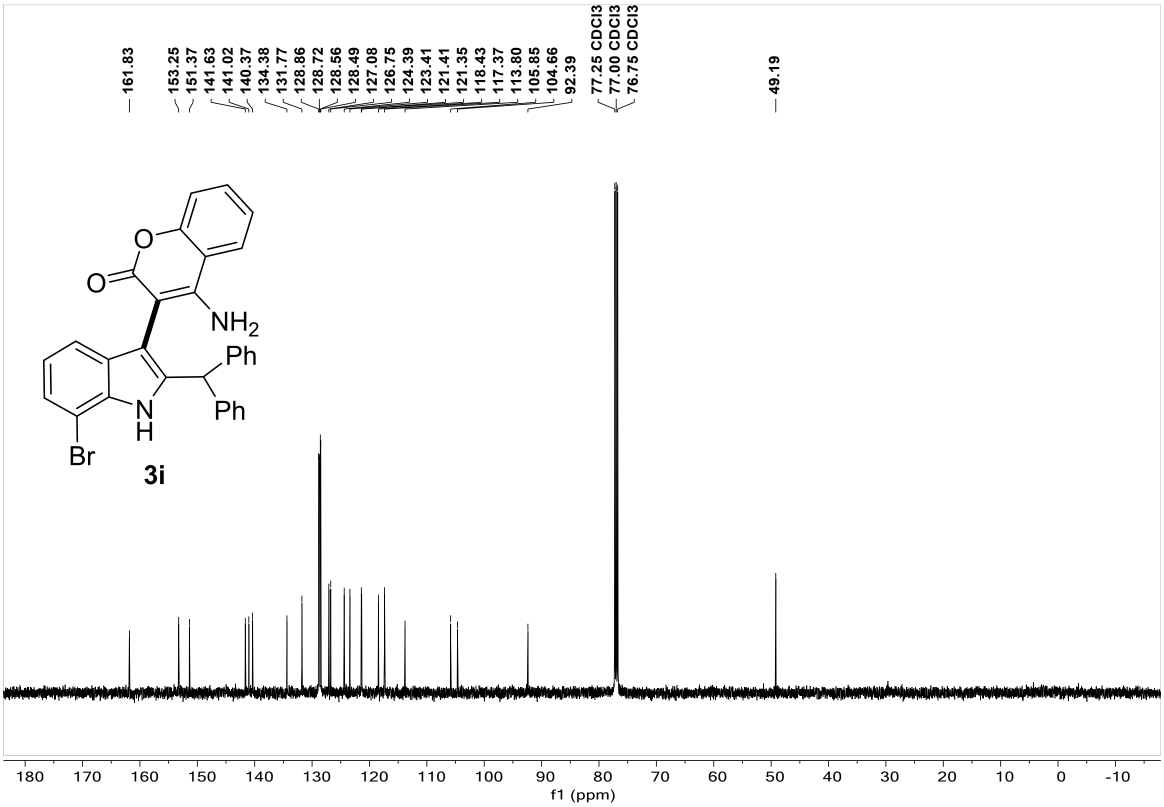
161.83
153.25
151.37
141.63
141.02
140.37
134.38
131.77
128.86
128.72
128.56
128.49
127.08
126.75
124.39
123.41
121.41
121.35
118.43
117.37
113.80
105.85
104.66
92.39
77.25 CDCl3
77.00 CDCl3
76.75 CDCl3
49.19
O
O
NH2
Ph
Ph
N
H
Br
3i
180 170 160 150 140 130 120 110 100 90 80 70 60 50 40 30 20 10 0 -10
f1 (ppm)

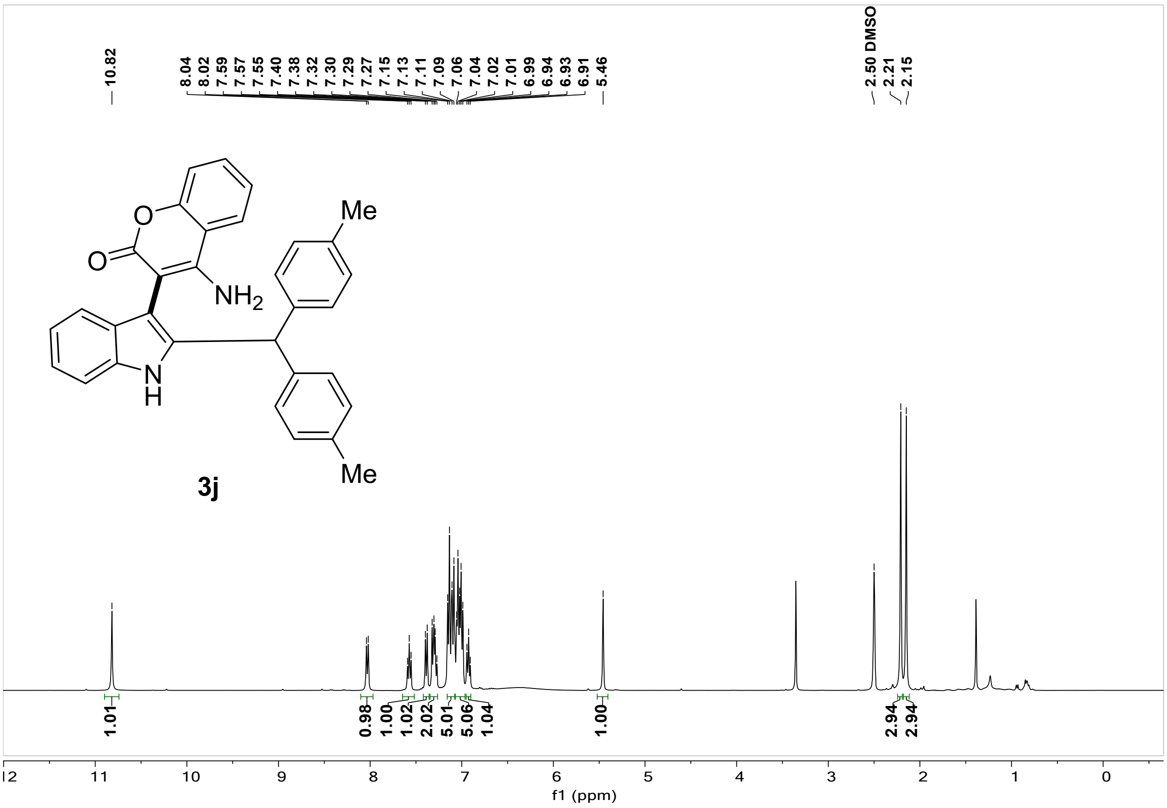
10.82
8.04
8.02
7.59
7.57
7.55
7.40
7.38
7.32
7.30
7.29
7.27
7.15
7.13
7.11
7.09
7.06
7.04
7.02
7.01
6.99
6.94
6.93
6.91
5.46
2.50 DMSO
2.21
2.15
O
O
NH2
Me
Me
N
H
3j
1.01
0.98
1.00
1.02
2.02
5.01
5.06
1.04
1.00
2.94
2.94
12 11 10 9 8 7 6 5 4 3 2 1 0
f1 (ppm)

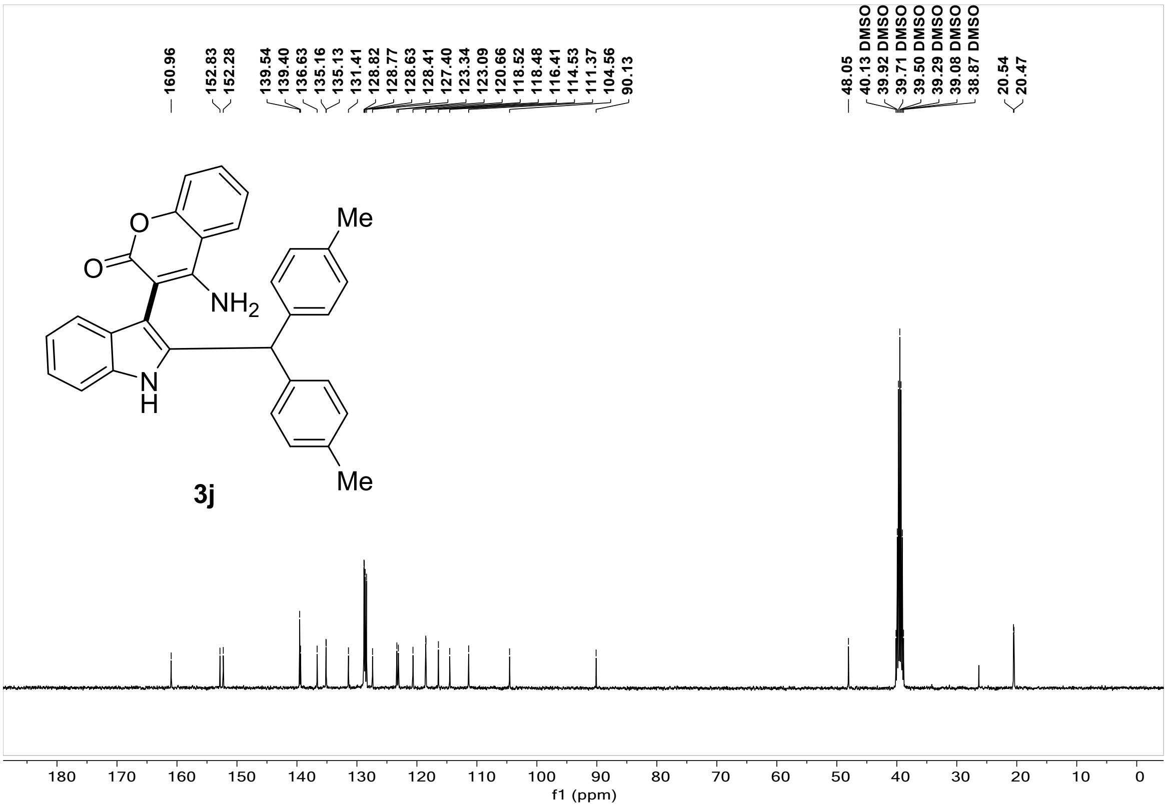
160.96
152.83
152.28
139.54
139.40
136.63
135.16
135.13
131.41
128.82
128.77
128.63
128.41
127.40
123.34
123.09
120.66
118.52
118.48
116.41
114.53
111.37
104.56
90.13
48.05
40.13 DMSO
39.92 DMSO
39.71 DMSO
39.50 DMSO
39.29 DMSO
39.08 DMSO
38.87 DMSO
20.54
20.47
Me
O
O
NH2
N
H
Me
f1 (ppm)

3j

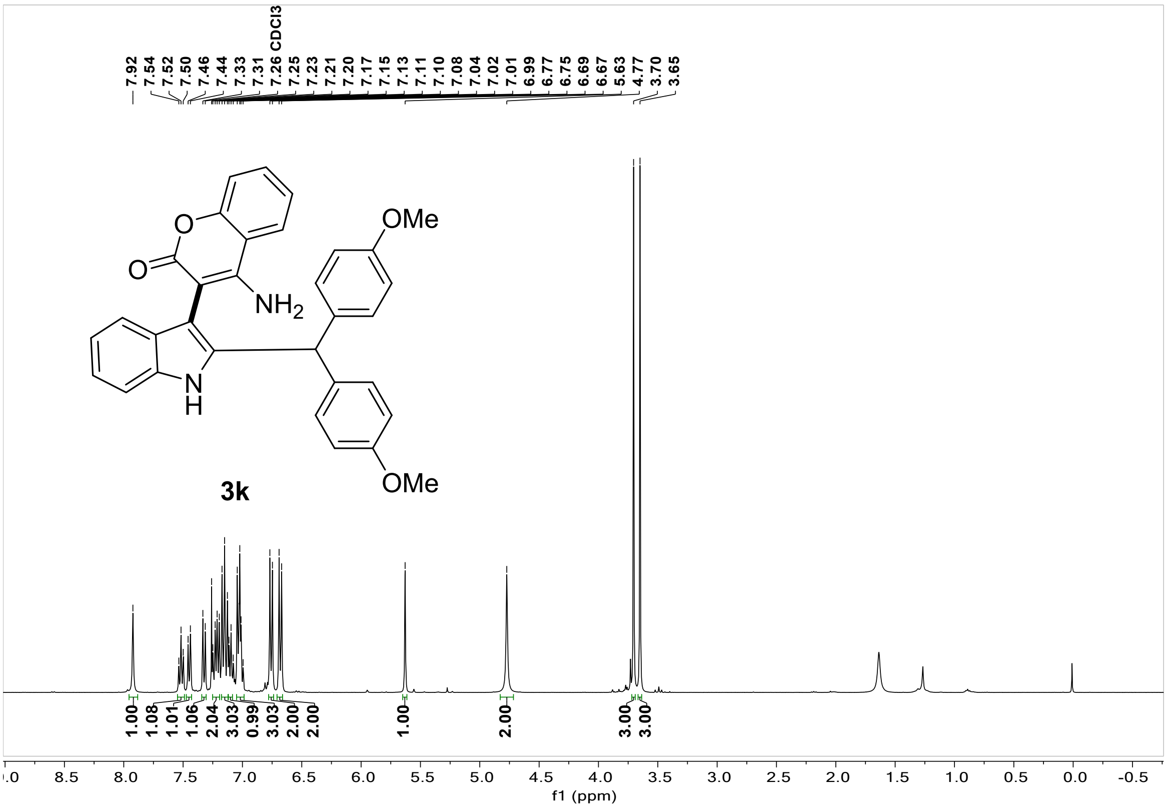
7.92
7.54
7.52
7.50
7.46
7.44
7.33
7.31
7.26 CDCl3
7.25
7.23
7.21
7.20
7.17
7.15
7.13
7.11
7.10
7.08
7.04
7.02
7.01
6.99
6.77
6.75
6.69
6.67
5.63
4.77
3.70
3.65
OMe
O
O
NH2
N
H
OMe
1.00
1.08
1.01
1.06
2.04
3.03
0.99
3.03
2.00
2.00
1.00
2.00
3.00
3.00
f1 (ppm)

3k

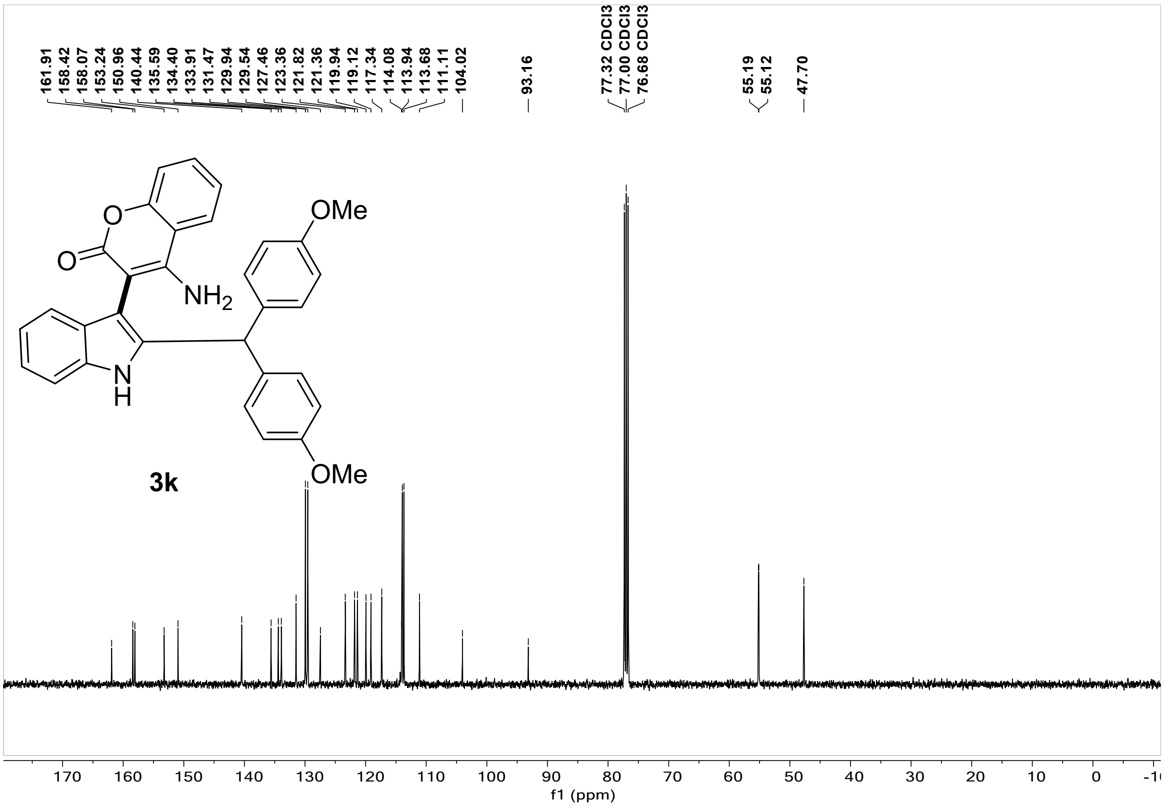
161.91
158.42
158.07
153.24
150.96
140.44
135.59
134.40
133.91
131.47
129.94
129.54
127.46
123.36
121.82
121.36
119.94
119.12
117.34
114.08
113.94
113.68
111.11
104.02
93.16
77.32 CDCl3
77.00 CDCl3
76.68 CDCl3
55.19
55.12
47.70
OMe
O
O
NH2
N
H
OMe
3k
f1 (ppm)

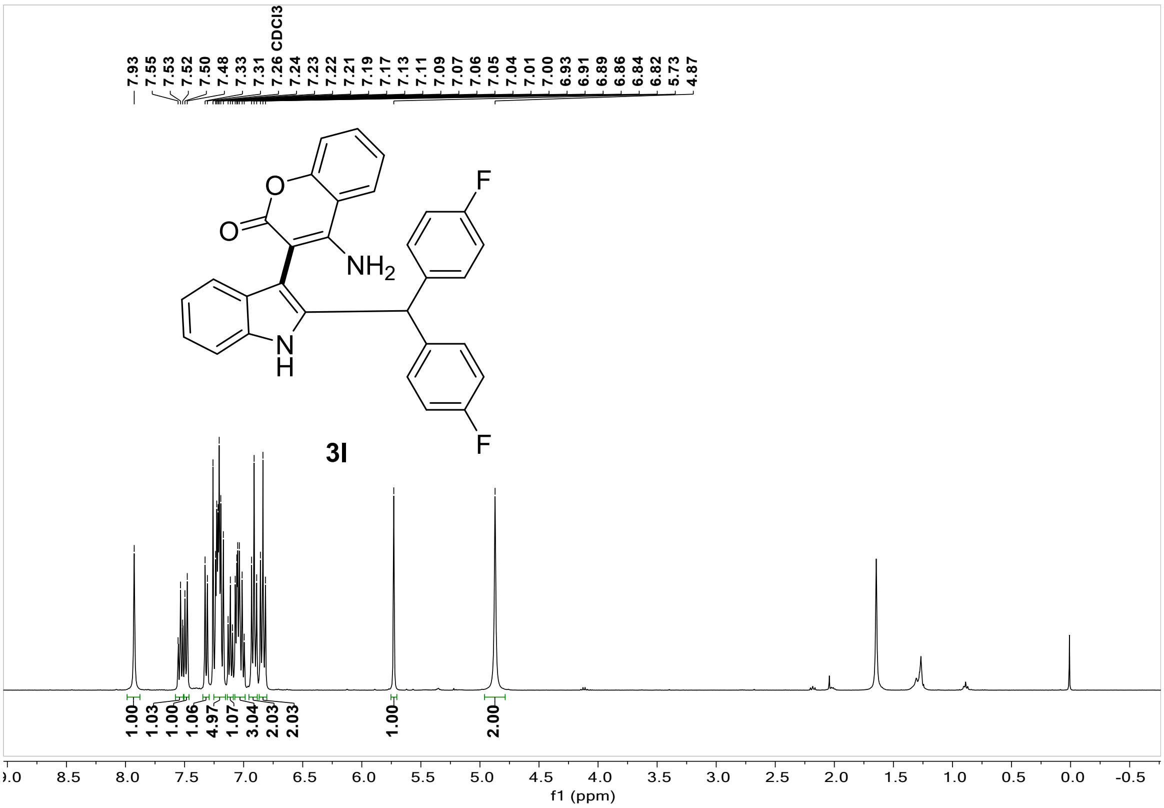
7.93
7.55
7.53
7.52
7.50
7.48
7.33
7.31
7.26 CDCl3
7.24
7.23
7.22
7.21
7.19
7.17
7.13
7.11
7.09
7.07
7.06
7.05
7.04
7.01
7.00
6.93
6.91
6.89
6.86
6.84
6.82
5.73
4.87
F
O
O
NH2
N
H
F
3l
1.00
1.03
1.00
1.06
4.97
1.07
3.04
2.03
2.03
1.00
2.00
f1 (ppm)

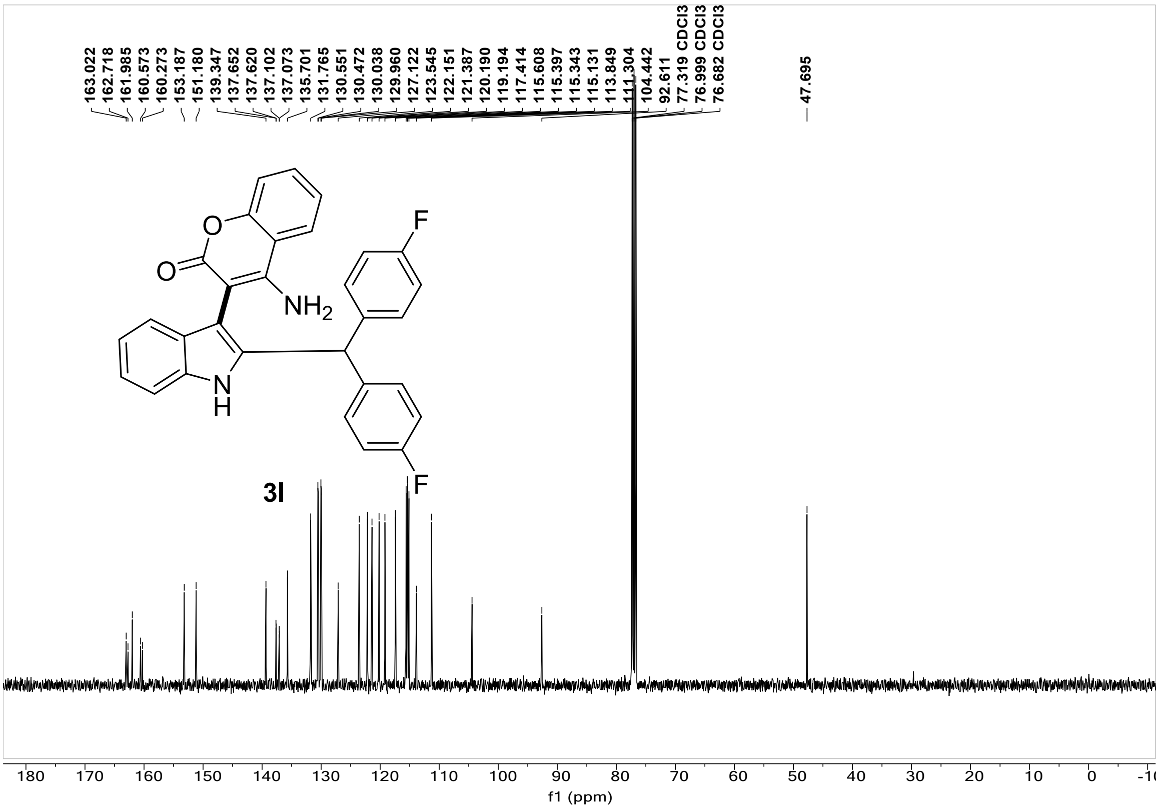
163.022
162.718
161.985
160.573
160.273
153.187
151.180
139.347
137.652
137.620
137.102
137.073
135.701
131.765
130.551
130.472
130.038
129.960
127.122
123.545
122.151
121.387
120.190
119.194
117.414
115.608
115.397
115.343
115.131
113.849
111.304
104.442
92.611
77.319 CDCl3
76.999 CDCl3
76.682 CDCl3
47.695
O
O
NH2
F
N
H
F
3l
180 170 160 150 140 130 120 110 100 90 80 70 60 50 40 30 20 10 0
f1 (ppm)

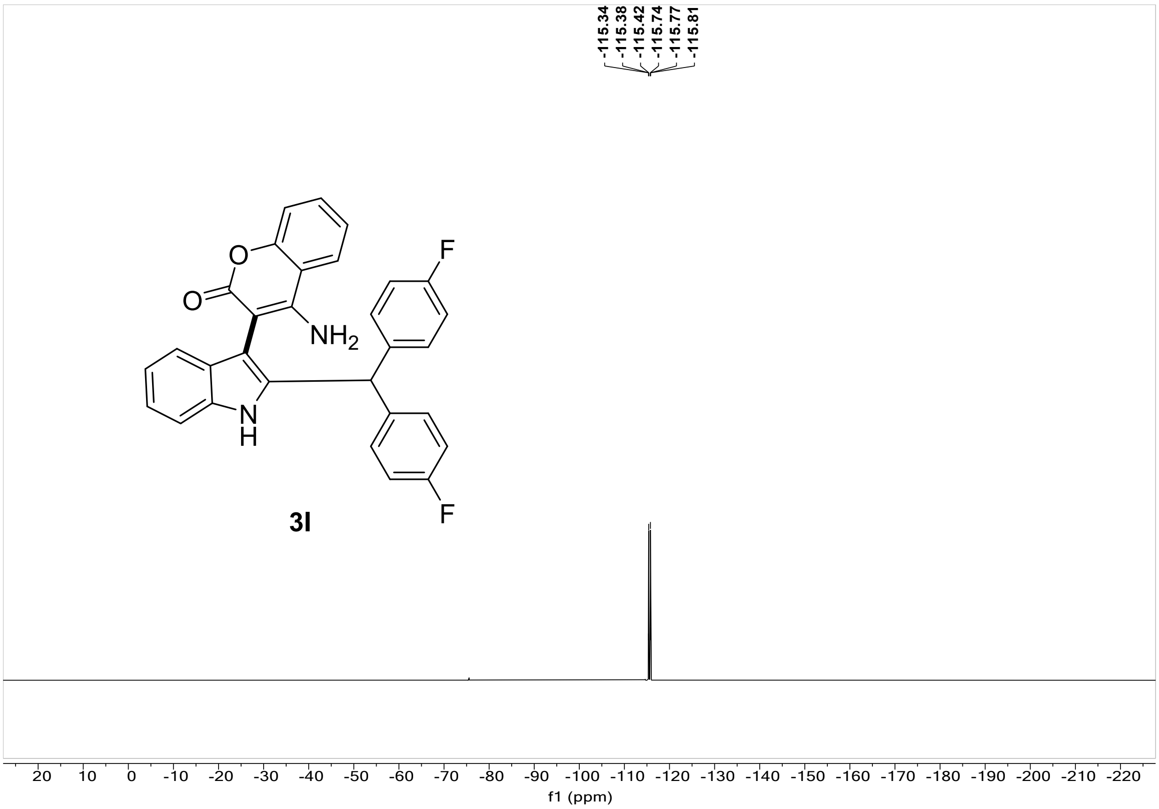
-115.34
-115.38
-115.42
-115.74
-115.77
-115.81
O
O
NH2
F
N
H
F
3l
20 10 0 -10 -20 -30 -40 -50 -60 -70 -80 -90 -100 -110 -120 -130 -140 -150 -160 -170 -180 -190 -200 -210 -220
f1 (ppm)

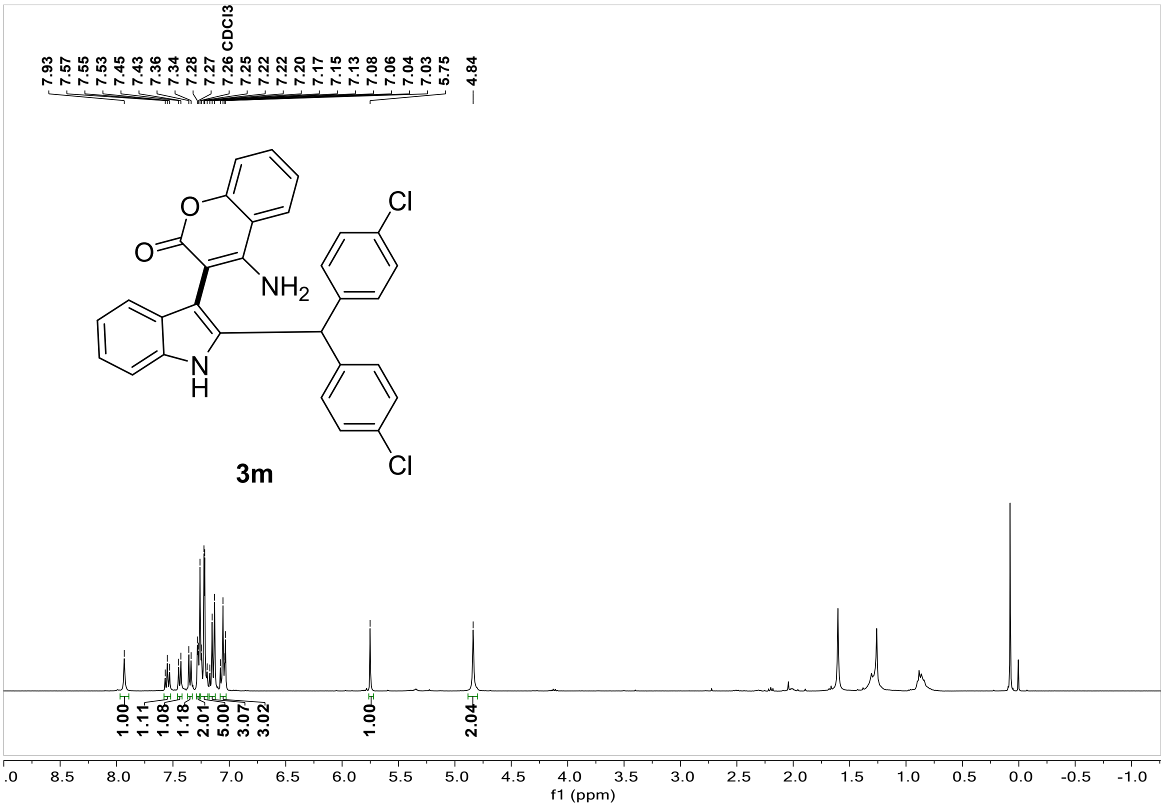
7.93
7.57
7.55
7.53
7.45
7.43
7.36
7.34
7.28
7.27
7.26 CDCl3
7.25
7.22
7.22
7.20
7.17
7.15
7.13
7.08
7.06
7.04
7.03
5.75
4.84
O
O
NH2
Cl
N
H
Cl
3m
1.00
1.11
1.08
1.18
2.01
5.00
3.07
3.02
1.00
2.04
f1 (ppm)

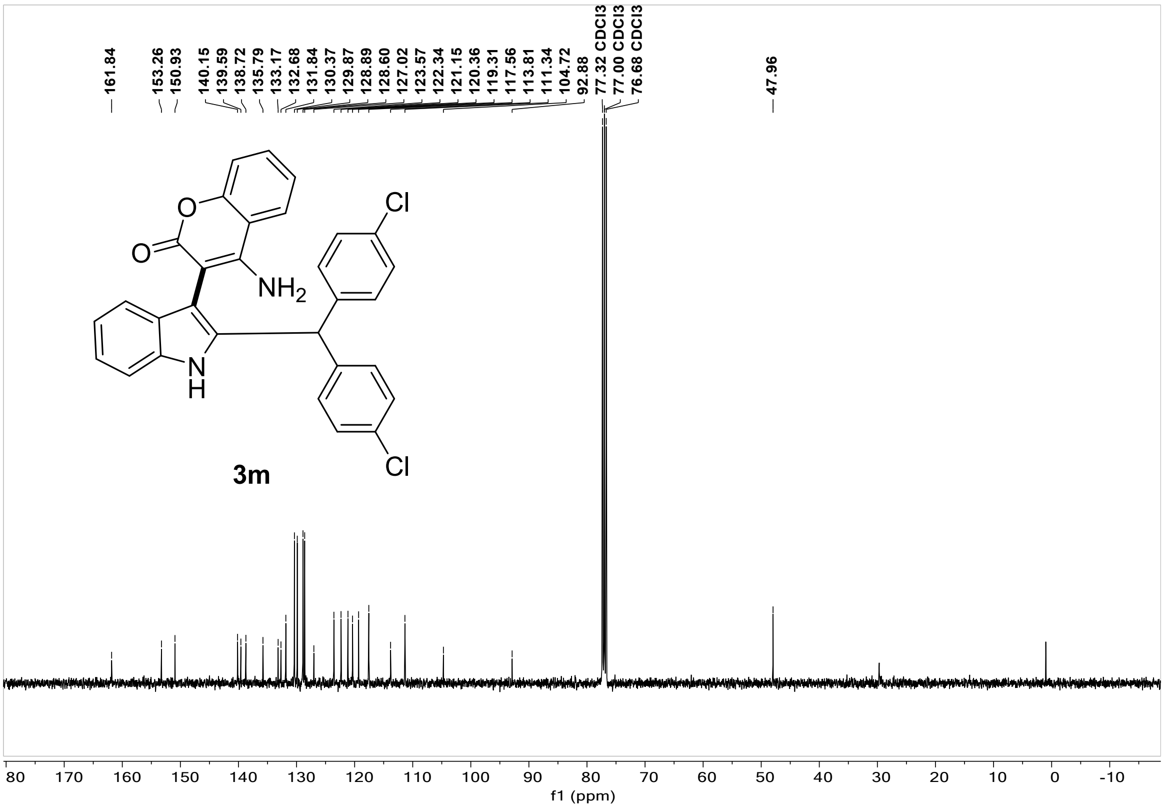
161.84
153.26
150.93
140.15
139.59
138.72
135.79
133.17
132.68
131.84
130.37
129.87
128.89
128.60
127.02
123.57
122.34
121.15
120.36
119.31
117.56
113.81
111.34
104.72
92.88
77.32 CDCl3
77.00 CDCl3
76.68 CDCl3
47.96
O
O
NH2
Cl
N
H
Cl
3m
f1 (ppm)

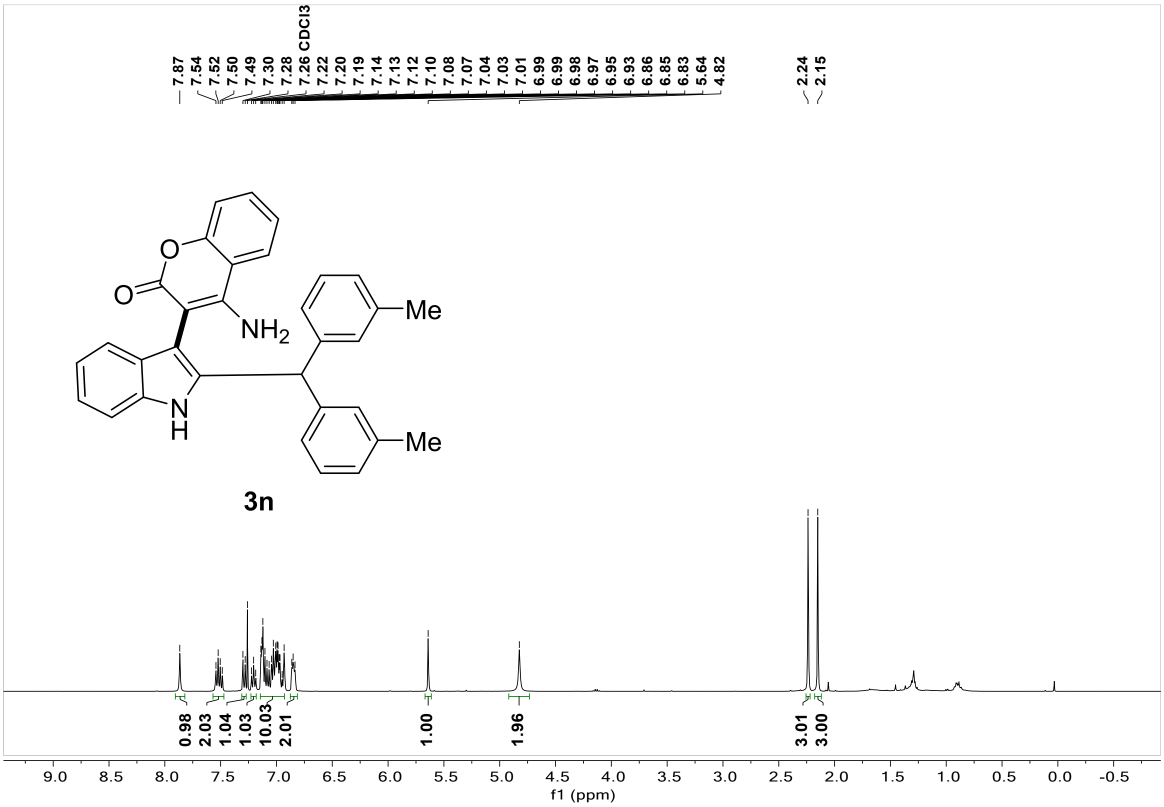

3n

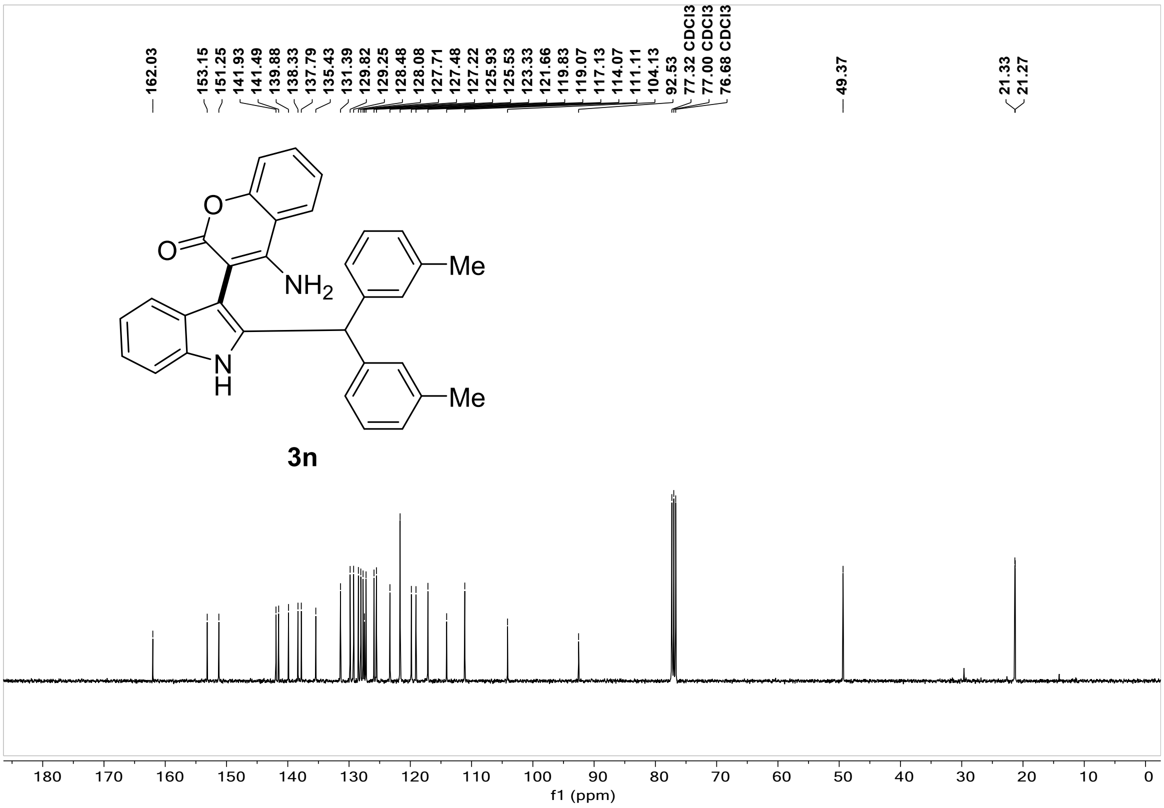

3n

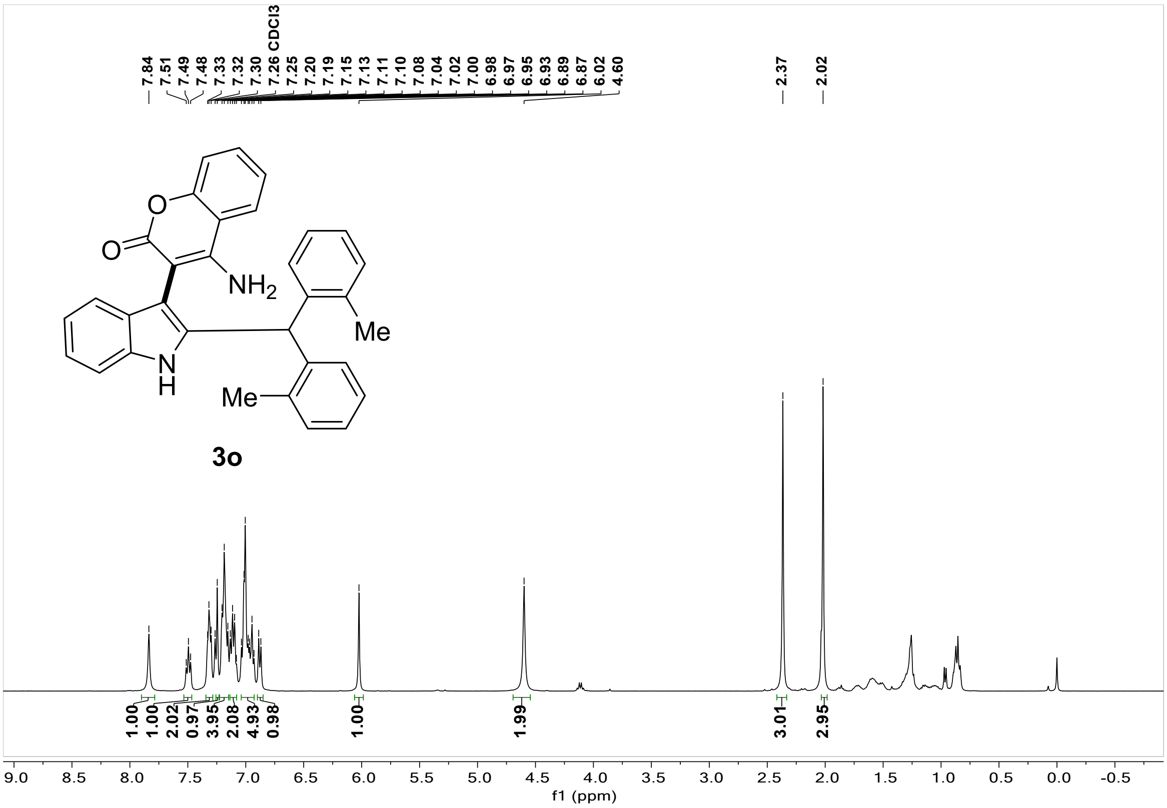
7.84 7.51 7.49 7.48 7.33 7.32 7.30 7.26 CDCl3 7.25 7.20 7.19 7.15 7.13 7.11 7.10 7.08 7.04 7.02 7.00 6.98 6.97 6.95 6.93 6.89 6.87 6.02 4.60
2.37 2.02
O
O
NH2
Me
N
H
Me
3o
1.00 1.00 2.02 0.97 3.95 2.08 4.93 0.98
1.00
1.99
3.01 2.95
9.0 8.5 8.0 7.5 7.0 6.5 6.0 5.5 5.0 4.5 4.0 3.5 3.0 2.5 2.0 1.5 1.0 0.5 0.0 -0.5
f1 (ppm)

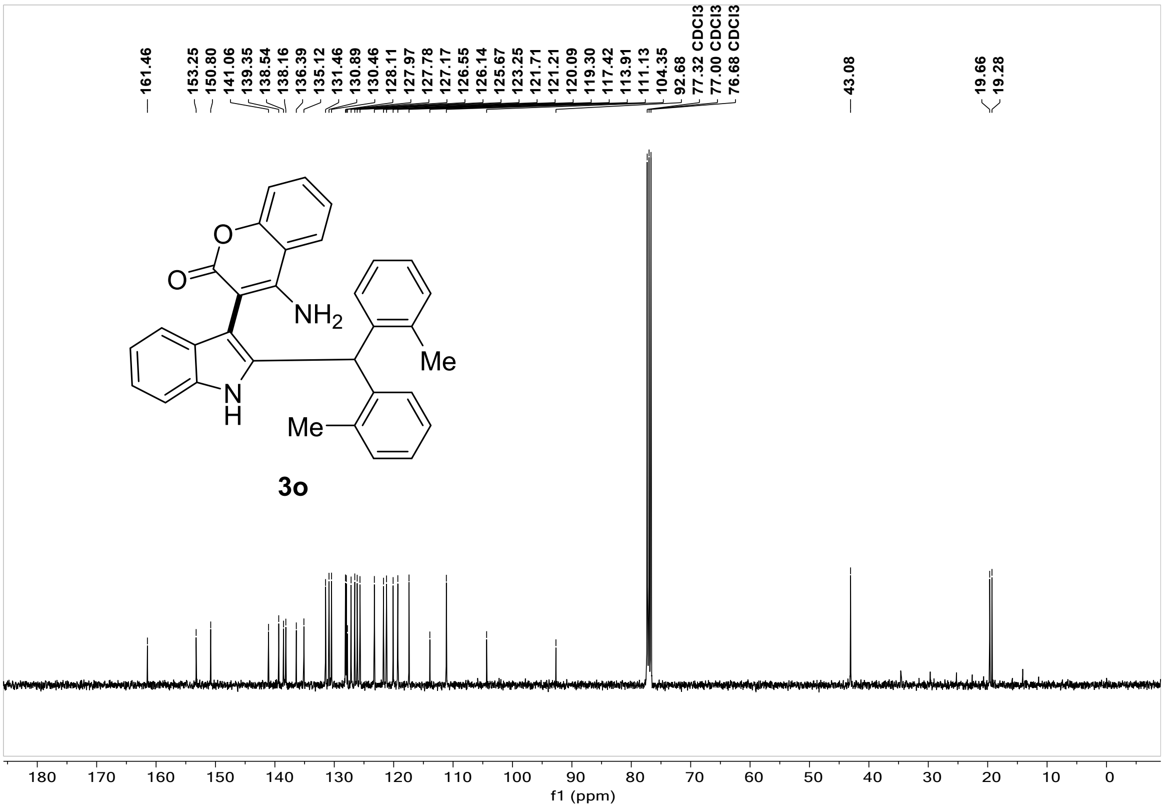
161.46
153.25 150.80 141.06 139.35 138.54 138.16 136.39 135.12 131.46 130.89 130.46 128.11 127.97 127.78 127.17 126.55 126.14 125.67 123.25 121.71 121.21 120.09 119.30 117.42 113.91 111.13 104.35 92.68 77.32 CDCl3 77.00 CDCl3 76.68 CDCl3
43.08
19.66 19.28
O
O
NH2
Me
N
H
Me
3o
180 170 160 150 140 130 120 110 100 90 80 70 60 50 40 30 20 10 0
f1 (ppm)

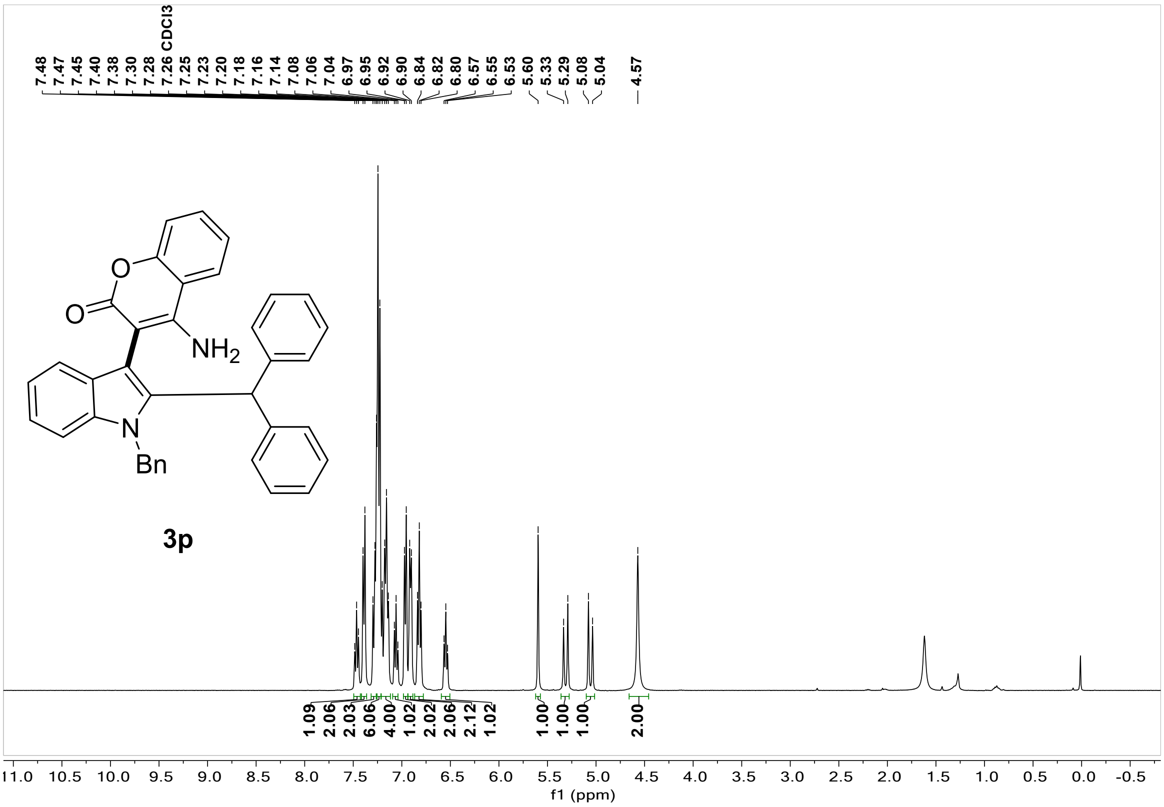
7.48
7.47
7.45
7.40
7.38
7.30
7.28
7.26 CDCl3
7.25
7.23
7.20
7.18
7.16
7.14
7.08
7.06
7.04
6.97
6.95
6.92
6.90
6.84
6.82
6.80
6.57
6.55
6.53
5.60
5.33
5.29
5.08
5.04
4.57
NH2
O
O
N
Bn
3p
1.09
2.06
2.03
6.06
4.00
1.02
2.02
2.06
2.12
1.02
1.00
1.00
1.00
2.00
f1 (ppm)

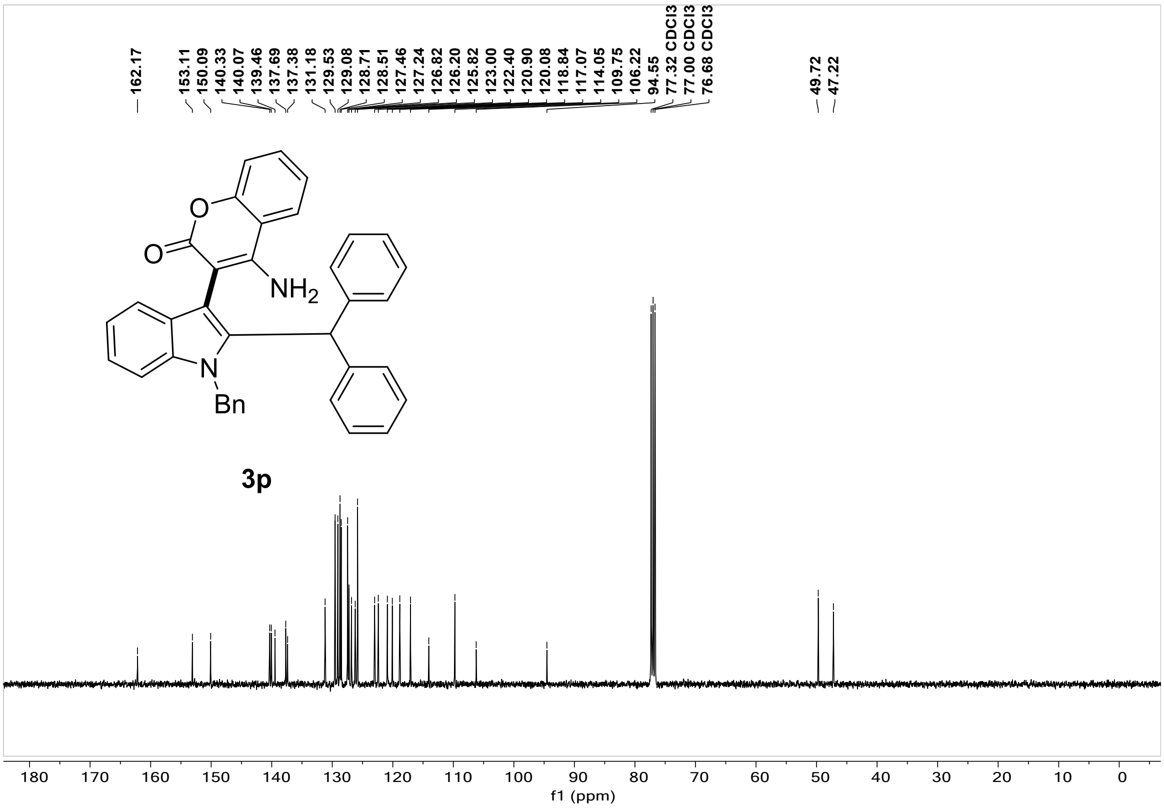
162.17
153.11
150.09
140.33
140.07
139.46
137.69
137.38
131.18
129.53
129.08
128.71
128.51
127.46
127.24
126.82
126.20
125.82
123.00
122.40
120.90
120.08
118.84
117.07
114.05
109.75
106.22
94.55
77.32 CDCl3
77.00 CDCl3
76.68 CDCl3
49.72
47.22
NH2
O
O
N
Bn
3p
f1 (ppm)

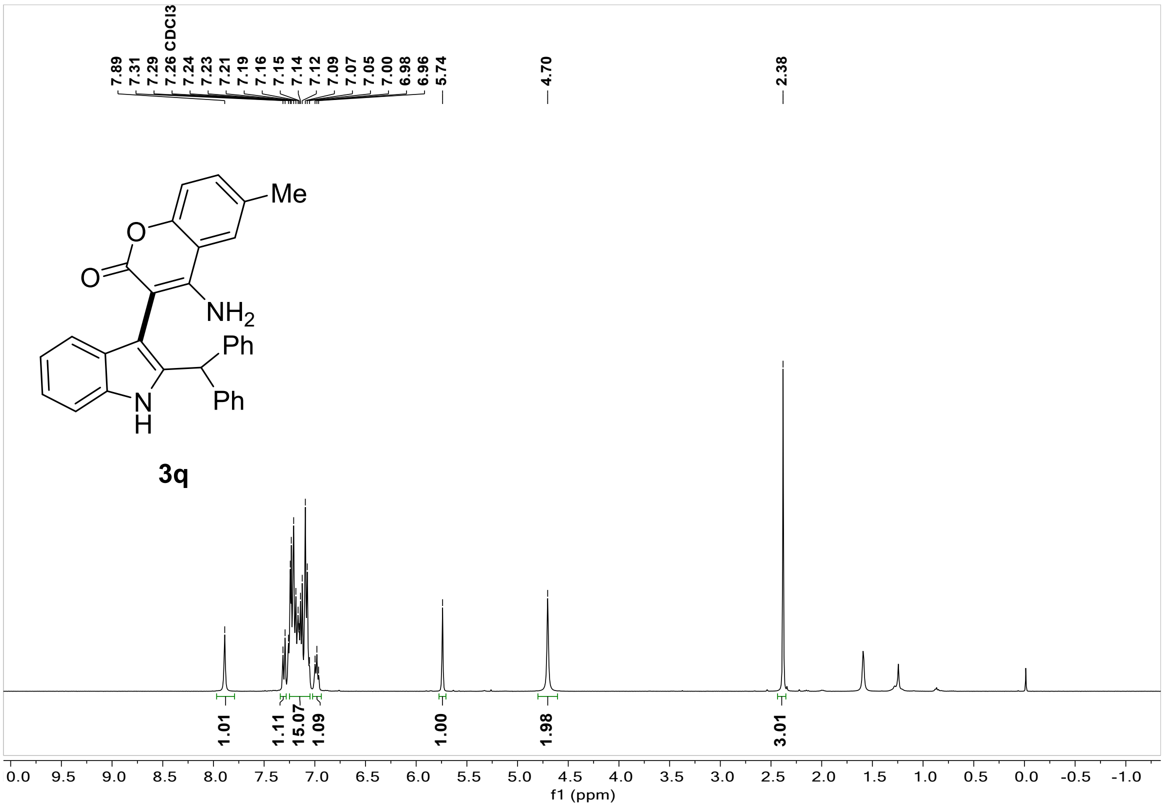
7.89
7.31
7.29
7.26 CDCl3
7.24
7.23
7.21
7.19
7.16
7.15
7.14
7.12
7.09
7.07
7.05
7.00
6.98
6.96
5.74
4.70
2.38
Me
O
O
NH2
Ph
Ph
N
H
3q
1.01
1.11
15.07
1.09
1.00
1.98
3.01
f1 (ppm)

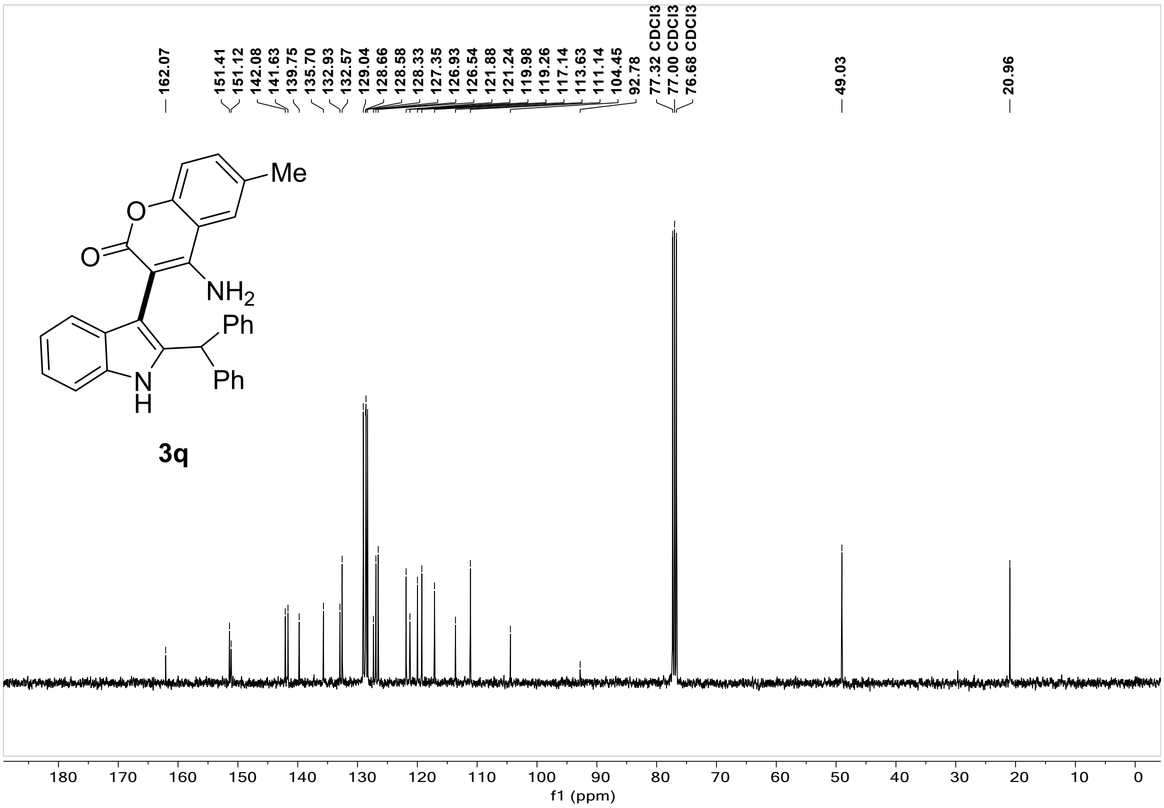
162.07
151.41
151.12
142.08
141.63
139.75
135.70
132.93
132.57
129.04
128.66
128.58
128.33
127.35
126.93
126.54
121.88
121.24
119.98
119.26
117.14
113.63
111.14
104.45
92.78
77.32 CDCl3
77.00 CDCl3
76.68 CDCl3
49.03
20.96
Me
O
O
NH2
Ph
Ph
N
H
3q
f1 (ppm)

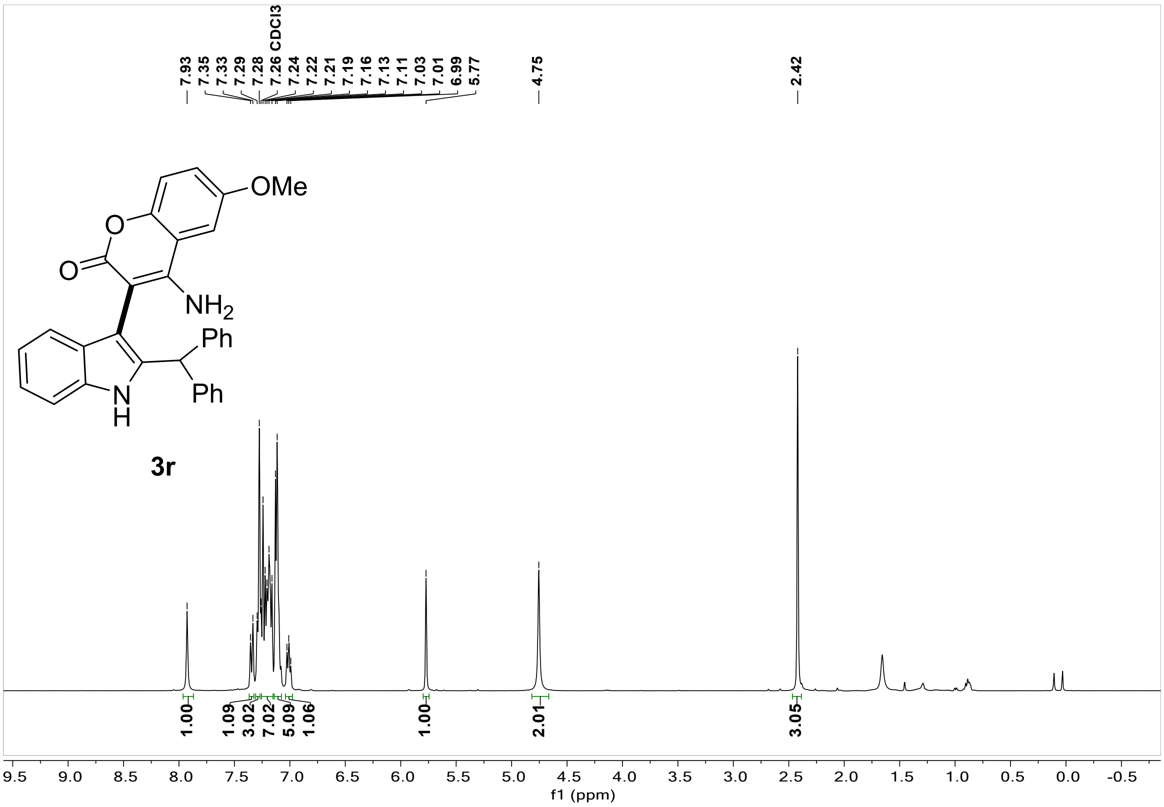
7.93
7.35
7.33
7.29
7.28
7.26 CDCl3
7.24
7.22
7.21
7.19
7.16
7.13
7.11
7.03
7.01
6.99
5.77
4.75
2.42
OMe
O
O
NH2
Ph
Ph
N
H
1.00
1.09
3.02
7.02
5.09
1.06
1.00
2.01
3.05
f1 (ppm)

3r

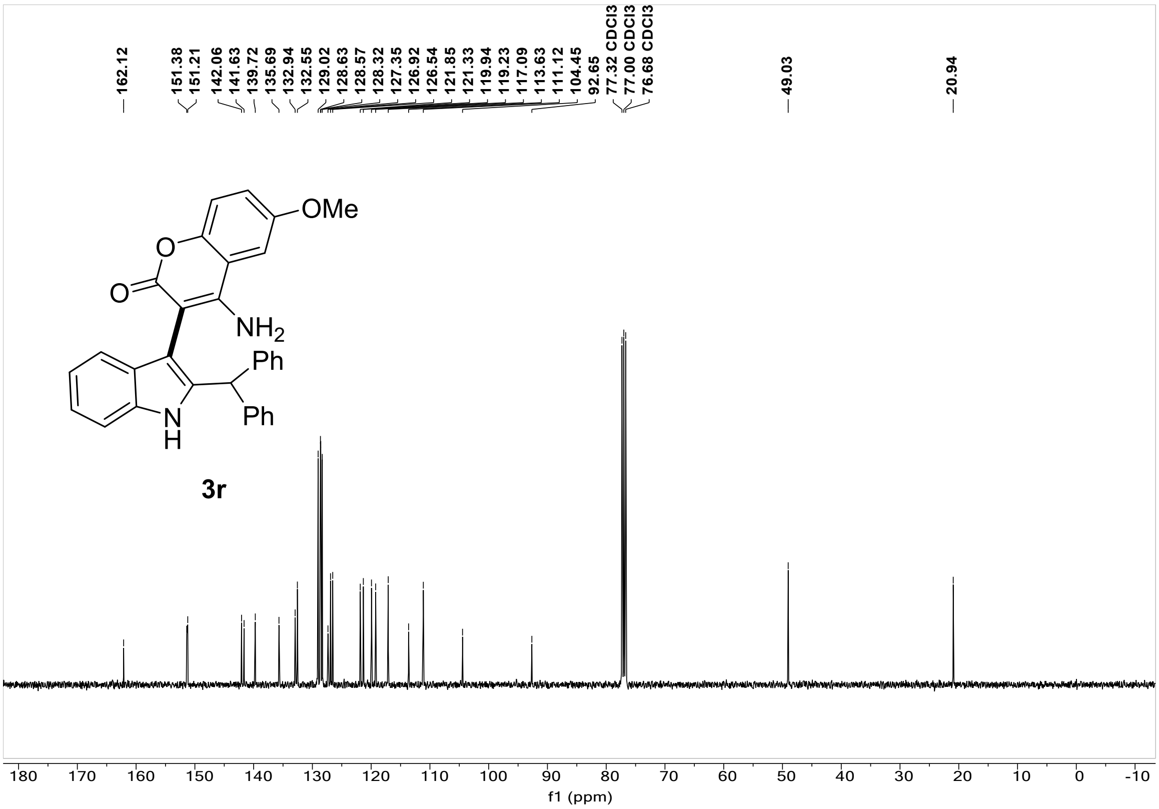
162.12
151.38
151.21
142.06
141.63
139.72
135.69
132.94
132.55
129.02
128.63
128.57
128.32
127.35
126.92
126.54
121.85
121.33
119.94
119.23
117.09
113.63
111.12
104.45
92.65
77.32 CDCl3
77.00 CDCl3
76.68 CDCl3
49.03
20.94
OMe
O
O
NH2
Ph
Ph
N
H
f1 (ppm)

3r

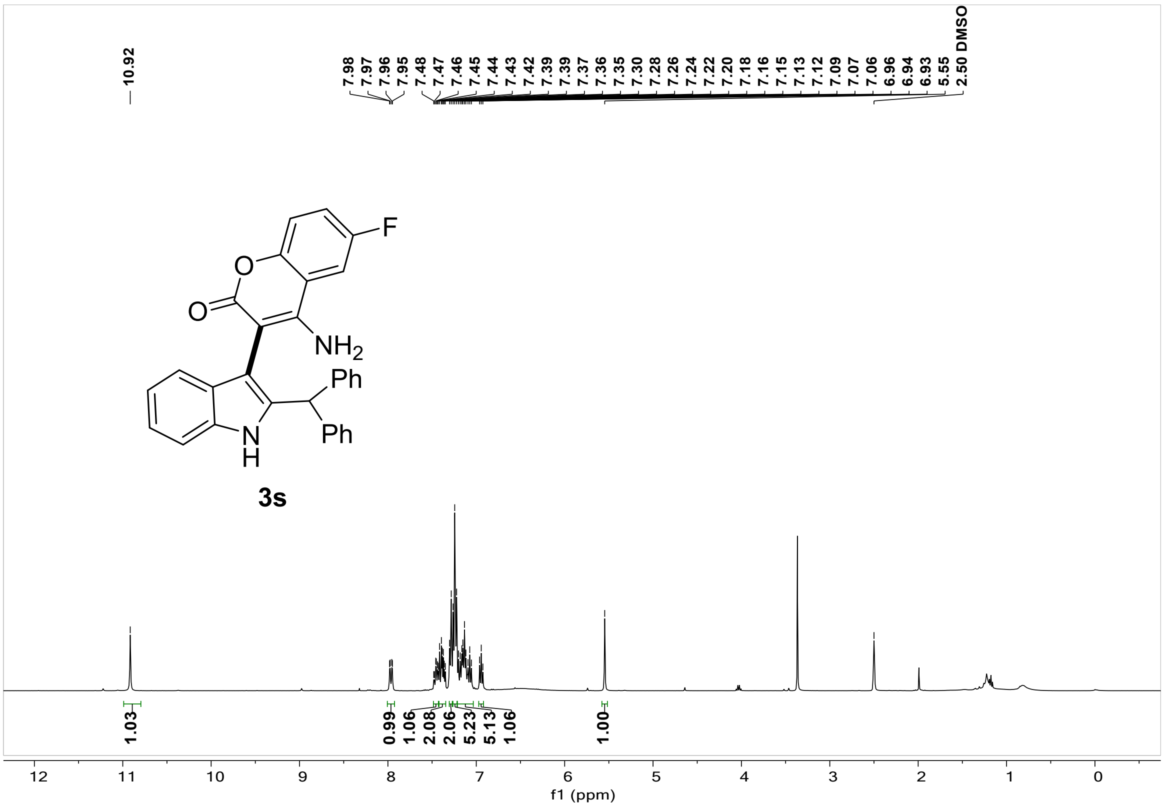
10.92
7.98
7.97
7.96
7.95
7.48
7.47
7.46
7.45
7.44
7.43
7.42
7.39
7.39
7.37
7.36
7.35
7.30
7.28
7.26
7.24
7.22
7.20
7.18
7.16
7.15
7.13
7.12
7.09
7.07
7.06
6.96
6.94
6.93
5.55
2.50 DMSO
F
O
O
NH2
Ph
Ph
N
H
3s
1.03
0.99
1.06
2.08
2.06
5.23
5.13
1.06
1.00
f1 (ppm)

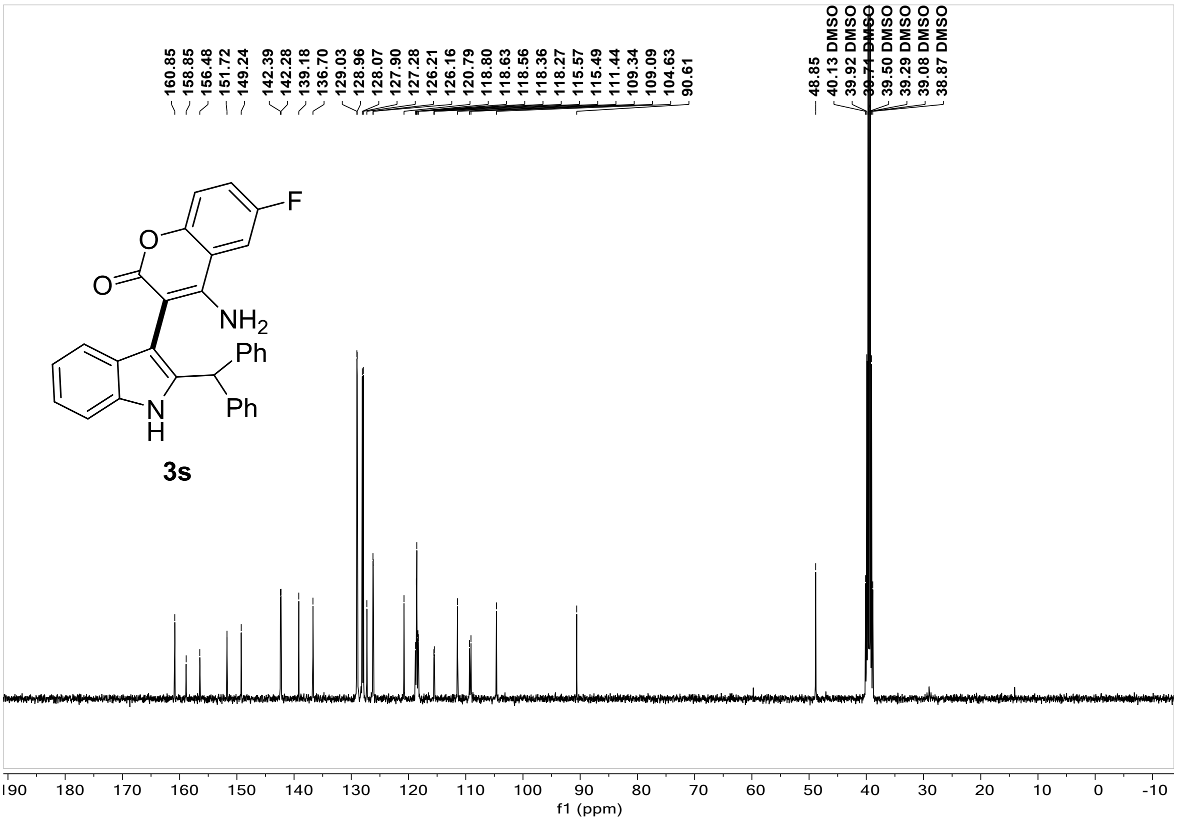
160.85
158.85
156.48
151.72
149.24
142.39
142.28
139.18
136.70
129.03
128.96
128.07
127.90
127.28
126.21
126.16
120.79
118.80
118.63
118.56
118.36
118.27
115.57
115.49
111.44
109.34
109.09
104.63
90.61
48.85
40.13 DMSO
39.92 DMSO
39.74 DMSO
39.50 DMSO
39.29 DMSO
39.08 DMSO
38.87 DMSO
F
O
O
NH2
Ph
Ph
N
H
3s
f1 (ppm)

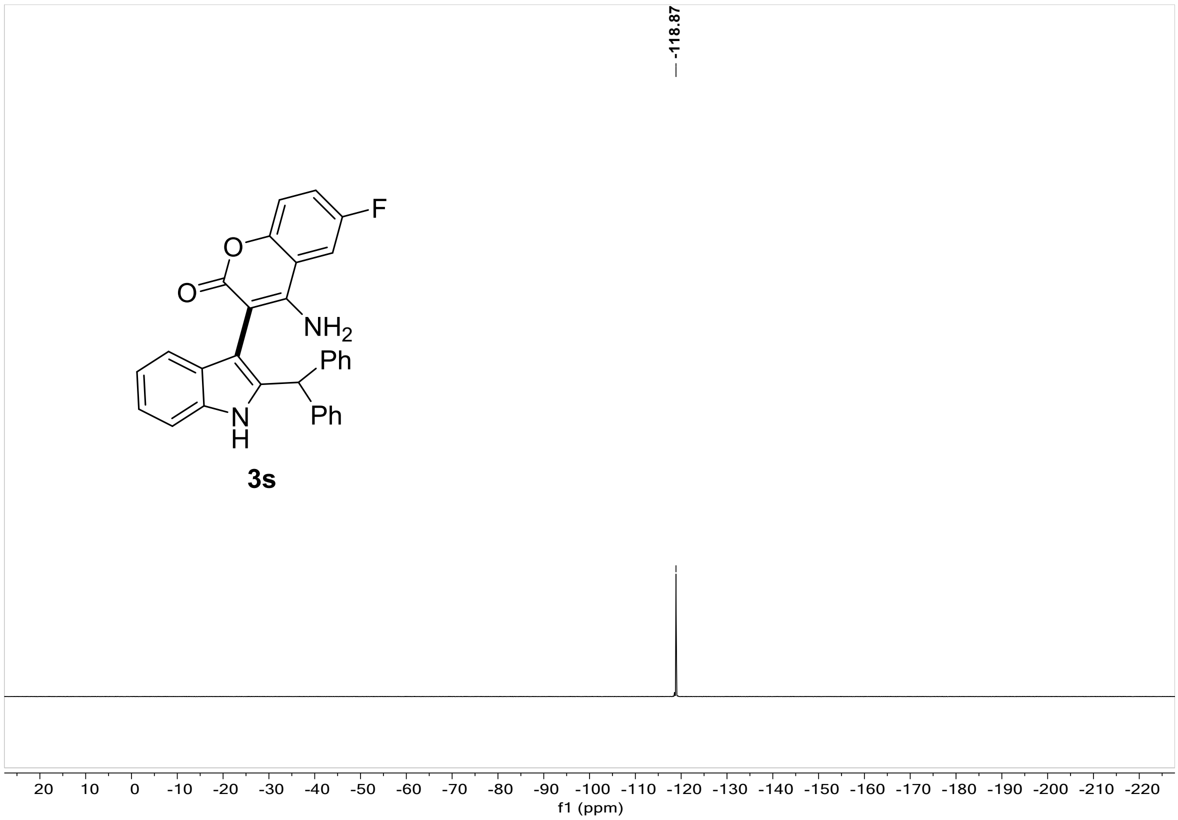
-118.87
F
O
O
NH2
Ph
Ph
N
H
3s
20 10 0 -10 -20 -30 -40 -50 -60 -70 -80 -90 -100 -110 -120 -130 -140 -150 -160 -170 -180 -190 -200 -210 -220
f1 (ppm)

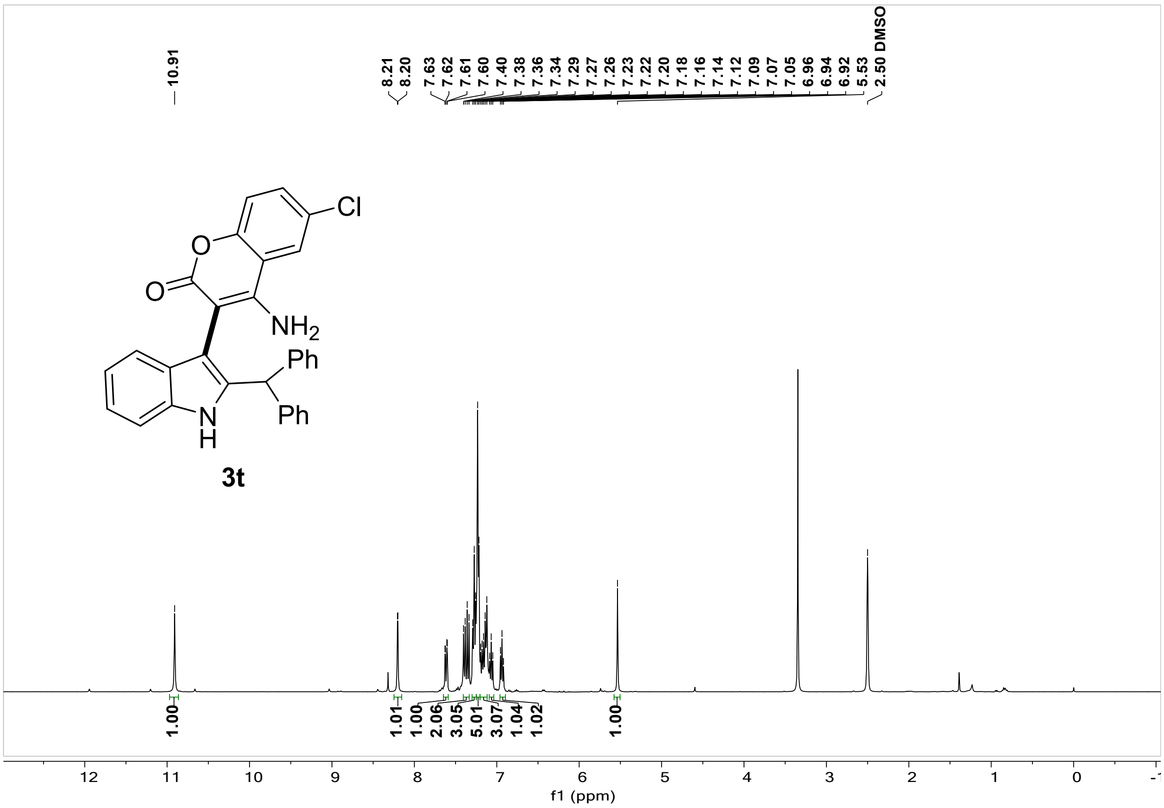
10.91
8.21
8.20
7.63
7.62
7.61
7.60
7.40
7.38
7.36
7.34
7.29
7.27
7.26
7.23
7.22
7.20
7.18
7.16
7.14
7.12
7.09
7.07
7.05
6.96
6.94
6.92
5.53
2.50 DMSO
Cl
O
O
NH2
Ph
Ph
N
H
3t
1.00
1.01
1.00
2.06
3.05
5.01
3.07
1.04
1.02
1.00
12 11 10 9 8 7 6 5 4 3 2 1 0
f1 (ppm)

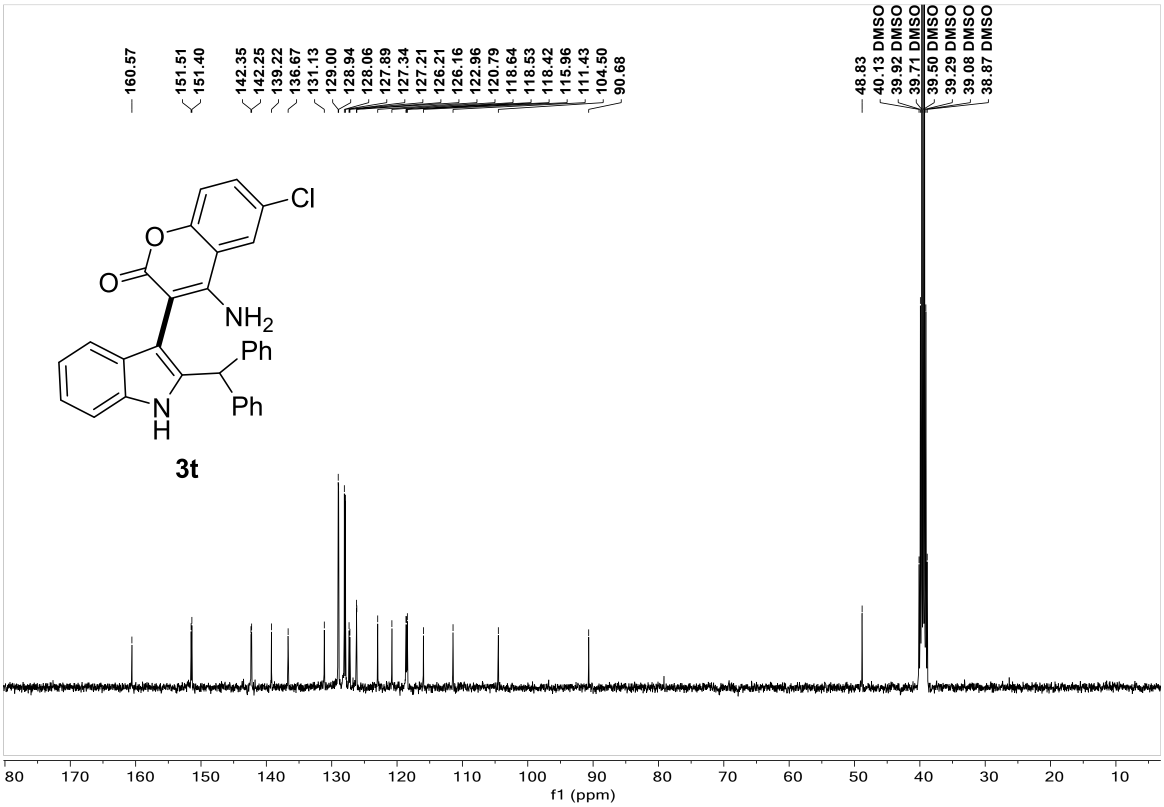
160.57
151.51
151.40
142.35
142.25
139.22
136.67
131.13
129.00
128.94
128.06
127.89
127.34
127.21
126.21
126.16
122.96
120.79
118.64
118.53
118.42
115.96
111.43
104.50
90.68
48.83
40.13 DMSO
39.92 DMSO
39.71 DMSO
39.50 DMSO
39.29 DMSO
39.08 DMSO
38.87 DMSO
Cl
O
O
NH2
Ph
Ph
N
H
3t
f1 (ppm)

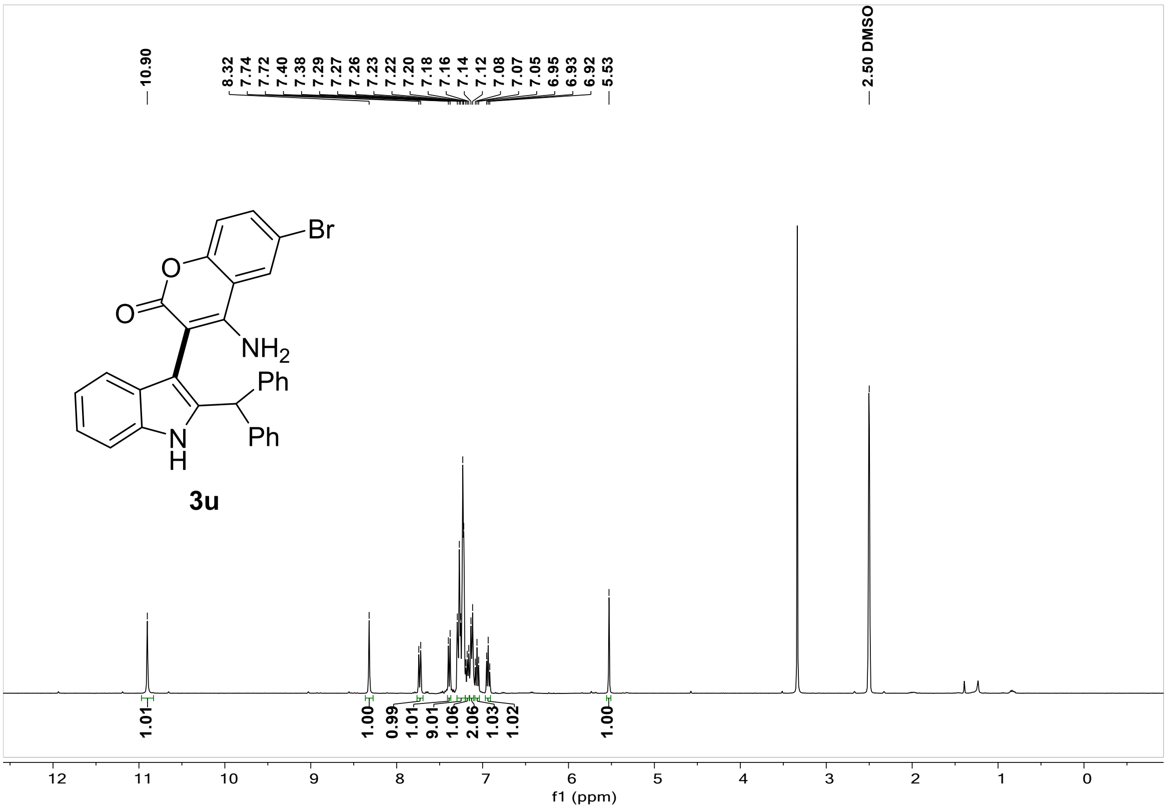
10.90
8.32
7.74
7.72
7.40
7.38
7.29
7.27
7.26
7.23
7.22
7.20
7.18
7.16
7.14
7.12
7.08
7.07
7.05
6.95
6.93
6.92
5.53
2.50 DMSO
Br
O
O
NH2
Ph
Ph
N
H
3u
1.01
1.00
0.99
1.01
9.01
1.06
2.06
1.03
1.02
1.00
f1 (ppm)

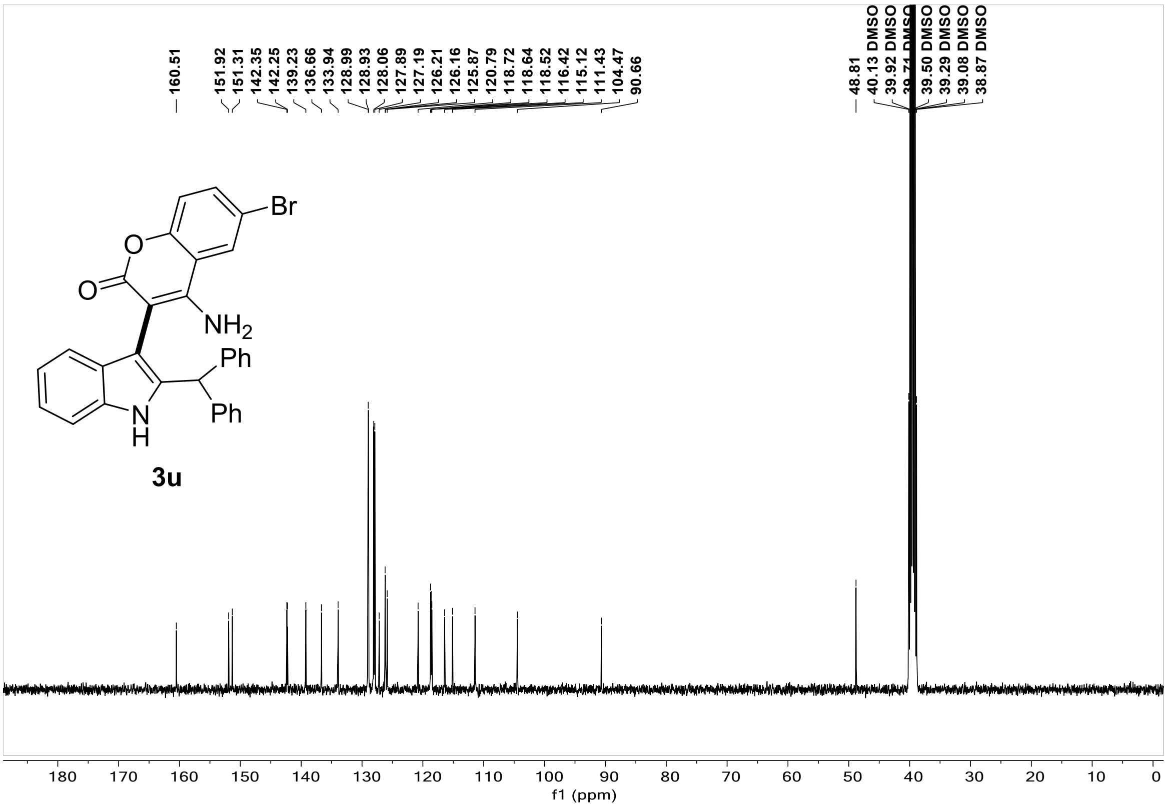
160.51
151.92
151.31
142.35
142.25
139.23
136.66
133.94
128.99
128.93
128.06
127.89
127.19
126.21
126.16
125.87
120.79
118.72
118.64
118.52
116.42
115.12
111.43
104.47
90.66
48.81
40.13 DMSO
39.92 DMSO
39.71 DMSO
39.50 DMSO
39.29 DMSO
39.08 DMSO
38.87 DMSO
Br
O
O
NH2
Ph
Ph
N
H
3u
180 170 160 150 140 130 120 110 100 90 80 70 60 50 40 30 20 10 0
f1 (ppm)

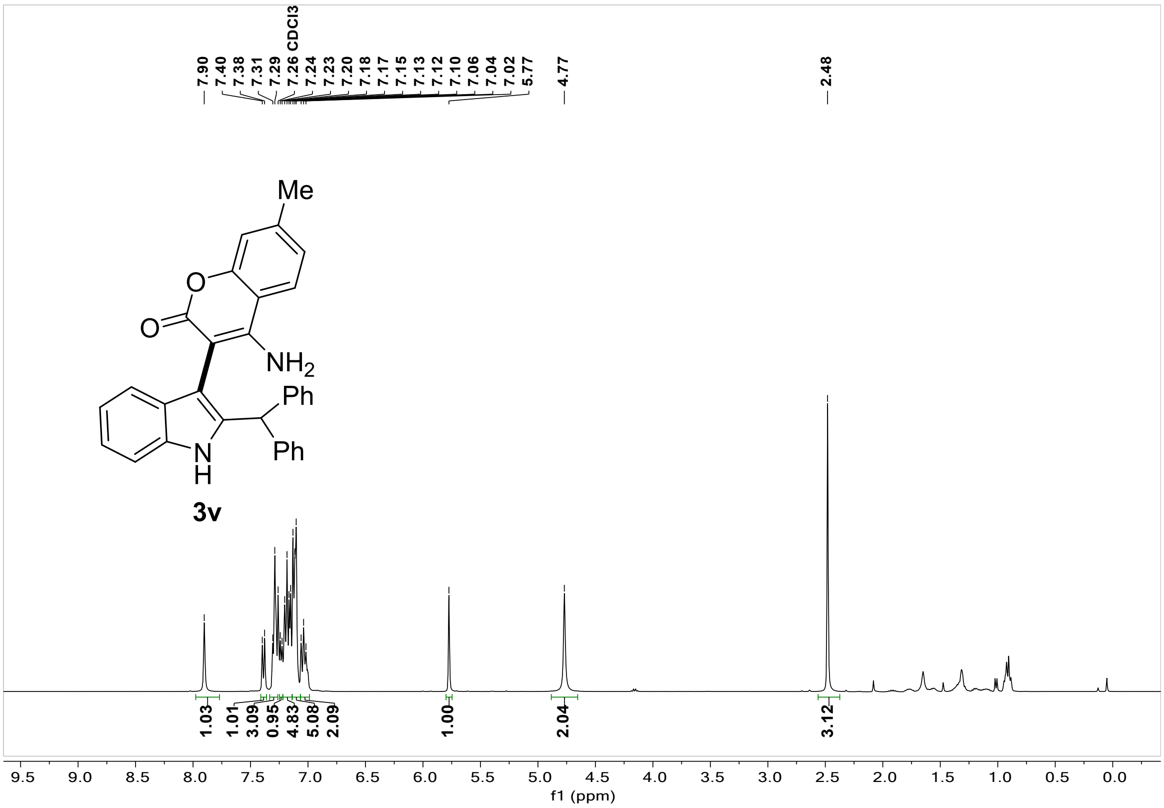
7.90
7.40
7.38
7.31
7.29
7.26 CDCl3
7.24
7.23
7.20
7.18
7.17
7.15
7.13
7.12
7.10
7.06
7.04
7.02
5.77
4.77
2.48
Me
O
O
NH2
Ph
Ph
N
H
3v
1.03
1.01
3.09
0.95
4.83
5.08
2.09
1.00
2.04
3.12
9.5 9.0 8.5 8.0 7.5 7.0 6.5 6.0 5.5 5.0 4.5 4.0 3.5 3.0 2.5 2.0 1.5 1.0 0.5 0.0
f1 (ppm)

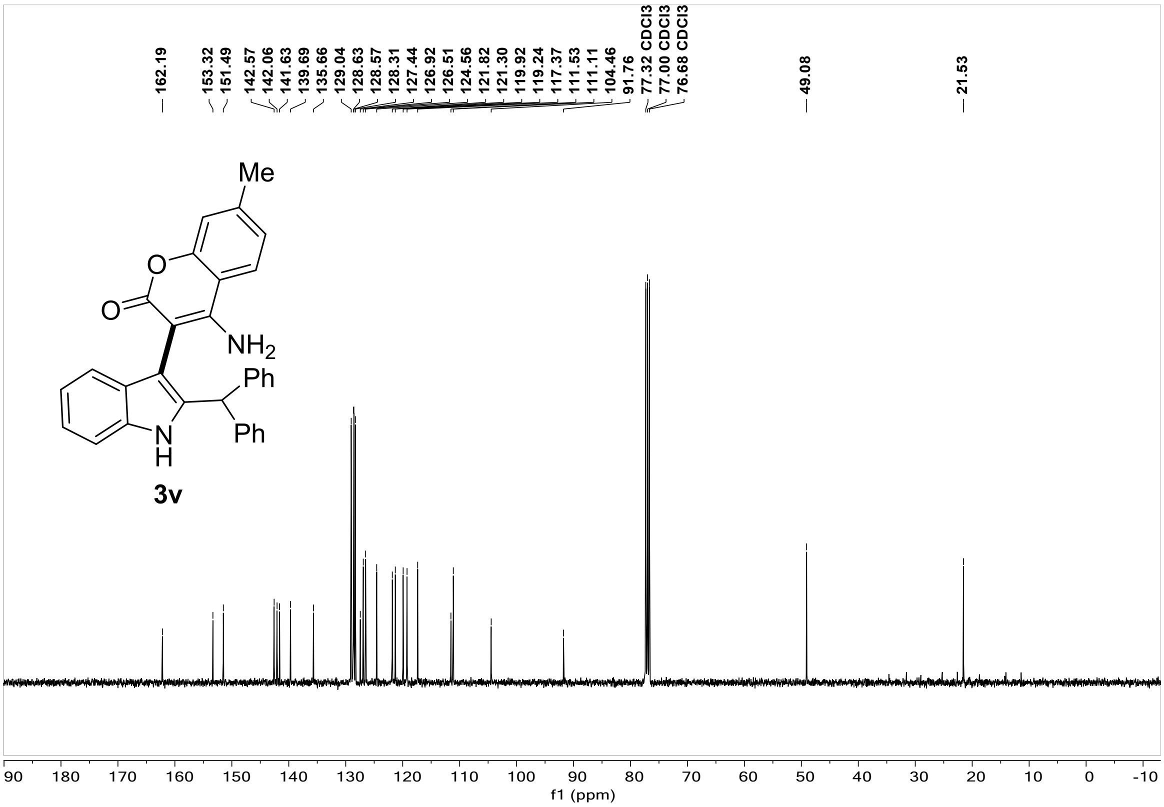

162.19
153.32
151.49
142.57
142.06
141.63
139.69
135.66
129.04
128.63
128.57
128.31
127.44
126.92
126.51
124.56
121.82
121.30
119.92
119.24
117.37
111.53
111.11
104.46
91.76
77.32 CDCl3
77.00 CDCl3
76.68 CDCl3
49.08
21.53
Me
O
O
NH2
Ph
Ph
N
H
3v
f1 (ppm)

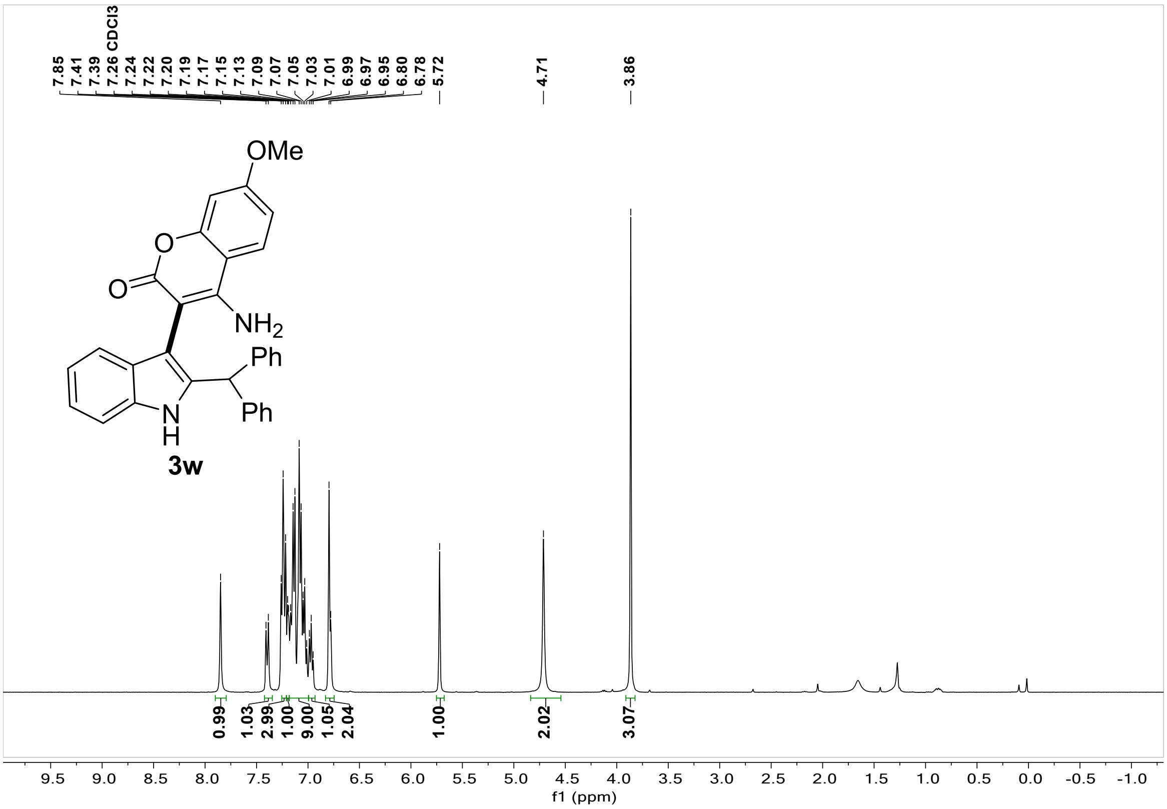

7.85
7.41
7.39
7.26 CDCl3
7.24
7.22
7.20
7.19
7.17
7.15
7.13
7.09
7.07
7.05
7.03
7.01
6.99
6.97
6.95
6.80
6.78
5.72
4.71
3.86
OMe
O
O
NH2
Ph
Ph
N
H
3w
0.99
1.03
2.99
1.00
9.00
1.05
2.04
1.00
2.02
3.07
f1 (ppm)

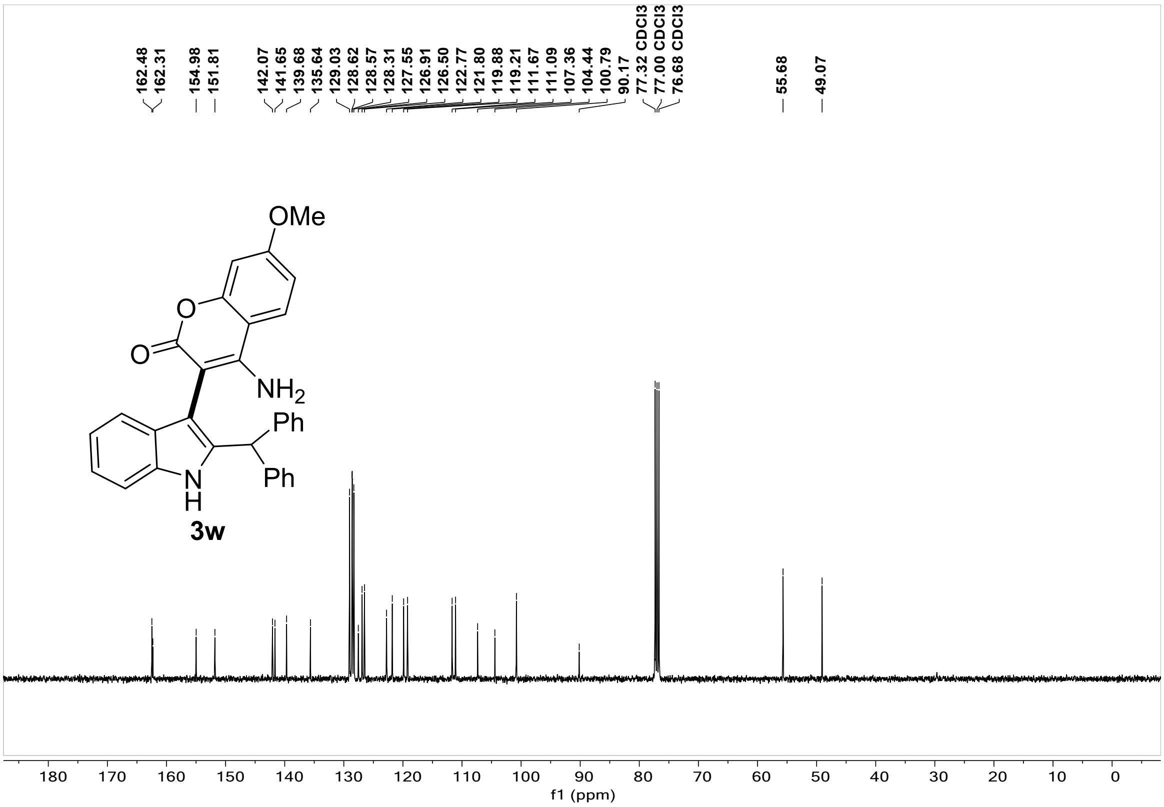
162.48
162.31
154.98
151.81
142.07
141.65
139.68
135.64
129.03
128.62
128.57
128.31
127.55
126.91
126.50
122.77
121.80
119.88
119.21
111.67
111.09
107.36
104.44
100.79
90.17
77.32 CDCl3
77.00 CDCl3
76.68 CDCl3
55.68
49.07
OMe
O
O
NH2
Ph
Ph
N
H
3w
f1 (ppm)

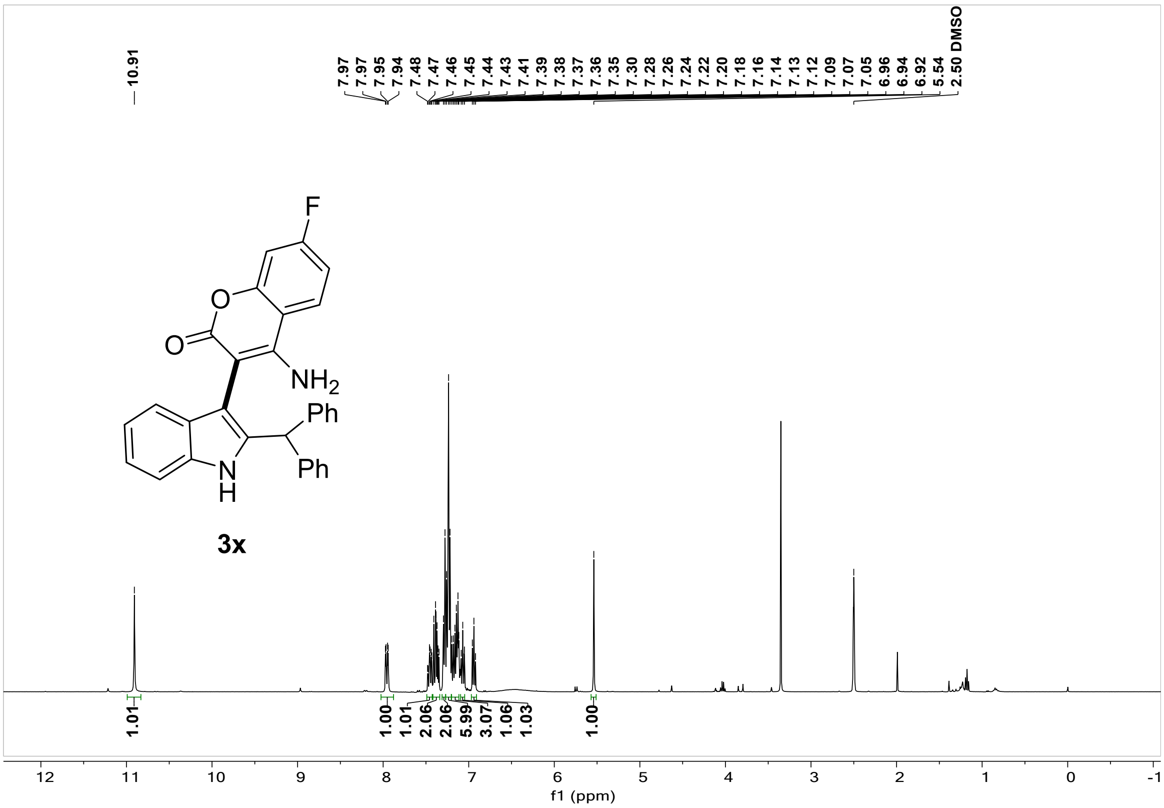
10.91
7.97
7.97
7.95
7.94
7.48
7.47
7.46
7.45
7.44
7.43
7.41
7.39
7.38
7.37
7.36
7.35
7.30
7.28
7.26
7.24
7.22
7.20
7.18
7.16
7.14
7.13
7.12
7.09
7.07
7.05
6.96
6.94
6.92
5.54
2.50 DMSO
F
O
O
NH2
Ph
Ph
N
H
3x
1.01
1.00
1.01
2.06
2.06
5.99
3.07
1.06
1.03
1.00
f1 (ppm)

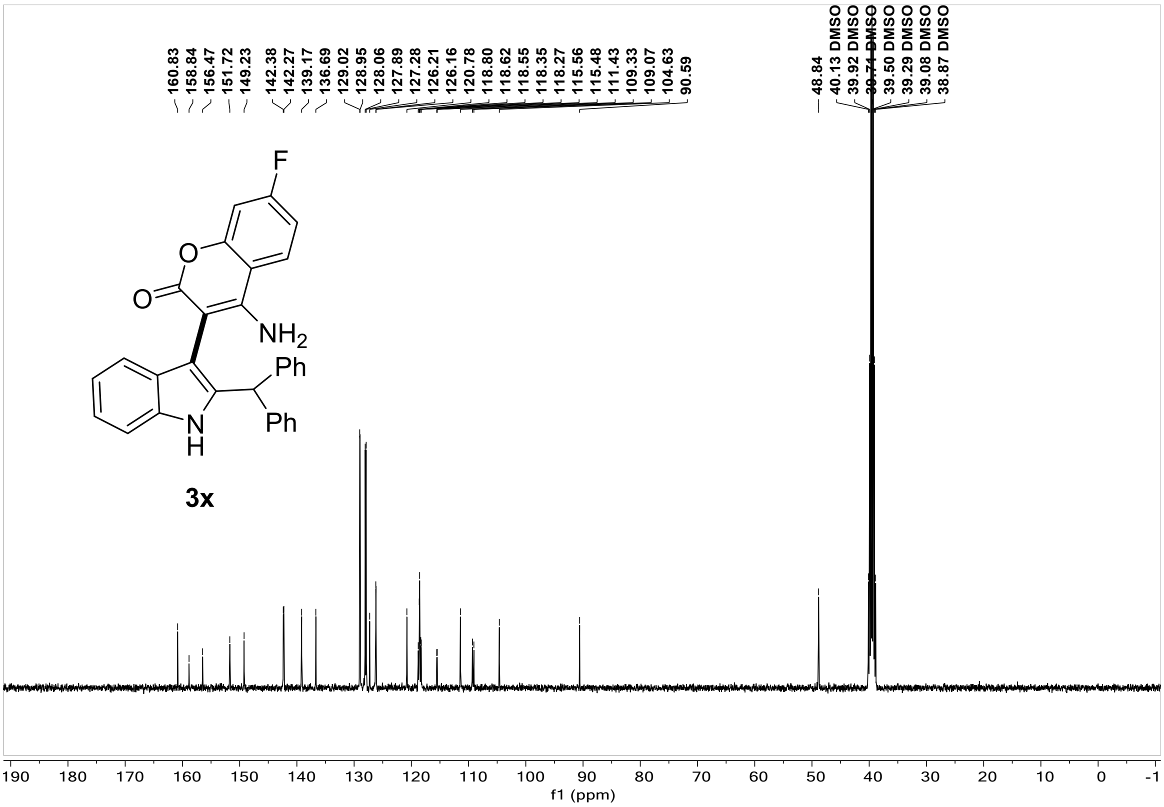
160.83
158.84
156.47
151.72
149.23
142.38
142.27
139.17
136.69
129.02
128.95
128.06
127.89
127.28
126.21
126.16
120.78
118.80
118.62
118.55
118.35
118.27
115.56
115.48
111.43
109.33
109.07
104.63
90.59
48.84
40.13 DMSO
39.92 DMSO
39.71 DMSO
39.50 DMSO
39.29 DMSO
39.08 DMSO
38.87 DMSO
F
O
O
NH2
Ph
Ph
N
H
3x
f1 (ppm)

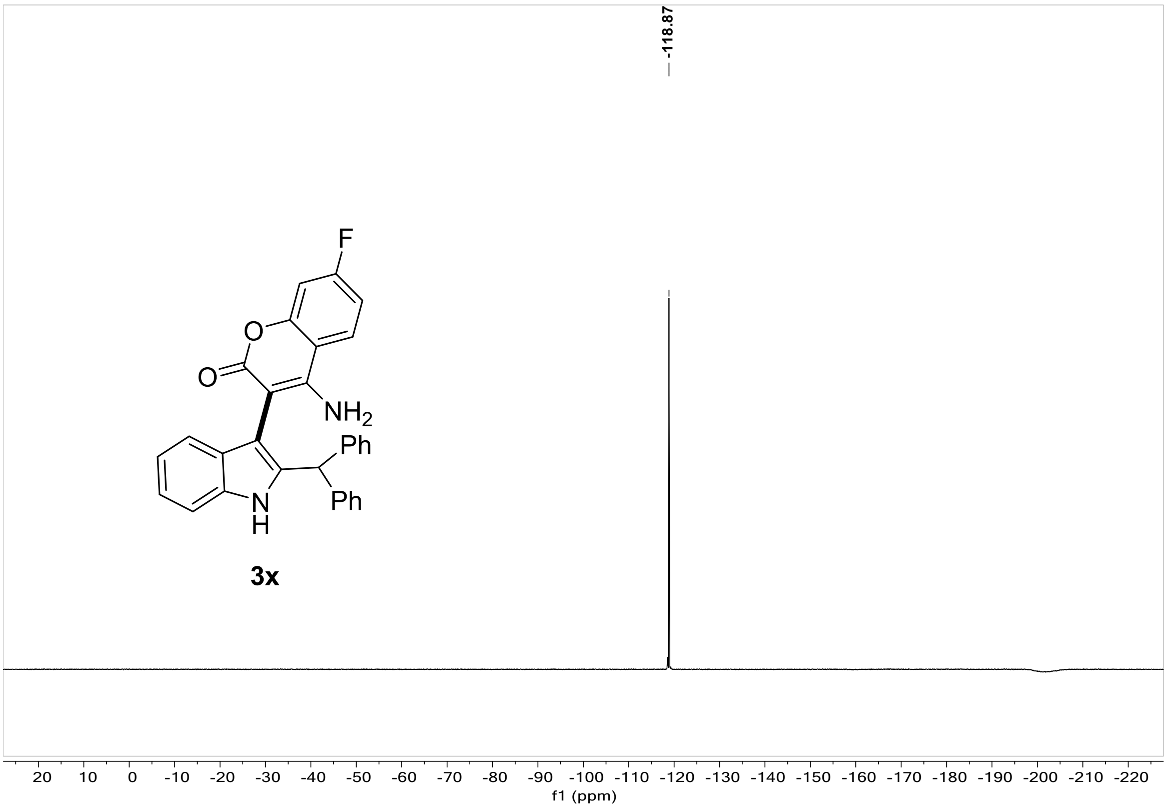
-118.87
F
O
O
NH2
Ph
Ph
N
H
3x
f1 (ppm)

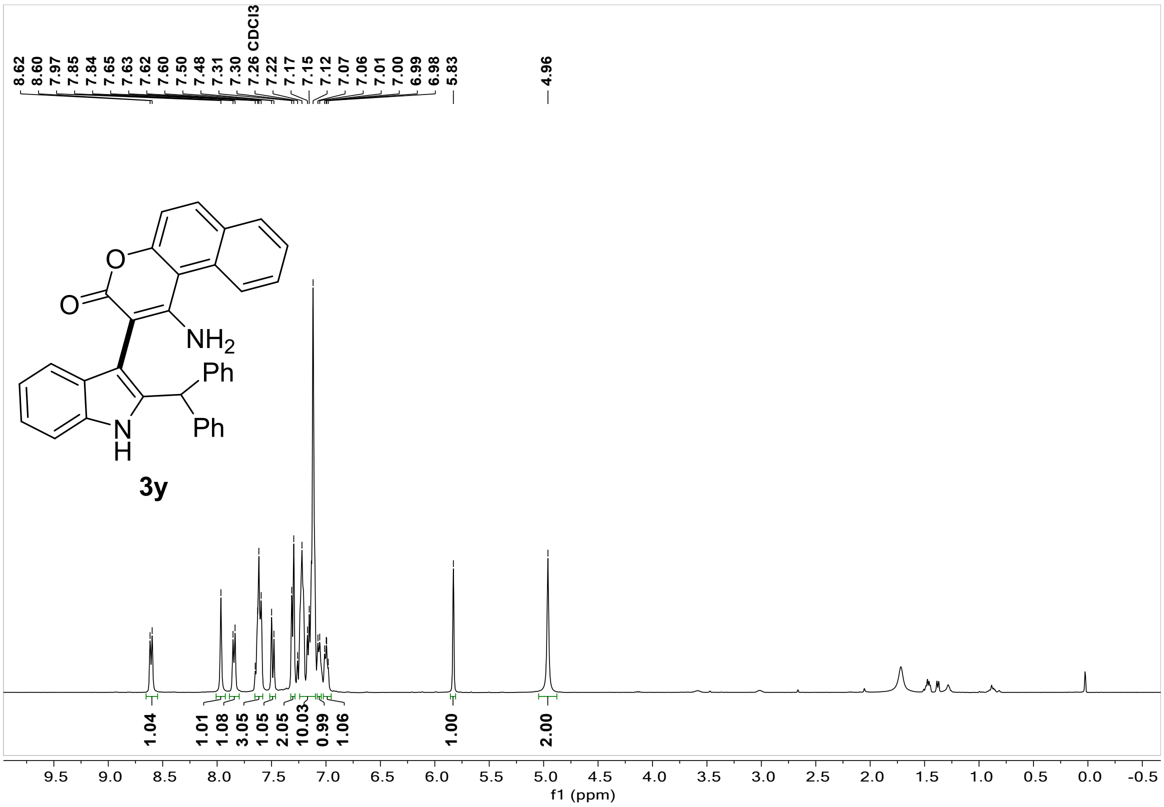
8.62 8.60 7.97 7.85 7.84 7.65 7.63 7.62 7.60 7.50 7.48 7.31 7.30 7.26 CDCl3 7.22 7.17 7.15 7.12 7.07 7.06 7.01 7.00 6.99 6.98 5.83 4.96
O
O
NH2
Ph
Ph
N
H
3y
1.04 1.01 1.08 3.05 1.05 2.05 10.03 0.99 1.06 1.00 2.00
9.5 9.0 8.5 8.0 7.5 7.0 6.5 6.0 5.5 5.0 4.5 4.0 3.5 3.0 2.5 2.0 1.5 1.0 0.5 0.0 -0.5
f1 (ppm)

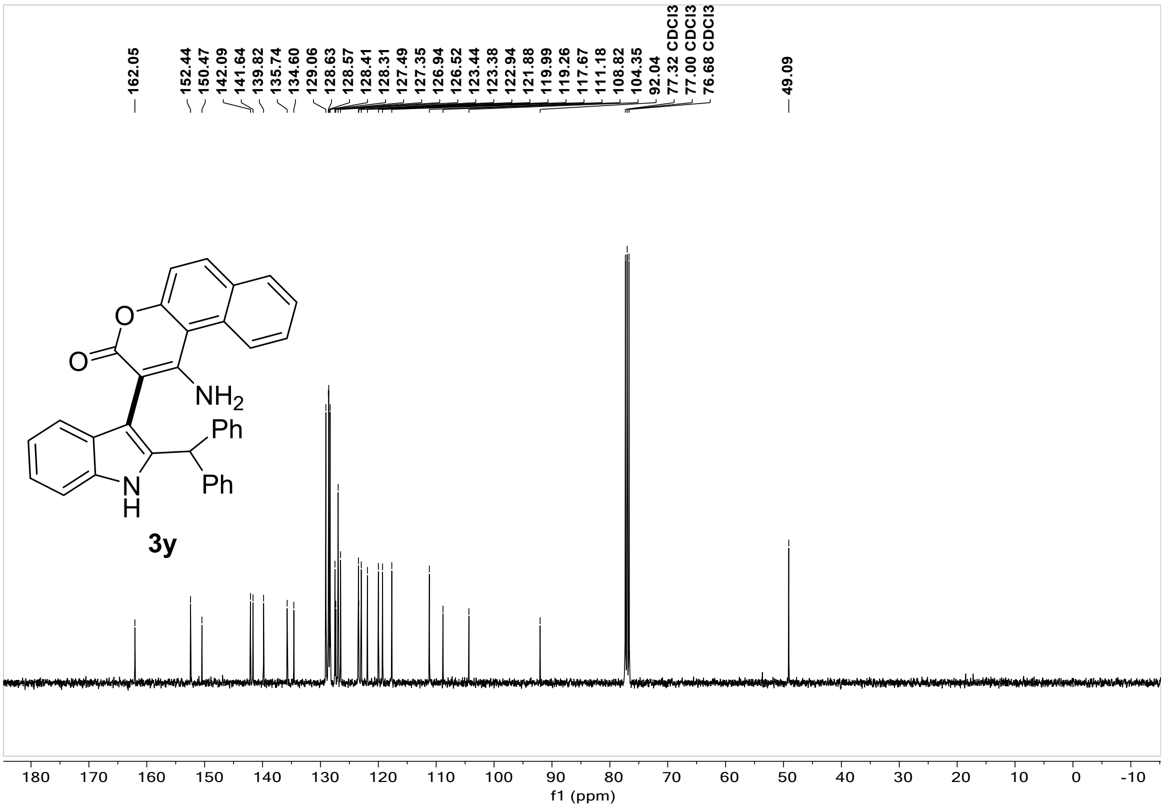
162.05 152.44 150.47 142.09 141.64 139.82 135.74 134.60 129.06 128.63 128.57 128.41 128.31 127.49 127.35 126.94 126.52 123.44 123.38 122.94 121.88 119.99 119.26 117.67 111.18 108.82 104.35 92.04 77.32 CDCl3 77.00 CDCl3 76.68 CDCl3 49.09
O
O
NH2
Ph
Ph
N
H
3y
180 170 160 150 140 130 120 110 100 90 80 70 60 50 40 30 20 10 0 -10
f1 (ppm)

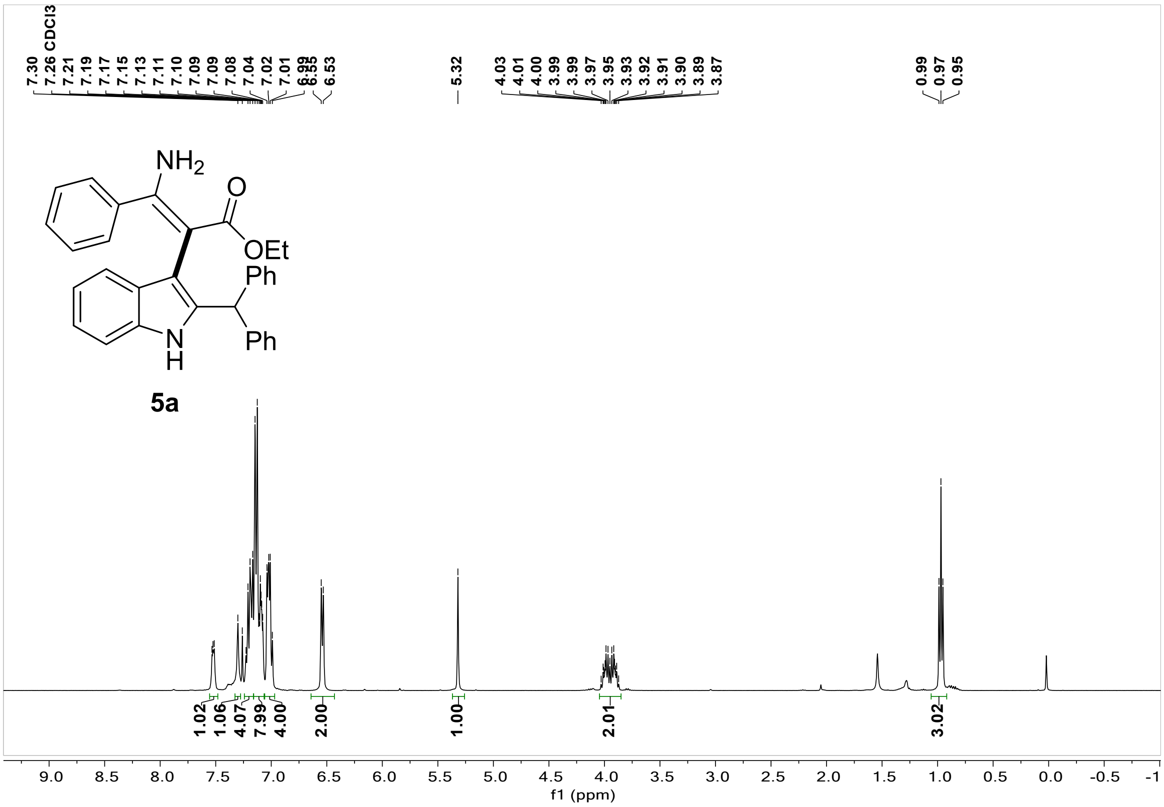
7.30
7.26 CDCl3
7.21
7.19
7.17
7.15
7.13
7.11
7.10
7.09
7.08
7.04
7.02
7.01
6.55
6.53
5.32
4.03
4.01
4.00
3.99
3.99
3.97
3.95
3.93
3.92
3.91
3.90
3.89
3.87
0.99
0.97
0.95
NH2
O
OEt
Ph
Ph
N
H
5a
1.02
1.06
4.07
7.99
4.00
2.00
1.00
2.01
3.02
f1 (ppm)

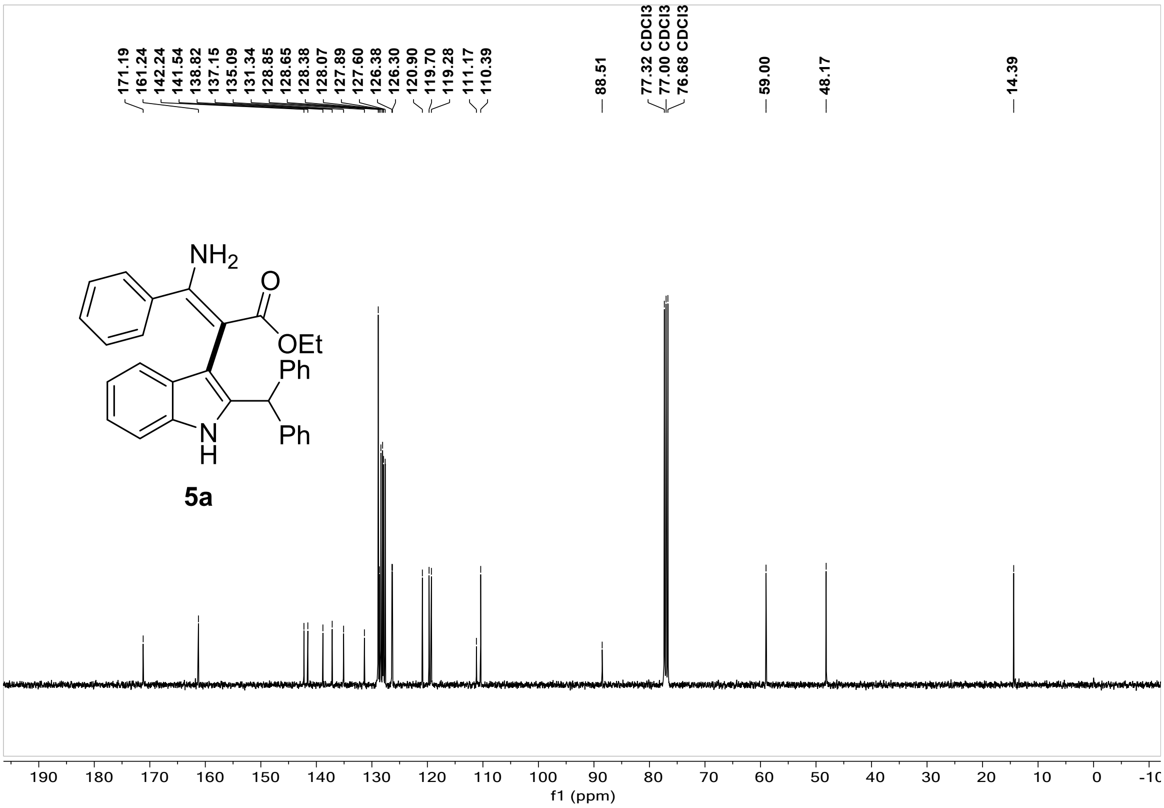
171.19
161.24
142.24
141.54
138.82
137.15
135.09
131.34
128.85
128.65
128.38
128.07
127.89
127.60
126.38
126.30
120.90
119.70
119.28
111.17
110.39
88.51
77.32 CDCl3
77.00 CDCl3
76.68 CDCl3
59.00
48.17
14.39
NH2
O
OEt
Ph
Ph
N
H
5a
f1 (ppm)

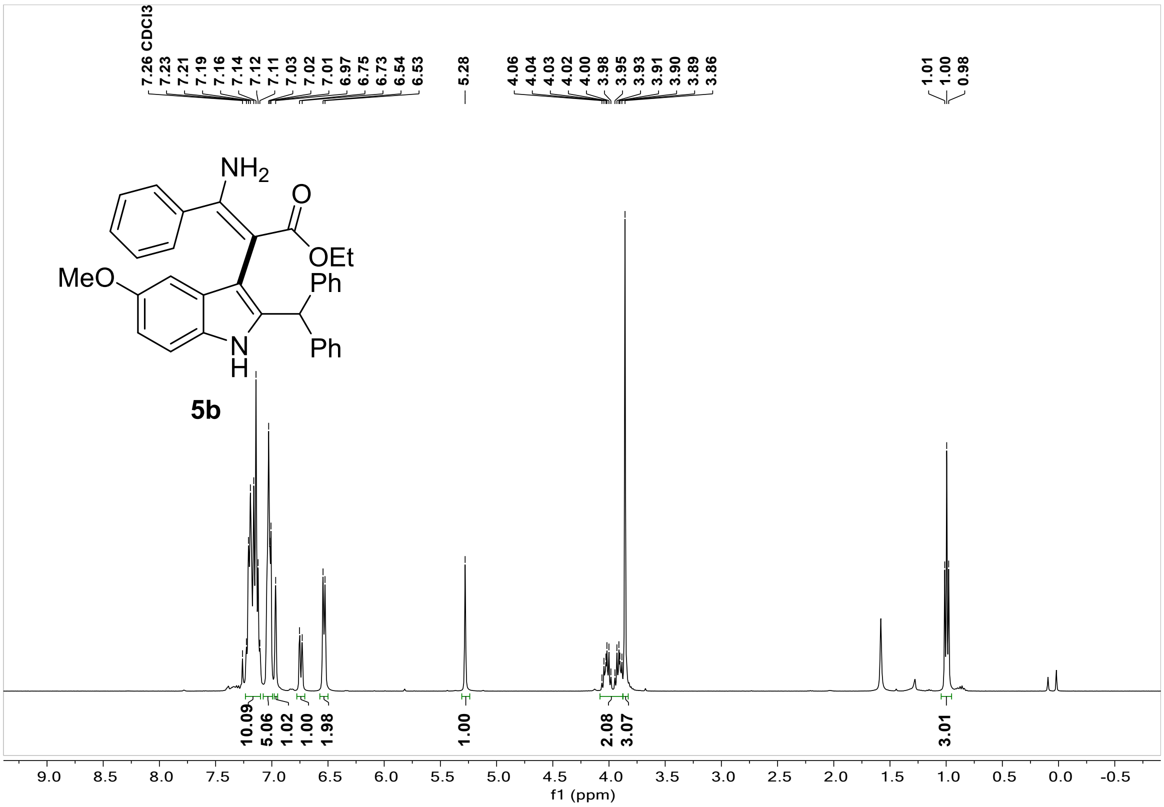
7.26 CDCl3
7.23
7.21
7.19
7.16
7.14
7.12
7.11
7.03
7.02
7.01
6.97
6.75
6.73
6.54
6.53
5.28
4.06
4.04
4.03
4.02
4.00
3.98
3.95
3.93
3.91
3.90
3.89
3.86
1.01
1.00
0.98
NH2
O
OEt
MeO
Ph
Ph
N
H
5b
10.09
5.06
1.02
1.00
1.98
1.00
2.08
3.07
3.01
9.0 8.5 8.0 7.5 7.0 6.5 6.0 5.5 5.0 4.5 4.0 3.5 3.0 2.5 2.0 1.5 1.0 0.5 0.0 -0.5
f1 (ppm)

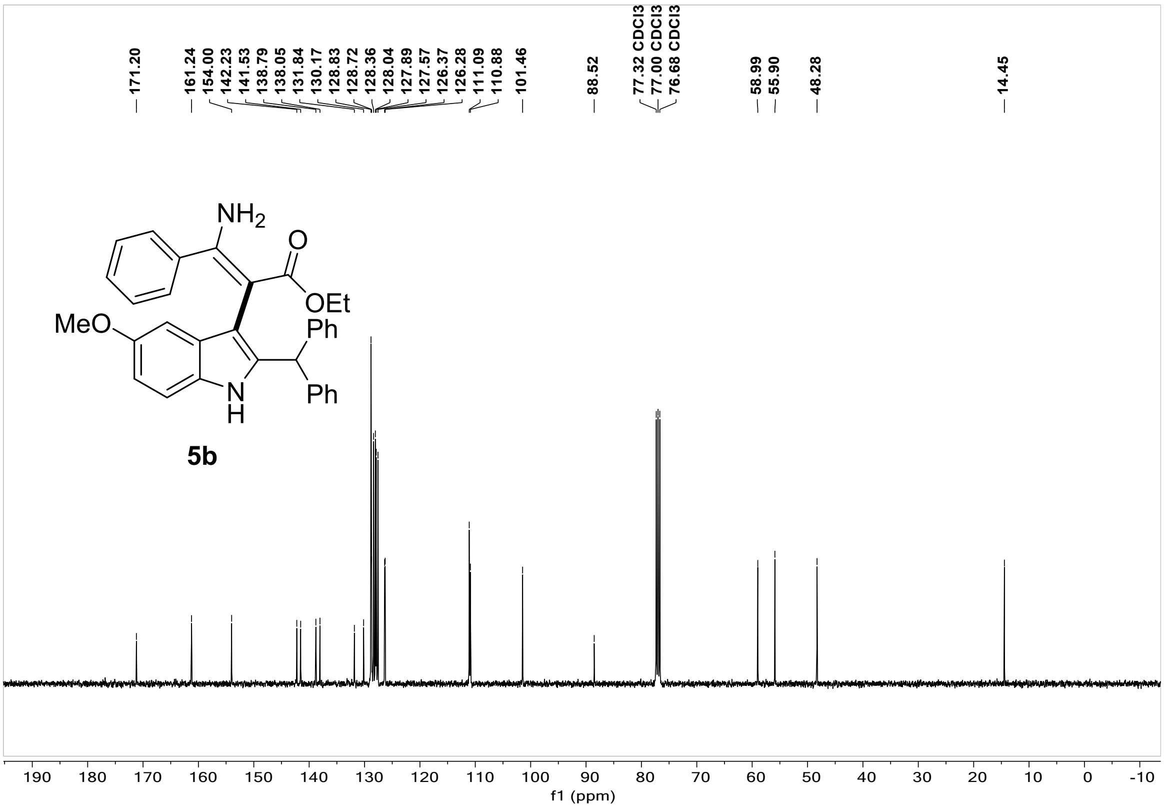
171.20
161.24
154.00
142.23
141.53
138.79
138.05
131.84
130.17
128.83
128.72
128.36
128.04
127.89
127.57
126.37
126.28
111.09
110.88
101.46
88.52
77.32 CDCl3
77.00 CDCl3
76.68 CDCl3
58.99
55.90
48.28
14.45
NH2
O
OEt
MeO
Ph
Ph
N
H
5b
190 180 170 160 150 140 130 120 110 100 90 80 70 60 50 40 30 20 10 0 -10
f1 (ppm)

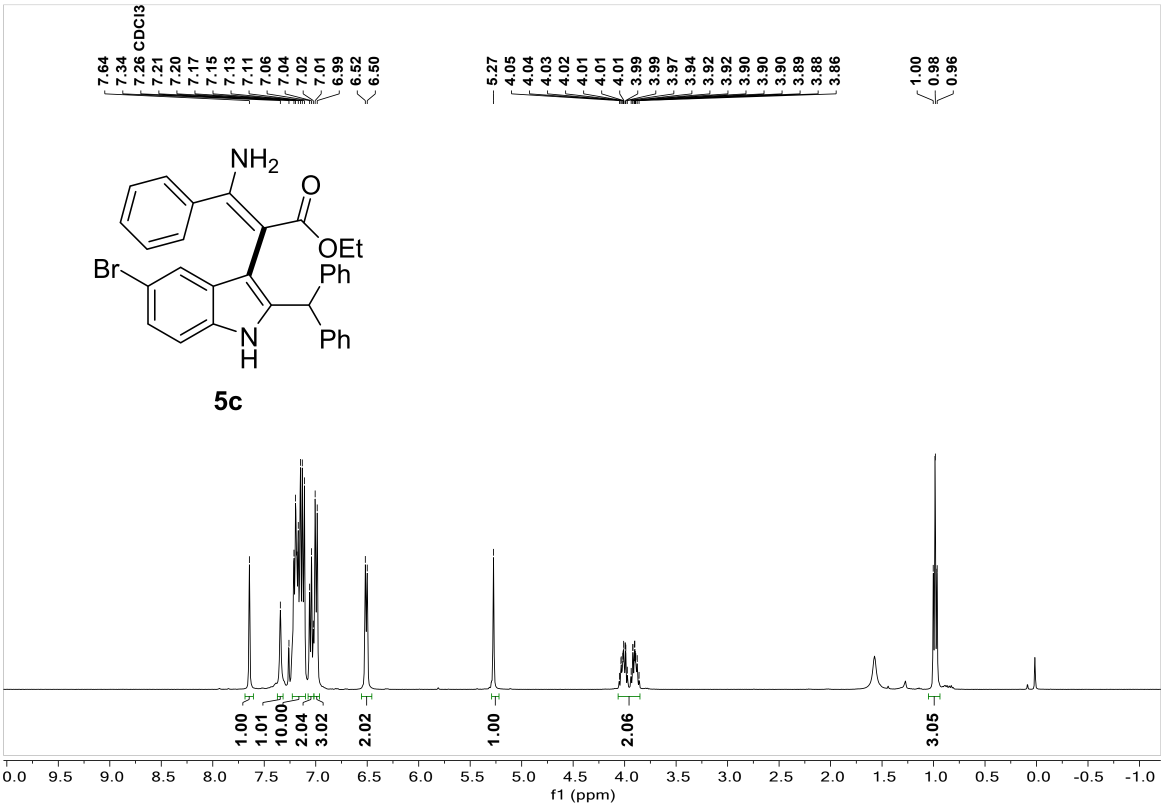
7.64
7.34
7.26 CDCl3
7.21
7.20
7.17
7.15
7.13
7.11
7.06
7.04
7.02
7.01
6.99
6.52
6.50
5.27
4.05
4.04
4.03
4.02
4.01
4.01
4.01
3.99
3.99
3.97
3.94
3.92
3.92
3.90
3.90
3.89
3.88
3.86
1.00
0.98
0.96
NH2
O
OEt
Br
Ph
Ph
N
H
5c
1.00
1.01
10.00
2.04
3.02
2.02
1.00
2.06
3.05
f1 (ppm)

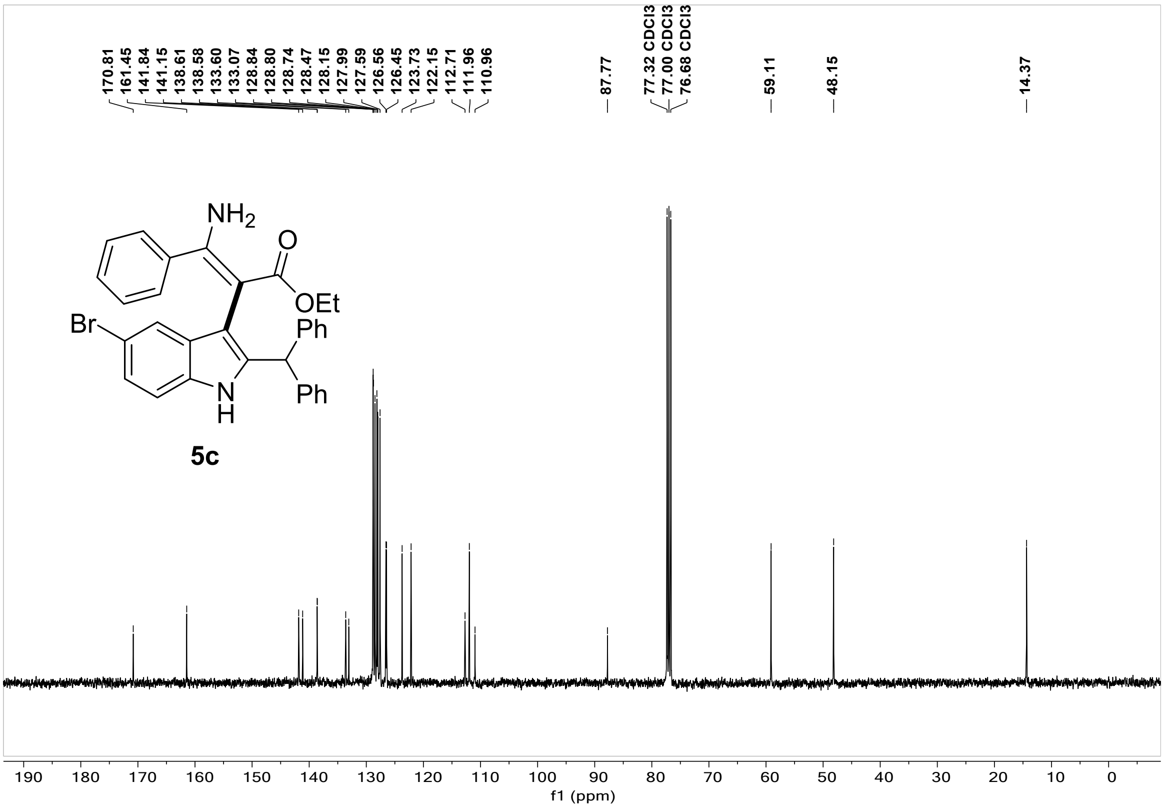
170.81
161.45
141.84
141.15
138.61
138.58
133.60
133.07
128.84
128.80
128.74
128.47
128.15
127.99
127.59
126.56
126.45
123.73
122.15
112.71
111.96
110.96
87.77
77.32 CDCl3
77.00 CDCl3
76.68 CDCl3
59.11
48.15
14.37
NH2
O
OEt
Br
Ph
Ph
N
H
5c
f1 (ppm)

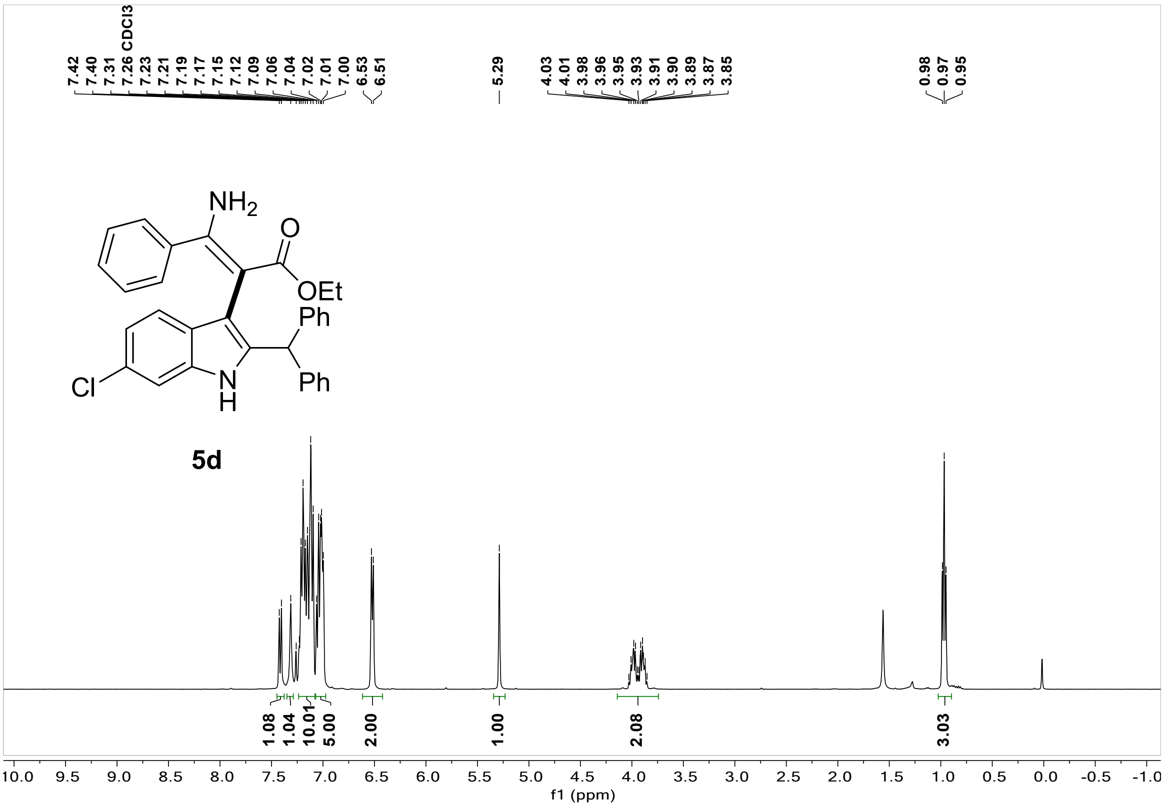
7.42
7.40
7.31
7.26 CDCl3
7.23
7.21
7.19
7.17
7.15
7.12
7.09
7.06
7.04
7.02
7.01
7.00
6.53
6.51
5.29
4.03
4.01
3.98
3.96
3.95
3.93
3.91
3.90
3.89
3.87
3.85
0.98
0.97
0.95
NH2
O
OEt
Ph
Ph
Cl
N
H
5d
1.08
1.04
10.01
5.00
2.00
1.00
2.08
3.03
10.0 9.5 9.0 8.5 8.0 7.5 7.0 6.5 6.0 5.5 5.0 4.5 4.0 3.5 3.0 2.5 2.0 1.5 1.0 0.5 0.0 -0.5 -1.0
f1 (ppm)

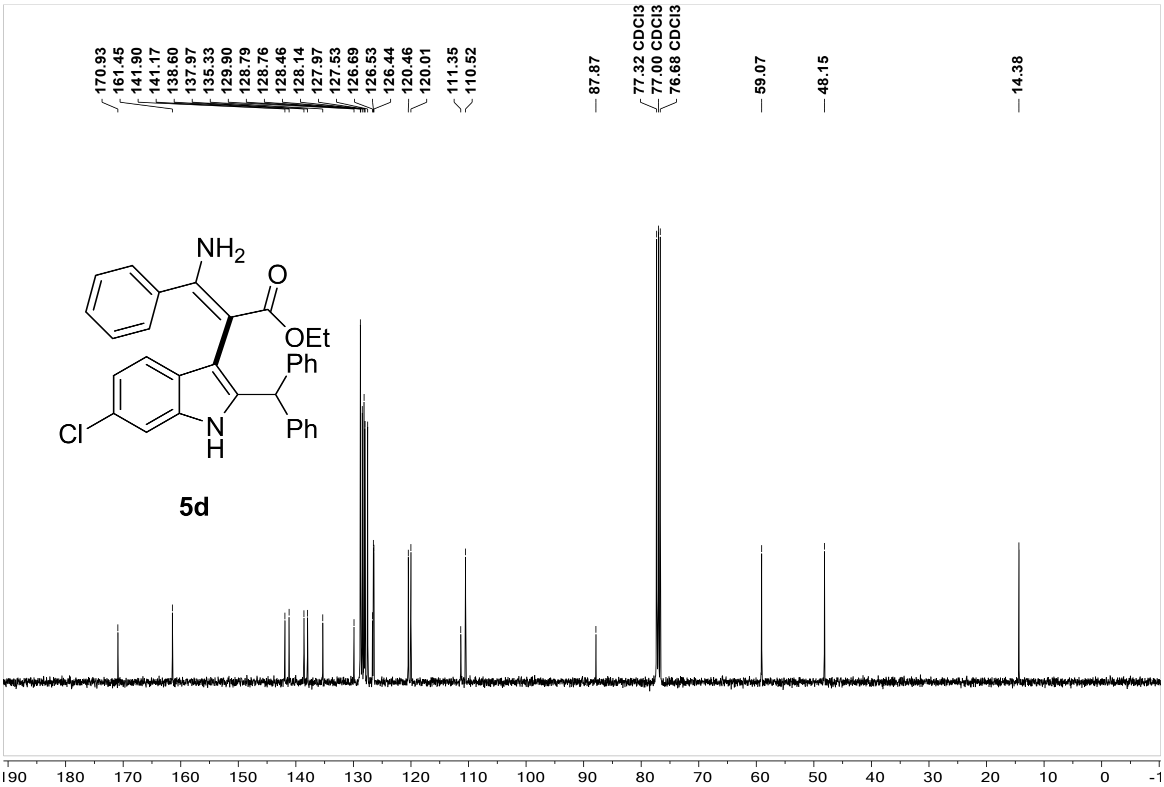
170.93
161.45
141.90
141.17
138.60
137.97
135.33
129.90
128.79
128.76
128.46
128.14
127.97
127.53
126.69
126.53
126.44
120.46
120.01
111.35
110.52
87.87
77.32 CDCl3
77.00 CDCl3
76.68 CDCl3
59.07
48.15
14.38
NH2
O
OEt
Ph
Ph
Cl
N
H
5d
190 180 170 160 150 140 130 120 110 100 90 80 70 60 50 40 30 20 10 0 -1
f1 (ppm)

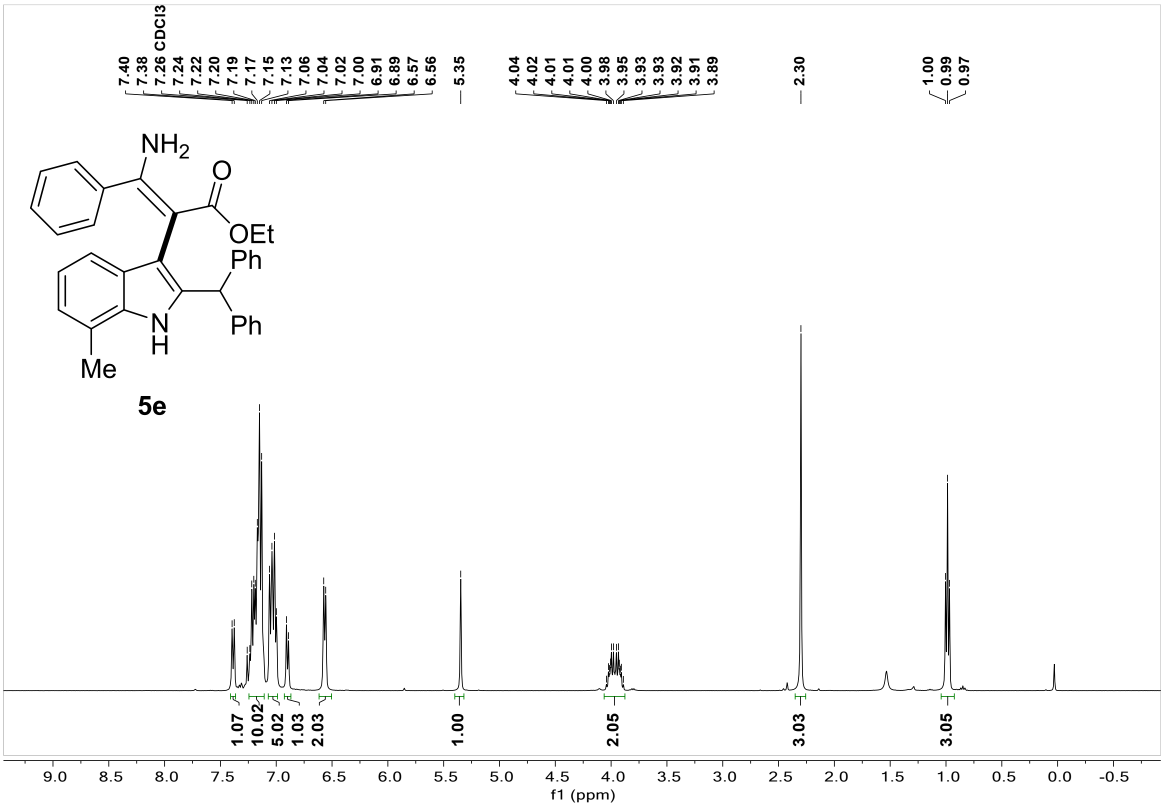
7.40
7.38
7.26 CDCl3
7.24
7.22
7.20
7.19
7.17
7.15
7.13
7.06
7.04
7.02
7.00
6.91
6.89
6.57
6.56
5.35
4.04
4.02
4.01
4.01
4.00
3.98
3.95
3.93
3.93
3.92
3.91
3.89
2.30
1.00
0.99
0.97
NH2
O
OEt
Ph
Ph
N
H
Me
5e
1.07
10.02
5.02
1.03
2.03
1.00
2.05
3.03
3.05
9.0 8.5 8.0 7.5 7.0 6.5 6.0 5.5 5.0 4.5 4.0 3.5 3.0 2.5 2.0 1.5 1.0 0.5 0.0 -0.5
f1 (ppm)

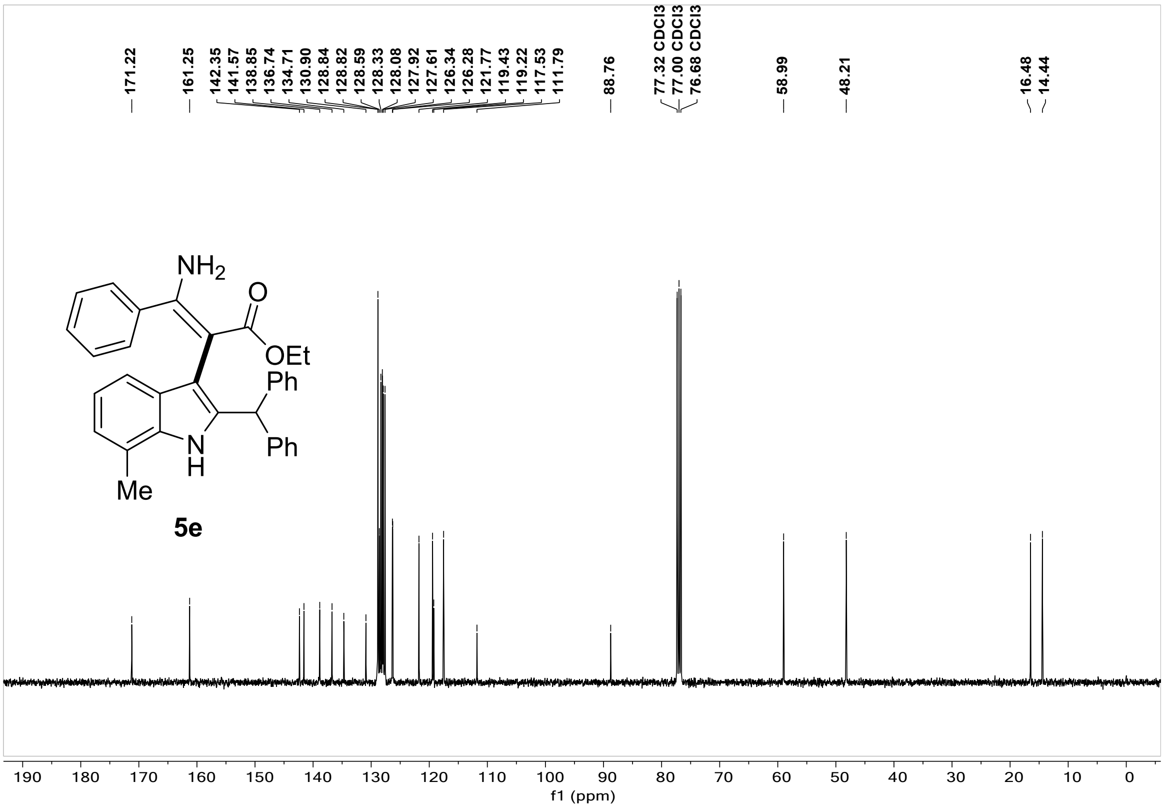
171.22
161.25
142.35
141.57
138.85
136.74
134.71
130.90
128.84
128.82
128.59
128.33
128.08
127.92
127.61
126.34
126.28
121.77
119.43
119.22
117.53
111.79
88.76
77.32 CDCl3
77.00 CDCl3
76.68 CDCl3
58.99
48.21
16.48
14.44
NH2
O
OEt
Ph
Ph
N
H
Me
5e
190 180 170 160 150 140 130 120 110 100 90 80 70 60 50 40 30 20 10 0
f1 (ppm)

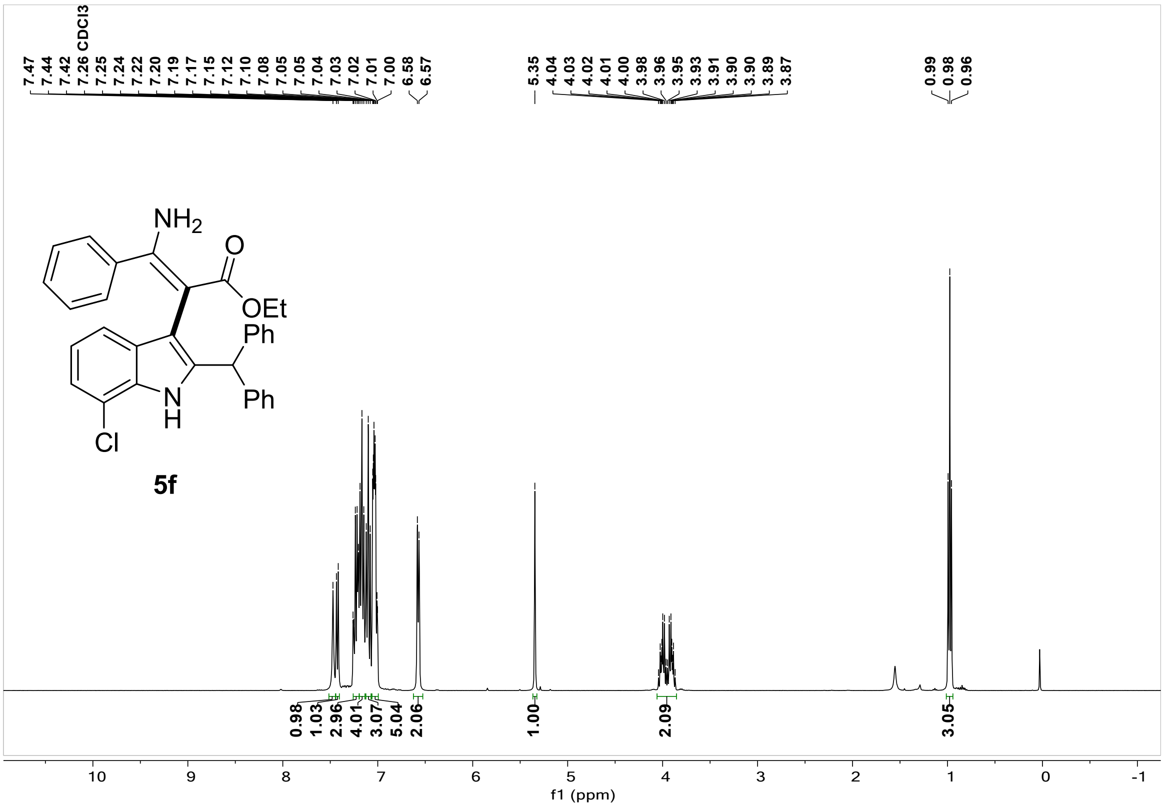
7.47
7.44
7.42
7.26 CDCl3
7.25
7.24
7.22
7.20
7.19
7.17
7.15
7.12
7.10
7.08
7.05
7.05
7.04
7.03
7.02
7.01
7.00
6.58
6.57
5.35
4.04
4.03
4.02
4.01
4.00
3.98
3.96
3.95
3.93
3.91
3.90
3.90
3.89
3.87
0.99
0.98
0.96
NH2
O
OEt
Ph
Ph
N
H
Cl
5f
0.98
1.03
2.96
4.01
3.07
5.04
2.06
1.00
2.09
3.05
10 9 8 7 6 5 4 3 2 1 0 -1
f1 (ppm)

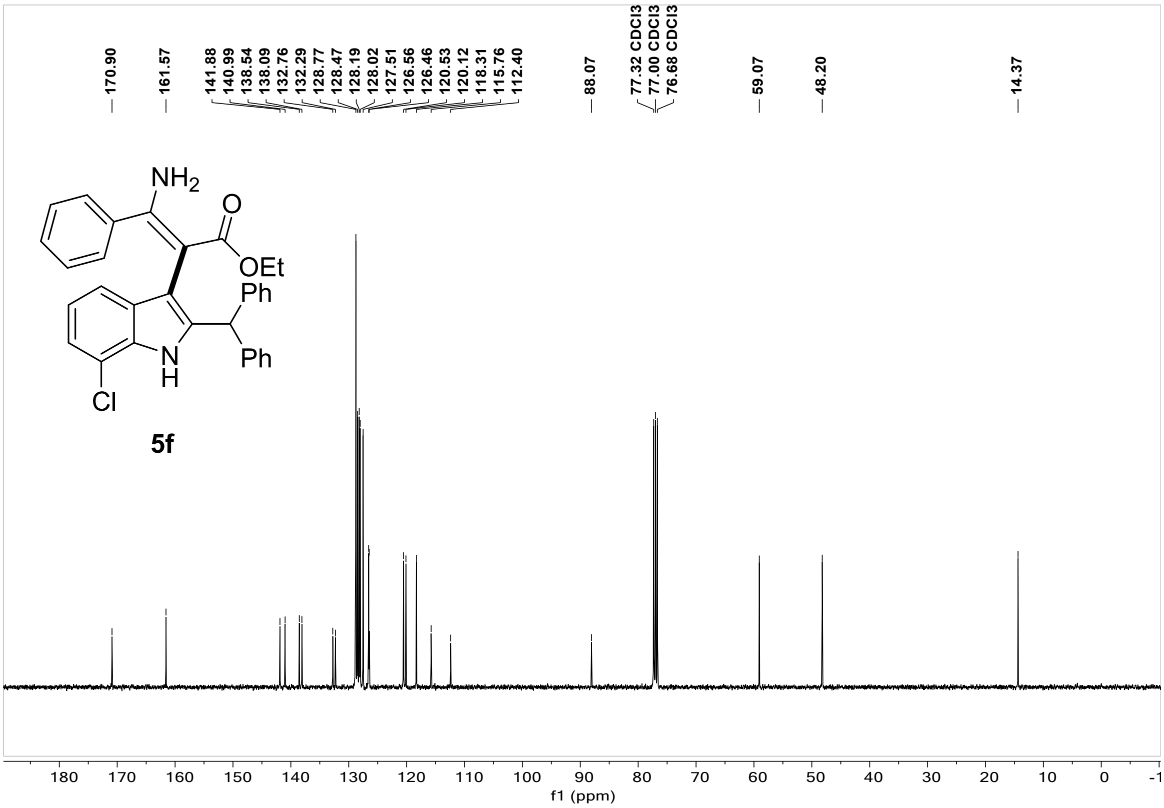
170.90
161.57
141.88
140.99
138.54
138.09
132.76
132.29
128.77
128.47
128.19
128.02
127.51
126.56
126.46
120.53
120.12
118.31
115.76
112.40
88.07
77.32 CDCl3
77.00 CDCl3
76.68 CDCl3
59.07
48.20
14.37
NH2
O
OEt
Ph
Ph
N
H
Cl
5f
180 170 160 150 140 130 120 110 100 90 80 70 60 50 40 30 20 10 0 -1
f1 (ppm)

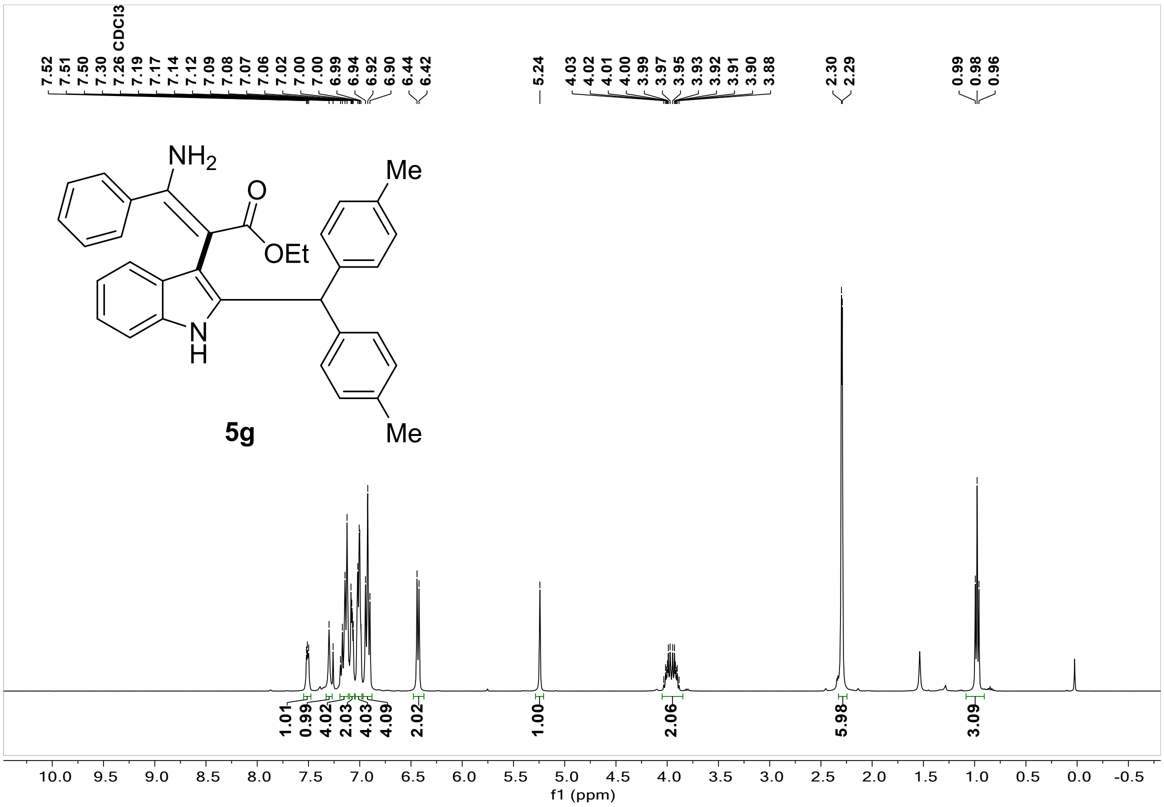
7.52
7.51
7.50
7.30
7.26 CDCl3
7.19
7.17
7.14
7.12
7.09
7.08
7.07
7.06
7.02
7.00
7.00
6.99
6.94
6.92
6.90
6.44
6.42
5.24
4.03
4.02
4.01
4.00
3.99
3.97
3.95
3.93
3.92
3.91
3.90
3.88
2.30
2.29
0.99
0.98
0.96
NH2
O
OEt
Me
N
H
Me
5g
1.01
0.99
4.02
2.03
4.03
4.09
2.02
1.00
2.06
5.98
3.09
10.0 9.5 9.0 8.5 8.0 7.5 7.0 6.5 6.0 5.5 5.0 4.5 4.0 3.5 3.0 2.5 2.0 1.5 1.0 0.5 0.0 -0.5
f1 (ppm)

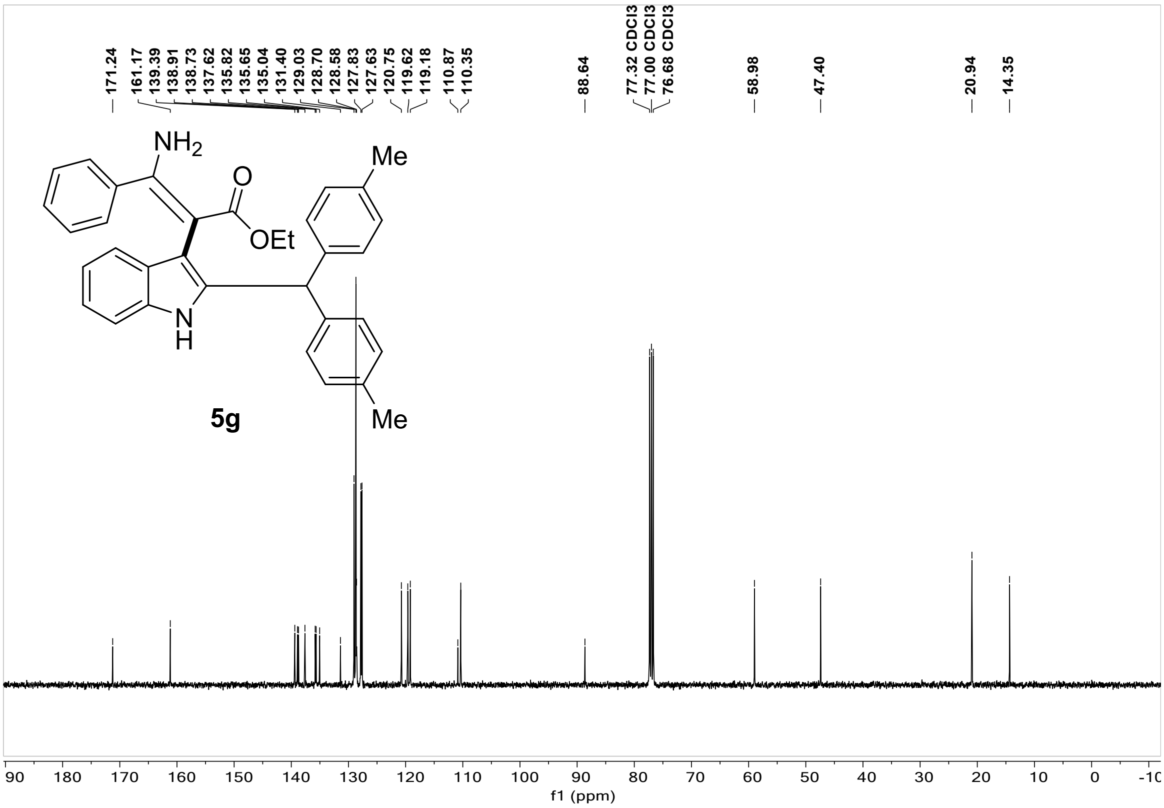
171.24
161.17
139.39
138.91
138.73
137.62
135.82
135.65
135.04
131.40
129.03
128.70
128.58
127.83
127.63
120.75
119.62
119.18
110.87
110.35
88.64
77.32 CDCl3
77.00 CDCl3
76.68 CDCl3
58.98
47.40
20.94
14.35
NH2
O
OEt
Me
N
H
Me
5g
190 180 170 160 150 140 130 120 110 100 90 80 70 60 50 40 30 20 10 0 -10
f1 (ppm)

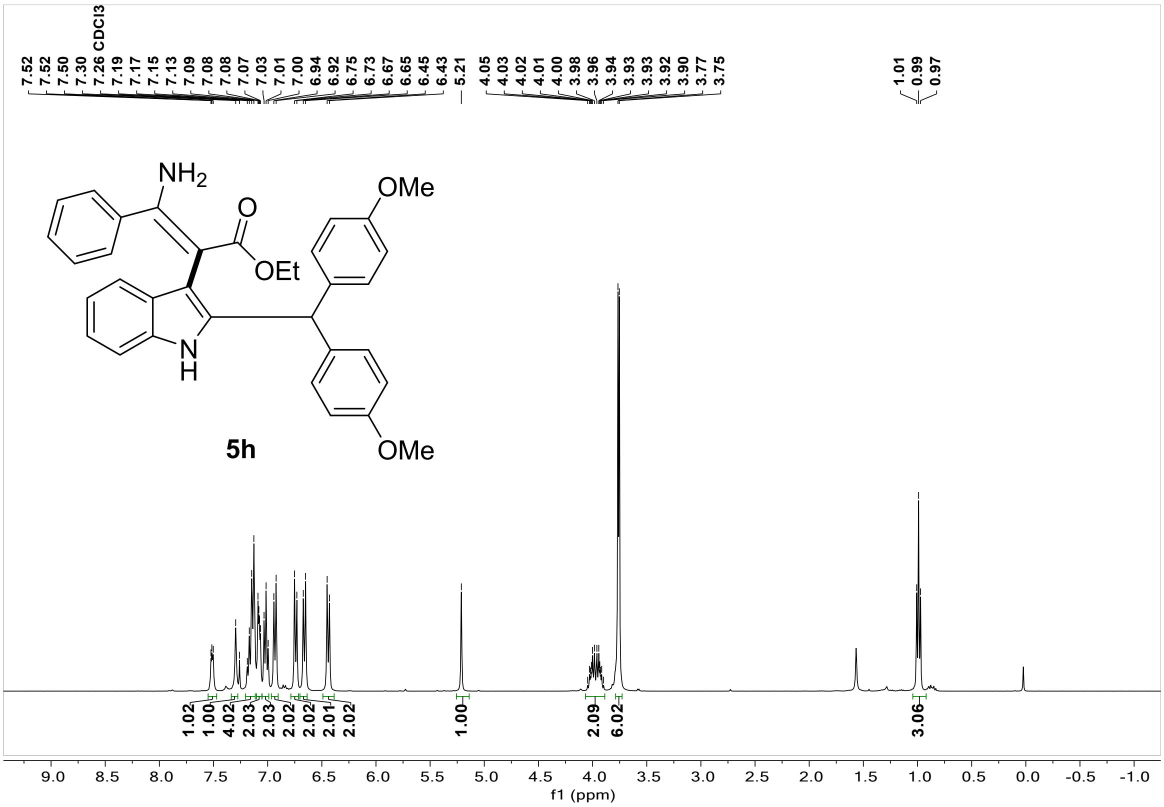
7.52 7.52 7.50 7.30 7.26 CDCl3 7.19 7.17 7.15 7.13 7.09 7.08 7.08 7.07 7.03 7.01 7.00 6.94 6.92 6.75 6.73 6.67 6.65 6.45 6.43
5.21
4.05 4.03 4.02 4.01 4.00 3.98 3.96 3.94 3.93 3.93 3.92 3.90 3.77 3.75
1.01 0.99 0.97
NH2
O
OEt
OMe
OMe
N
H
5h
1.02 1.00 4.02 2.03 2.03 2.02 2.02 2.01 2.02
1.00
2.09 6.02
3.06
9.0 8.5 8.0 7.5 7.0 6.5 6.0 5.5 5.0 4.5 4.0 3.5 3.0 2.5 2.0 1.5 1.0 0.5 0.0 -0.5 -1.0
f1 (ppm)

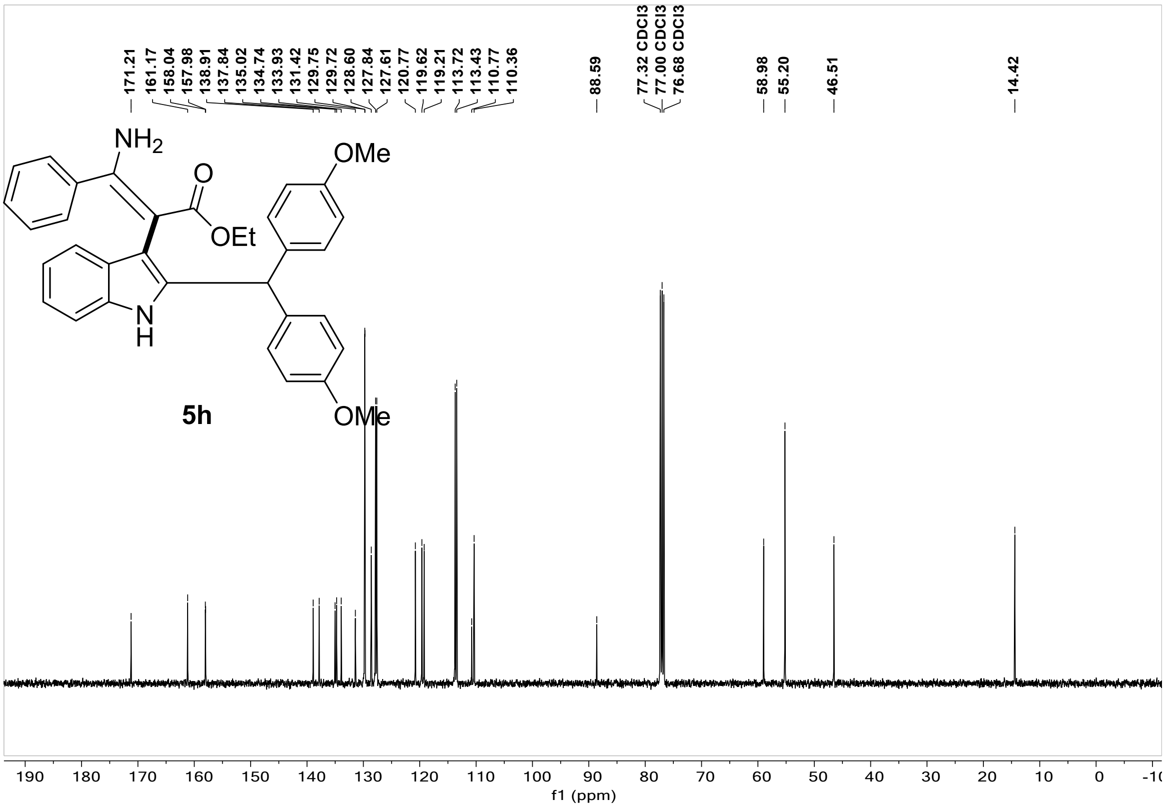
171.21
161.17 158.04 157.98 138.91 137.84 135.02 134.74 133.93 131.42 129.75 129.72 128.60 127.84 127.61 120.77 119.62 119.21 113.72 113.43 110.77 110.36
88.59
77.32 CDCl3 77.00 CDCl3 76.68 CDCl3
58.98
55.20
46.51
14.42
NH2
O
OEt
OMe
OMe
N
H
5h
190 180 170 160 150 140 130 120 110 100 90 80 70 60 50 40 30 20 10 0
f1 (ppm)

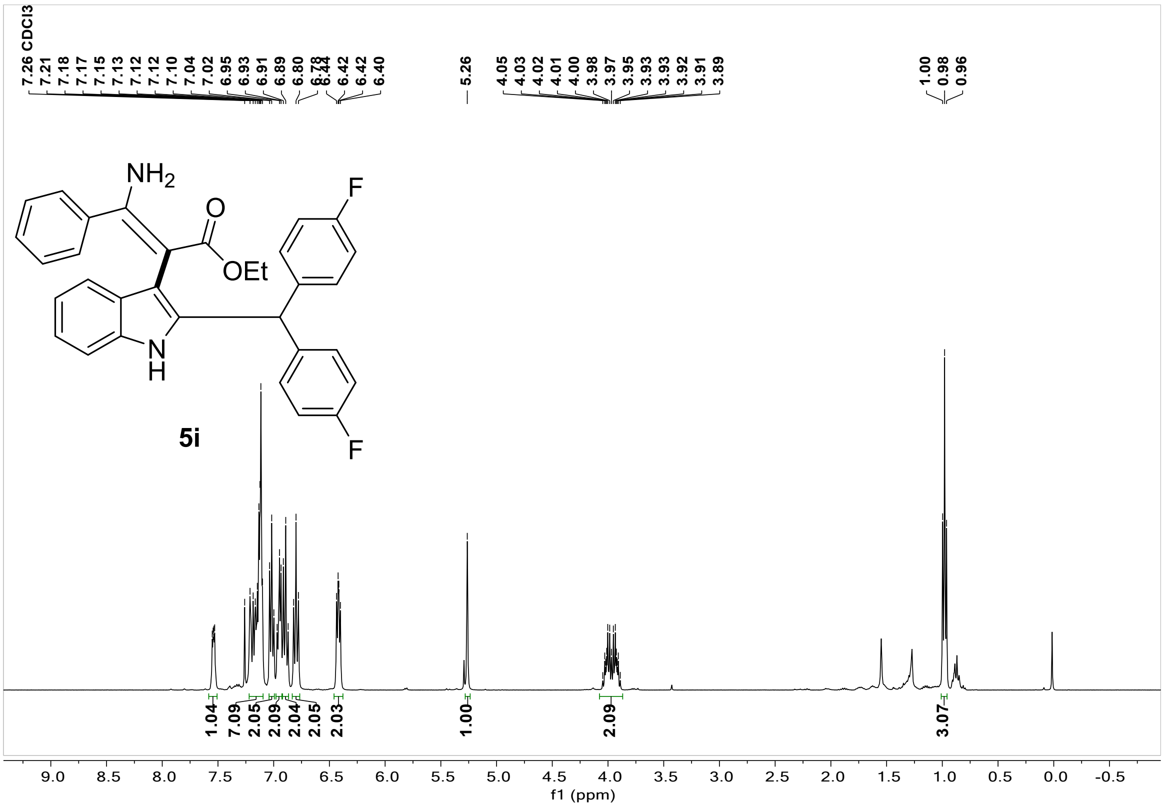
7.26 CDCl3
7.21
7.18
7.17
7.15
7.13
7.12
7.12
7.10
7.04
7.02
6.95
6.93
6.91
6.89
6.80
6.78
6.44
6.42
6.42
6.40
5.26
4.05
4.03
4.02
4.01
4.00
3.98
3.97
3.95
3.93
3.93
3.92
3.91
3.89
1.00
0.98
0.96
NH2
O
OEt
F
N
H
F
5i
1.04
7.09
2.05
2.09
2.04
2.05
2.03
1.00
2.09
3.07
f1 (ppm)

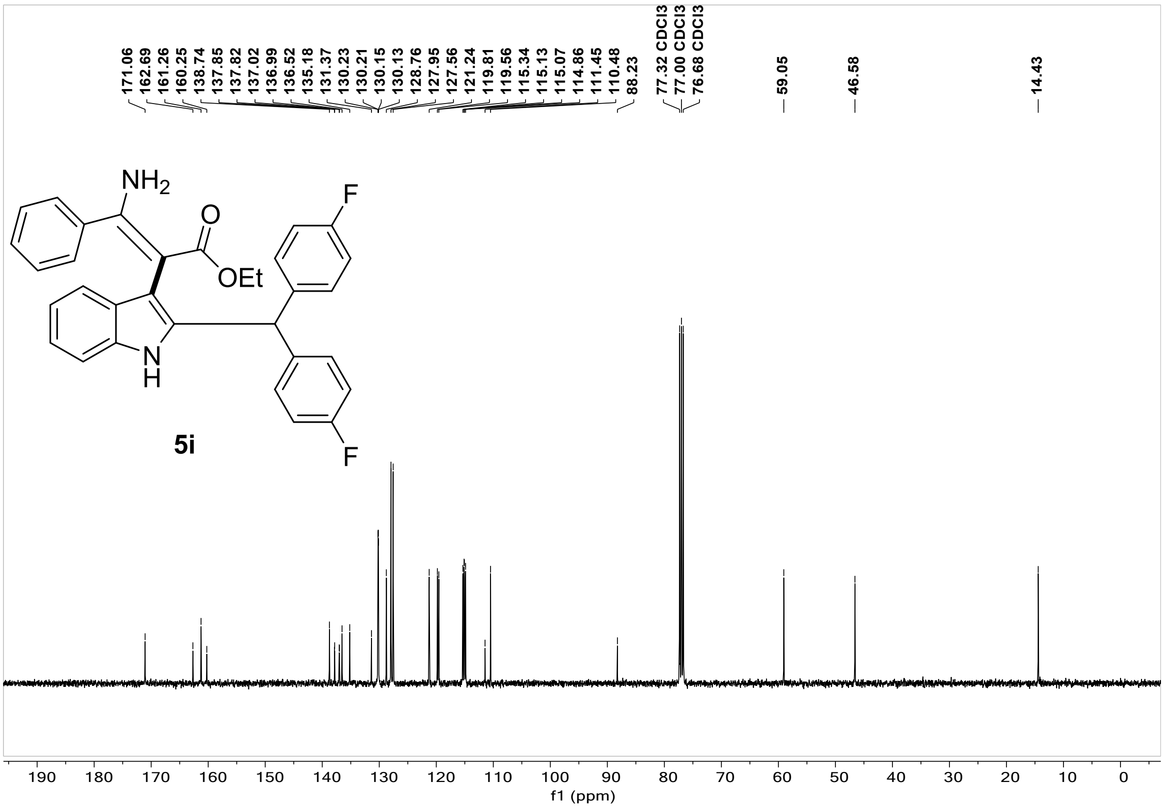
171.06
162.69
161.26
160.25
138.74
137.85
137.82
137.02
136.99
136.52
135.18
131.37
130.23
130.21
130.15
130.13
128.76
127.95
127.56
121.24
119.81
119.56
115.34
115.13
115.07
114.86
111.45
110.48
88.23
77.32 CDCl3
77.00 CDCl3
76.68 CDCl3
59.05
46.58
14.43
NH2
O
OEt
F
N
H
F
5i
f1 (ppm)

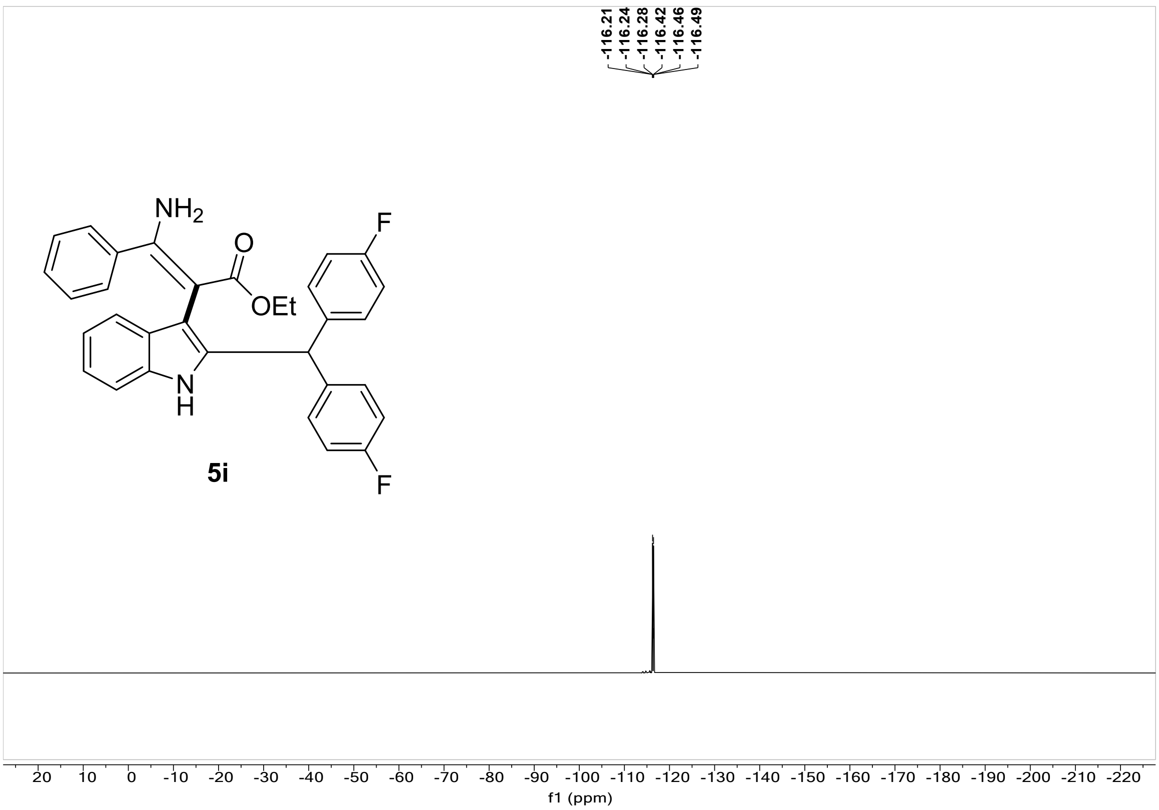

-116.21
-116.24
-116.28
-116.42
-116.46
-116.49
NH2
O
OEt
F
N
H
F
5i
20 10 0 -10 -20 -30 -40 -50 -60 -70 -80 -90 -100 -110 -120 -130 -140 -150 -160 -170 -180 -190 -200 -210 -220
f1 (ppm)

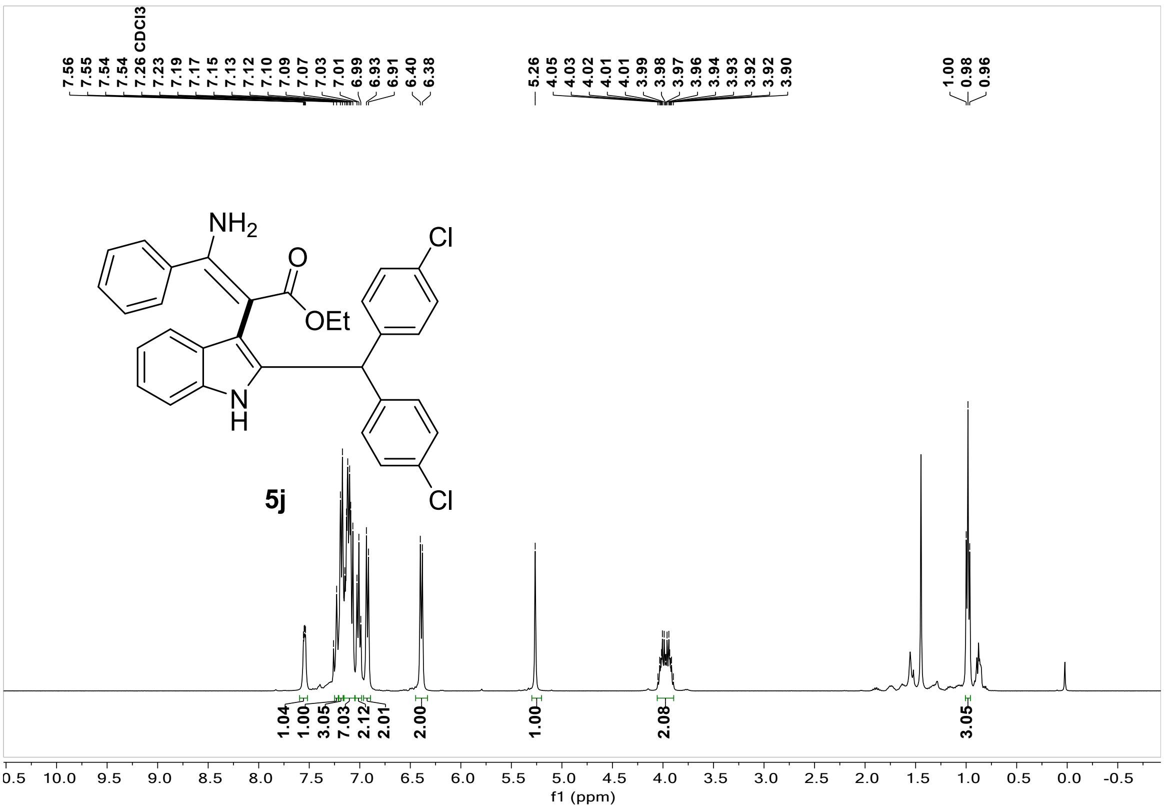

7.56
7.55
7.54
7.26 CDCl3
7.23
7.19
7.17
7.15
7.13
7.12
7.10
7.09
7.07
7.03
7.01
6.99
6.93
6.91
6.40
6.38
5.26
4.05
4.03
4.02
4.01
3.99
3.98
3.97
3.96
3.94
3.93
3.92
3.90
1.00
0.98
0.96
NH2
O
OEt
Cl
N
H
Cl
5j
1.04
1.00
3.05
7.03
2.12
2.01
2.00
1.00
2.08
3.05
0.5 10.0 9.5 9.0 8.5 8.0 7.5 7.0 6.5 6.0 5.5 5.0 4.5 4.0 3.5 3.0 2.5 2.0 1.5 1.0 0.5 0.0 -0.5
f1 (ppm)

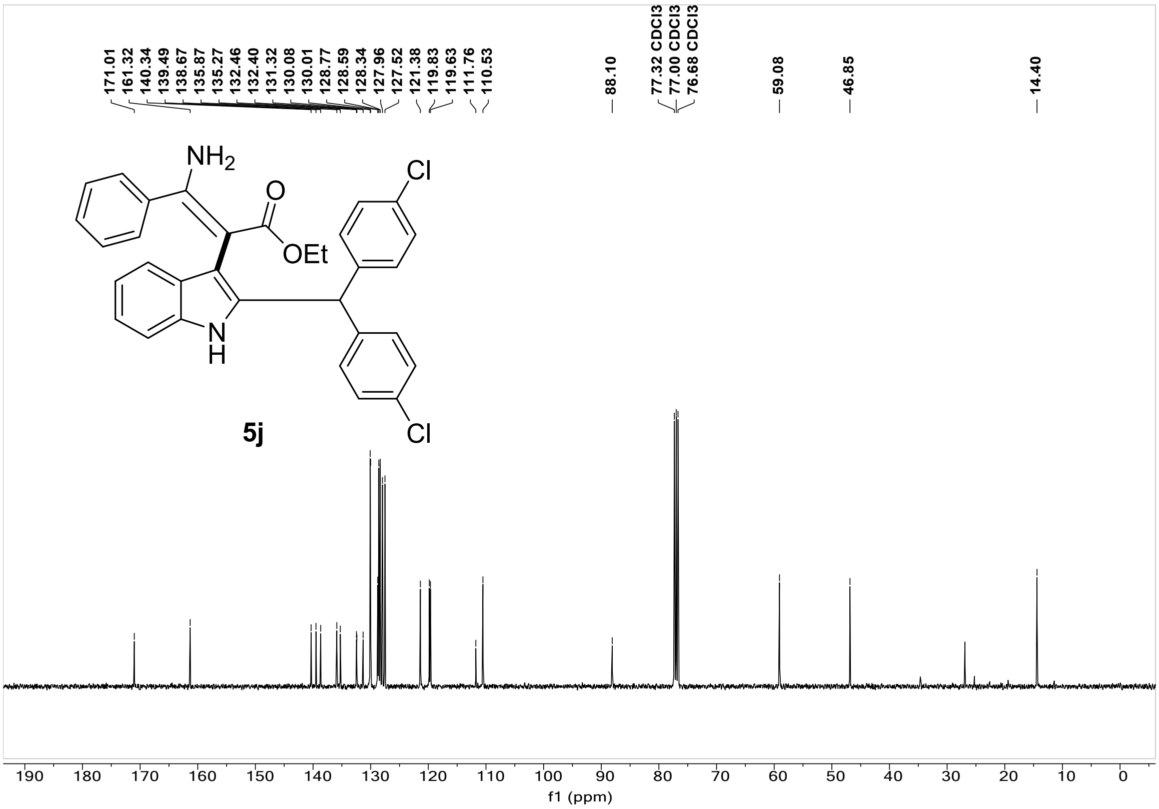
171.01
161.32
140.34
139.49
138.67
135.87
135.27
132.46
132.40
131.32
130.08
130.01
128.77
128.59
128.34
127.96
127.52
121.38
119.83
119.63
111.76
110.53
88.10
77.32 CDCl3
77.00 CDCl3
76.68 CDCl3
59.08
46.85
14.40
NH2
O
OEt
Cl
Cl
N
H
5j
f1 (ppm)

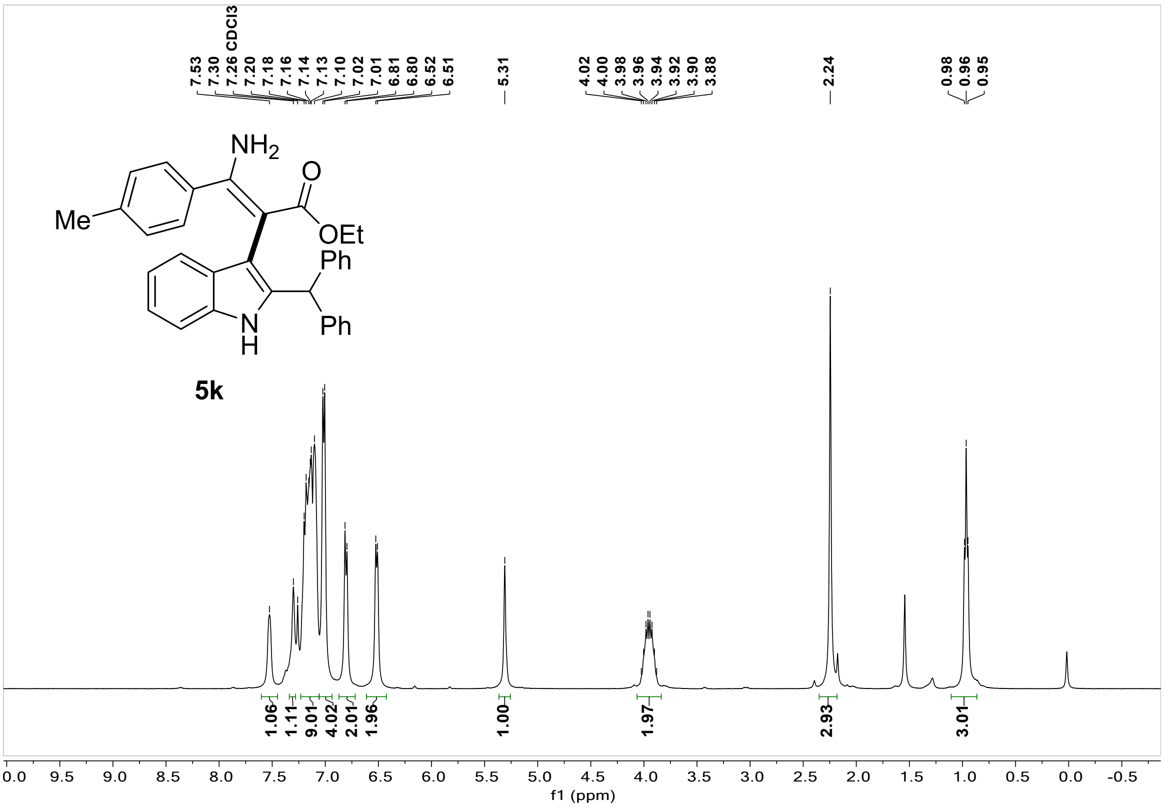
7.53
7.30
7.26 CDCl3
7.20
7.18
7.16
7.14
7.13
7.10
7.02
7.01
6.81
6.80
6.52
6.51
5.31
4.02
4.00
3.98
3.96
3.94
3.92
3.90
3.88
2.24
0.98
0.96
0.95
NH2
O
Me
OEt
Ph
Ph
N
H
5k
1.06
1.11
9.01
4.02
2.01
1.96
1.00
1.97
2.93
3.01
f1 (ppm)

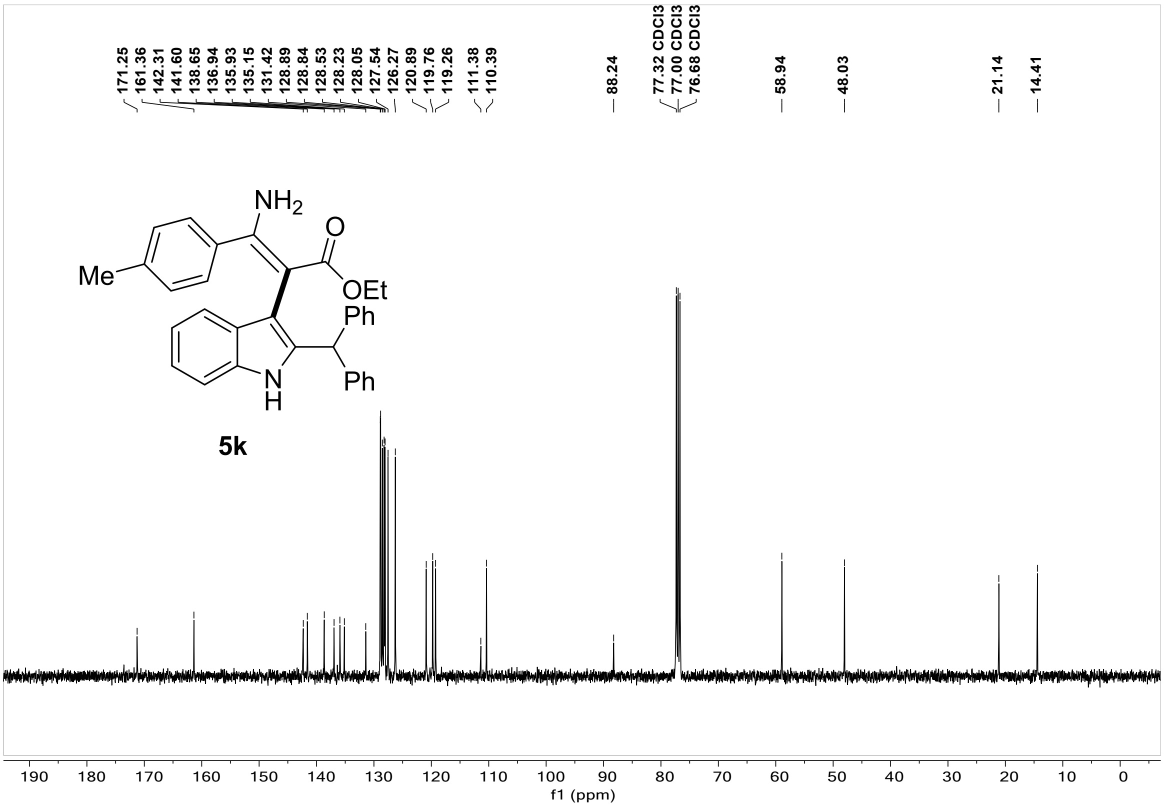
171.25
161.36
142.31
141.60
138.65
136.94
135.93
135.15
131.42
128.89
128.84
128.53
128.23
128.05
127.54
126.27
120.89
119.76
119.26
111.38
110.39
88.24
77.32 CDCl3
77.00 CDCl3
76.68 CDCl3
58.94
48.03
21.14
14.41
NH2
O
Me
OEt
Ph
N
H
Ph
5k
190 180 170 160 150 140 130 120 110 100 90 80 70 60 50 40 30 20 10 0
f1 (ppm)

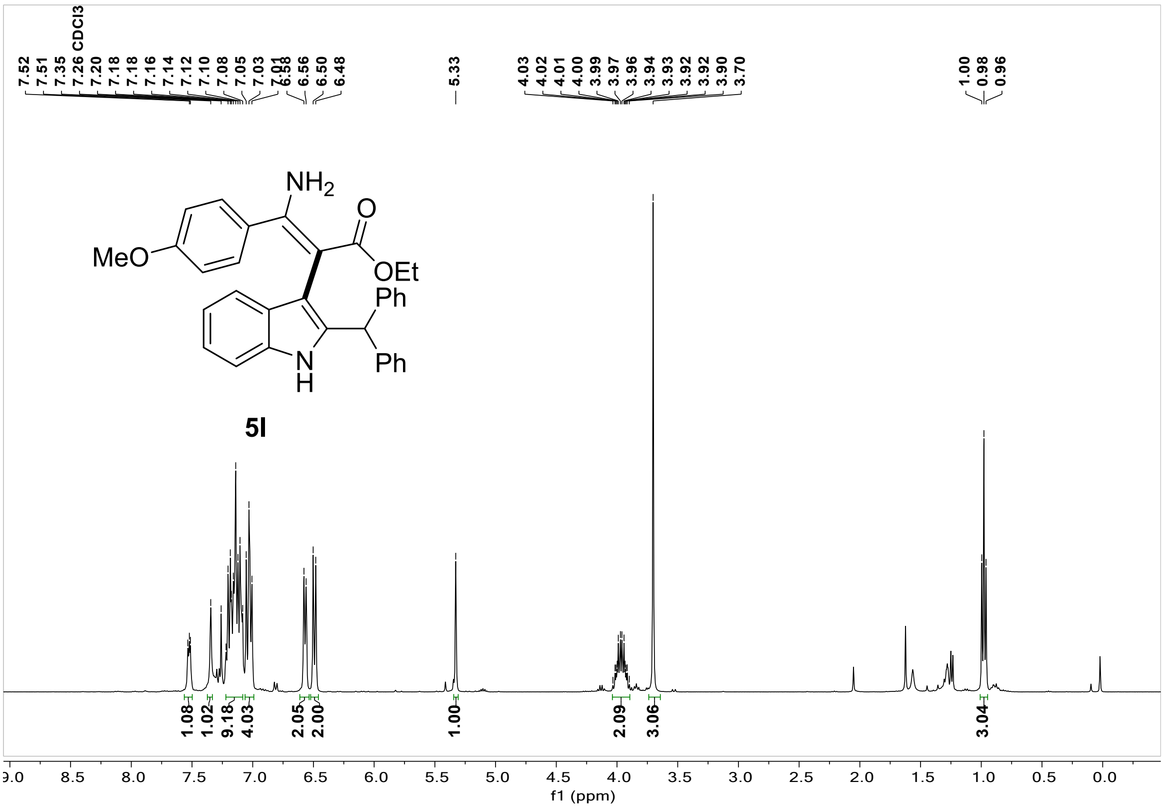
7.52
7.51
7.35
7.26 CDCl3
7.20
7.18
7.18
7.16
7.14
7.12
7.10
7.08
7.05
7.03
7.01
6.58
6.56
6.50
6.48
5.33
4.03
4.02
4.01
4.00
3.99
3.97
3.96
3.94
3.93
3.92
3.92
3.90
3.70
1.00
0.98
0.96
NH2
O
MeO
OEt
Ph
N
H
Ph
5l
1.08
1.02
9.18
4.03
2.05
2.00
1.00
2.09
3.06
3.04
8.5 8.0 7.5 7.0 6.5 6.0 5.5 5.0 4.5 4.0 3.5 3.0 2.5 2.0 1.5 1.0 0.5 0.0
f1 (ppm)

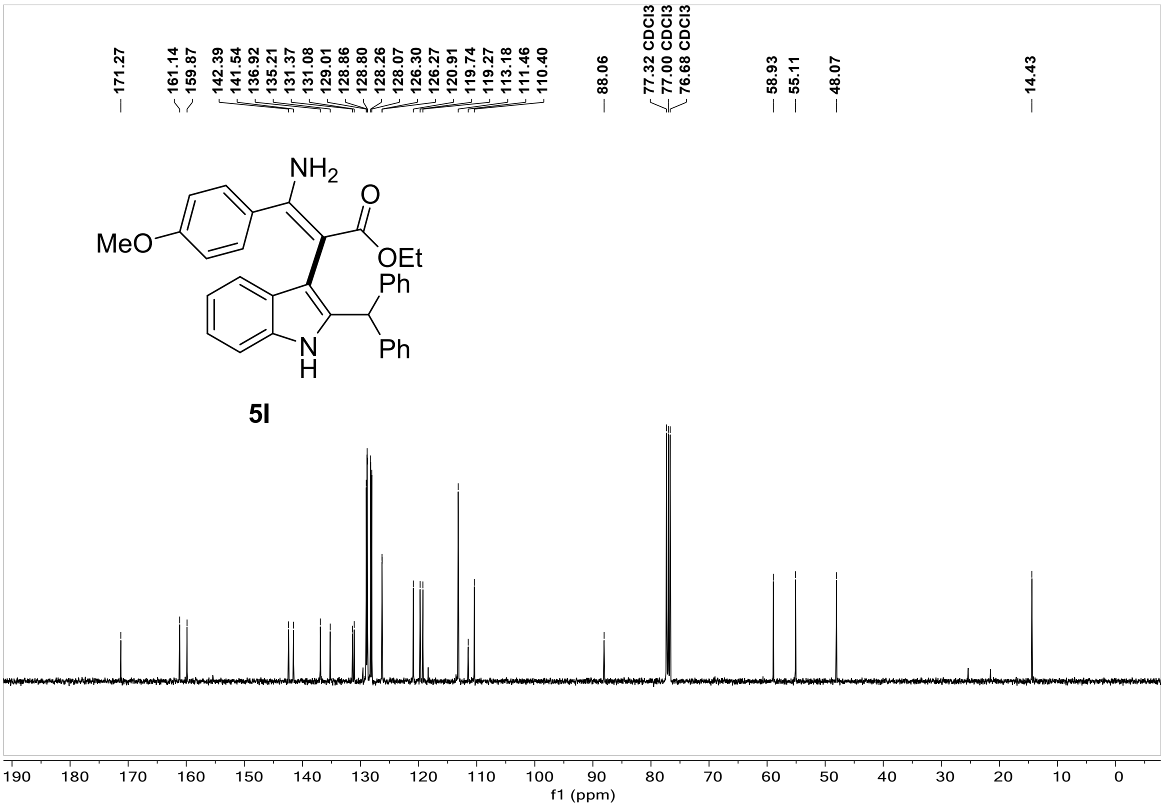
171.27
161.14
159.87
142.39
141.54
136.92
135.21
131.37
131.08
129.01
128.86
128.80
128.26
128.07
126.30
126.27
120.91
119.74
119.27
113.18
111.46
110.40
88.06
77.32 CDCl3
77.00 CDCl3
76.68 CDCl3
58.93
55.11
48.07
14.43
NH2
O
MeO
OEt
Ph
N
H
Ph
5l
190 180 170 160 150 140 130 120 110 100 90 80 70 60 50 40 30 20 10 0
f1 (ppm)

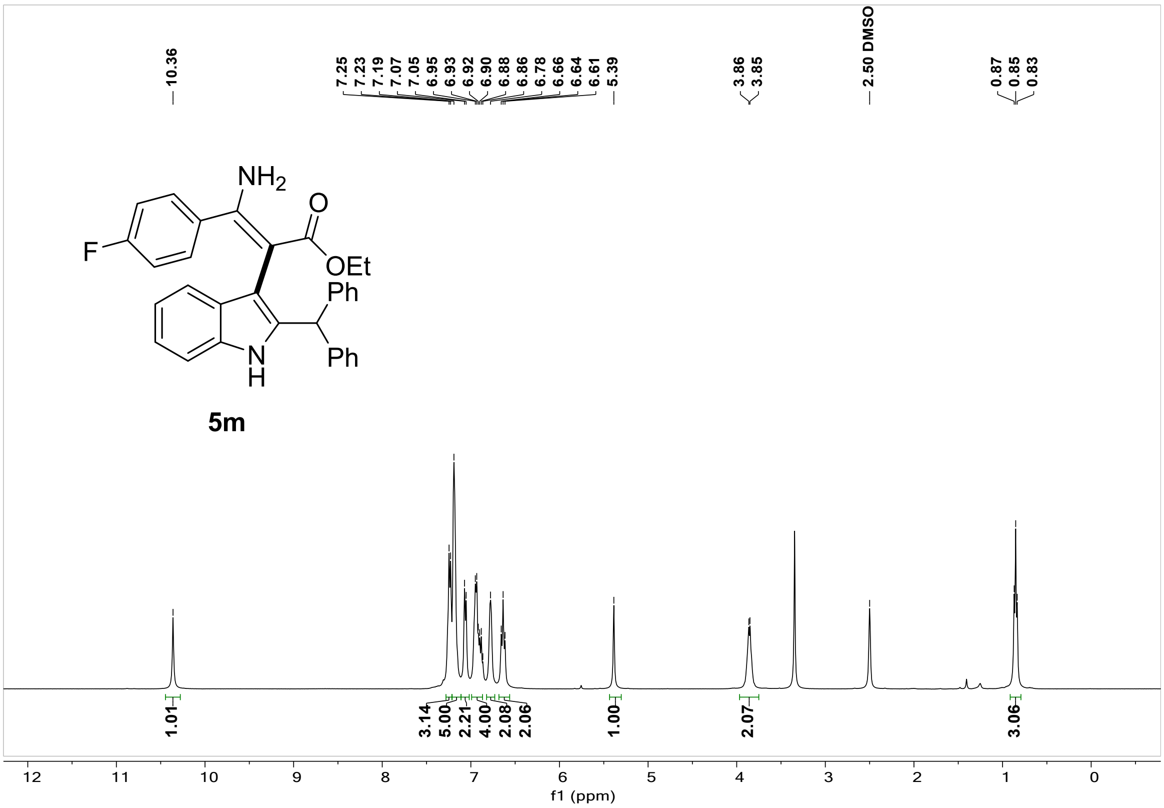
10.36
7.25
7.23
7.19
7.07
7.05
6.95
6.93
6.92
6.90
6.88
6.86
6.78
6.66
6.64
6.61
5.39
3.86
3.85
2.50 DMSO
0.87
0.85
0.83
NH2
O
F
OEt
Ph
N
H
Ph
5m
1.01
3.14
5.00
2.21
4.00
2.08
2.06
1.00
2.07
3.06
12 11 10 9 8 7 6 5 4 3 2 1 0
f1 (ppm)

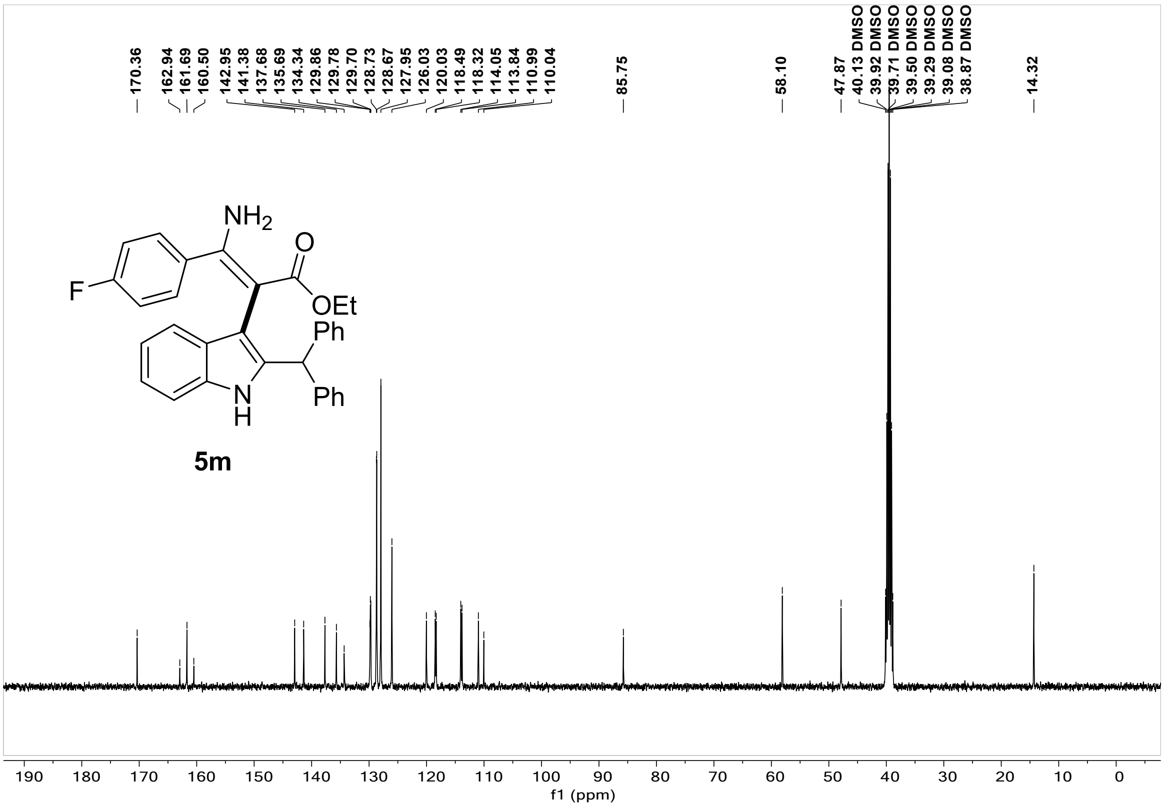
170.36
162.94
161.69
160.50
142.95
141.38
137.68
135.69
134.34
129.86
129.78
129.70
128.73
128.67
127.95
126.03
120.03
118.49
118.32
114.05
113.84
110.99
110.04
85.75
58.10
47.87
40.13 DMSO
39.92 DMSO
39.71 DMSO
39.50 DMSO
39.29 DMSO
39.08 DMSO
38.87 DMSO
14.32
NH2
O
F
OEt
Ph
N
H
Ph
5m
190 180 170 160 150 140 130 120 110 100 90 80 70 60 50 40 30 20 10 0
f1 (ppm)

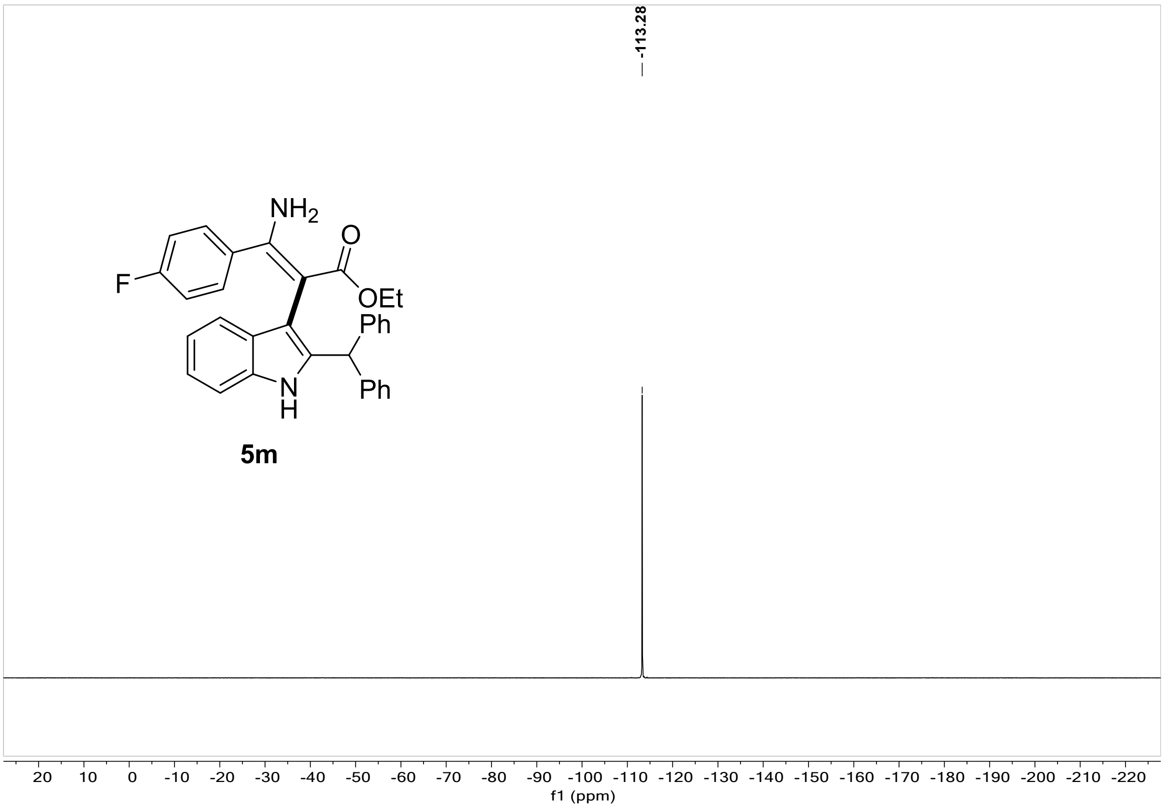
-113.28
NH2
O
F
OEt
Ph
N
H
Ph
5m
20 10 0 -10 -20 -30 -40 -50 -60 -70 -80 -90 -100 -110 -120 -130 -140 -150 -160 -170 -180 -190 -200 -210 -220
f1 (ppm)

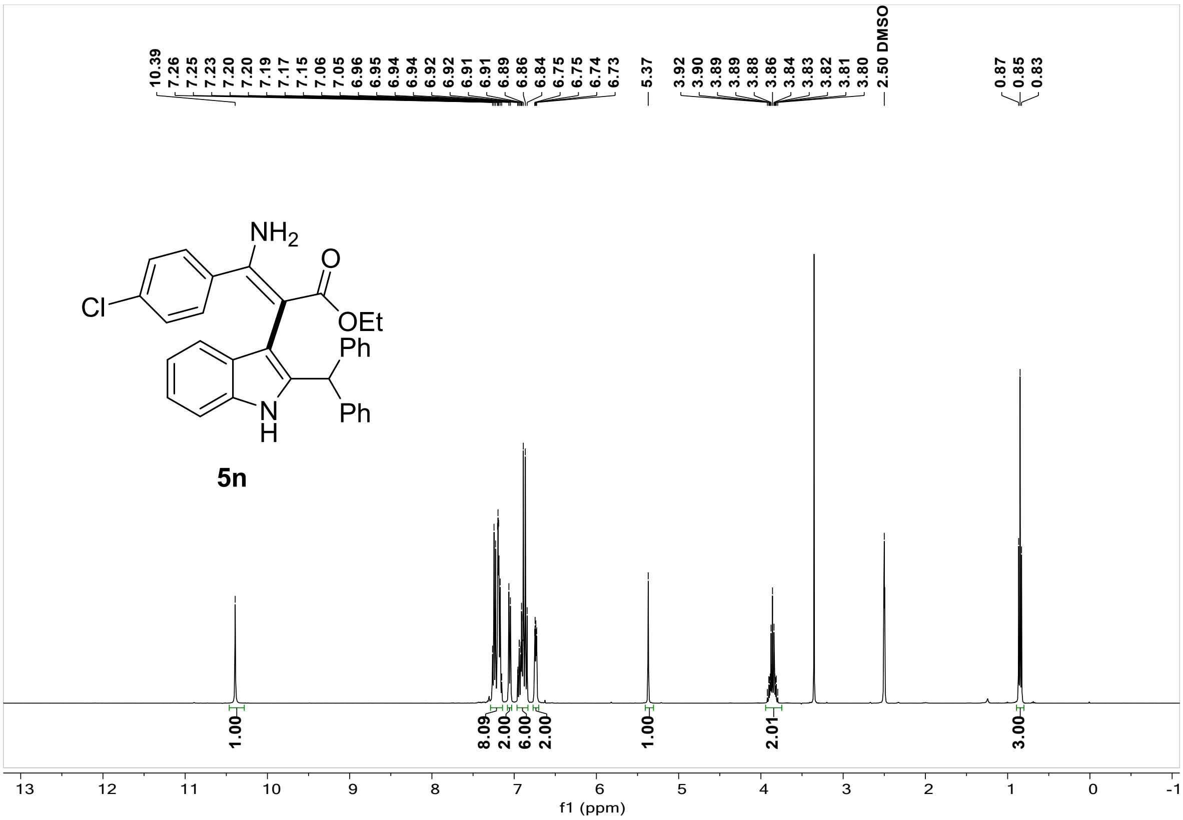
10.39
7.26
7.25
7.23
7.20
7.20
7.19
7.17
7.15
7.06
7.05
6.96
6.95
6.94
6.94
6.92
6.92
6.91
6.91
6.89
6.86
6.84
6.75
6.75
6.74
6.73
5.37
3.92
3.90
3.89
3.89
3.88
3.86
3.84
3.83
3.82
3.81
3.80
2.50 DMSO
0.87
0.85
0.83
NH2
O
Cl
OEt
Ph
Ph
N
H
5n
1.00
8.09
2.00
6.00
2.00
1.00
2.01
3.00
f1 (ppm)

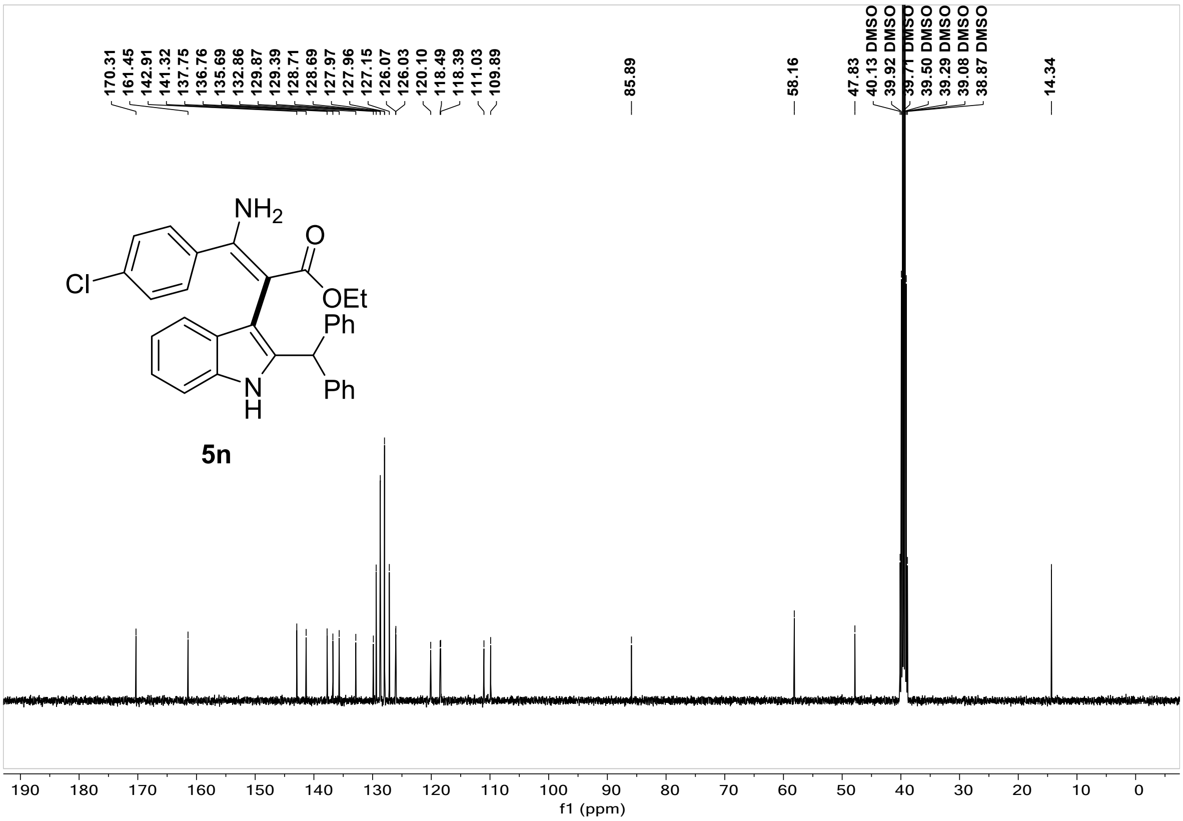
170.31
161.45
142.91
141.32
137.75
136.76
135.69
132.86
129.87
129.39
128.71
128.69
127.97
127.96
127.15
126.07
126.03
120.10
118.49
118.39
111.03
109.89
85.89
58.16
47.83
40.13 DMSO
39.92 DMSO
39.71 DMSO
39.50 DMSO
39.29 DMSO
39.08 DMSO
38.87 DMSO
14.34
NH2
O
Cl
OEt
Ph
Ph
N
H
5n
f1 (ppm)

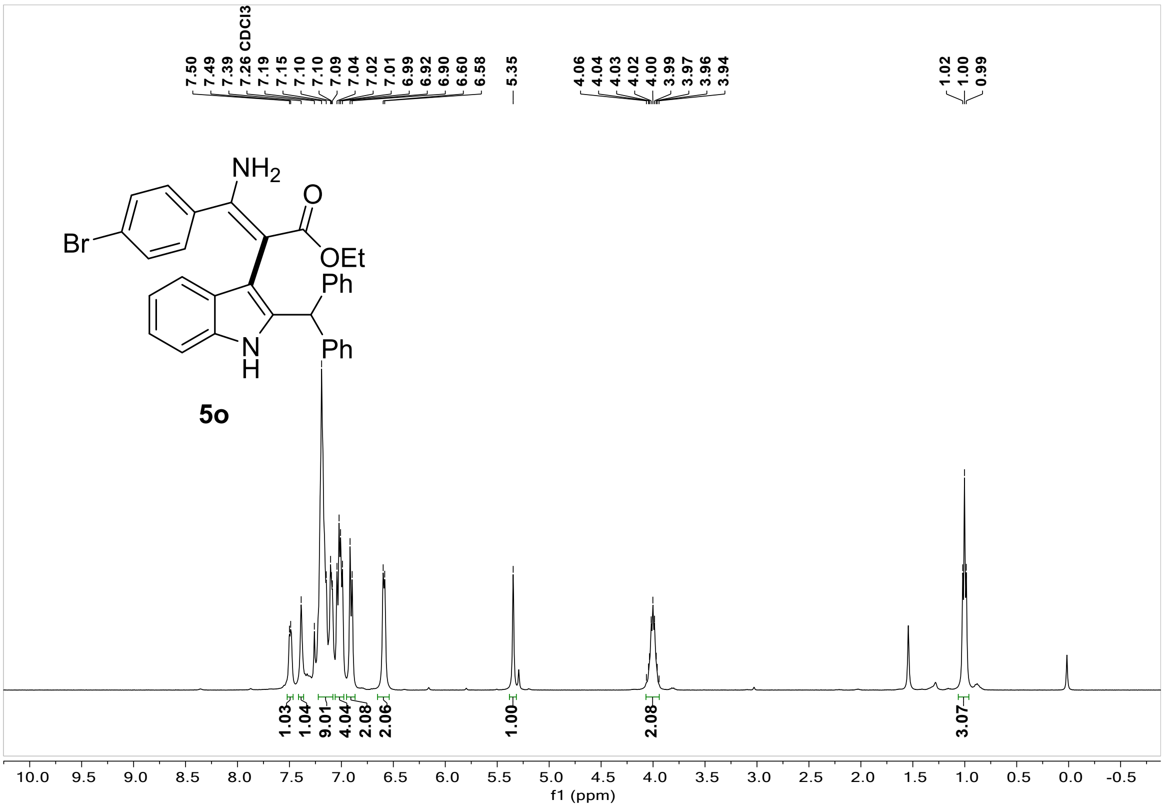
7.50
7.49
7.39
7.26 CDCl3
7.19
7.15
7.10
7.10
7.09
7.04
7.02
7.01
6.99
6.92
6.90
6.60
6.58
5.35
4.06
4.04
4.03
4.02
4.00
3.99
3.97
3.96
3.94
1.02
1.00
0.99
NH2
O
Br
OEt
Ph
N
H
Ph
5o
1.03
1.04
9.01
4.04
2.08
2.06
1.00
2.08
3.07
10.0 9.5 9.0 8.5 8.0 7.5 7.0 6.5 6.0 5.5 5.0 4.5 4.0 3.5 3.0 2.5 2.0 1.5 1.0 0.5 0.0 -0.5
f1 (ppm)

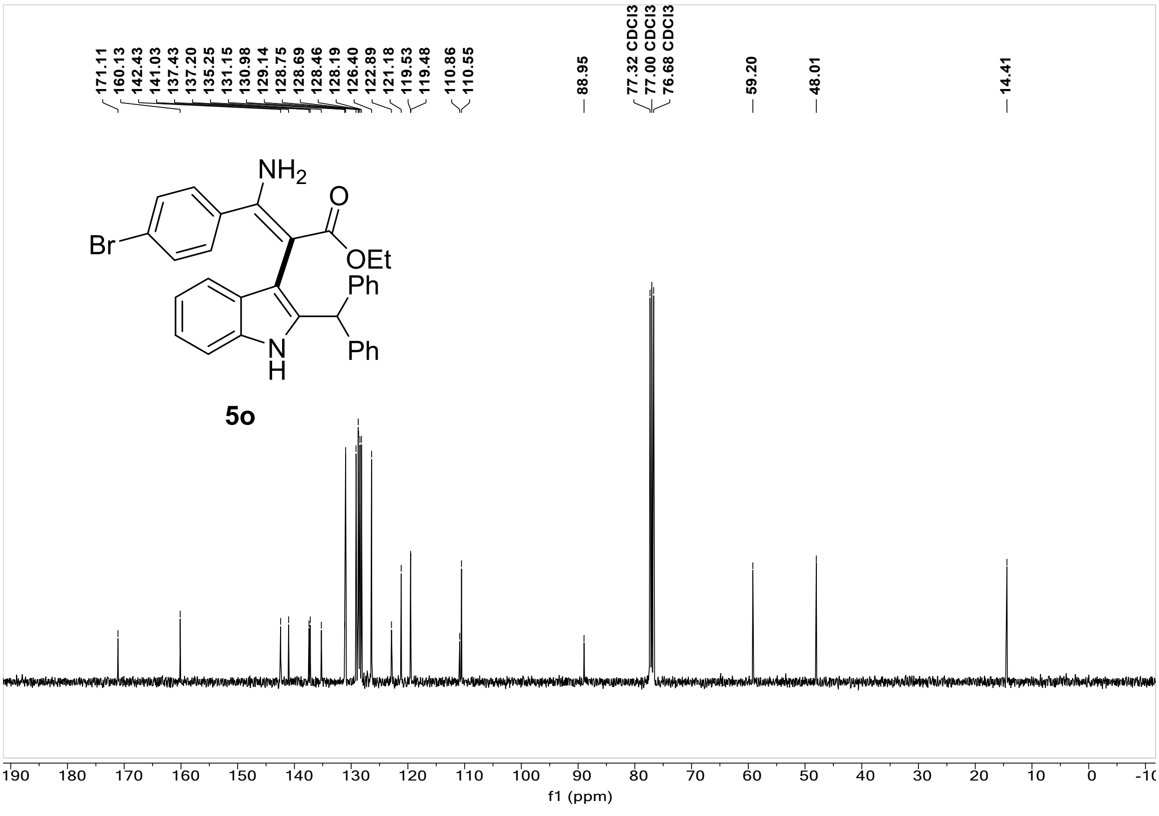
171.11
160.13
142.43
141.03
137.43
137.20
135.25
131.15
130.98
129.14
128.75
128.69
128.46
128.19
126.40
122.89
121.18
119.53
119.48
110.86
110.55
88.95
77.32 CDCl3
77.00 CDCl3
76.68 CDCl3
59.20
48.01
14.41
NH2
O
Br
OEt
Ph
N
H
Ph
5o
190 180 170 160 150 140 130 120 110 100 90 80 70 60 50 40 30 20 10 0 -10
f1 (ppm)

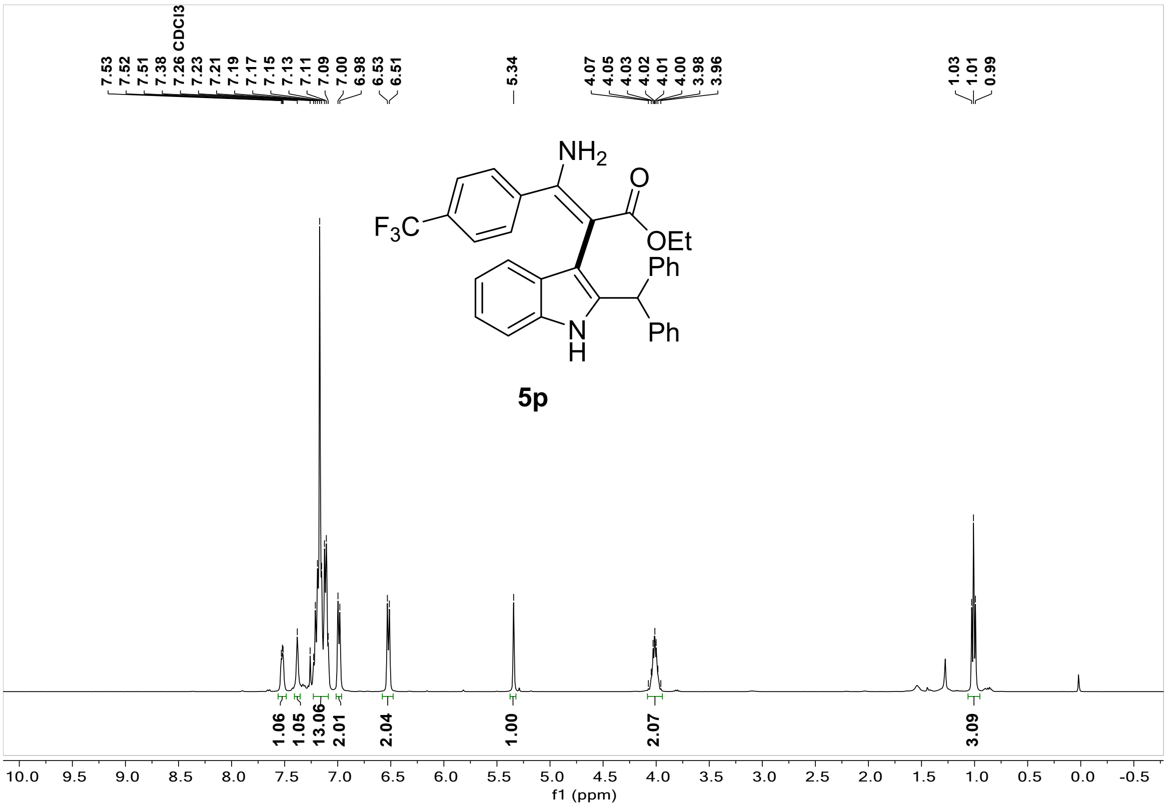
7.53
7.52
7.51
7.38
7.26 CDCl3
7.23
7.21
7.19
7.17
7.15
7.13
7.11
7.09
7.00
6.98
6.53
6.51
5.34
4.07
4.05
4.03
4.02
4.01
4.00
3.98
3.96
1.03
1.01
0.99
NH2
O
F3C
OEt
Ph
N
H
Ph
5p
1.06
1.05
13.06
2.01
2.04
1.00
2.07
3.09
10.0 9.5 9.0 8.5 8.0 7.5 7.0 6.5 6.0 5.5 5.0 4.5 4.0 3.5 3.0 2.5 2.0 1.5 1.0 0.5 0.0 -0.5
f1 (ppm)

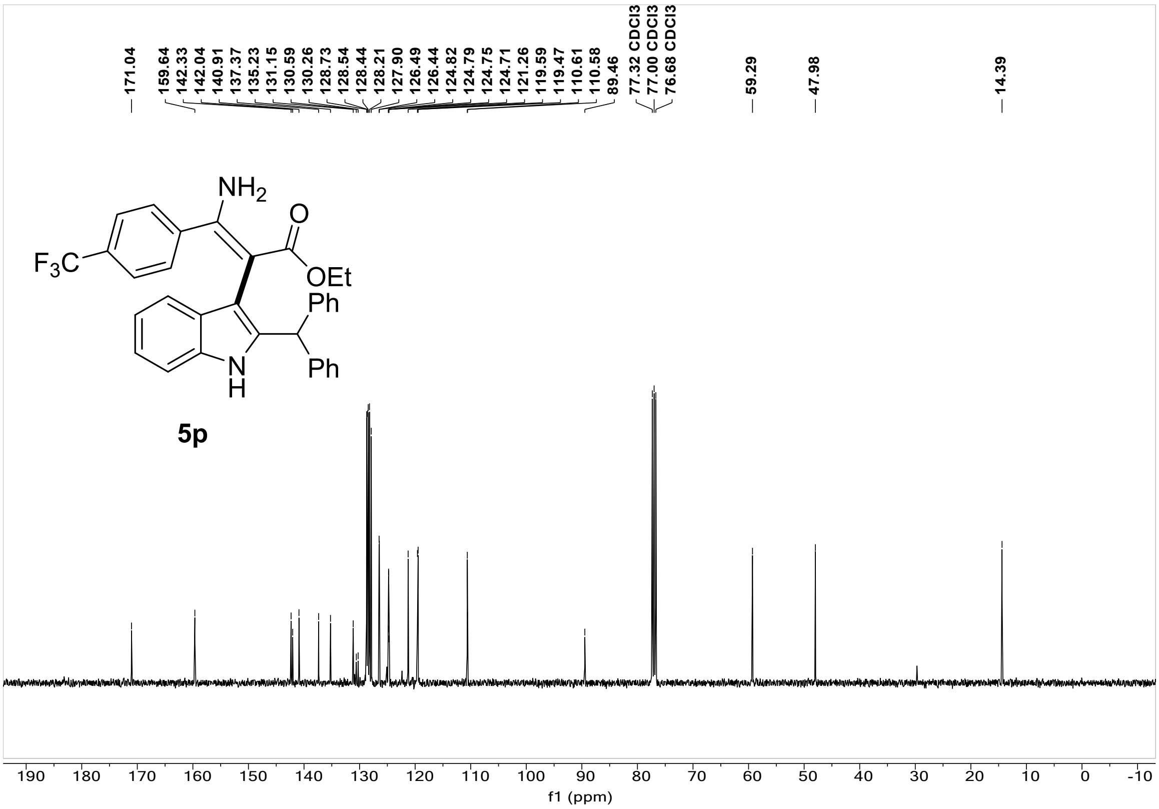
171.04
159.64
142.33
142.04
140.91
137.37
135.23
131.15
130.59
130.26
128.73
128.54
128.44
128.21
127.90
126.49
126.44
124.82
124.79
124.75
124.71
121.26
119.59
119.47
110.61
110.58
89.46
77.32 CDCl3
77.00 CDCl3
76.68 CDCl3
59.29
47.98
14.39
NH2
O
F3C
OEt
Ph
N
H
Ph
5p
190 180 170 160 150 140 130 120 110 100 90 80 70 60 50 40 30 20 10 0 -10
f1 (ppm)

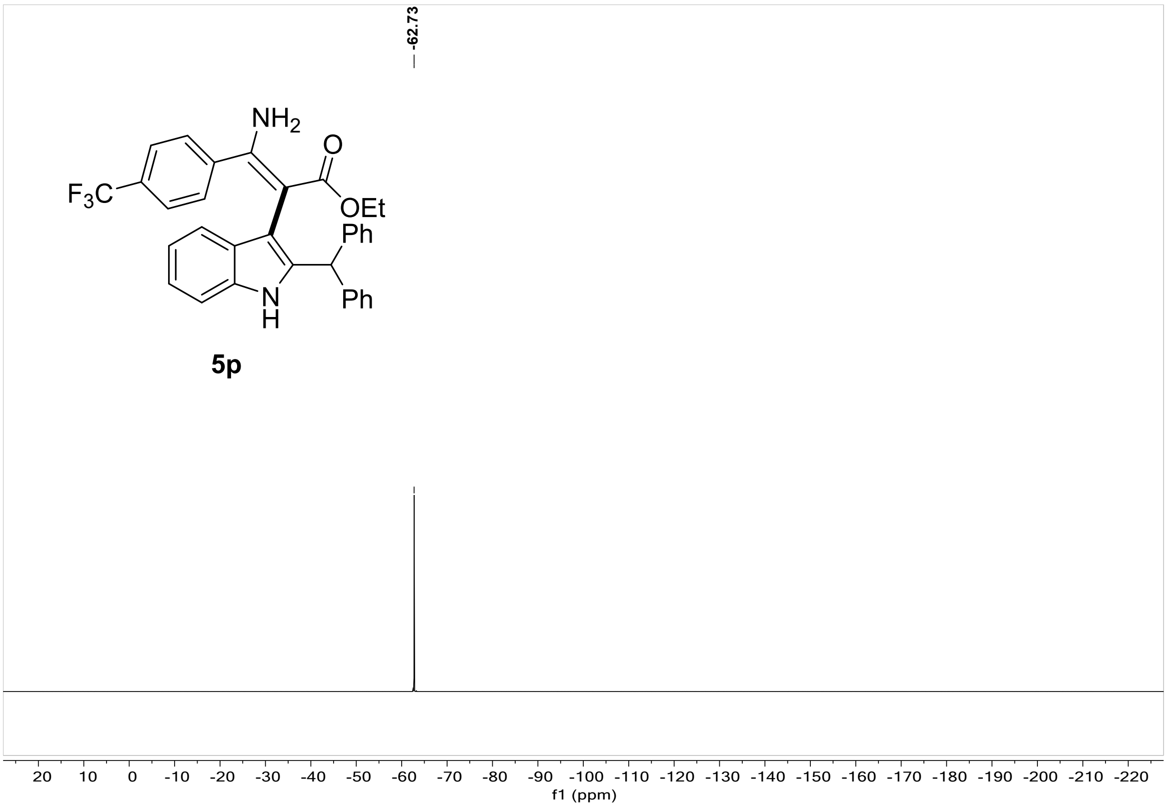

-62.73
NH2
O
F3C
OEt
Ph
N
H
Ph
5p
20 10 0 -10 -20 -30 -40 -50 -60 -70 -80 -90 -100 -110 -120 -130 -140 -150 -160 -170 -180 -190 -200 -210 -220
f1 (ppm)

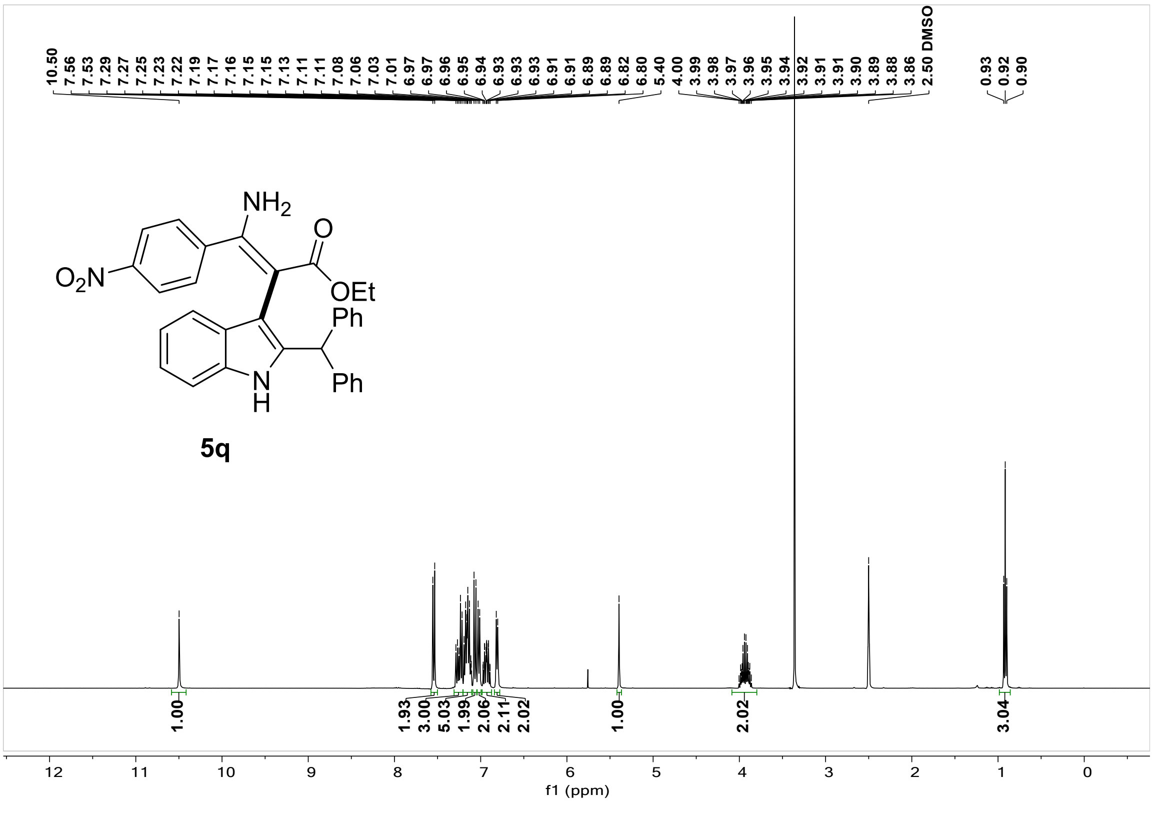

10.50 7.56 7.53 7.29 7.27 7.25 7.23 7.22 7.19 7.17 7.16 7.15 7.15 7.13 7.11 7.11 7.08 7.06 7.03 7.01 6.97 6.97 6.96 6.95 6.94 6.93 6.93 6.93 6.91 6.91 6.89 6.89 6.82 6.80 5.40 4.00 3.99 3.98 3.97 3.96 3.95 3.94 3.92 3.91 3.91 3.90 3.89 3.88 3.86 2.50 DMSO 0.93 0.92 0.90
NH2
O
O2N
OEt
Ph
N
H
Ph
5q
1.00 1.93 3.00 5.03 1.99 2.06 2.11 2.02 1.00 2.02 3.04
12 11 10 9 8 7 6 5 4 3 2 1 0
f1 (ppm)

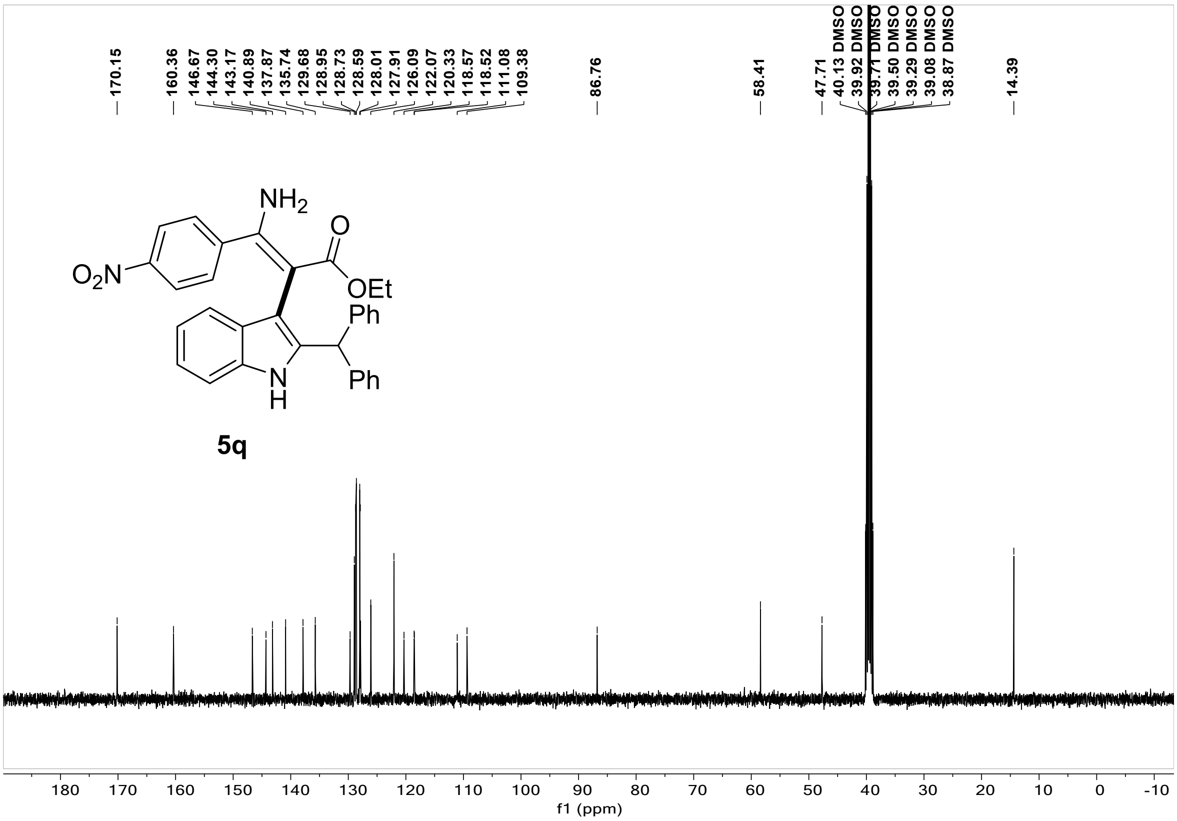
170.15
160.36
146.67
144.30
143.17
140.89
137.87
135.74
129.68
128.95
128.73
128.59
128.01
127.91
126.09
122.07
120.33
118.57
118.52
111.08
109.38
86.76
58.41
47.71
40.13 DMSO
39.92 DMSO
39.71 DMSO
39.50 DMSO
39.29 DMSO
39.08 DMSO
38.87 DMSO
14.39
NH2
O
O2N
OEt
Ph
N
H
Ph
5q
f1 (ppm)

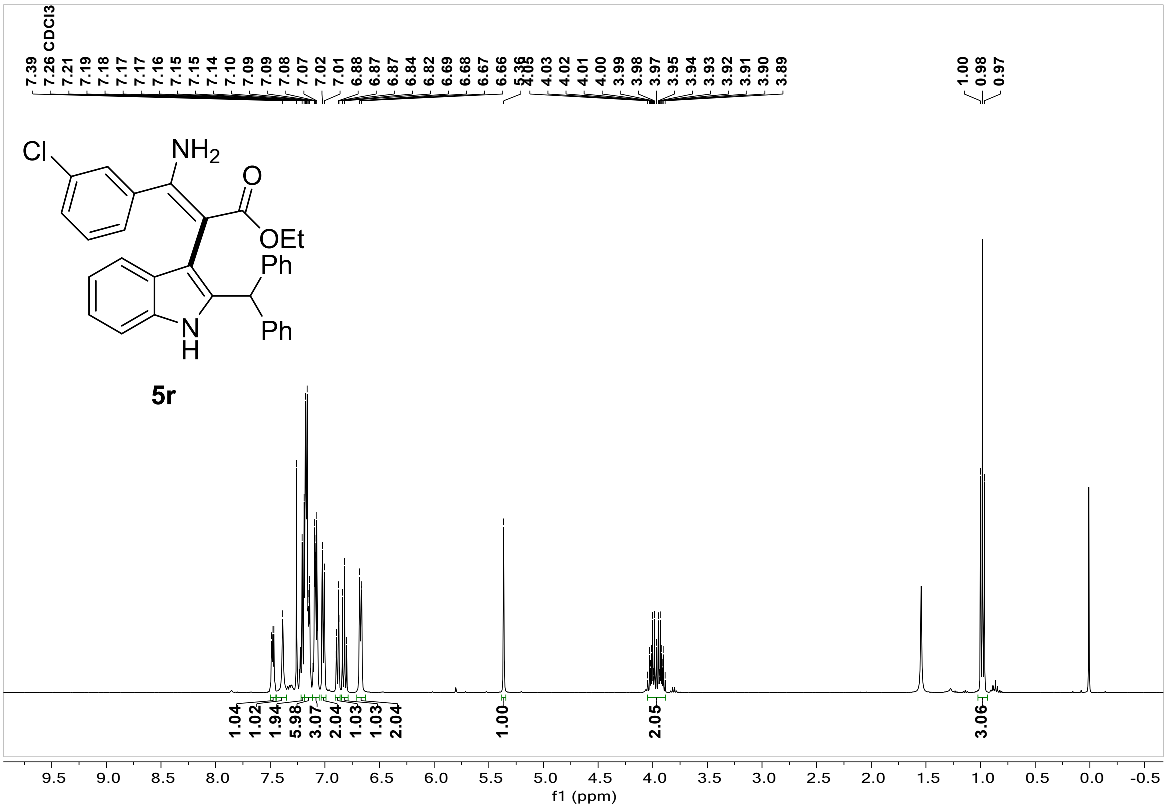
7.39
7.26 CDCl3
7.21
7.19
7.18
7.17
7.17
7.16
7.15
7.15
7.14
7.10
7.09
7.09
7.08
7.07
7.02
7.01
6.88
6.87
6.87
6.84
6.82
6.69
6.68
6.67
6.66
5.36
4.05
4.03
4.02
4.01
4.00
3.99
3.98
3.97
3.95
3.94
3.93
3.92
3.91
3.90
3.89
1.00
0.98
0.97
Cl
NH2
O
OEt
Ph
N
H
Ph
5r
1.04
1.02
1.94
5.98
3.07
2.04
1.03
1.03
2.04
1.00
2.05
3.06
f1 (ppm)

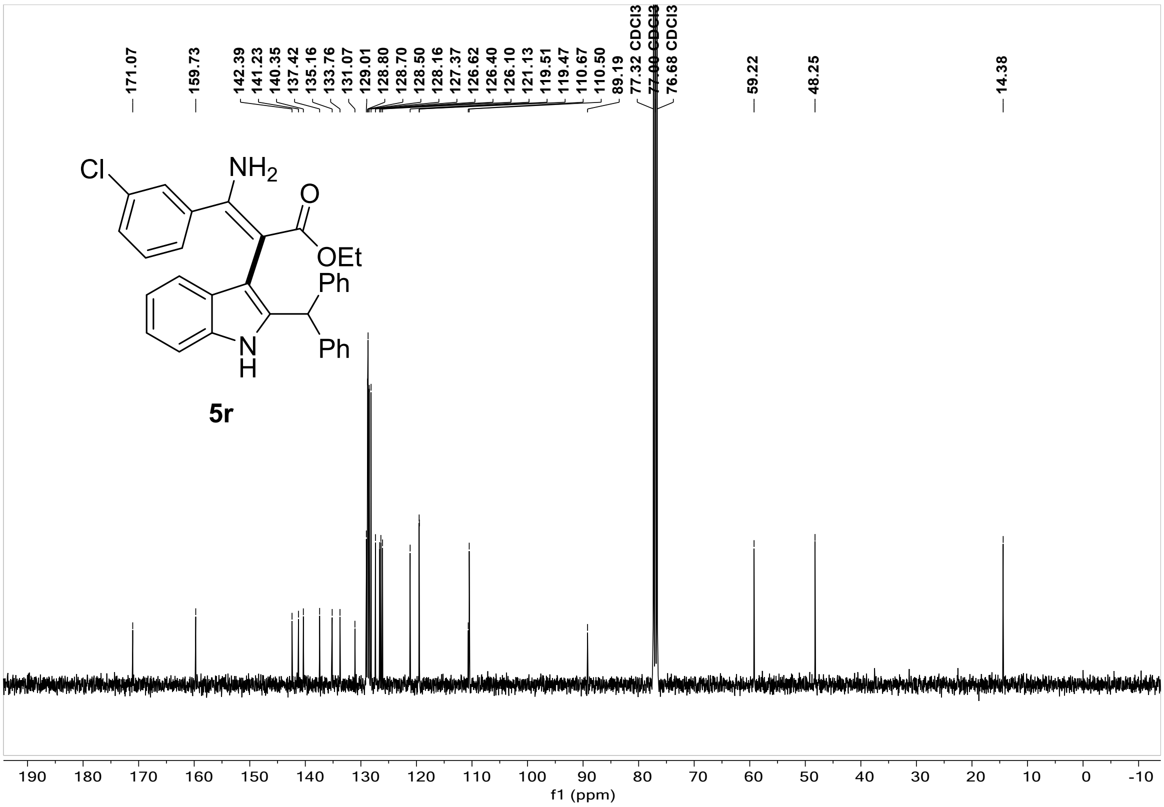
171.07
159.73
142.39
141.23
140.35
137.42
135.16
133.76
131.07
129.01
128.80
128.70
128.50
128.16
127.37
126.62
126.40
126.10
121.13
119.51
119.47
110.67
110.50
89.19
77.32 CDCl3
77.00 CDCl3
76.68 CDCl3
59.22
48.25
14.38
Cl
NH2
O
OEt
Ph
Ph
N
H
5r
190 180 170 160 150 140 130 120 110 100 90 80 70 60 50 40 30 20 10 0 -10
f1 (ppm)

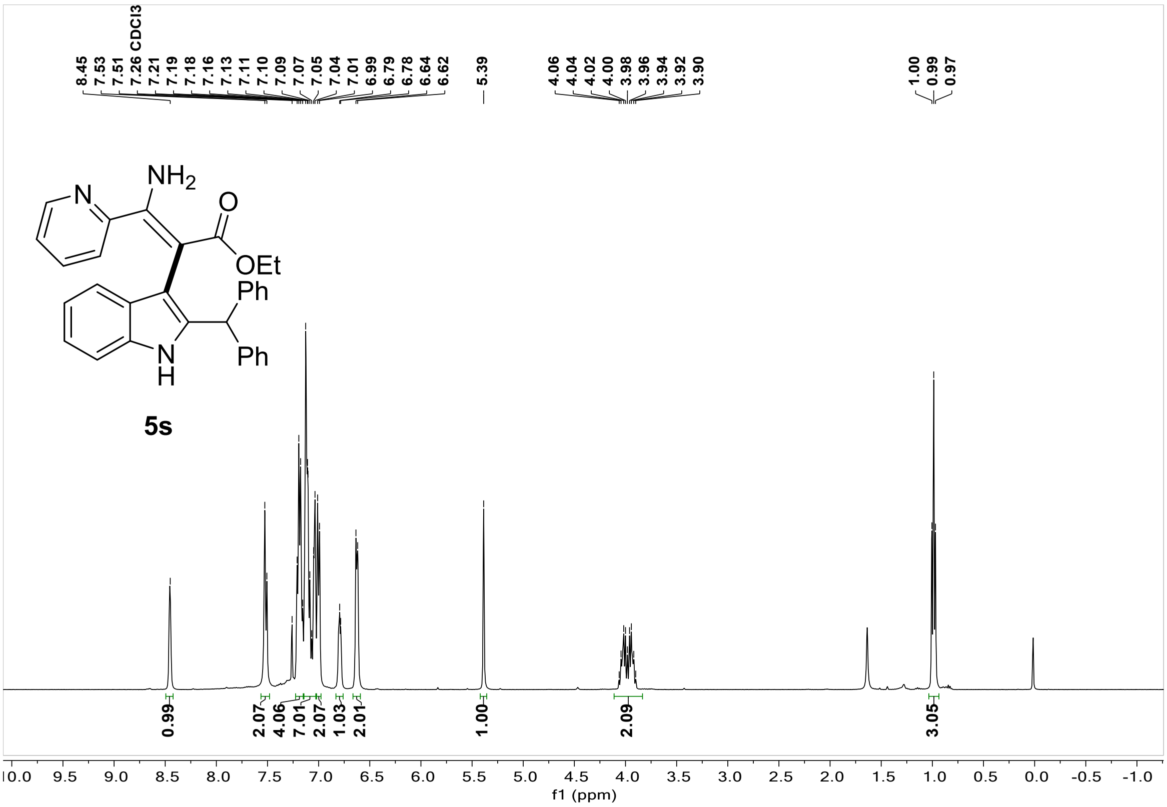
8.45
7.53
7.51
7.26 CDCl3
7.21
7.19
7.18
7.16
7.13
7.11
7.10
7.09
7.07
7.05
7.04
7.01
6.99
6.79
6.78
6.64
6.62
5.39
4.06
4.04
4.02
4.00
3.98
3.96
3.94
3.92
3.90
1.00
0.99
0.97
N
NH2
O
OEt
Ph
Ph
N
H
5s
0.99
2.07
4.06
7.01
2.07
1.03
2.01
1.00
2.09
3.05
10.0 9.5 9.0 8.5 8.0 7.5 7.0 6.5 6.0 5.5 5.0 4.5 4.0 3.5 3.0 2.5 2.0 1.5 1.0 0.5 0.0 -0.5 -1.0
f1 (ppm)

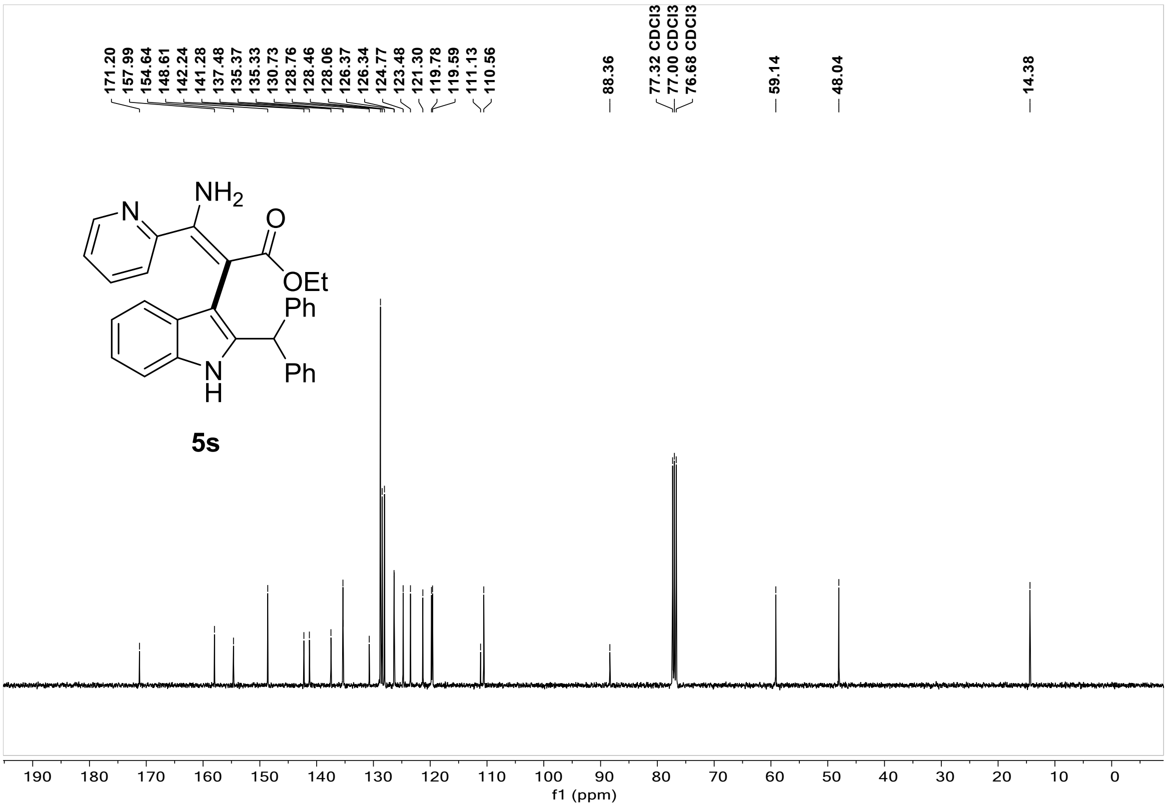
171.20
157.99
154.64
148.61
142.24
141.28
137.48
135.37
135.33
130.73
128.76
128.46
128.06
126.37
126.34
124.77
123.48
121.30
119.78
119.59
111.13
110.56
88.36
77.32 CDCl3
77.00 CDCl3
76.68 CDCl3
59.14
48.04
14.38
NH2
N
O
OEt
Ph
Ph
N
H
5s
190 180 170 160 150 140 130 120 110 100 90 80 70 60 50 40 30 20 10 0
f1 (ppm)

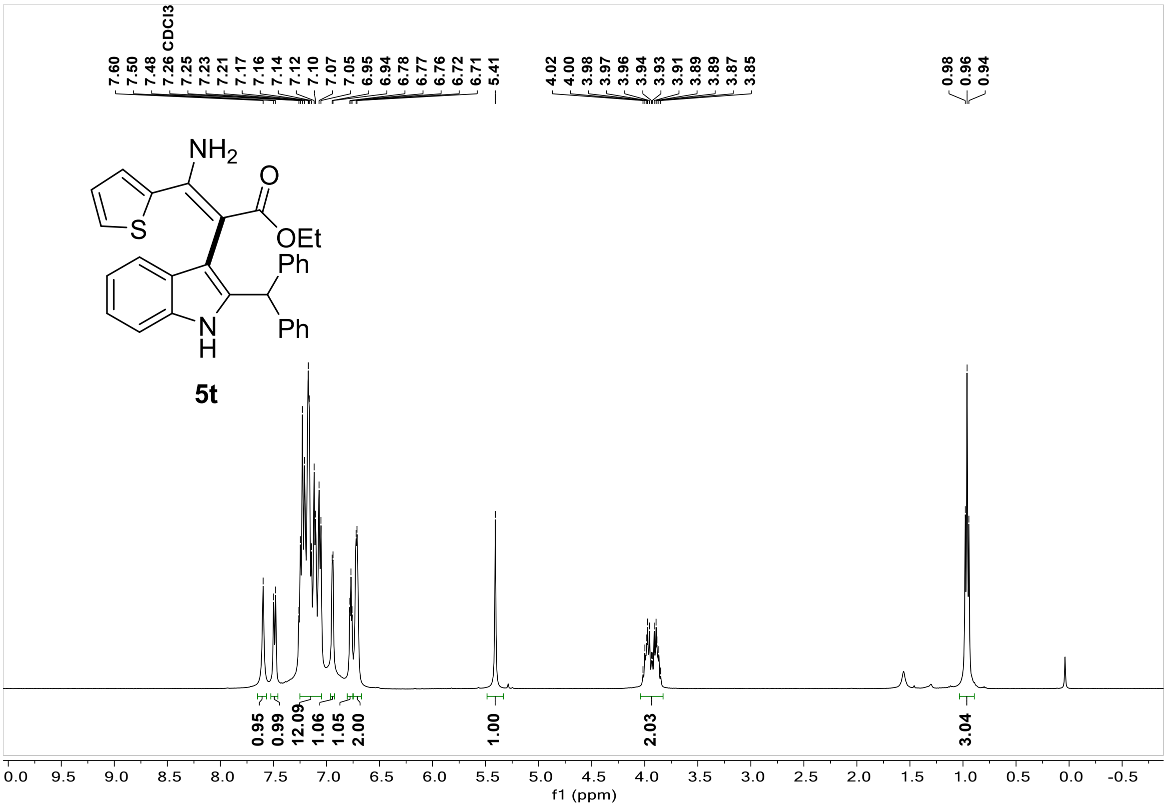
7.60
7.50
7.48
7.26 CDCl3
7.25
7.23
7.21
7.17
7.16
7.14
7.12
7.10
7.07
7.05
6.95
6.94
6.78
6.77
6.76
6.72
6.71
5.41
4.02
4.00
3.98
3.97
3.96
3.94
3.93
3.91
3.89
3.87
3.85
0.98
0.96
0.94
NH2
O
S
OEt
Ph
Ph
N
H
5t
0.95
0.99
12.09
1.06
1.05
2.00
1.00
2.03
3.04
0.0 9.5 9.0 8.5 8.0 7.5 7.0 6.5 6.0 5.5 5.0 4.5 4.0 3.5 3.0 2.5 2.0 1.5 1.0 0.5 0.0 -0.5
f1 (ppm)

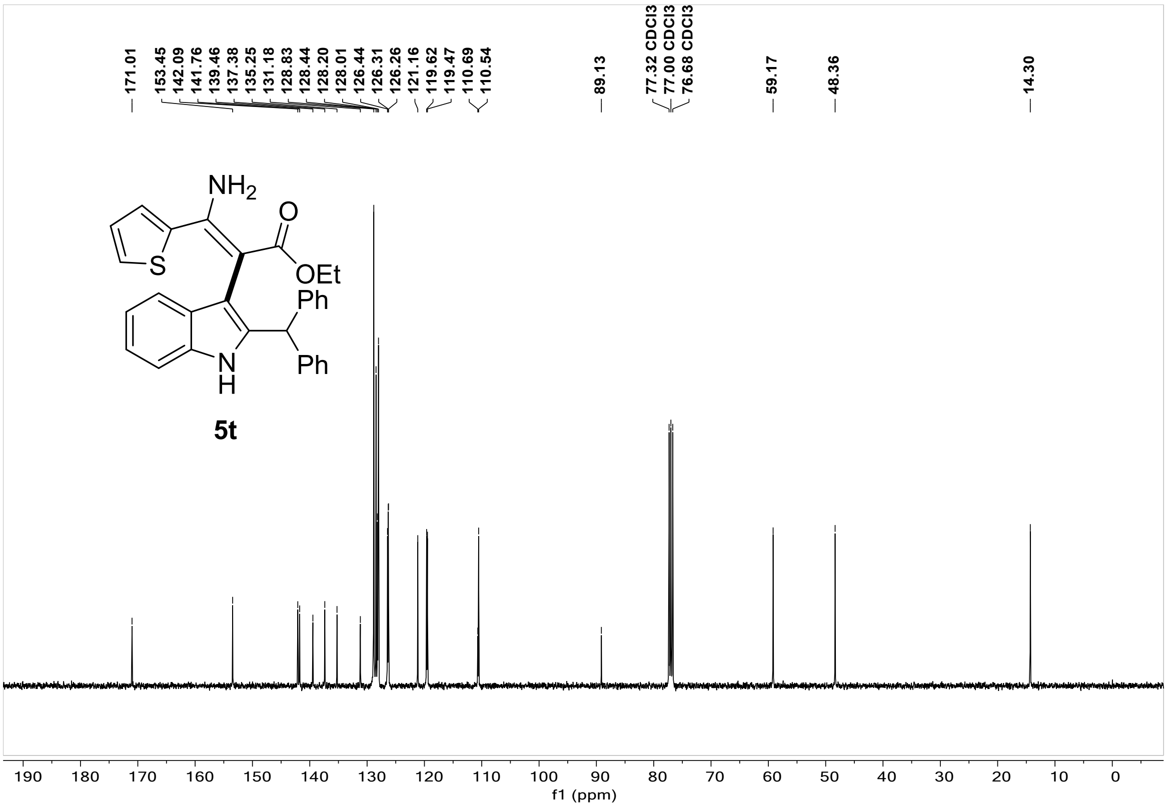
171.01
153.45
142.09
141.76
139.46
137.38
135.25
131.18
128.83
128.44
128.20
128.01
126.44
126.31
126.26
121.16
119.62
119.47
110.69
110.54
89.13
77.32 CDCl3
77.00 CDCl3
76.68 CDCl3
59.17
48.36
14.30
NH2
O
OEt
S
Ph
Ph
N
H
5t
190 180 170 160 150 140 130 120 110 100 90 80 70 60 50 40 30 20 10 0
f1 (ppm)

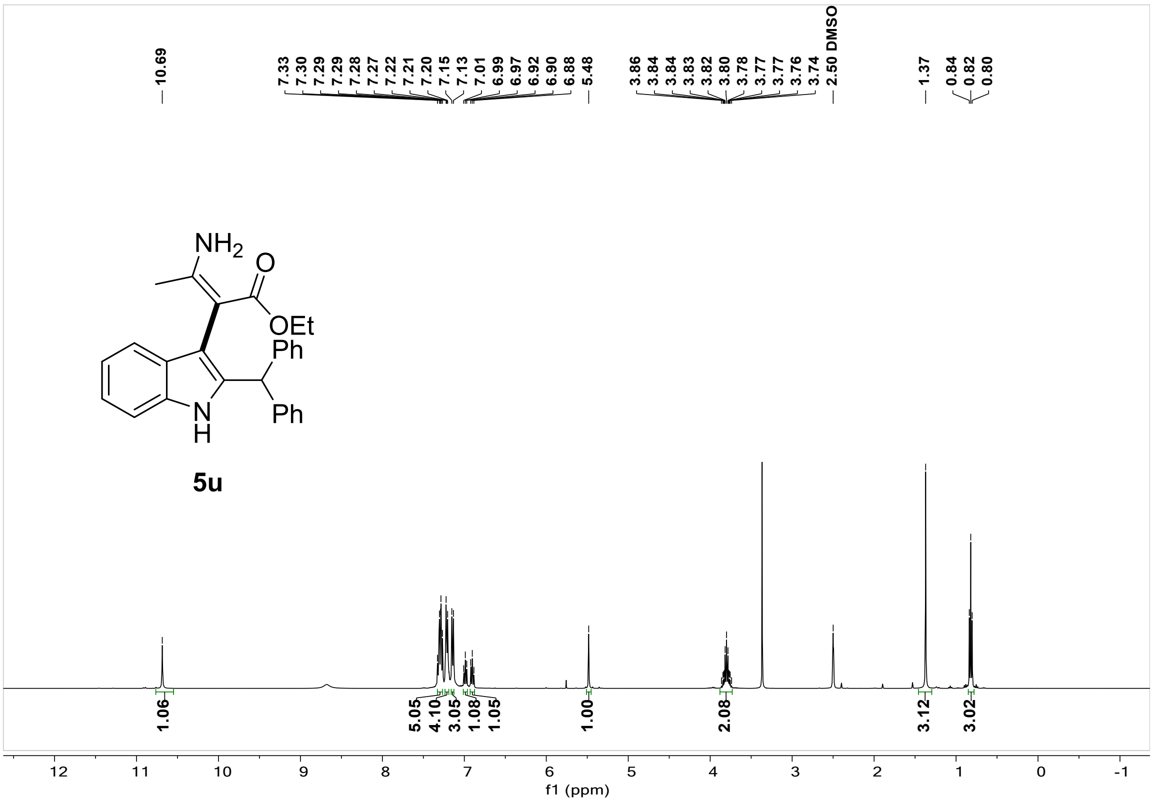
10.69
7.33
7.30
7.29
7.28
7.27
7.22
7.21
7.20
7.15
7.13
7.01
6.99
6.97
6.92
6.90
6.88
5.48
3.86
3.84
3.84
3.83
3.82
3.80
3.78
3.77
3.77
3.76
3.74
2.50 DMSO
1.37
0.84
0.82
0.80
NH2
O
OEt
Ph
Ph
N
H
5u
1.06
5.05
4.10
3.05
1.08
1.05
1.00
2.08
3.12
3.02
12 11 10 9 8 7 6 5 4 3 2 1 0 -1
f1 (ppm)

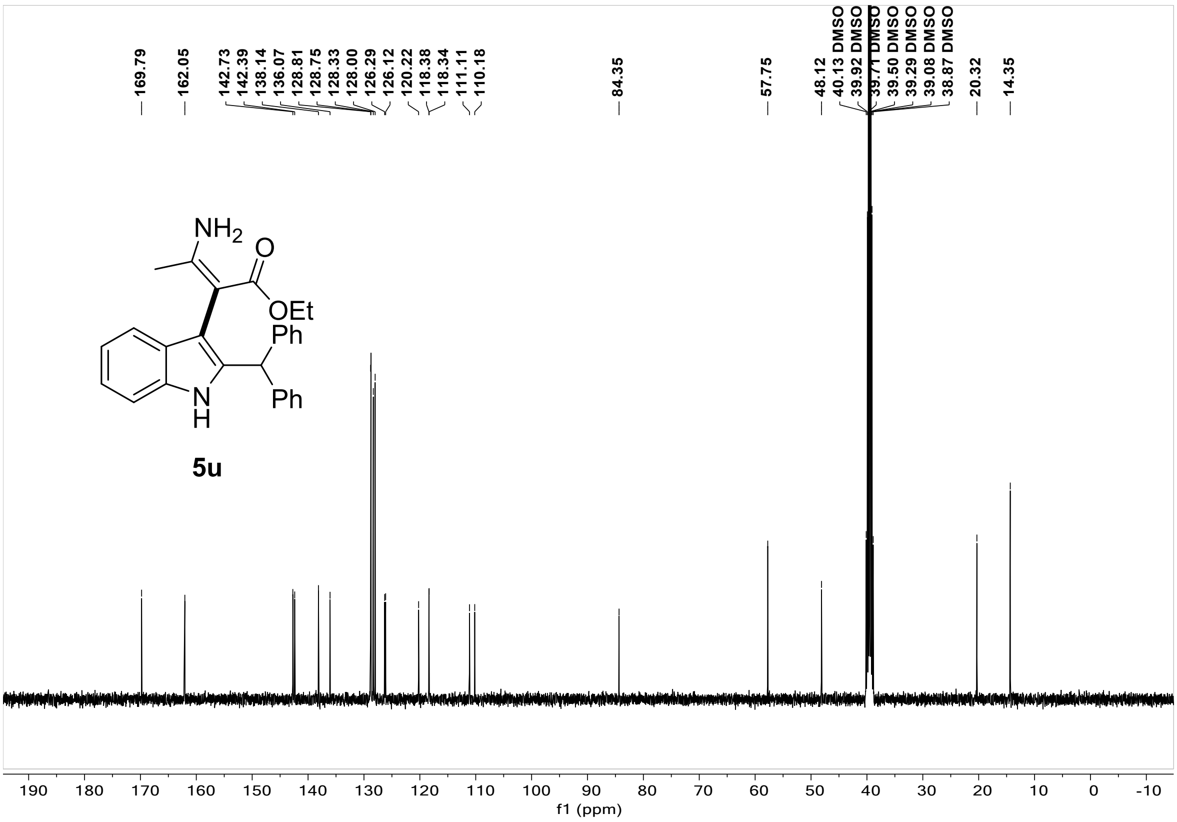
169.79
162.05
142.73
142.39
138.14
136.07
128.81
128.75
128.33
128.00
126.29
126.12
120.22
118.38
118.34
111.11
110.18
84.35
57.75
48.12
40.13 DMSO
39.92 DMSO
39.71 DMSO
39.50 DMSO
39.29 DMSO
39.08 DMSO
38.87 DMSO
20.32
14.35
NH2
O
OEt
Ph
Ph
N
H
5u
190 180 170 160 150 140 130 120 110 100 90 80 70 60 50 40 30 20 10 0 -10
f1 (ppm)

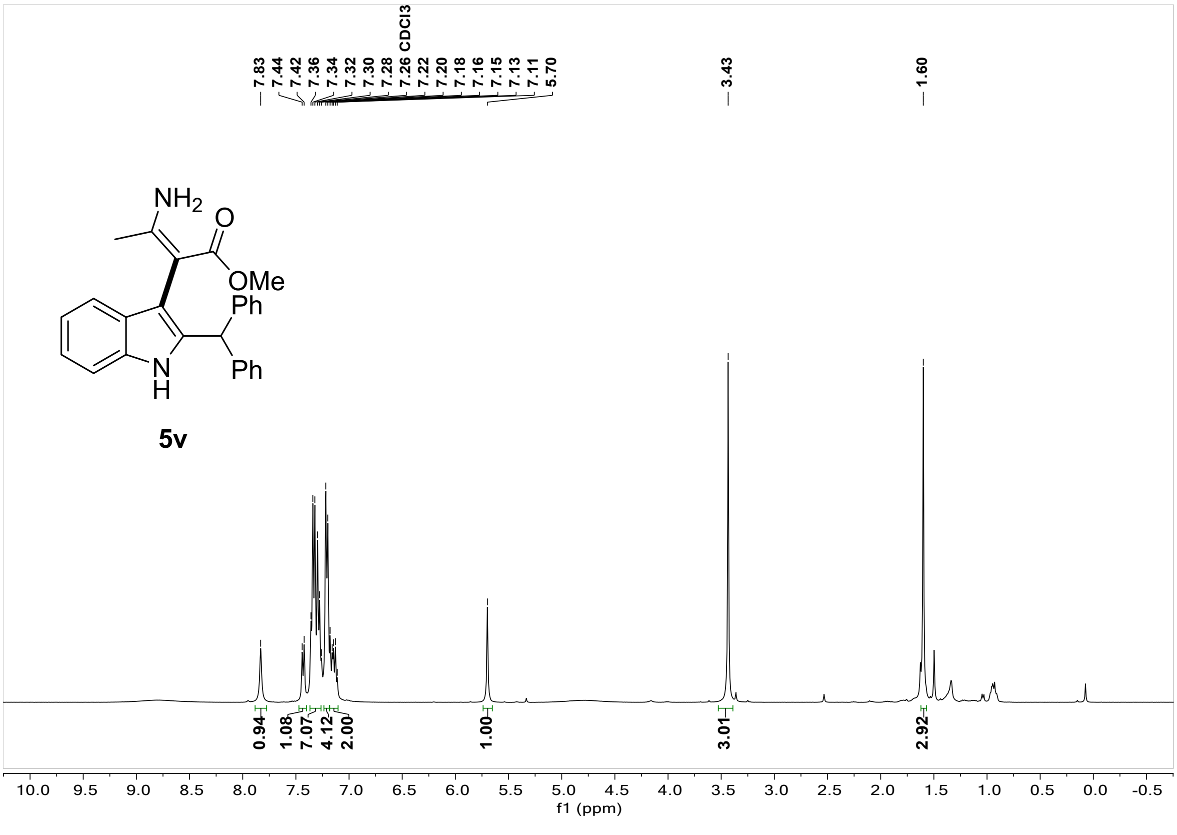
7.83
7.44
7.42
7.36
7.34
7.32
7.30
7.28
7.26 CDCl3
7.22
7.20
7.18
7.16
7.15
7.13
7.11
5.70
3.43
1.60
NH2
O
OMe
Ph
Ph
N
H
5v
0.94
1.08
7.07
4.12
2.00
1.00
3.01
2.92
10.0 9.5 9.0 8.5 8.0 7.5 7.0 6.5 6.0 5.5 5.0 4.5 4.0 3.5 3.0 2.5 2.0 1.5 1.0 0.5 0.0 -0.5
f1 (ppm)

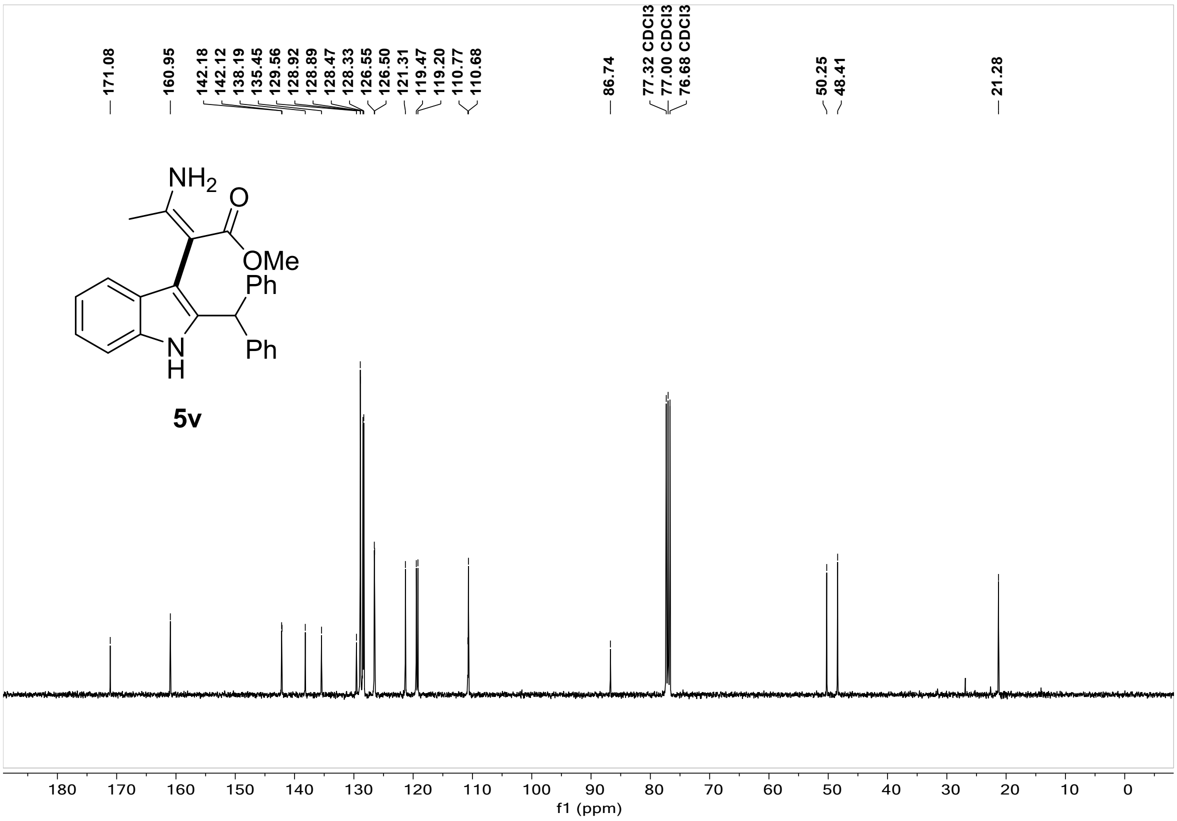
171.08
160.95
142.18
142.12
138.19
135.45
129.56
128.92
128.89
128.47
128.33
126.55
126.50
121.31
119.47
119.20
110.77
110.68
86.74
77.32 CDCl3
77.00 CDCl3
76.68 CDCl3
50.25
48.41
21.28
NH2
O
OMe
Ph
N
H
Ph
5v
f1 (ppm)

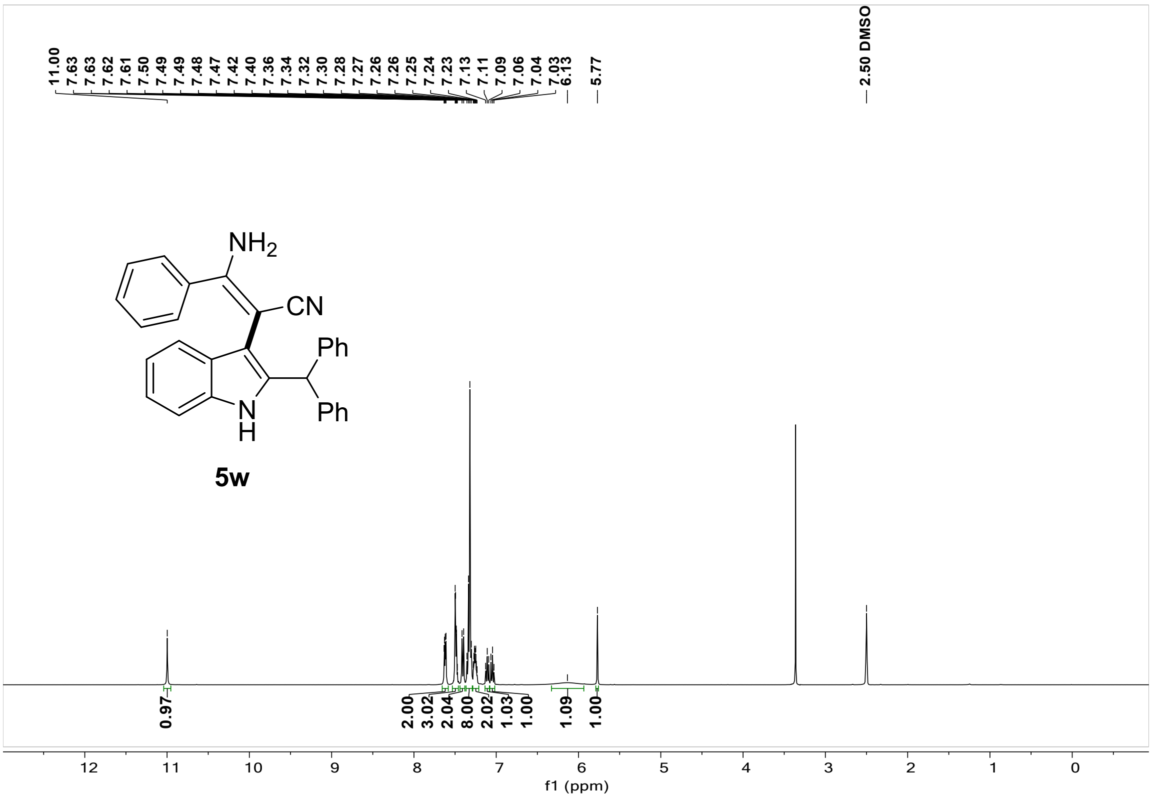
11.00
7.63
7.63
7.62
7.61
7.50
7.49
7.49
7.48
7.47
7.42
7.40
7.36
7.34
7.32
7.30
7.28
7.27
7.26
7.26
7.25
7.24
7.23
7.13
7.11
7.09
7.06
7.04
7.03
6.13
5.77
2.50 DMSO
NH2
CN
Ph
N
H
Ph
5w
0.97
2.00
3.02
2.04
8.00
2.02
1.03
1.00
1.09
1.00
f1 (ppm)

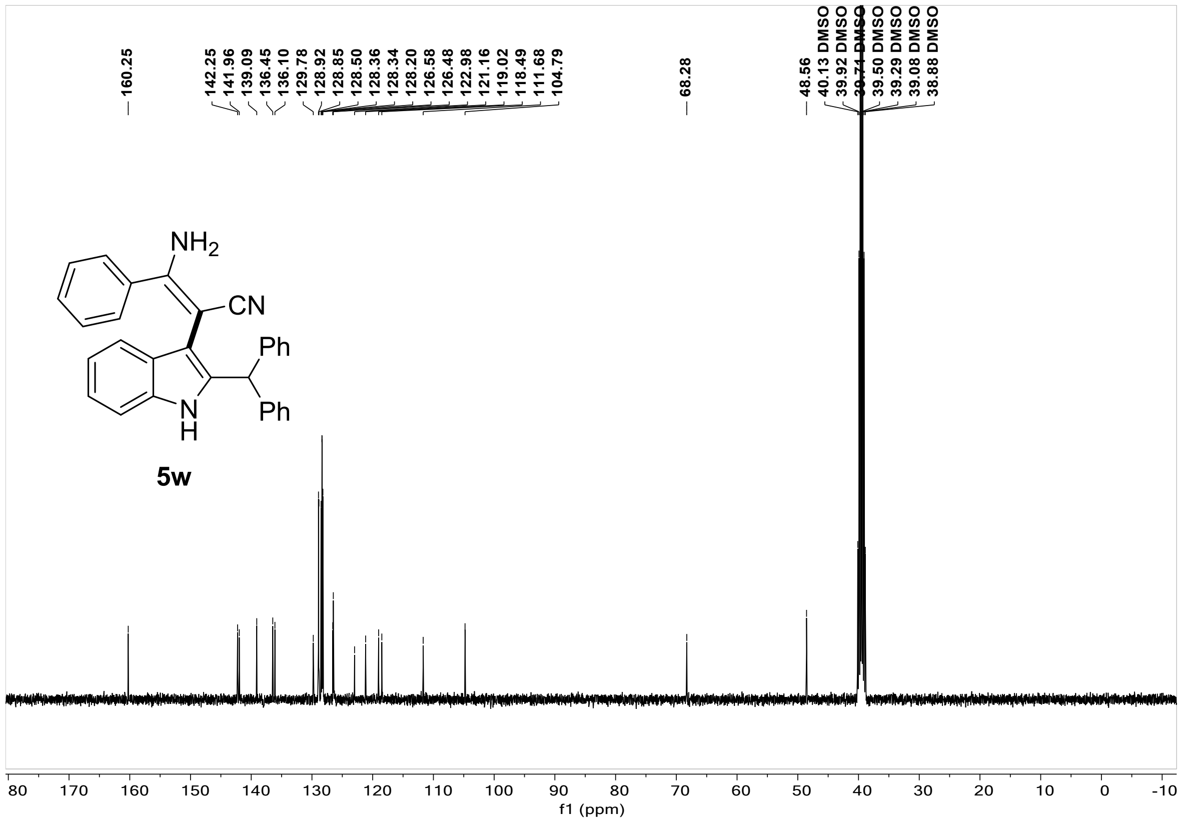
160.25
142.25
141.96
139.09
136.45
136.10
129.78
128.92
128.85
128.50
128.36
128.34
128.20
126.58
126.48
122.98
121.16
119.02
118.49
111.68
104.79
68.28
48.56
40.13 DMSO
39.92 DMSO
39.71 DMSO
39.50 DMSO
39.29 DMSO
39.08 DMSO
38.88 DMSO
NH2
CN
Ph
Ph
N
H
5w
f1 (ppm)

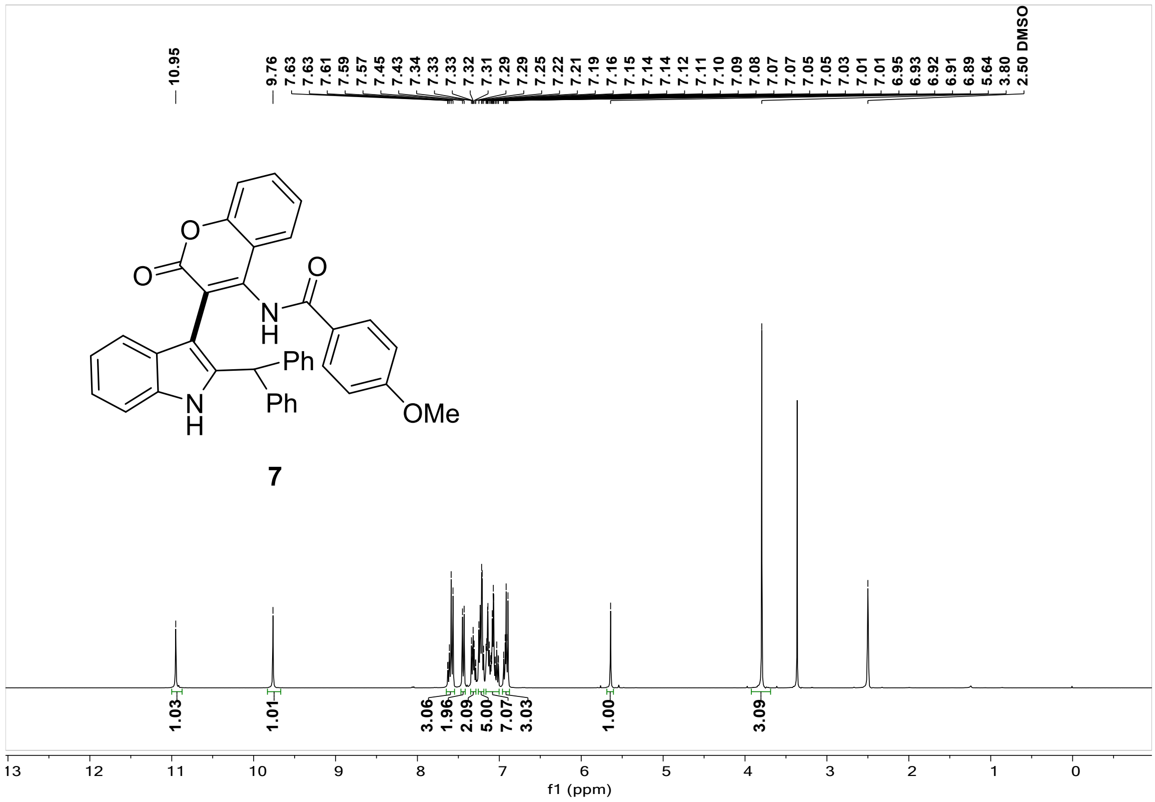
10.95
9.76
7.63
7.63
7.61
7.59
7.57
7.45
7.43
7.34
7.33
7.33
7.32
7.31
7.29
7.29
7.25
7.22
7.21
7.19
7.16
7.15
7.14
7.14
7.12
7.11
7.10
7.09
7.08
7.07
7.07
7.05
7.05
7.03
7.01
7.01
6.95
6.93
6.92
6.91
6.89
5.64
3.80
2.50 DMSO
O
O
O
N
H
Ph
Ph
N
H
OMe
7
1.03
1.01
3.06
1.96
2.09
5.00
7.07
3.03
1.00
3.09
f1 (ppm)

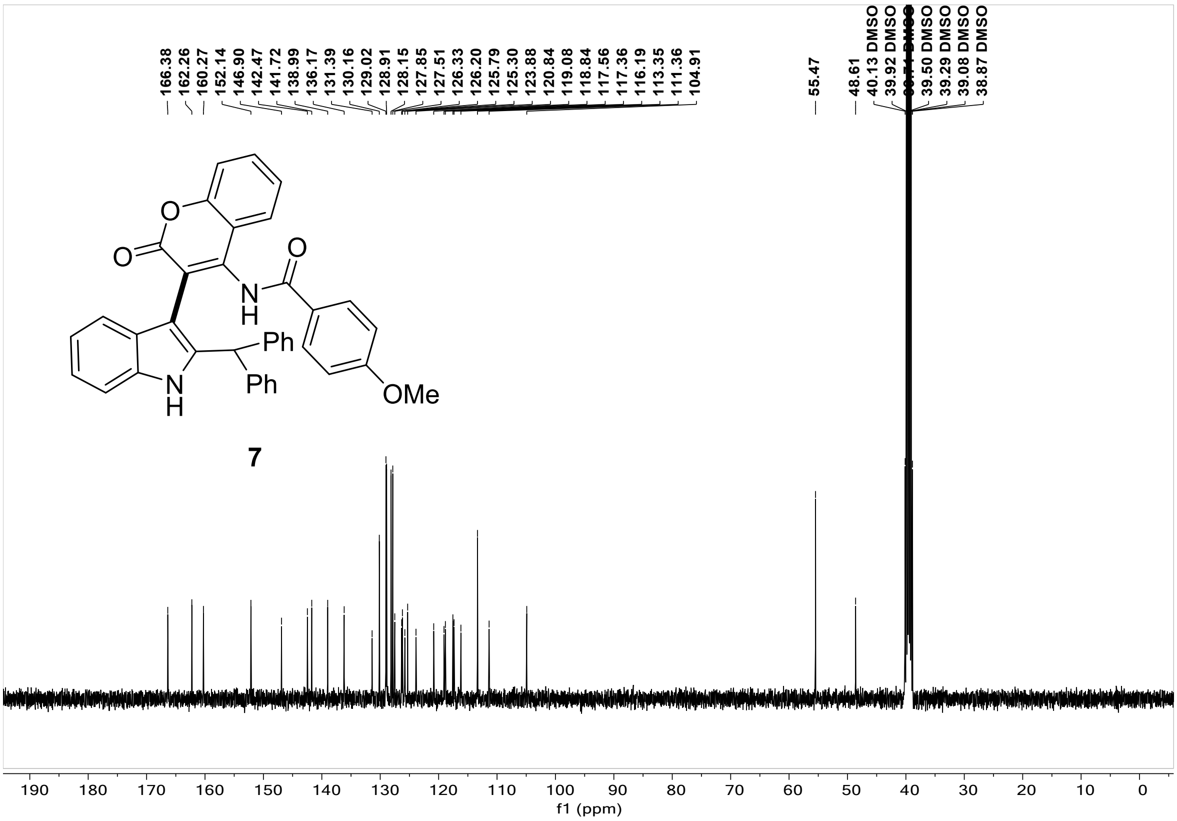
166.38
162.26
160.27
152.14
146.90
142.47
141.72
138.99
136.17
131.39
130.16
129.02
128.91
128.15
127.85
127.51
126.33
126.20
125.79
125.30
123.88
120.84
119.08
118.84
117.56
117.36
116.19
113.35
111.36
104.91
55.47
48.61
40.13 DMSO
39.92 DMSO
39.50 DMSO
39.29 DMSO
39.08 DMSO
38.87 DMSO
O
O
N
H
Ph
Ph
N
H
OMe
7
f1 (ppm)

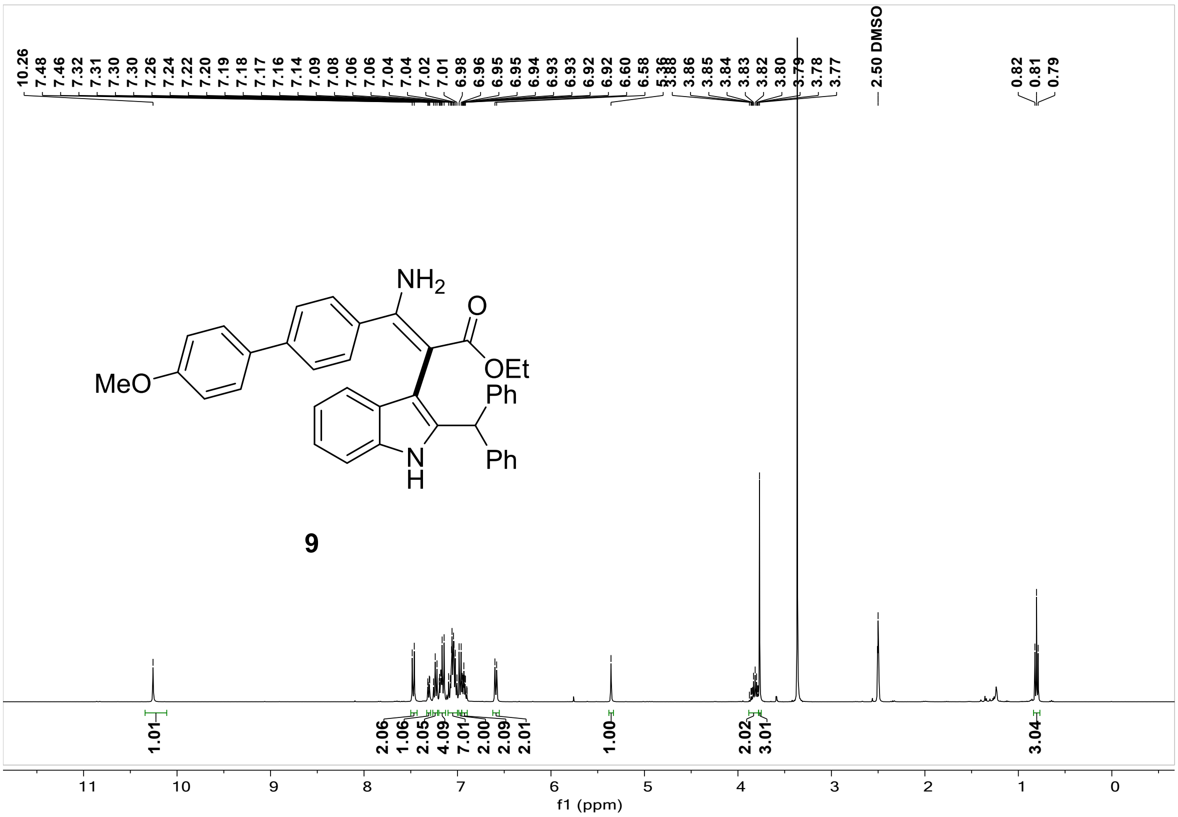
10.26
7.48
7.46
7.32
7.31
7.30
7.26
7.24
7.22
7.20
7.19
7.18
7.17
7.16
7.14
7.09
7.08
7.06
7.06
7.04
7.04
7.02
7.01
6.98
6.96
6.95
6.95
6.94
6.93
6.93
6.92
6.92
6.60
6.58
5.36
3.88
3.86
3.85
3.84
3.83
3.82
3.80
3.78
3.77
2.50 DMSO
0.82
0.81
0.79
NH2
O
OEt
MeO
Ph
Ph
N
H
9
1.01
2.06
1.06
2.05
4.09
7.01
2.00
2.09
2.01
1.00
2.02
3.01
3.04
f1 (ppm)

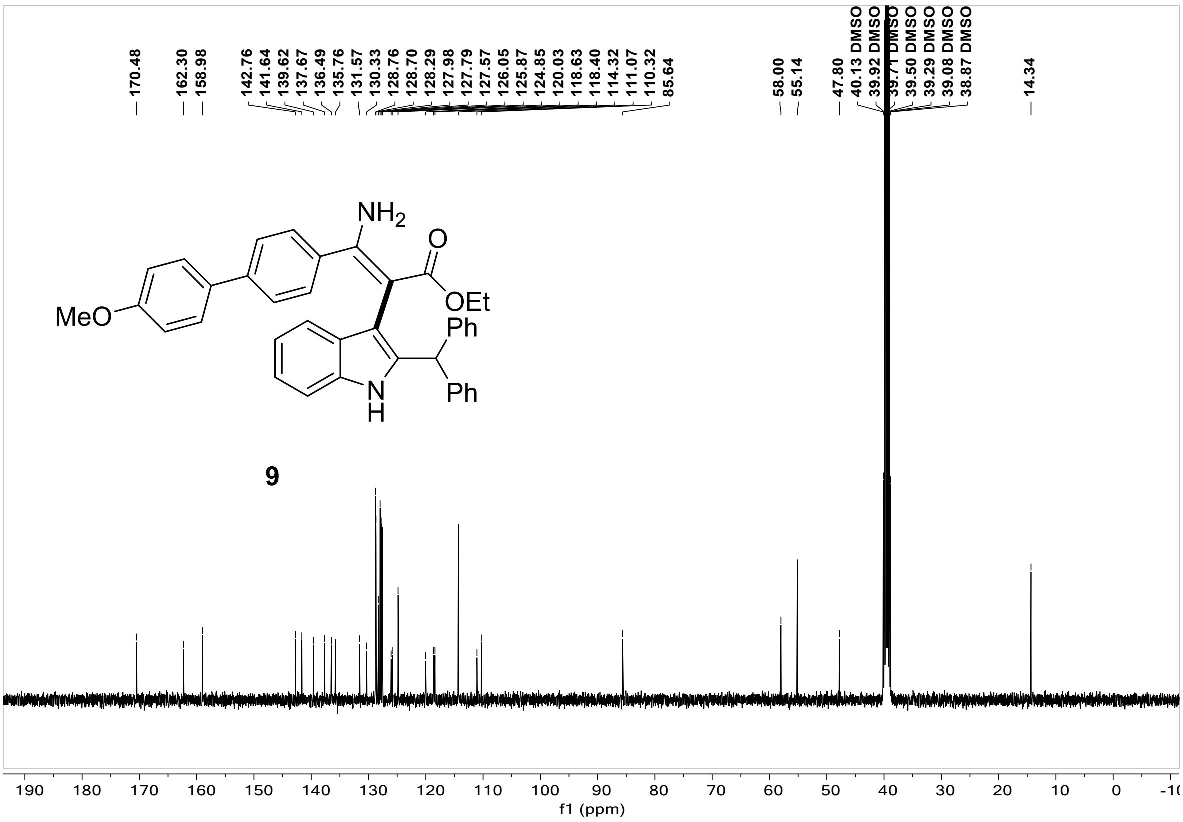
170.48
162.30
158.98
142.76
141.64
139.62
137.67
136.49
135.76
131.57
130.33
128.76
128.70
128.29
127.98
127.79
127.57
126.05
125.87
124.85
120.03
118.63
118.40
114.32
111.07
110.32
85.64
58.00
55.14
47.80
40.13 DMSO
39.92 DMSO
39.71 DMSO
39.50 DMSO
39.29 DMSO
39.08 DMSO
38.87 DMSO
14.34
NH2
O
OEt
MeO
Ph
Ph
N
H
9
f1 (ppm)
